# A Gold Carbene Manifold to Prepare Fused γ‐Lactams by Oxidative Cyclisation of Ynamides

**DOI:** 10.1002/chem.201804378

**Published:** 2018-10-30

**Authors:** Fernando Sánchez‐Cantalejo, Joshua D. Priest, Paul W. Davies

**Affiliations:** ^1^ School of Chemistry University of Birmingham Birmingham UK

**Keywords:** cyclisation, gold, oxidation, ynamide, γ-lactam

## Abstract

Gold‐catalysed oxidative cyclisation reactions of ynamides offer great promise in γ‐lactam synthesis but are limited by preferential over‐oxidation to form α‐keto imides. Evaluating the factors that might limit *N*‐cyclisation pathways led to effective gold‐catalysed conditions that allow access to different fused γ‐lactams on changing the ynamide *N*‐substituent and accommodate previously incompatible substitution patterns. New and efficient methods for the synthesis of functionalised 3‐aryl indoles and cyclohepta[c]pyrrol‐1‐one derivatives are presented. These conditions illustrate the complementarity of gold catalysis to other metals.

γ‐Lactams are common motifs in bioactive natural products and pharmaceuticals.^1^ The cyclisation of acetamido metal carbenes **A** by reaction at *N*‐tethered C−H or C−C bonds provides a powerful hydrocarbon‐forming strategy allowing access to different types of lactam (Scheme 1 a).^2^ Divergent pathways are often accessible in such processes and controlled by modifying the substrate structure, with α‐proton, ‐methyl, or ‐electron‐withdrawing groups commonly encountered, and by tuning the reaction conditions and choice of catalyst.^3^, ^4^, ^5^ The wider application of carbene‐based γ‐lactam formation is limited by the most common precursors, diazoacetamides **G** (Scheme 1 b). The need to install sacrificial and highly reactive diazo groups is deleterious for step and process economy, safe‐handling, and wider compatibility with desirable structural or functional features.

**Scheme 1:**
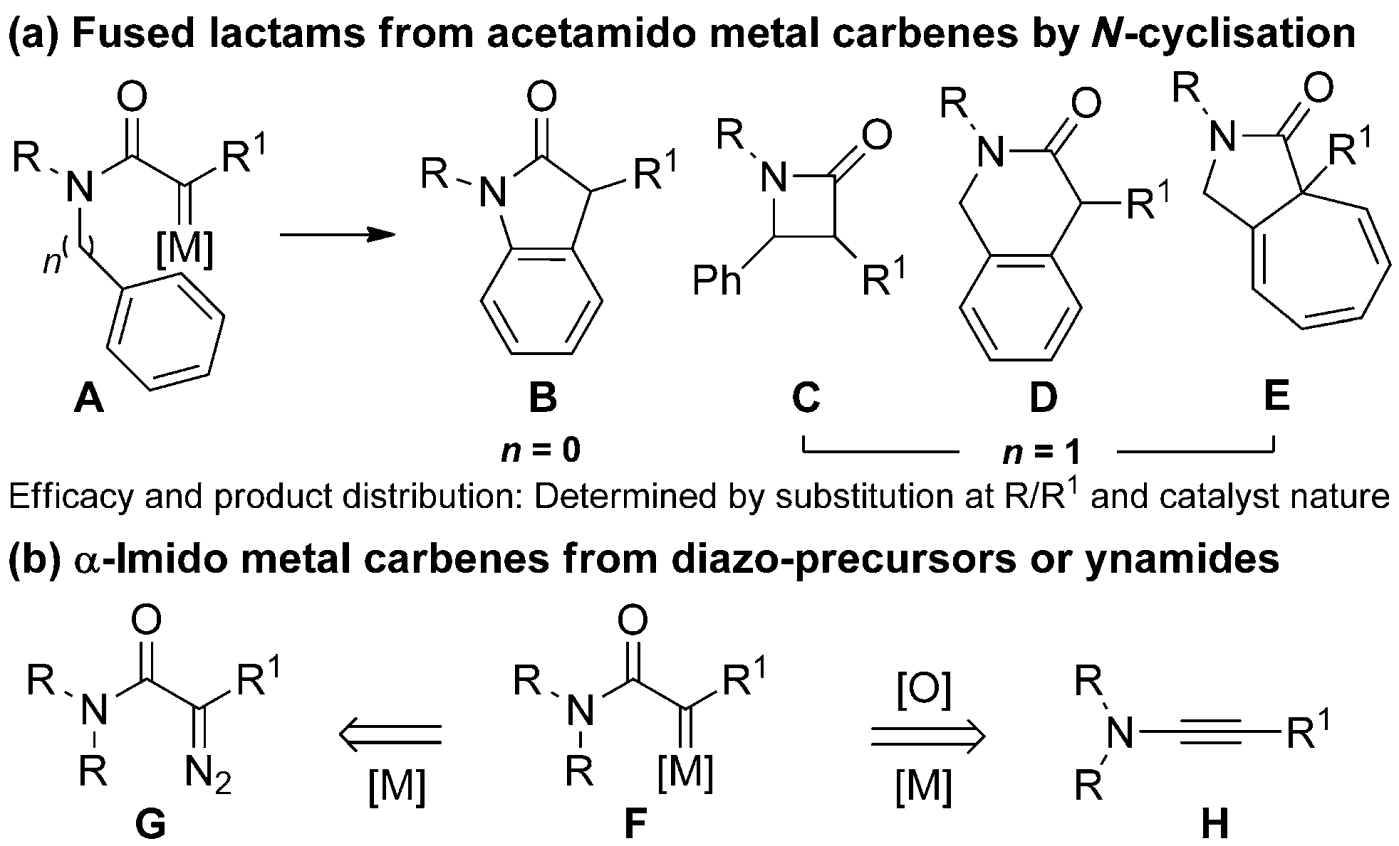
(a) Utility of acetamido metal carbenes to access lactam motifs; (b) ynamide versus diazo method to access metal carbene reactivity.

Metal‐catalysed oxidation of ynamides **H** provides a diazo‐free access to α‐imido carbenoids (Scheme 1 b).^6^, ^7^ Ynamides are readily prepared with diverse substitution patterns^8^ while gold catalysis displays excellent functional group tolerance.^9^ Together this offers great promise for lactam synthesis. However, gold‐catalysed oxidative cyclisations do not currently provide a general alternative to the use of diazoacetamide precursors.^10^

Generating sub‐stoichiometric quantities of electrophilic organometallics from a (super)stoichiometric nucleophilic oxidant presents an intrinsic challenge as over‐oxidation of the ynamide to α‐keto imides can dominate.6a Furthermore, *N*‐aryl and *N*‐benzyl substituents are used in gold‐catalysed oxidative processes without forming lactams indicating that *N*‐cyclisation of the organogold species is slow when compared to diazoacetamide processes.6a, 11

Reported gold‐catalysed oxidative *N*‐cyclisations are sensitive to the choice of carbene α‐substituent: Over‐oxidation predominates over cyclisation when an *N*‐allyl ynamide has an electron‐rich substituent, or when an *N*‐phenyl ynamide has a substituent other than hydrogen (Scheme 2).10a,10b A three‐component coupling (Scheme 2, bottom) further illustrates the challenge of controlling competing pathways: Intermolecular coupling is favoured over *N*‐cyclisation, and over‐oxidation again dominates when the ynamide bears an α‐electron donor‐group.11d, 12

**Scheme 2:**
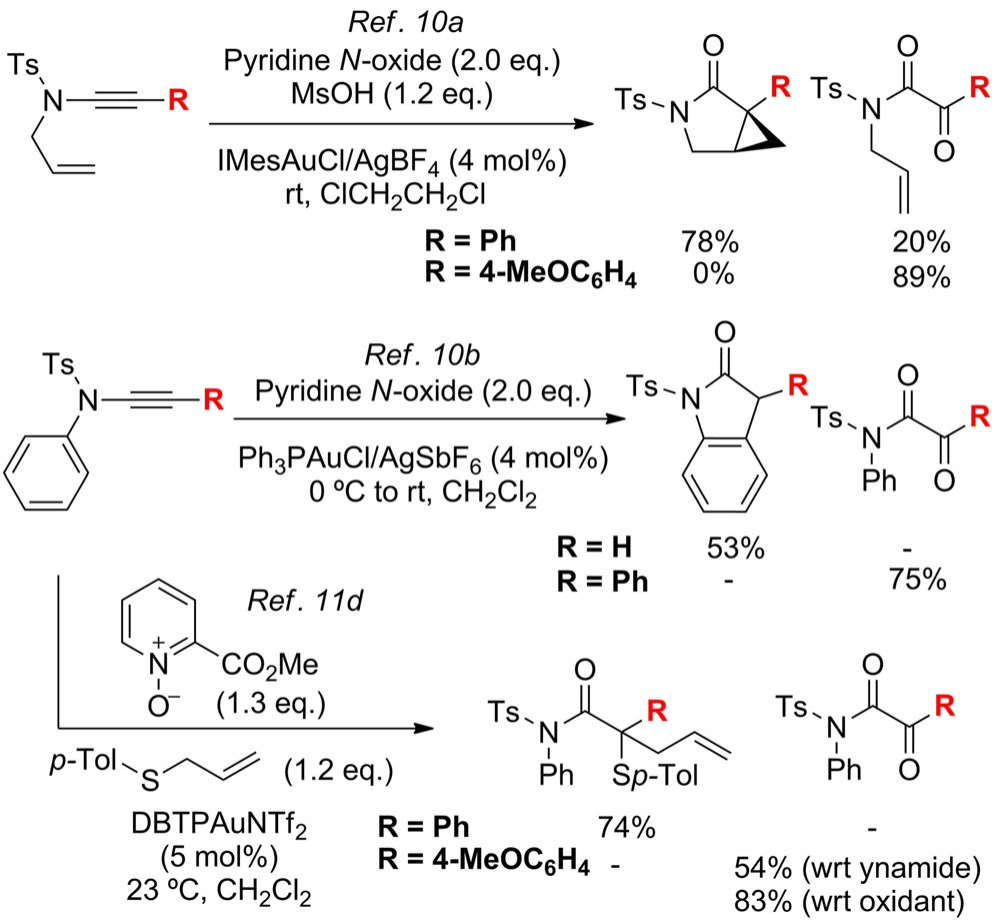
Gold‐catalysed oxidative reactions: The impact of ynamide C‐substitution on α‐ketoimide formation versus desirable inter‐ or intramolecular pathways. DTBP=tris(2,4‐di‐*t*‐butylphenyl)phosphite.

This apparent proclivity for over‐oxidation under gold catalysis has seen Rh^I [13]^ and Zn^II [14]^ catalysed oxidative *N*‐cyclisations developed to access cyclopropanation, metathesis and Friedel–Craft pathways. As the metal will strongly influence reaction mode, process efficiency and substrate generality, our interest in accessing gold carbene reactivity from ynamides^15^ prompted us to explore the aspects that appear to limit wider use of gold‐catalysis in this area. We show here that gold‐catalysed oxidative *N*‐cyclisation can be considered as a general and productive tool for fused γ‐lactam synthesis that complements and diverges from current methods employing gold and other metal catalysts.

We investigated the formation of oxindoles over α‐ketoimides by gold‐catalysed oxidation of an internal *N*‐phenyl ynamide. We hypothesised that, while competing over‐oxidation exacerbates the problem, oxidative *N*‐cyclisations of ynamides are challenging because the ynamide‐derived carbenoids are geometrically and electronically compromised against cyclisation.

Application of the ynamide approach must consider that the vinyl gold carbenoid **J** that precedes the gold carbene **K** is also a functional electrophile (Scheme 3).10a, 11d The 5‐*endo*‐*trig* cyclisation is required from **J** 16 for an *N*‐phenyl ynamide **I**, indicating that formation of gold carbene **K**/**K′** might be needed prior to cyclisation. In contrast, oxidation can occur from either **J** or **K**/**K′**. Furthermore, the electron‐withdrawing *N*‐substituent that stabilises the ynamide can reduce the relative rate of cyclisation of the gold carbene relative to diazoacetamide‐derived metal carbenes:^17^ The *s‐cis* and *s‐trans* amide rotamers **K**/**K′** will have significantly different localised dipole moments, and the required *s‐cis* rotamer **K′** is predicted to be disfavoured;^18^ a second electron‐withdrawing group on nitrogen also reduces the nucleophilicity of the aromatic ring.

**Scheme 3:**
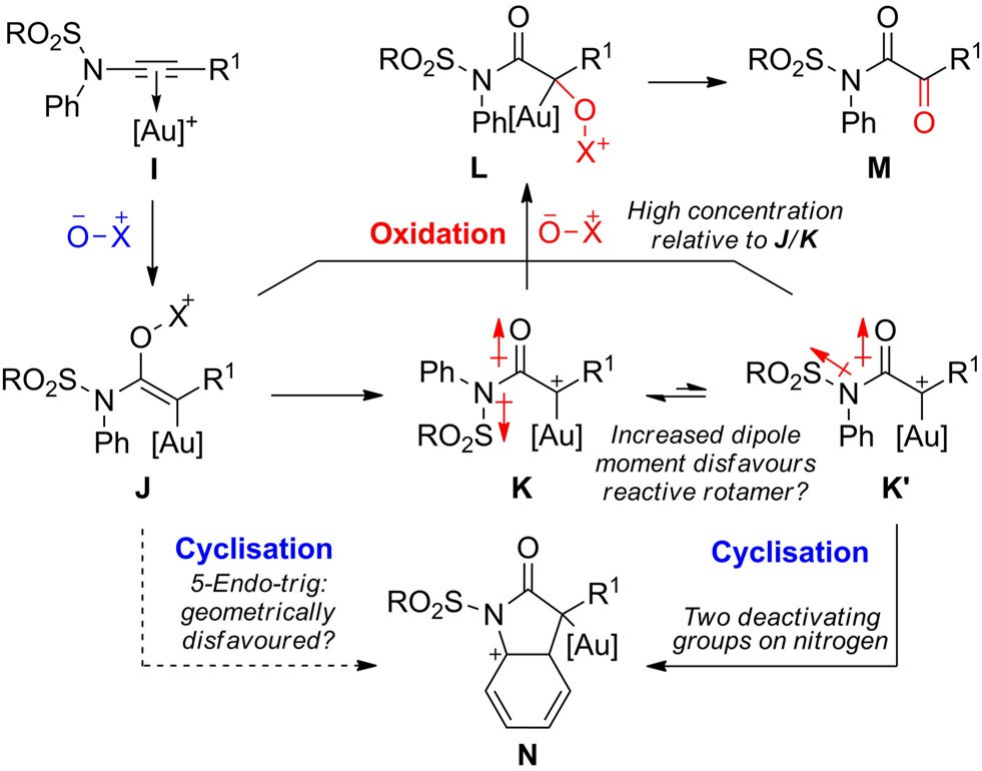
A proposed model to explain the challenge of oxidative *N*‐cyclisation from ynamides. While oxidation is viable from all organometallic species, the desired cyclisation needs access to a disfavoured rotamer of the gold carbene.

With this model in mind, we found that oxindole **3 a** could indeed be accessed in high yield from *N*‐phenyl‐*N*‐(phenylethynyl)methanesulfonamide **1 a** when using relatively dilute conditions at elevated temperature, a bulky electron‐rich ligand on gold, and a polar solvent such as nitromethane or acetonitrile (Scheme 4, see the Supporting Information). Yields dropped off sharply with more electron‐deficient and less bulky ligands. Methylpicolinate‐derived oxidant **2 a** proved effective at near stoichiometric levels to the ynamide. 2‐Bromopyridine *N*‐oxide **2 e** 11c was similarly effective but degrades on standing at room temperature, leaving **2 a** as a more practical choice. Other *N*‐oxides used in alkyne oxidation, such as 8‐ethylquinoline *N*‐oxide **2 b**,^19^ were inferior here.

**Scheme 4:**
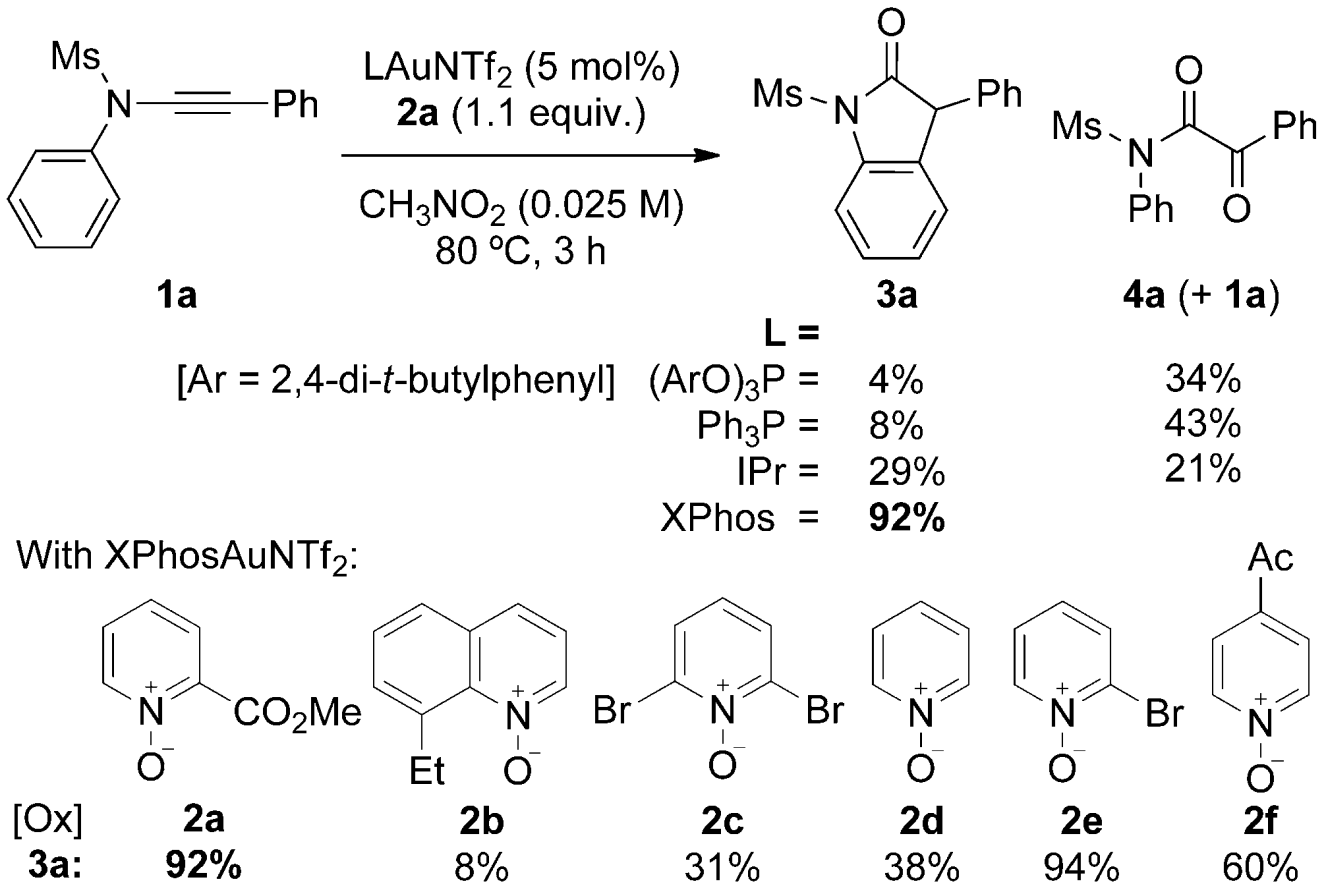
Effect of ligand and oxidant on the gold‐catalysed oxidative cyclisation of an internal ynamide to form a 3‐phenyl oxindole.

The selectivity for *N*‐cyclisation appeared consistent with our model: an electron‐rich ligand on gold and heating will both aid elimination of the pyridine nucleofuge to form gold carbene **K** from the potentially unproductive **J** (Scheme 3).^20^ The reactive rotamer **K′** is rendered more accessible due to the polar reaction media, which ameliorates the impact from dipole discrepancy, and heating, which aids interconversion between amide rotamers. Over‐oxidation to **L** is slowed by use of a bulky and deactivated N‐oxide at higher dilution.

Exploring the transformation more widely (Scheme 5) showed that inductively and mesomerically electron‐donating and electron‐withdrawing groups were tolerated on both the *N*‐aryl (**3 b**–**f**,**h**) and C‐terminus positions (**3 g**–**m**). The ready incorporation of desirable and synthetically useful functionality, including thioether **3 d**, aryl iodide **3 h** and esters **3 e**,**i**,**j**, highlights the tolerance of the gold‐catalysed method.

**Scheme 5:**
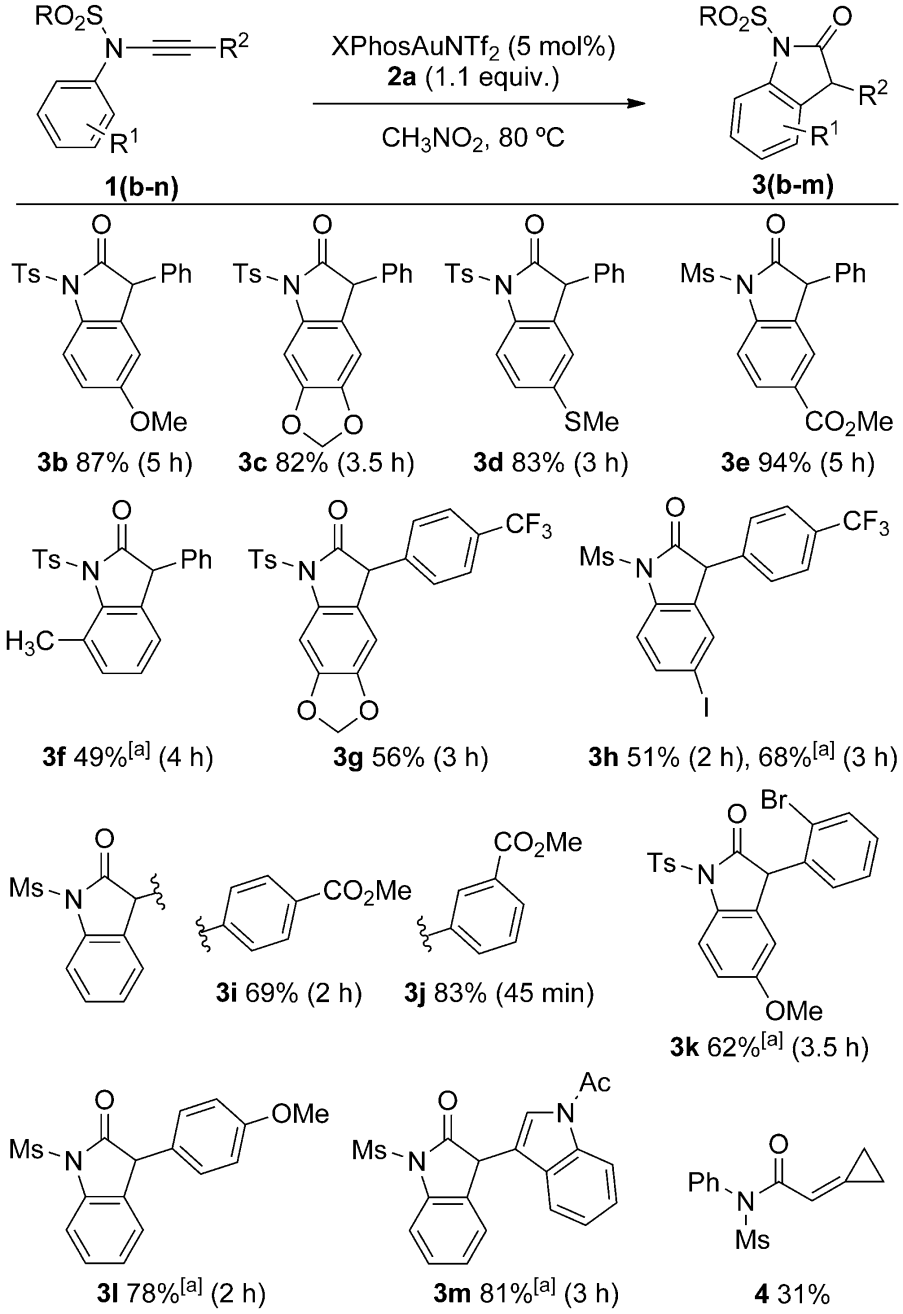
Substrate scope for the oxindole synthesis. Isolated yields after column chromatography. [a] **2 a** added portion‐wise (see the Supporting Information for details).

Formation of the oxindole **3 l** is noteworthy as electron‐donating ynamide substituents such as the *p*‐methoxybenzene group were either not tolerated or not assessed in diverse ynamide oxidation processes (Scheme 2).10a,10c, 11a,11c,11d Steric bulk at the *ortho*‐positions of both substituents can also be accommodated (**3 f**/**k**). In both cases, over‐oxidation does start to compete, but simply adding the oxidant portion‐wise over the course of the reaction recovers the good yields of oxindoles (see the Supporting Information for details). The 3‐(indol‐3‐yl) oxindole motif (**3 m**) common within bioactive natural products can be accessed in good yield as a result. Alkyl substituents on the ynamide were not tolerated; 1,2‐CH insertion dominates even when leading to a strained system (**1 n**→**4**).6a Deprotection of *N*‐sulfonyl lactams is well established and **3 f** underwent desulfonylation with sodium naphthalenide^21^ to give the NH oxindole in 69 % yield (see the Supporting Information).

*N*‐Allyl ynamide **5** reacted smoothly under our conditions to give 3‐azabicyclo[3.1.0]hexane **6** in high yield, contrasting the exclusive over‐oxidation previously observed with this electron‐rich substituent (Scheme 6, cf. Scheme 2).10a, 13, 22 Achieving this outcome by adding the oxidant in a single portion highlights the greater challenge in cyclizing onto *N*‐aryl rather than *N*‐allyl groups.

**Scheme 6:**
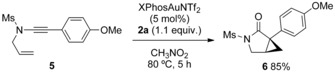
Formation of 3‐azabicyclo[3.1.0]hexane derivative with an electron‐donating α‐substituent.

Ye's group avoided over‐oxidation in a synthesis of dihydrooxyisoquinolinones from *N*‐benzylated ynamides by using Zn(OTf)_2_. A Friedel–Crafts pathway rather than metal carbene insertion is enforced from the organozinc species.^14^ Having invoked formation of a gold carbene, we were intrigued to see whether this would translate into different reactivity with *N*‐benzylated substrates.^18^, ^23^

An oxidative Büchner‐type cyclopropanation and electrocyclic ring‐opening process was observed using *p*‐methoxy benzyl ynamides **7 a**–**d** to give [5.3.0]azabicycles **8 a**–**d** under our conditions (Scheme 7). No β‐lactam formation by CH insertion at the benzylic position was seen, possibly due to the deactivating effect of two electron‐withdrawing groups on nitrogen.3d No conversion to other products was seen when **8 a** was re‐subjected to the catalysis conditions, or exposed to trifluoroacetic acid, indicating an irreversible cyclopropanation.^23^, ^24^ A dynamic cycloheptatriene to norcaradiene relationship was apparent from NMR spectroscopy (see the Supporting Information). Norcaradiene **8 a“** was trapped out with PTAD at lower temperature to access polycycle **9** in good yield. Reaction of **7 e** unveils the cationic character of gold carbenes with the *m*‐methoxy substituent stabilising the Wheland intermediate en route to 3‐oxy‐1,4‐dihydroisoquinoline **10**.

**Scheme 7:**
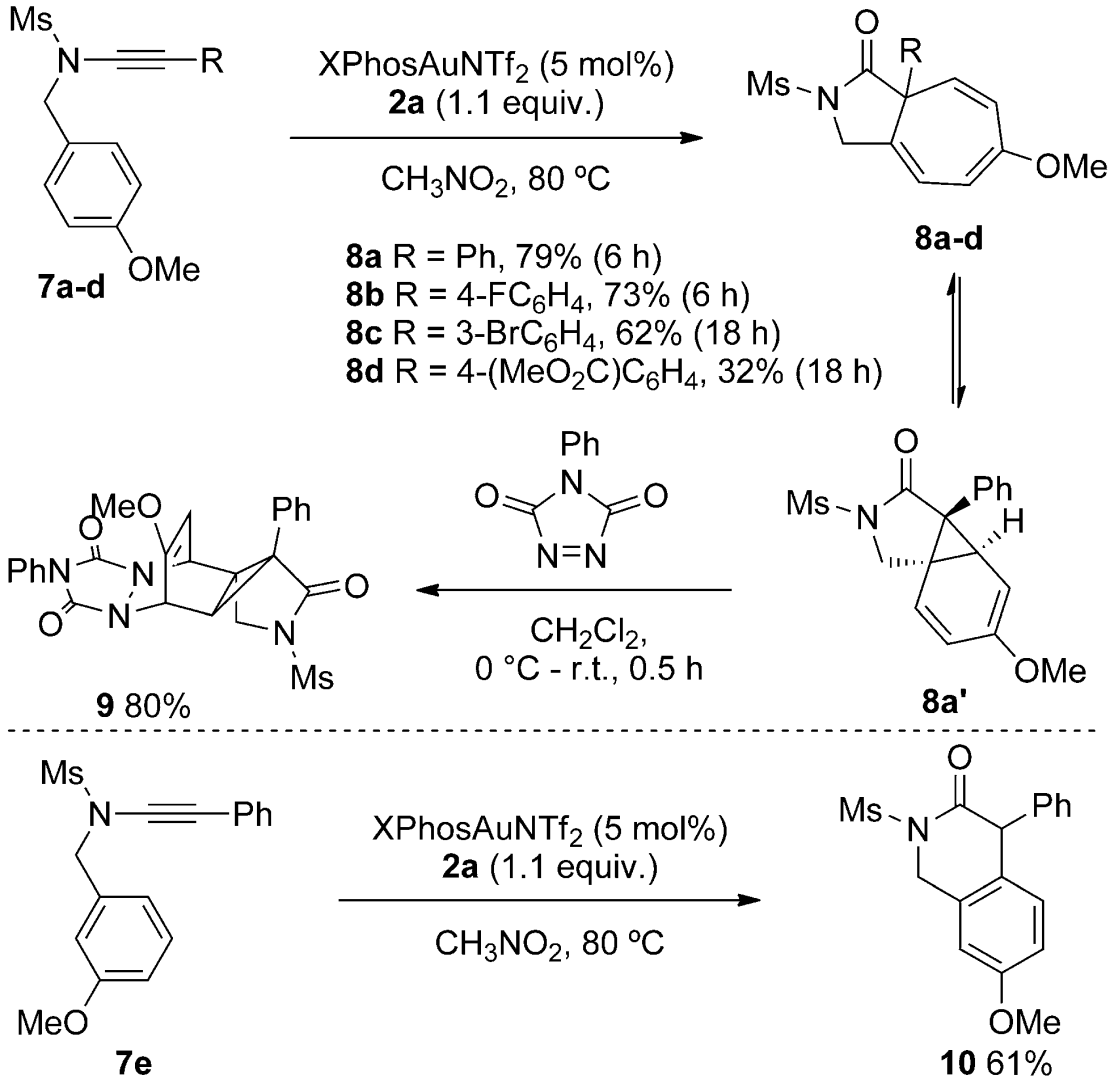
Top: Oxidative Büchner‐type reaction of ynamides and trapping the norcaradiene valence tautomer. Bottom: Use of electronic influence to direct a Friedel–Crafts type pathway to form a 3‐oxy‐1,4‐dihydroisoquinoline.

While an ynamide bearing an unsubstituted benzyl group favoured the [5.3.0]azabicycle (29 %) over 3‐oxy‐1,4‐dihydroisoquinoline (5 %), substantial over‐oxidation was observed (see the Supporting Information). The addition of a methyl substituent was sufficient to direct the reaction solely to the cycloheptatriene‐fused lactam **8 f** in preparatively useful yield (Scheme 8). The allyl enol ether **8 g** was also formed despite the potential for fragmenting spirocyclisation on elimination of an allyl cation from a cationic Wheland intermediate.10c Use of *ortho*‐substituted substrates affords the 7‐substituted products **8 h**/**i**. Some Büchner‐type product **8 j** was even isolated from the mesityl system that requires formation of a highly strained cyclopropane made up of three contiguous quaternary centres. Introducing structural constraints to disfavour ring‐opening saw the fused cyclopropane **8 k** formed in good yield.

**Scheme 8:**
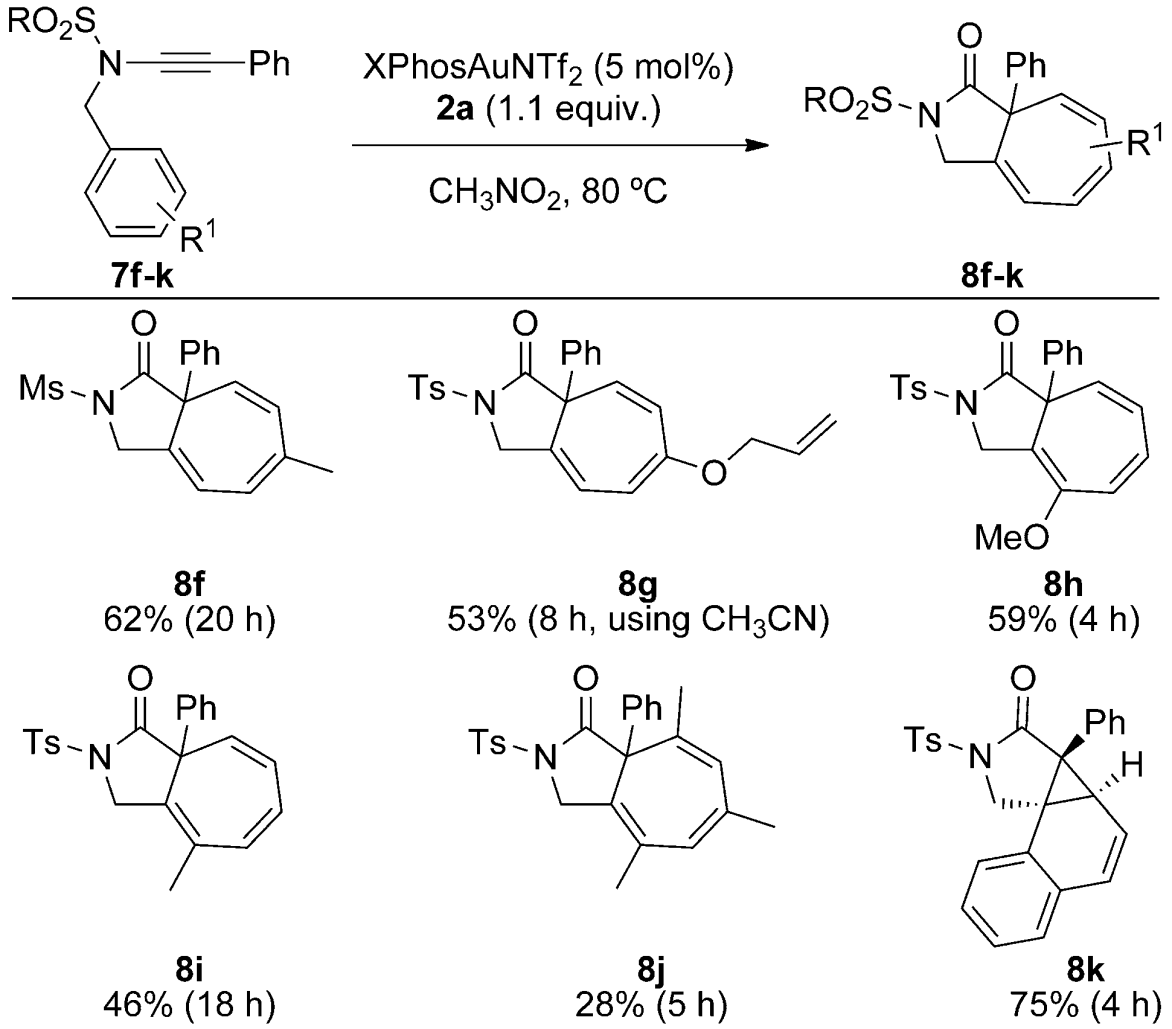
Varying the benzylic substituent for oxidative Büchner‐type reactions.

In conclusion, by considering factors that might limit *N*‐cyclisation pathways in gold‐catalysed oxidative reactions of ynamides, conditions have been developed that allow formation of fused γ‐lactams and tolerate a range of useful functional groups and steric and electronic influences. Different *N*‐heterocycles, including 3‐aryl oxindoles and cyclohepta[c]pyrrol‐1‐one derivatives, are accessed by varying the nitrogen‐substituent. The assembly of a novel sp^3^‐rich framework, **9**, illustrates how this approach can be used to construct molecular complexity in short order from modular and readily assembled precursors. The inherent competing over‐oxidation pathway that has limited gold‐catalysed *N*‐cyclisation reactions of ynamides can be overcome, even allowing use of α‐substituents that favour oxidation in previous reports. Selective formation of [5.3.0]azabicyclic Büchner type products from a gold carbene manifold contrasts with the oxyisoquinoline derivatives accessed from zinc catalysis and the β‐lactams accessed from diazo precursors, illustrating the complementarity of gold‐catalysed methods against other metals. 1All compounds are prepared in the racemic series with relative stereochemistry indicated.


## Conflict of interest

The authors declare no conflict of interest.

## Supporting information

As a service to our authors and readers, this journal provides supporting information supplied by the authors. Such materials are peer reviewed and may be re‐organized for online delivery, but are not copy‐edited or typeset. Technical support issues arising from supporting information (other than missing files) should be addressed to the authors.

Supplementary
