# Supplementary material for: A Gold Carbene Manifold to Prepare Fused γ‐Lactams by Oxidative Cyclisation of Ynamides
Source: Chemistry. 2018 Oct 30;24(65):17215–9. doi: 10.1002/chem.201804378 (PMC6282577; doi:10.1002/chem.201804378)

CHEMISTRY
A European Journal

Supporting Information

A Gold Carbene Manifold to Prepare Fused γ-Lactams by Oxidative Cyclisation of Ynamides

Fernando Sánchez-Cantalejo, Joshua D. Priest, and Paul W. Davies*[a]

chem_201804378_sm_miscellaneous_information.pdf

Author Contributions

F.S. Conceptualization: Supporting; Formal analysis: Equal; Funding acquisition: Supporting; Investigation: Lead; Methodology: Equal; Project administration: Supporting; Validation: Equal; Writing – original draft: Supporting
J.P. Conceptualization: Supporting; Formal analysis: Equal; Investigation: Supporting; Methodology: Equal; Validation: Lead; Writing – original draft: Supporting; Writing – review & editing: Supporting
P.D. Conceptualization: Lead; Formal analysis: Supporting; Funding acquisition: Lead; Investigation: Supporting; Methodology: Equal; Project administration: Lead; Resources: Lead; Supervision: Lead; Validation: Supporting; Writing – original draft: Lead; Writing – review & editing: Lead.

SUPPLEMENTARY INFORMATION

General Experimental 2

Starting Materials 4

Preparative Methods for Starting Materials 5

Preparation of Ynamides 13

Reaction Optimisation 25

Products of the Oxidative Cyclisation Reactions 26

References 40

^{1}H and ^{13}C NMR Spectra of Novel Ynamides and Precursors 42

^{1}H and ^{13}C NMR Spectra of Novel Catalysis Products 79

General Experimental

Commercially available chemicals/reagents were purchased from Aldrich, Acros, Strem, Alfa Aesar and used without further purification, unless reported. All catalysis reactions were carried out under argon in heat gun-dried glassware unless stated otherwise. Anhydrous nitromethane was dried over 4Å molecular sieves. 1,2-DME was dried over 4Å MS and degassed with argon. Dry solvents used were purified using a Pure Solv-MD solvent purification system and were transferred under nitrogen. Sodium napthalenide was prepared by adding sodium metal (2 equiv.) and naphthalene (1 equiv.) to a dried Schlenk tube under argon. Vacuum was applied and the flask backfilled with argon three times. 1,2-DME (dried over 4Å MS and degassed with argon, 2.75 mL per mmol napthalene) was added and the mixture stirred for 45 minutes before use. All reactions were stirred using Teflon coated magnetic stirrer bars. The following cooling baths were used: 0 °C (ice/water), -10 °C (ice/NaCl), -41 °C (dry ice/acetonitrile) and -78 °C (dry ice/acetone). For reactions above room temperature pre-heated paraffin oil baths or metal heating blocks on stirrer hotplates were employed and the temperature was controlled using an external probe. Reactions were monitored by thin layer chromatography using Merck silica gel 60 F^{254} (aluminium support) TLC plates which were developed using standard visualizing agents: UV fluorescence (254 nm), potassium permanganate /Δ or vanillin /Δ. Flash column chromatography was carried out on silica gel (60Å pore size, 40-63 µm). Melting points were measured in open capillaries using Stuart Scientific melting point apparatus and are uncorrected. Infrared spectra were recorded using a Perkin-Elmer Spectrum 100 FTIR spectrometer using an ATR attachment; selected absorbencies (ν_{max}) are reported. NMR Spectra were recorded using Bruker AVIII300 (^{1}H = 300 MHz) and Bruker AVIII400 (^{1}H = 400 MHz, ^{13}C = 101 MHz) spectrometers at ambient temperatures unless otherwise specified, and in commercial (Aldrich or Goss Scientific), TMS free, deuterated solvents. Chemical shifts (δ) are given in ppm relative to TMS and are calibrated using residual solvent peaks. Multiplicity is denoted in ^{1}H NMR by: s (singlet), d (doublet), t (triplet), q (quadruplet), m (multiplet), br. (broad), app. (apparent). ^{13}C NMR spectra were recorded using the UDEFT or PENDANT pulse sequences from the Bruker standard pulse program library. ^{13}C DEPT spectra and 2D COSY, HSQC and HMBC spectra were recorded in order to assist with NMR assignment when necessary. Compound numbering is arbitrary and shown in the insert. NMR spectra were processed using MestReNova software. Mass spectra were obtained using Waters GCT

Premier (EI), Waters LCT (ES) or Waters Synapt (ES) spectrometers. High resolution spectra used a lock-mass to adjust the calibrated mass scale.

Gold catalyst synthesised as previously described in the literature.[1]

Chloro(dimethylsulfide) gold(I)

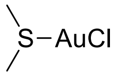

$KAuCl_4$ (145 mg, 0.384 mmol.) was dissolved in MeOH (1.6 mL) before the addition of dimethylsulfide (76 μL, 1.0 mmol.) as a solution in MeOH (0.4 mL) while minimising light exposure. The reaction mixture was stirred for 30 mins before collection of the white precipitate by vacuum filtration while continuing to minimise light exposure. The solid obtained was washed sequentially with a few drops each of MeOH, Et_2O and pentane before briefly air drying. The matte white crystals obtained were further dried under high vacuum, product sensitive to light (112 mg, 99%); ^{1}H NMR (300 MHz, $CDCl_3$) δ 2.75 (s, 6H); IR (neat): ν_{max} 2996, 1436, 1422, 1411, 1032, 993, 954 cm^{-1}.

Spectral data in accordance with that previously reported in the literature.[2]

Chloro[2-dicyclohexyl(2′,4′,6′-triisopropylbiphenyl)phosphine] gold(I)

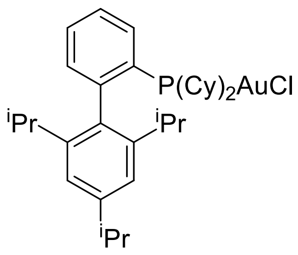

Dimethylsulfide Gold (I) Chloride (57.8 mg, 0.196 mmol.) and 2-dicyclohexylphosphino-2′,4′,6′-triisopropylbiphenyl (93.5 mg, 0.196 mmol.) were combined in a round bottomed flask containing dry CH_2Cl_2 (5 mL) held under an argon atmosphere. The resulting solution was stirred for 2 h while minimising light exposure. Following this the solvent is concentrated to approximately 20% of its initial volume before the addition of ^{n}hexane (5 mL), the solvent mixture was then further concentrated until a white precipitate formed, which was filtered off and washed with a few drops of ^{n}hexane and briefly air dried before further drying under high vacuum to yield white crystals (111 mg, 82%); mp: 243 - 245 °C (degrades); ^{1}H NMR (300 MHz, $CDCl_3$) δ 7.65 – 7.56 (m, 1H), 7.54 – 7.44 (m, 2H), 7.30 - 7.24 (m, 1H), 7.08 (s, 2H), 2.98 (hept., *J* = 6.9 Hz, 1H), 2.22 (hept., *J* = 6.8 Hz, 2H), 2.16 – 1.98 (m, 4H), 1.88 - 1.71 (m, 6H), 1.71 – 1.60 (m, 2H), 1.59 – 1.41 (m, 3H), 1.38 (s, 3H), 1.36 (s, 3H), 1.31 (s, 3H), 1.29 (s, 3H), 1.28 – 1.12 (m, 7H), 0.95 (s, 3H), 0.93 (s, 3H); ^{31}P NMR (121 MHz, $CDCl_3$) δ 35.28; IR (neat): ν_{max} 2920, 2849, 1607, 1460, 1445, 1426, 1381, 1361, 1292, 1269, 1174, 1123, 1054, 1001, 872, 853, 770, 738 cm^{-1}.

Spectral data in accordance with that previously reported in the literature.[3]

Bis(trifluoromethanesulfonyl)imido[2-di-*tert*-butyl(2′,4′,6′ triisopropylbiphenyl)phosphine] gold(I)

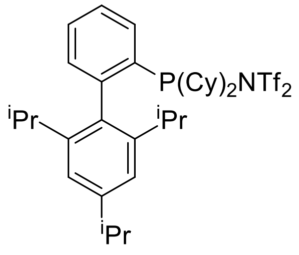

Chloro[2-di-*tert*-butyl(2′,4′,6′-triisopropylbiphenyl)phosphine] gold(I) (98.6 mg, 0.139 mmol.) was dissolved in dry CH_2Cl_2 (4.5 mL) and held under an argon atmosphere before the addition of silver bis(trifluoromethanesulfonyl)imide (55.2 mg, 0.142 mmol.). The immediate precipitation of a white solid was observed and the mixture was protected from light and stirred for 1.5 h before filtering through a pipette sized celite column eluted with CH_2Cl_2. The filtrate was evaporated under reduced pressure to yield a white crystalline solid that was further dried under high vacuum (121 mg, 91%). ^{31}P NMR (121 MHz, $CDCl_3$) δ 32.95 (s); IR (neat): ν_{max} 2932, 2856, 1449, 1397, 1355, 1188, 1129, 1057, 952, 823, 768 cm^{-1}.

Spectral data in accordance with that previously reported in the literature.[1a,d]

Starting Materials

The following compounds were prepared following literature procedures:

***N*-(4-Methoxyphenyl)-4-methylbenzenesulfonamide (A)**

(97%).[4] Spectroscopic data were identical to those reported in literature.[5]

Methyl 4-(methylsulfonamido)benzoate (B)

(98%).[6] Spectroscopic data were identical to those reported in literature.[7]

1*H*-Indole-3-carbaldehyde (C)

(75%).[8] Spectroscopic data were identical to those reported in literature.[8]

Methyl 4-(2,2-dibromovinyl)benzoate (D)

(69%).[9] Spectroscopic data were identical to those reported in literature.[9]

1-(2,2-Dibromovinyl)-4-fluorobenzene (E)

(82%).[10] Spectroscopic data were identical to those reported in literature.[10]

1-Bromo-3-(2,2-dibromovinyl)benzene (F)

(37%).[11] Spectroscopic data were identical to those reported in literature.[11]

3-Iodo-1*H*-indole (G)

(93%).[12] Spectroscopic data were identical to those reported in literature.[13]

1-Ethynyl-4-(trifluoromethyl)benzene (H)

(60%).[14] Spectroscopic data were identical to those reported in literature.[14]

Methyl 4-ethynylbenzoate (I)
(76%).[15] Spectroscopic data were identical to those reported in literature.[15]
Methyl 3-ethynylbenzoate (J)
(91%).[16]Spectroscopic data were identical to those reported in literature.[16]
1-Bromo-2-ethynylbenzene (K)
(85%).[17] Spectroscopic data were identical to those reported in literature.[18]
1-Ethynyl-4-methoxybenzene (L)
(93%).[19] Spectroscopic data were identical to those reported in literature.[20]
(*E*)-But-1-en-3-yn-1-ylbenzene (M)
(47%).[21] Spectroscopic data were identical to those reported in literature.[21]
Bromophenylacetylene (N)
(92%).[22] Spectroscopic data were identical to those reported in literature.[22]
1-(2,2-Dibromovinyl)-4-methoxybenzene (O)
(92%).[23] Spectroscopic data were identical to those reported in literature.[23]

2-(2,2-Dibromovinyl)furan (P)
(82%).[23] Spectroscopic data were identical to those reported in literature.[23]
1-(2,2-Dibromovinyl)-4-fluorobenzene (Q)
(82%).[10] Spectroscopic data were identical to those reported in literature.[10]

Preparative Methods for Starting Materials

General Procedure 1 (GP1) Preparation of *N*-arylsulfonamides

Methanesulfonyl chloride or *p*-toluenesulfonyl chloride (1.2 eq.) was added in small portions over 10 min to a solution of arylamine (1.0 eq.) in pyridine (0.5 M) at 0 °C. After the addition was completed the reaction was allowed to warm to room temperature and stirred overnight. Most of the solvent was evaporated under reduced pressure and the residue was diluted with CH_2Cl_2, washed with 2M $HCl_{(aq)}$ and the organic phase separated. The aqueous layer was extracted with CH_2Cl_2 three times and the combined organic extracts were dried over $MgSO_4$, filtered and the solvent removed under reduced pressure. The residue was purified by flash chromatography over silica gel.

N-Phenylmethanesulfonamide (R)

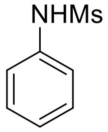

Following **GP1** using aniline (1.64 mL, 18.0 mmol). After purification by flash chromatography [^{n}hexane:AcOEt 3:1] sulfonamide **R** was isolated as a white solid

(2.96 g, 96%); ^{1}H-NMR (300 MHz, $CDCl_3$): δ = 7.42-7.32 (m, 2H), 7.25-7.16 (m, 3H), 6.54 (brs, 1H), 3.02 (s, 3H); ^{13}C-NMR (101 MHz, $CDCl_3$): δ = 136.9, 129.8, 125.6, 120.9, 39.4; IR (Neat): ν_{max} 3251, 1598, 1495, 1471, 1393, 1320, 1147, 1075, 975, 919, 894, 754, 692 cm^{-1}.

Spectroscopic data were identical to those reported in literature.[24]

***N*-(Benzo[d][1,3]dioxol-5-yl)-4-methylbenzenesulfonamide (S)**

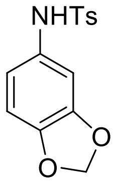

Following **GP1** using 3,4-(methylenedioxy)aniline (2.33 mL, 17.0 mmol). After purification by flash chromatography [^{n}hexane:AcOEt 7:3] sulfonamide **S** was isolated as a white solid (4.76 g, 96%); mp: 144-146 °C; ^{1}H-NMR (300 MHz, $CDCl_3$): δ = 7.60 (d, *J* = 8.3 Hz, 2H), 7.23 (d, *J* = 8.3 Hz, 2H), 6.67 (d, *J* = 2.2 Hz, 1H), 6.63 (d, *J* = 8.2 Hz, 1H), 6.40 (dd, *J* = 8.2, 2.2 Hz, 1H), 6.33 (brs, 1H), 5.94 (s, 2H), 2.40 (s. 3H); ^{13}C-NMR (101 MHz, $CDCl_3$): δ = 148.1, 146.0, 143.9, 136.0, 130.3, 129.7, 127.5, 116.9, 108.3, 105.5, 101.6, 21.7; IR (Neat): ν_{max} 3285, 2906, 1597, 1, 1416, 1344, 1314, 1248, 1194, 1156, 1129, 1089, 1040, 937, 899, 815, 784, 705, 672, 596 cm^{-1}; HR-MS (EI-TOF): *m/z*: calcd for $C_{14}H_{13}NO_4S$: 291.0565 found 291.0566 $[M]^+$.

4-Methyl-*N*-(4-(methylthio)phenyl)benzenesulfonamide (T)

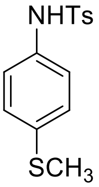

Following **GP1** using 4-(methylthio)aniline (1.87 mL, 15.0 mmol). After purification by flash chromatography [^{n}hexane:AcOEt 65:35] sulfonamide **T** was isolated as a white solid (4.18 g, 95%); mp: 100-102 °C; ^{1}H-NMR (300 MHz, $CDCl_3$): δ = 7.62 (d, *J* = 8.0 Hz, 2H), 7.22 (d, *J* = 8.0 Hz, 2H), 7.12 (d, *J* = 8.6 Hz, 2H), 6.98 (d, *J* = 8.6 Hz, 2H), 6.56 (brs, 1H), 2.43 (s, 3H), 2.38 (s, 3H); ^{13}C-NMR (101 MHz, $CDCl_3$): δ = 144.0, 136.0, 135.5, 133.9, 129.8, 127.8, 127.4, 122.7, 21.6, 16.3; IR (Neat): ν_{max} 3317, 2915, 1598, 1492, 1450, 1393, 1336, 1296, 1281, 1216, 1155, 1088, 1013, 915, 802, 704, 661, 573 cm^{-1}; HR-MS (EI-TOF): *m/z*: calcd for $C_{14}H_{15}NO_2S_2$: 293.0544 found 293.0545 $[M]^+$.

4-Methyl-*N*-(*o*-tolyl)benzenesulfonamide (U)

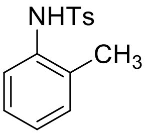

Following **GP1** using *o*-toluidine (1.81 mL, 17.0 mmol). After purification by flash chromatography [^{n}hexane:AcOEt 85:15] sulfonamide **U** was isolated as a white solid (4.27 g, 96%); mp: 94-96 °C; ^{1}H-NMR (300 MHz, $CDCl_3$): δ = 7.58 (d, *J* = 8.3 Hz, 2H), 7.29 (d, *J* = 7.7 Hz, 1H), 7.19 (d, *J* = 8.3 Hz, 2H), 7.16-7.02 (m, 3H), 6.27 (brs, 1H), 2.37 (s, 3H), 1.97 (s, 3H); ^{13}C-NMR (101 MHz, $CDCl_3$): δ = 143.9, 136.9, 134.7, 131.5, 130.9, 129.7, 127.3, 127.1, 126.3, 124.5, 21.7, 17.7; IR (Neat): ν_{max} 3277, 1599, 1488, 1396, 1329, 1158, 1091, 903, 816, 762, 675 cm^{-1}; HR-MS (EI-TOF): *m/z*: calcd for $C_{14}H_{15}NO_2S$: 261.0824 found 261.0828 $[M]^+$.

***N*-(4-Iodophenyl)methanesulfonamide (V)**

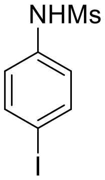

Following **GP1** using 4-iodoaniline (5.56 mL, 25.5 mmol). After purification by flash chromatography [^{n}hexane:AcOEt 7:3] sulfonamide **V** was isolated as a white solid (6.21 g, 83%); mp: 128-130 °C; ^{1}H-NMR (300 MHz, $CDCl_3$): δ = 7.66 (d, *J* = 8.8 Hz, 2H), 6.99 (d, *J* = 8.8 Hz, 2H), 6.75 (brs, 1H), 3.02 (s, 3H); ^{13}C-NMR (101 MHz, $CDCl_3$): δ = 138.8, 136.7, 122.5, 89.3, 39.6; IR (Neat): ν_{max} 3292, 3015, 2936, 1586, 1485, 1445, 1382, 1316, 1274, 1215, 1144, 985, 915, 809, 758, 648 cm^{-1}; HR-MS (EI-TOF): *m/z*: calcd for $C_7H_8NO_2SI$: 296.9321 found 296.9326 $[M]^+$.

General Procedure 2 (GP2): Mesylation of functionalised amines

Methanesulfonyl chloride (1.12 eq.) was added in small portions over 10 min to a solution of the relvent amine (1.0 eq.) and triethylamine (1.15 eq.) in dry CH_2Cl_2 (0.3 M) at 0 °C. After the addition was completed the reaction was warmed to room temperature and stirred overnight. The reaction was washed with saturated $NH_4Cl_{(aq.)}$, the organic phase separated and the aqueous layer extracted three times with CH_2Cl_2. The combined organic extracts were dried over $MgSO_4$, filtered and the solvent removed under reduced pressure. The crude was purified by flash chromatography over silica gel.

***N*-(4-Methoxybenzyl)methanesulfonamide (W)**

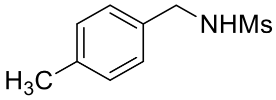

Following **GP2** using 4-methylbenzylamine (0.64 mL, 5.0 mmol). After purification by flash chromatography [^{n}hexane:EtOAc 1:1] sulfonamide **W** was obtained as tan crystals (822 mg, 89%); mp: 92-93 °C; ^{1}H NMR (300 MHz, $CDCl_3$) δ = 7.23 (d, *J* = 8.1 Hz, 2H), 7.17 (d, *J* = 8.1 Hz, 2H), 4.54 (s, 1H), 4.29 (d, *J* = 6.0 Hz, 2H), 2.87 (s, 3H), 2.35 (s, 3H); IR (neat): ν_{max} 3253, 2923, 1515, 1449, 1433, 1308, 1252, 1153, 1134, 1074, 965, 844, 802, 758, 710, 669 cm^{-1}.

Spectroscopic data is in accordance with the literature.[25]

***N*-(4-Methylbenzyl)methanesulfonamide (X)**

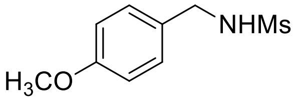

Following **GP2** using 4-methoxybenzylamine (0.65 mL, 5.0 mmol). After purification by flash chromatography [^{n}hexane:EtOAc 1:1] sulfonamide **X** was obtained as white crystals (803 mg, 75%); mp: 96-97 °C; ^{1}H NMR (300 MHz, $CDCl_3$) δ = 7.27 (d, *J* = 8.6 Hz, 2H), 6.89 (d, *J* = 8.6 Hz, 2H), 4.55 (brs, 1H), 4.26 (d, *J* = 5.3 Hz, 2H), 3.81 (s, 3H), 2.86 (s, 3H); IR (Neat): ν_{max} 3284, 1612, 1514, 1430, 1325, 1302, 1245, 1226, 1138, 1027, 999, 958, 862, 835, 763, 754, 719, 680 cm^{-1}.

Spectroscopic data is in accordance with the literature.[26]

***N*-(3-Methoxybenzyl)methanesulfonamide (Y)**

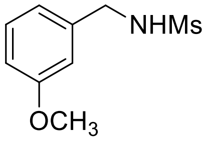

Following **GP2** using 3-methoxybenzylamine (0.26 mL, 2.0 mmol). After purification by flash chromatography [^{n}hexane:EtOAc 3:2] sulfonamide **Y** was obtained as white crystals (61%); mp: 61-62 °C; ^{1}H NMR (300 MHz, $CDCl_3$) δ = 7.30 (d, *J* = 7.8 Hz, 1H), 6.95–6.83 (m, 3H), 4.62 (br. s, 1H), 4.30 (d, *J* = 5.8 Hz, 2H), 3.81 (s, 3H), 2.89 (s, 3H); ^{13}C NMR (101 MHz, $CDCl_3$) δ 160.0, 138.2, 130.0, 120.0, 113.6, 113.4, 55.3, 47.2, 41.1; IR (neat): ν_{max} 3251, 3025, 2950, 2844, 1600, 1492, 1471, 1447, 1432, 1416, 1360, 1311, 1287, 1253, 1165, 1151, 1132, 1093, 1034, 977, 912, 863, 841, 788, 738, 695 cm^{-1}; HR-MS (ES-TOF): *m/z* calcd for $C_9H_{13}NO_3SNa$: 238.0514 found 238.0520 $[M+Na]^+$.

***N*-Allylmethanesulfonamide (Z)**

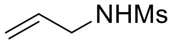

Following **GP2** using allylamine (1.88 mL, 25.0 mmol). After purification by flash chromatography [^{n}hexane:EtOAc 65:35] sulfonamide **Z** was obtained as a colourless oil (3.32 g, 98%); ^{1}H-NMR (300 MHz, $CDCl_3$): δ = 5.87 (ddt, *J* = 17.0, 10.2, 5.8 Hz, 1H), 5.31 (app. dq, *J* = 17.0, 1.5 Hz, 1H), 5.23 (app. dq, *J* = 10.2, 1.5 Hz, 1H), 3.78 (app. tt, *J* = 5.8, 1.5 Hz, 2H), 2.97 (s, 3H); ^{13}C-NMR (101 MHz, $CDCl_3$): δ = 133.6, 117.8, 45.7, 41.0; IR (neat): ν_{max} 3286, 1434, 1412, 1308, 1141, 1062, 697, 926, 837, 754 cm^{-1}.

General Procedure 3 (GP3): Titanium tetrachloride imide formation

Using a previously described protocol from the literature.[27]

The aldehyde (10 mmol, 1.0 eq.), H_2NTs (1.7 g, 10 mmol, 1.0 eq.) and Et_3N (4.2 mL, 30 mmol, 3.0 eq.) were dissolved in dry CH_2Cl_2 (50 mL) under an argon atmosphere before being cooled to 0 °C. A solution of $TiCl_4$ (0.6 mL, 5.5 mmol, 0.55 eq.) in dry CH_2Cl_2 (10 mL) was then added over *ca*. 5 mins, the reaction mixture was then held at 0 °C for 2 h before warming to r.t. and vacuum filtering through celite. Unless otherwise stated, at this point the filtrate was freed of all volatiles by rotary evaporation under reduced pressure and the solid residue acquired was then suspended in the specified dry solvent under argon (either PhMe or Et_2O) (75 mL) and the solid broken up and vigorously stirred for 20 mins. This suspension was then allowed to settle and decanted, with as much of the supernatant as possible poured onto a vacuum filter through celite. This process was then repeated using the remainder of the solid, and the combined filtrates then subjected to rotary evaporation under reduced pressure to obtain the imides, which owing to their reasonable purity were used directly in the $NaBH_4$ reduction.

General Procedure 4 (GP4): Reduction of the *N*-tosyl imide with $NaBH_4$

The imide (15 mmol, 1.0 eq.) was suspended in MeOH (30 mL, 0.5 M) before cooling to 0 °C. $NaBH_4$ (15 mmol, 1.0 eq.) was then added slowly in portions over *ca*. 5 minutes, the reaction mixture was then allowed to warm to r.t. and stirred for 18 h before quenching with H_2O (30 mL) and stirring for 15 mins. The reaction mixture was diluted with EtOAc (50 mL) and placed in a separating funnel, before extracting with further EtOAc (2 × 50 mL), the pooled extracts were then dried over Na_2SO_4, filtered and the solvent removed by rotary evaporation under reduced pressure. The crude sulfonamide was typically of reasonable purity and could be used directly in the next step, otherwise it was purified as described.

***N*-(4-(Allyloxy)benzyl)-4-methylbenzenesulfonamide (AA)**

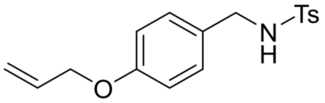

4-Hydroxybenzaldehyde (2.4 g, 20 mmol) was dissolved in acetone (24 mL) with K_2CO_3 (8.4 g, 61 mmol) and allyl bromide (2.6 mL, 30 mmol) and stirred at r.t. for 2 h and then heated to reflux for 2 h. H_2O and EtOAc were then added and the mixture placed in a separating funnel and extracted with EtOAc. The extracts were dried over Na_2SO_4, filtered, and the solvents removed under reduced pressure, purified by flash chromatography [^{n}hexane:EtOAc 95:5–9:1] to give 4-allyloxybenzaldehyde (pale yellow oil, 2.0 g, 62%). This was directly used in **GP3** (Et_2O used in work up rather than EtOAc) to give the crude imide (1.8 g, 50%) which was then directly used in **GP4** to give **AA** as a pale yellow solid (1.6 g, 92%); mp: 92 °C; ^{1}H NMR (300 MHz, $CDCl_3$) δ 7.75 (d, *J* = 8.2 Hz, 2H), 7.31 (d, *J* = 8.2 Hz, 2H), 7.10 (d, *J* = 8.7 Hz, 2H), 6.81 (d, *J* = 8.7 Hz, 2H), 6.12–5.95 (m, 1H), 5.39 (d app. q, *J* = 17.3, 1.5 Hz, 1H), 5.28 (d app. q, *J* = 10.5, 1.5 Hz, 1H), 4.62–4.56 (m, 1H), 4.50 (d app. t, *J* = 5.3, 1.5 Hz, 2H), 4.05 (d, *J* = 6.1 Hz, 2H), 2.44 (s, 3H); ^{13}C NMR (101 MHz, $CDCl_3$) δ 158.4, 143.6, 137.1, 133.2, 129.8, 129.4, 128.6, 127.3, 117.9, 115.0, 68.9, 46.9, 21.7; IR (neat): ν_{max} 3300, 2918, 2865, 1613, 1598, 1586, 1511, 1453, 1419, 1325, 1303, 1243, 1155, 1092, 1047, 1013, 997, 939, 868, 815, 786, 707, 668 cm^{-1}; HR-MS (ESI-TOF): *m/z* calcd for $C_{17}H_{19}NO_3SNa$: 340.0983 found 340.0986 $[M+Na]^+$.

***N*-(2-Methoxybenzyl)-4-methylbenzenesulfonamide (AB)**

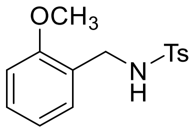

Following **GP3** using 2-methoxybenzaldehyde (1.6 g, 12 mmol) and at completion the reaction mixture was vacuum filtered through celite and the filtrate placed in a separating funnel and rapidly washed with H_2O (*ca*. 40 mL) and brine (20 mL), the organic fraction was then dried over Na_2SO_4, filtered and stripped of solvent under reduced pressure giving the crude imide (2.4 g, 72%) which was

used directly in **GP4** to give **AB** as a pale yellow solid (2.3 g, 96%); mp: 61–62 °C; ^{1}H NMR (300 MHz, $CDCl_3$) δ 7.66 (d, *J* = 8.3 Hz, 2H), 7.23–7.16 (m, 3H), 7.06 (dd, *J* = 7.4, 1.6 Hz, 1H), 6.80 (app. td, *J* = 7.4, 0.9 Hz, 1H), 6.73 (d, *J* = 8.2 Hz, 1H), 5.09 (t, *J* = 6.3 Hz, 1H), 4.13 (d, *J* = 6.3 Hz, 2H), 3.74 (s, 3H), 2.38 (s, 3H); IR (neat): ν_{max} 3287, 3267, 2938, 1600, 1489, 1462, 1436, 1420, 1321, 1307, 1293, 1281, 1239, 1151, 1118, 1094, 1071, 1045, 1028, 861, 842, 814, 758, 718, 705, 659 cm^{-1}.

Spectroscopic data in accordance with the literature.[28]

4-Methyl-*N*-(2-methylbenzyl)benzenesulfonamide (AC)

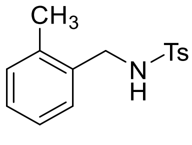

Following **GP3** using 2-methylbenzaldehyde (2.7 g, 23 mmol) and at completion the reaction mixture was quenched with saturated solution of $NaHCO_3$ and the aqueous layer was extracted with DCM (3 × 20 mL). The combined organic layers were washed with brine, separated and dried over Na_2SO_4 before being filtered and freed of solvent by rotary evaporation under reduced pressure. The crude product was recrystallized from EtOH to give the imide as white needles (3.6 g, 58%) which were then directly used in **GP4** to provide the crude sulfonamide, which was purified by recrystallisation from EtOH to give **AC** as white needles (2.6 g, 72%); mp: 121-122 °C; ^{1}H NMR (300 MHz, $CDCl_3$) δ 7.77 (d, *J* = 8.2 Hz, 2H), 7.32 (d, *J* = 8.2 Hz, 2H), 7.22–7.09 (m, 4H), 4.48 (s, 1H), 4.09 (d, *J* = 6.0 Hz, 2H), 2.45 (s, 3H), 2.25 (s, 3H); IR (neat): ν_{max} 3262, 3024, 2925, 1597, 1490, 1460, 1424, 1319, 1303, 1152, 1116, 1092, 1034, 949, 879, 840, 807, 748, 732, 703, 655 cm^{-1}.

Spectroscopic data in accordance with the literature.[29]

4-Methyl-*N*-(2,4,6-trimethylbenzyl)benzenesulfonamide (AD)

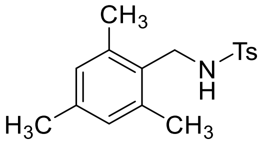

Following **GP3** using 2,4,6-trimethylbenzaldehyde (3.0 g, 20 mmol) (PhMe used in work up) to give the crude imide (4.7 g, 77%) which was then directly used in **GP4** to provide the crude sulfonamide, which was purified by recrystallisation from EtOH to give **AD** as white needles (2.0 g, 41%); mp: 141-143 °C; ^{1}H NMR (400 MHz, $CDCl_3$) δ 7.79 (d, *J* = 8.2 Hz, 2H), 7.33 (d, *J* = 8.2 Hz, 2H), 6.79 (s, 2H), 4.18 (t, *J* = 5.3 Hz, 1H), 4.06 (d, *J* = 5.3 Hz, 2H), 2.45 (s, 3H), 2.23 (s, 3H), 2.14 (s, 6H); ^{13}C NMR (101 MHz, $CDCl_3$) δ 143.5, 138.0, 137.3, 136.3, 129.7, 129.2, 128.9, 127.3, 41.2, 21.5, 20.9, 19.1; IR (neat): ν_{max} 3287, 2916, 1598, 1448, 1403, 1343, 1318, 1306, 1159, 1091, 1038, 1023, 915, 856, 819, 780, 708, 669 cm^{-1}; HR-MS (ES+): *m/z*: calcd for $C_{17}H_{21}NO_2SNa$: 326.1191 found 326.1193 $[M+Na]^+$.

4-Methyl-*N*-(naphthalen-1-ylmethyl)benzenesulfonamide (AE)

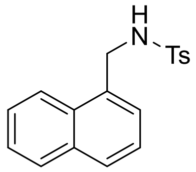

Following **GP3** from 1-naphthaldehyde (1.6 mL, 11 mmol) gave the crude imide (2.5 g, 70%), most of which (2.3 g, 7.4 mmol) was used directly in **GP4** to give the above sulfonamide **AE** (2.1 g, 94%); mp: 145 °C; ^{1}H NMR (300 MHz, $CDCl_3$) δ 7.83–7.62 (m, 5H), 7.41–7.38 (m, 2H), 7.25–7.17 (m, 4H), 4.62 (t, *J* = 5.7 Hz, 1H); 4.45 (d, *J* = 5.7 Hz, 2H), 2.36 (s, 3H); IR (neat): ν_{max} 3327, 3295, 3063, 2922, 1597, 1510, 1492, 1417, 1393, 1339, 1317, 1303, 1157, 1093, 1035, 968, 919, 902, 858, 802, 779, 714, 667 cm^{-1}.

Spectroscopic data in accordance with the literature.[29]

3-(2,2-Dibromovinyl)-1-(methylsulfonyl)-1*H*-indole (AG)

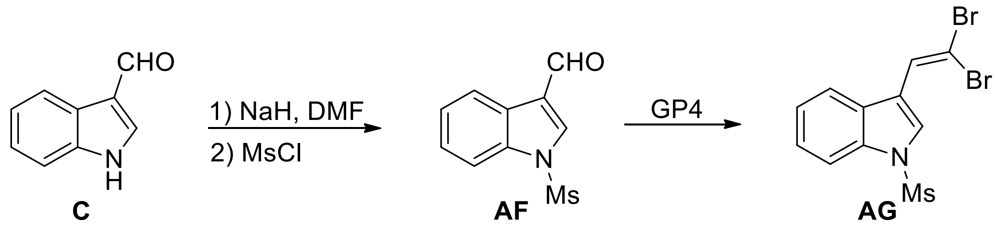

Sodium hydride (250 mg, 55% in mineral oil, 6.0 mmol) was added at 0 °C under an argon atmosphere to a solution of 1*H*-indole-3-carbaldehyde (**C**) (725 mg, 5.0 mmol) in dry DMF (10 mL). The mixture was stirred at 0 °C for 30 min before the dropwise addition of methanesufonyl chloride (465 µL, 6.0 mmol). After stirring at 0 °C for 2h the reaction was diluted with EtOAc (20 mL), saturated $NH_4Cl_{(aq.)}$ (20 mL) was added and the layers partitioned. The organic phase was washed with saturated $NaHCO_{3(aq.)}$, brine and dried over $MgSO_4$. After filtration, the solution was concentrated under reduced pressure and the residue was purified by flash chromatography [^{n}hexane:EtOAc 3:1] to afford aldehyde **AF** as a white solid (698 mg, 63%); mp: 166-168 °C; ^{1}H-NMR (300 MHz, $CDCl_3$): δ = 10.12 (s, 1H), 8.35 (dd, *J* = 7.3, 1.4 Hz, 1H), 8.12 (s, 1H), 7.89 (dd, *J* = 7.3, 1.7 Hz, 1H), 7.50 (td, *J* = 7.3, 1.7 Hz, 1H), 7.45 (td, *J* = 7.3, 1.4 Hz, 1H), 3.29 (s, 3H); ^{13}C-NMR (101 MHz, $CDCl_3$): δ = 185.4, 136.1, 135.4, 126.8, 126.4, 125.5, 123.1, 122.5, 112.9, 41.9; IR (Neat): ν_{max} 3141, 3023, 1675, 1541, 1481, 1443, 1366, 1329, 1234, 1174, 1127, 975, 787, 746, 698, 585 cm^{-1}; HR-MS (EI-TOF): *m/z*: calcd for $C_{10}H_9NO_3S$: 223.0303 found 223.0299 $[M]^+$.

Following a literature method,[30] aldehyde **AF** (447 mg, 2.0 mmol) was added at 0 °C to a mixture of triphenylphosphine (4 eq.) and carbon tetrabromide (2 eq.) in dry CH_2Cl_2 (0.1 M). The reaction was stirred until complete consumption of the starting aldehyde was observed by TLC. The reaction was directly dry-loaded onto silica gel and purified by flash

chromatography [^{n}hexane:EtOAc 4:1]. Dibromoalkene **AG** was isolated as a white solid (504 mg, 67%); mp: 168-170 °C; ^{1}H-NMR (300 MHz, $CDCl_3$): δ = 8.18 (s, 1H), 7.92 (dd, *J* = 7.7, 1.3 Hz, 1H), 7.64 (dd, *J* = 7.7, 1.4 Hz, 1H), 7.61 (s, 1H), 7.43 (td, *J* = 7.7, 1.4 Hz, 1H), 7.37 (td, *J* = 7.7, 1.3 Hz, 1H), 3.16 (s, 3H); ^{13}C-NMR (101 MHz, $CDCl_3$): δ = 134.3, 129.5, 126.8, 125.9, 124.8, 124.0, 119.3, 117.4, 113.3, 90.7, 41.2; IR (Neat): ν_{max} 3182, 3034, 2933, 1543, 1447, 1357, 1269, 1164, 1142, 1020, 977, 871, 852, 785, 756, 736, 592, 568 cm^{-1}; (ES-TOF): *m/z*: calcd for $C_{11}H_{10}NO_2S^{79}Br^{81}Br$: 319.8779 found 379.8776 $[M+H]^+$.

1-(3-Ethynyl-1*H*-indol-1-yl)ethanone (AI)

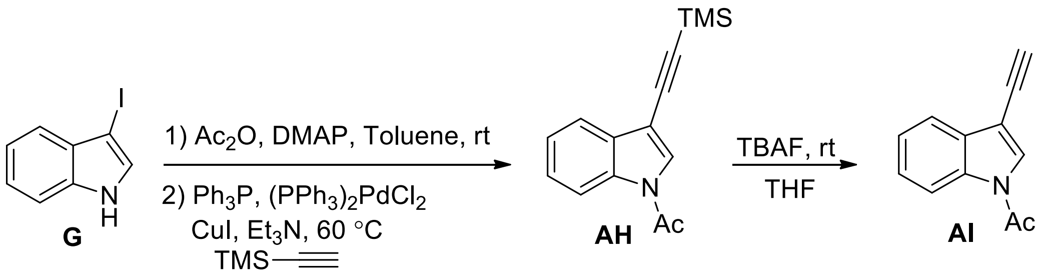

Acetic anhydride (695 µL, 7.41 mmol) and *N*,*N*-dimethylaminopyridine (181 mg, 1.48 mmol) were added to a solution of 3-iodo-1*H*-indole (**G**) (400 mg, 1.65 mmol) in dry toluene (13.5 mL) at room temperature under an argon atmosphere. After 30 min, triethylamine (13.5 mL), PPh_3 (43.1 mg, 0.16 mmol), CuI (31.3 mg, 0.16 mmol), $(PPh_3)_2PdCl_2$ (57.7 mg, 0.08 mmol), and trimethylsilylacetylene (1.14 mL, 8.23 mmol) were added to the reaction mixture and the temperature was raised to 60 °C. After 30 min the reaction was cooled to room temperature, quenched with 10% NH_4Cl solution, transfered to a separating funnel and extracted with toluene (3 × 10 mL). The combined organic extracts were dried over $MgSO_4$, filtered and the solvent removed under reduced pressure. The crude residue obtained was purified by flash chromatography [^{n}hexane:EtOAc 5%] to afford **AH** (282 mg, 67%) as a white solid; mp: 88-90 °C; ^{1}H-NMR (300 MHz, $CDCl_3$): δ = 8.44-8.38 (m, 1H), 7.69-7.65 (m, 1H), 7.63 (s, 1H), 7.40 (ddd, *J* = 7.3, 7.3, 1.5 Hz, 1H), 7.35 (ddd, *J* = 7.3, 7.3, 1.3 Hz, 1H), 2.63 (s, 3H), 0.29 (s, 9H); ^{13}C-NMR (101 MHz, $CDCl_3$): δ = 168.3, 135.1, 130.6, 128.7, 126.2, 124.3, 120.2, 116.7, 105.5, 99.3, 96.3, 24.0, 0.2; IR (Neat): ν_{max} 3154, 2961, 2159, 1713, 1552, 1449, 1374, 1351, 1335, 1243, 1216, 1085, 1012, 936, 840, 756, 701, 669 cm^{-1}; (EI-TOF): *m/z*: calcd for $C_{15}H_{17}NOSi$: 255.1079 found 255.1078 $[M]^+$.

A TBAF solution (1.76 mL, 1 M in THF, 1.76 mmol) was added dropwise at 0 °C to a solution of **AH** (430 mg, 1.68mmol) in THF (17 mL). After stirring at 0 °C for 10 min, the reaction mixture was quenched with water (17 mL) and transferred to a seperating funnel, the

organic phase separated and the aqueous layer extracted with EtOAc (3 × 5 mL). The combined organic extracts were dried over $MgSO_4$, filtered and the solvents removed under reduced pressure. The crude residue obtained was purified by flash chromatography [^{n}hexane:EtOAc 5%] to afford **AI** (190 mg, 62%) as a white solid; mp: 76-78 °C; ^{1}H-NMR (300 MHz, $CDCl_3$): δ = 8.45-8.39 (m, 1H), 7.72-7.67 (m, 1H), 7.66 (s, 1H), 7.41 (ddd, *J* = 7.3, 7.3, 1.4 Hz, 1H), 7.35 (ddd, *J* = 7.3, 7.3, 1.2 Hz, 1H), 3.29 (s, 1H), 2.64 (s, 3H); ^{13}C-NMR (101 MHz, $CDCl_3$): δ = 168.2, 135.0, 130.4, 129.1, 126.2, 124.4, 120.0, 116.7, 104.2, 81.5, 75.5, 24.0; IR (Neat): ν_{max} 3305, 3132, 3088, 1682, 1556, 1450, 1386, 1344, 1222, 1175, 1118, 1009, 933, 828, 759, 741 cm^{-1}; HR-MS (EI-TOF): *m/z*: calcd for $C_{12}H_9NO$: 183.0684 found 183.0687 $[M]^+$.

***N*-benzylmethanesulfonamide (AK)**

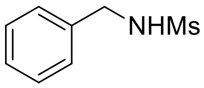

Produced using **GP2** using benzylamine (1.9 mL, 17 mmol.), purified by flash chromatography (^{n}hexane:EtOAc [9:1 – 7:3]); **AK** was obtained as white crystals (2.6 g, 82%); mp: 64 - 65 °C; ^{1}H NMR (300 MHz, $CDCl_3$) δ 7.41 – 7.28 (m, 5H), 4.84 (s, 1H), 4.31 (d, *J* = 6.2 Hz, 2H), 2.85 (s, 3H); IR (neat): ν_{max} 3225, 1455, 1436, 1412, 1294, 1132, 1082, 1060, 1015, 975, 875, 765, 737, 695 cm^{-1}.

Spectroscopic data in accordance with the literature.[31]

Preparation of Ynamides

General Procedure 5 (GP5)

Following the method of Evano,[32] a 10-25 mL flask was charged with sulfonamide (1.0 eq.), 1,1-dibromo-1-alkene (1.5 eq.), Cs_2CO_3 (4.0 eq.) and copper (I) iodide (0.12 eq.). The flask was fitted with a rubber septum, evacuated under high vacuum and backfilled with argon (this operation was repeated three times). Dry DMF or 1,4-dioxane (0.5 M with respect to the sulfonamide) and *N*,*N*'-dimethylethylenediamine (0.19 eq.) were then added and the mixture was stirred at 60 °C until complete consumption of the starting sulfonamide was observed by TLC (typically 15 – 24 h). For reactions in DMF the reaction was diluted with water, extracted with diethyl ether and the combined organic layers were washed with brine, dried over $MgSO_4$, filtered and concentrated under reduced pressure. Where the reaction is specified as being completed in 1,4-dioxane, instead the reaction mixture was diluted with EtOAc and vacuum filtered through a short pad of silica, eluting with additional EtOAc,

before concentration of the filtrate under reduced pressure. In both cases the crude residue was purified by flash chromatography.

General Procedure 6 (GP6)

Following the method of Stahl,[33] $CuCl_2$ (0.2 eq.), sulfonamide (5.0 eq.) and Na_2CO_3 (2.0 eq.) were added to a 500 mL three-necked round-bottomed flask. The flask was purged with oxygen for 15 min and a solution of pyridine (2.0 eq.) in dry toluene (0.2 M) was added. A balloon filled with oxygen was connected to the flask and the stirred mixture was heated at 70 °C. After 15 min, a solution of alkyne (1.0 eq.) in dry toluene (0.2 M) was added by syringe pump over 4 h. The mixture was allowed to stir at 70 °C for another 4 h and cooled to rt. The reaction mixture was filtered over a plug of silica gel, washed with EtOAc and concentrated under reduced pressure. The crude residue was purified by flash chromatography.

General Procedure 7 (GP7)

Following the method of Stahl[33] slightly modified, the sulfonamide (6.0 eq.), $CuCl_2$ (0.2 eq.) and Na_2CO_3 (2.0 eq.) were added to a 500 mL three-necked round-bottomed flask. The flask was purged with oxygen for 15 min before addition of pyridine (2.0 eq.) as a solution in dry toluene (0.1 M). A balloon filled with oxygen was connected to the flask and the mixture was heated to 70 °C with stirring. After 15 min, a solution of alkyne (1.0 eq.) in dry toluene (0.1 M) was added by syringe pump over 6 h. After addition of the alkyne was complete the reaction mixture was stirred at 70 °C for a further 6 h. The reaction mixture was cooled to rt and then filtered through a short plug of silica gel eluting with EtOAc, the filtrate was then concentrated under reduced pressure. The crude residue obtained was then purified by flash chromatography.

General Procedure 8 (GP8)

Following the method of Hsung,[34] to a solution of 1-bromoalkyne (1.1 eq.) in dry toluene (1.0 M) in a flame dried flask under argon were added the sulfonamide (1.0 eq.), K_2CO_3 (4.0 eq.), $CuSO_4{\cdot}5H_2O$ (0.2 eq.) and 1,10-phenanthroline (0.4 eq.). The reaction mixture was then stirred at 60-65 °C and monitored by TLC until complete (typically 15 – 24 h) or until the reaction progressed no further. The mixture was cooled to room temperature, diluted with dichloromethane and filtered through a short plug of silica gel eluting with further dichloromethane. The filtrate was concentrated under reduced pressure and the crude residue purified by flash chromatography.

N-Phenyl-N-(phenylethynyl)methanesulfonamide (1a)

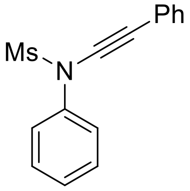

Following **GP8** using sulfonamide **R** (530 mg, 3.10 mmol) and phenylacetylene (68 µL, 0.62 mmol). After purification by flash chromatography [^{n}hexane:EtOAc 4:1] ynamide **1a** was isolated as a white solid (112 mg, 67%); mp: 76-78 °C; ^{1}H-NMR (300 MHz, $CDCl_3$): δ = 7.63-7.55 (m, 2H), 7.42-7.34 (m, 2H), 7.52-7.28 (m, 8H), 3.17 (s, 3H); ^{13}C-NMR (101 MHz, $CDCl_3$): δ = 138.8, 131.7, 129.6, 128.5, 128.3, 125.7, 122.4, 82.1, 71.1, 37.0; IR (Neat): ν_{max} 3078, 3009, 2928, 2243, 1588, 1482, 1365, 1324, 1164, 1156, 962, 893, 744, 689 cm^{-1}.

Spectroscopic data were identical to those reported in literature.[35]

N-(4-Methoxyphenyl)-4-methyl-N-(phenylethynyl)benzenesulfonamide (1b)

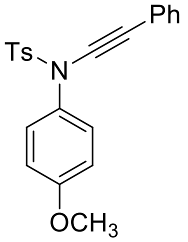

Following **GP6** using sulfonamide **A** (3.30 g, 11.9 mmol) and phenylacetylene (261 µL, 2.38 mmol). After purification by flash chromatography [^{n}hexane:EtOAc 9:1] ynamide **1b** was isolated as a brown solid (354 mg, 39%); mp: 128-130 °C; ^{1}H-NMR (300 MHz, $CDCl_3$): δ = 7.63 (d, *J* = 8.3 Hz, 2H), 7.42-7.34 (m, 2H), 7.34-7.26 (m, 5H), 7.18 (d, *J* = 9.0 Hz, 2H), 6.84 (d, *J* = 9.0 Hz, 2H), 3.81 (s, 3H), 2.45 (s, 3H); ^{13}C-NMR (101 MHz, $CDCl_3$): δ = 159.7, 145.0, 133.1, 131.8, 131.5, 129.6, 128.5, 128.4, 128.1, 128.0, 122.9, 114.4, 83.6, 70.1, 55.6, 21.8; IR (Neat): ν_{max} 2967, 2846, 2239, 1598, 1501, 1462, 1441, 1367, 1300, 1248, 1213, 1170, 1076, 1021, 913, 838, 758, 667 cm^{-1}; HR-MS (ES-TOF): *m/z*: calcd for $C_{22}H_{19}NO_3NaS$: 400.1000 found 400.0983 $[M+Na]^+$.

N-(Benzo[*d*][1,3]dioxol-5-yl)-4-methyl-N-(phenylethynyl)benzenesulfonamide (1c)

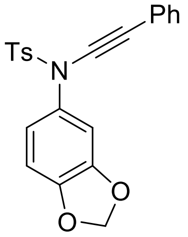

Following **GP8** using sulfonamide **S** (1.05 g, 3.60 mmol) and bromophenylacetylene (720 mg, 3.96 mmol) for 60 h. After purification by flash chromatography [^{n}hexane:EtOAc 9:1] ynamide **1c** was isolated as a brown solid (255 mg, 16%); mp: 68-70 °C; ^{1}H-NMR (300 MHz, $CDCl_3$): δ = 7.66 (d, *J* = 8.3 Hz, 2H), 7.42-7.26 (m, 7H), 6.78 (t, *J* = 1.3 Hz, 1H), 6.75-6.70 (m, 2H), 6.01 (s, 2H), 2.46 (s, 3H); ^{13}C-NMR (101 MHz, $CDCl_3$): δ = 148.0, 147.9, 145.2, 133.0, 132.8, 131.6, 129.7, 128.5, 128.4, 128.1, 122.8, 120.7, 108.2, 108.1, 102.0, 83.4, 70.4, 21.9; IR (Neat): ν_{max} 3060, 2919, 2239, 1597, 1503, 1482, 1442, 1376, 1336, 1247, 1170, 1135, 1061, 1034, 931, 868, 758, 723, 659 cm^{-1}; HR-MS (ES-TOF): *m/z*: calcd for $C_{22}H_{17}NO_4SNa$: 414.0776 found 414.0771 $[M+Na]^+$.

4-Methyl-*N*-(4-(methylthio)phenyl)-*N*-(phenylethynyl)benzenesulfonamide (1d)

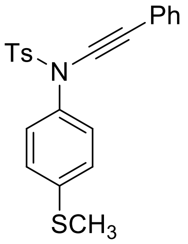

Following **GP7** using sulfonamide **T** (4.00 g, 13.6 mmol) and phenylacetylene (250 µL, 2.28 mmol). After purification by flash chromatography [^{n}hexane:EtOAc 9:1] ynamide **1d** was isolated as a white solid (591 mg, 66%); mp: 102-104 °C; ^{1}H-NMR (300 MHz, $CDCl_3$): δ = 7.63 (d, *J* = 8.3 Hz, 2H), 7.42-7.34 (m, 2H), 7.34-7.27 (m, 5H), 7.24-7.15 (m, 4H), 2.49 (s, 3H), 2.45 (s, 3H); ^{13}C-NMR (101 MHz, $CDCl_3$): δ = 145.2, 139.5, 136.0, 133.0, 131.6, 129.7, 128.5, 128.4, 128.1, 126.9, 126.7, 122.7, 83.1, 70.6, 21.9, 15.7; IR (Neat): ν_{max} 3022, 2934, 2243, 1594, 1484, 1466, 1444, 1412, 1363, 1330, 1269, 1242, 1168, 1138, 1084, 1059, 978, 965, 860, 825, 760 cm^{-1}; HR-MS (ES-TOF): *m/z*: calcd for $C_{22}H_{19}NO_2NaS_2$: 416.0755 found 416.0746 $[M+Na]^+$.

Methyl 4-(*N*-(phenylethynyl)methylsulfonamido)benzoate (1e)

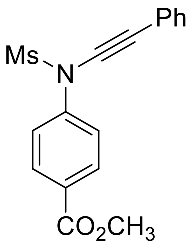

Following **GP7** using sulfonamide **B** (3.66 g, 16.0 mmol) and phenylacetylene (293 µL, 2.66 mmol). After purification by flash chromatography [^{n}hexane:EtOAc 4:1] ynamide **1e** was isolated as a white solid (277 mg, 32%); mp: 58-60 °C; ^{1}H-NMR (300 MHz, $CDCl_3$): δ = 8.11 (d, *J* = 8.8 Hz, 2H), 7.68 (d, *J* = 8.8 Hz, 2H), 7.53-7.42 (m, 2H), 7.39-7.29 (m, 3H), 3.94 (s, 3H), 3.20 (s, 3H); ^{13}C-NMR (101 MHz, $CDCl_3$): δ = 166.2, 142.7, 131.8, 131.0, 129.5, 128.7, 128.6, 124.4, 122.0, 81.0, 72.6, 52.5, 37.6; IR (Neat): ν_{max} 3046, 3008, 2937, 2237, 1709, 1604, 1503, 1436, 1362, 1326, 1285, 1210, 1161, 1114, 1069, 1017, 973, 919, 852, 753 cm^{-1}; HR-MS (EI-TOF): *m/z*: calcd for $C_{17}H_{15}NO_4S$: 329.0722 found 329.0723 $[M]^+$.

4-Methyl-*N*-(phenylethynyl)-*N*-(*o*-tolyl)benzenesulfonamide (1f)

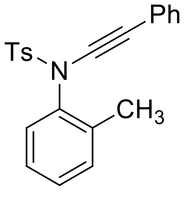

Following **GP8** using sulfonamide **U** (1.25 g, 4.80 mmol) and bromophenylacetylene (**N**) (960 mg, 5.30 mmol) for 36 h. After purification by flash chromatography [^{n}hexane:EtOAc 19:1] ynamide **1f** was isolated as a white solid (175 mg, 10%); mp: 96-98 °C; ^{1}H-NMR (300 MHz, $CDCl_3$): δ = 7.76 (d, *J* = 8.3 Hz, 2H), 7.44-7.31 (m, 4H), 7.31-7.22 (m, 5H), 7.19-7.08 (m, 1H), 6.96 (d, *J* = 7.8 Hz, 1H), 2.48 (s, 3H), 2.34 (s, 3H); ^{13}C-NMR (101 MHz, $CDCl_3$): δ = 145.1, 138.0, 137.7, 134.4, 131.7, 131.5, 129.8, 129.4, 128.6, 128.4, 128.2, 127.9, 126.8, 123.0, 83.5, 69.6, 21.9, 18.2; IR (Neat): ν_{max} 3063, 3037, 2932, 2864, 2238, 1598, 1489, 1444, 1364, 1302, 1220, 1171, 1118, 1091, 1072, 1028, 916, 810, 760, 713, 675, 653 cm^{-1}; HR-MS (ES-TOF): *m/z*: calcd for $C_{22}H_{19}NO_2SNa$: 384.1034 found 384.1045 $[M+Na]^+$.

***N*-(Benzo[*d*][1,3]dioxol-5-yl)-4-methyl-*N*-((4-(trifluoromethyl)phenyl)ethynyl)benzene-sulfonamide (1g)**

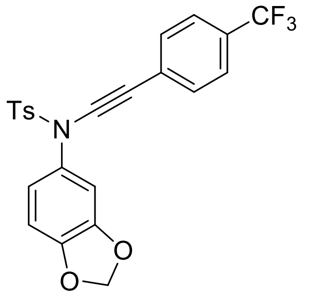

1-(Bromoethynyl)-4-(trifluoromethyl)benzene was prepared as follows. *n*-Butyllithium (0.75 mL, 2.5 M in hexane, 1.25 eq.) was added dropwise to a solution of 1-ethynyl-4-(trifluoromethyl)benzene (**H**) (254 mg, 1.49 mmol) in dry THF (3 mL) at –78 °C. After 10 min bromine (0.11 mL, 2.1 mmol, 1.4 eq.) was added dropwise over 10 min at the same temperature. After the addition was completed, the reaction was allowed to reach room temperature and then quenched by the slow addition of a $Na_2S_2O_3$ solution. The organic phase was separated and the aqueous layer extracted with Et_2O. The combined organic extracts were dried over $MgSO_4$, filtered and the solvents removed under reduced pressure. The product was used directly in the next step without further purification [^{1}H-NMR (300 MHz, $CDCl_3$): δ = 7.63-7.50 (m, 4H). Spectroscopic data were identical to those reported in literature[36]]. Following **GP8** using sulfonamide **S** (395 mg, 1.36 mmol) and bromoalkyne **AJ** (370 mg, 1.49 mmol) for 90 h. After purification by flash chromatography [^{n}hexane:EtOAc 9:1] ynamide **1g** was isolated as a colourless oil (55 mg, 9%); ^{1}H-NMR (300 MHz, $CDCl_3$): δ = 7.65 (d, *J* = 8.3 Hz, 2H), 7.54 (d, *J* = 8.1 Hz, 2H), 7.45 (d, *J* = 8.1 Hz, 2H), 7.32 (d, *J* = 8.3 Hz, 2H), 6.79-6.68 (m, 3H), 6.02 (s, 2H), 2.46 (s, 3H); ^{13}C-NMR (101 MHz, $CDCl_3$): δ = 148.1, 145.4, 133.0, 132.3, 131.3, 129.8, 129.6 (q, *J* = 33.2 Hz), 128.5, 128.5, 126.8, 125.4 (q, *J* = 3.6 Hz), 124.1 (q, *J* = 272.1 Hz), 120.8, 108.3, 108.2, 102.1, 85.9, 69.5, 21.9; IR (Neat): ν_{max} 2913, 2236, 1613, 1483, 1373, 1319, 1248, 1168, 1122, 1104, 1068, 933, 812, 840, 736, 681 cm^{-1}, 658;HR-MS (ES-TOF): *m/z*: calcd for $C_{23}H_{16}NO_4SNaF_3$: 482.0650 found 482.0643 $[M+Na]^+$.

***N*-(4-Iodophenyl)-*N*-((4-(trifluoromethyl)phenyl)ethynyl)methanesulfonamide (1h)**

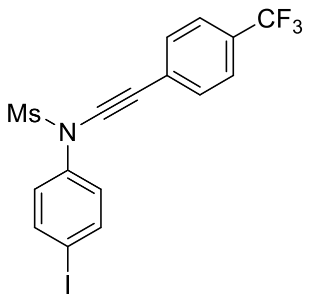

Following **GP7** using sulfonamide **V** (2.00 g, 6.72 mmol) and alkyne **H** (191 mg, 1.12 mmol). After purification by flash chromatography [^{n}hexane:EtOAc 9:1] ynamide **1h** was isolated as a white solid (247 mg, 47%); mp: 56-58 °C; ^{1}H-NMR (300 MHz, $CDCl_3$): δ = 7.79 (d, *J* = 8.8 Hz, 2H), 7.58 (d, *J* = 8.4 Hz, 2H), 7.52 (d, *J* = 8.4 Hz, 2H), 7.32 (d, *J* = 8.8 Hz, 2H), 3.17 (s, 3H); ^{13}C-NMR (101 MHz, $CDCl_3$): δ = 138.9, 138.3, 131.5, 130.0 (q, *J* = 32.8 Hz), 127.3, 126.2, 125.4 (q, *J* = 3.7 Hz), 124.0 (q, *J* = 272.4 Hz), 93.9, 83.9, 70.7, 37.5; IR (Neat): ν_{max} 3027, 3010, 2932, 2242, 1752, 1717, 1614, 1563, 1479, 1449, 1364, 1321, 1209, 1161, 1119, 1075,

1010, 961, 840, 767, 739, 690 cm^{-1}; HR-MS (EI-TOF): *m/z*: calcd for $C_{16}H_{11}NO_2SF_3I$: 464.9507 found 464.9506 $[M]^+$.

Methyl 4-((*N*-phenylmethylsulfonamido)ethynyl)benzoate (1i)

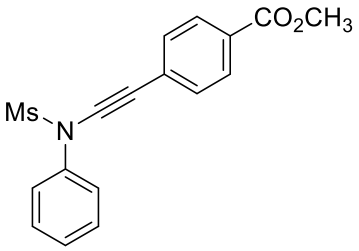

Following **GP7** using sulfonamide **R** (1.85 g, 10.8 mmol) and alkyne **I** (288 mg, 1.80 mmol). After purification by flash chromatography [^{n}hexane:EtOAc 4:1] ynamide **1i** was isolated as a white solid (533 mg, 90%); mp: 148-150 °C; ^{1}H-NMR (300 MHz, $CDCl_3$): δ = 7.98 (d, *J* = 8.6 Hz, 2H), 7.62-7.55 (m, 2H), 7.52-7.35 (m, 5H), 3.92 (s, 3H), 3.17 (s, 3H); ^{13}C-NMR (101 MHz, $CDCl_3$): δ = 166.6, 138.5, 131.0, 129.8, 129.6, 129.4, 128.8, 127.4, 125.8, 85.2, 70.9, 52.3, 37.4; IR (Neat): ν_{max} 3014, 3005, 2925, 2235, 1712, 1604, 1589, 1480, 1455, 1436, 1367, 1331, 1275, 1162, 1016, 964, 929, 861, 831, 768, 744 cm^{-1}; HR-MS (EI-TOF): *m/z*: calcd for $C_{17}H_{15}NO_4S$: 329.0722 found 329.0723 $[M]^+$.

Methyl 3-((*N*-phenylmethylsulfonamido)ethynyl)benzoate (1j)

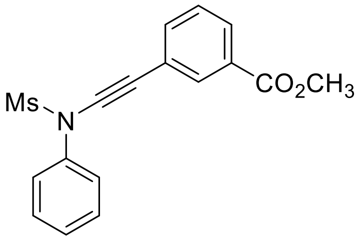

Following **GP7** using sulfonamide **R** (1.13 g, 6.60 mmol) and alkyne **J** (176 mg, 1.10 mmol). After purification by flash chromatography [^{n}hexane:EtOAc 4:1] ynamide **1j** was isolated as a white solid (300 mg, 83%); mp: 91-93 °C; ^{1}H-NMR (400 MHz, $CDCl_3$): δ = 8.11 (app t, *J* = 1.6 Hz, 1H), 7.97 (app dt, *J* = 7.9, 1.6 Hz, 1H), 7.67-7.55 (m, 3H), 7.46 (app t, *J* = 7.7 Hz, 2H), 7.43-7.36 (m, 2H), 3.92 (s, 3H), 3.17 (s, 3H); ^{13}C-NMR (101 MHz, $CDCl_3$): δ = 166.5, 138.6, 135.7, 132.7, 130.6, 129.7, 129.3, 128.7, 128.7, 125.8, 123.0, 83.0, 70.3, 52.4, 37.2; IR (Neat): ν_{max} 3017, 2925, 2242, 1716, 1590, 1490, 1441, 1358, 1289, 1255, 1203, 1160, 1108, 1088, 1072, 979, 936, 758, 689 cm^{-1}; HR-MS (ES-TOF): *m/z*: calcd for $C_{17}H_{15}NO_4SNa$: 352.0619 found 352.0627 $[M+Na]^+$.

***N*-((2-Bromophenyl)ethynyl)-*N*-(4-methoxyphenyl)-4-methylbenzenesulfonamide (1k)**

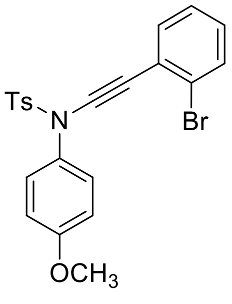

Following **GP7** using sulfonamide **A** (2.51 g, 9.05 mmol) and alkyne **K** (273 mg, 1.51 mmol). After purification by flash chromatography [^{n}hexane:EtOAc 9:1] ynamide **1k** was isolated as a white solid (167 mg, 24%); mp: 80-82 °C; ^{1}H-NMR (300 MHz, $CDCl_3$): δ = 7.66 (d, *J* = 8.3 Hz, 2H), 7.55 (dd, *J* = 7.7, 1.1 Hz, 1H), 7.42 (dd, *J* = 7.7, 1.7 Hz, 1H), 7.32-7.19 (m, 5H), 7.12 (td, *J* = 7.7, 1.7 Hz, 1H), 6.84 (d, *J* = 9.0 Hz, 2H), 3.81 (s, 3H), 2.44 (s, 3H); ^{13}C-NMR (101 MHz, $CDCl_3$): δ = 159.7, 145.1,

133.2, 133.1, 132.4, 131.5, 129.7, 129.0, 128.6, 128.1, 127.1, 125.4, 125.2, 114.4, 87.8, 69.1, 55.6, 21.8; IR (Neat): ν_{max} 3079, 2975, 2915, 2238, 1598, 1503, 1437, 1366, 1303, 1257, 1206, 1170, 1031, 901, 834, 812, 750, 670 cm^{-1}; HR-MS (ES-TOF): *m/z*: calcd for $C_{22}H_{19}NO_3SBr$: 456.0269 found 456.0266 $[M+H]^+$.

N-((4-Methoxyphenyl)ethynyl)-N-phenylmethanesulfonamide (1l)

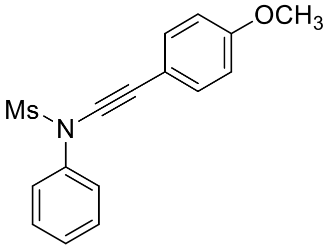

Following **GP7** using sulfonamide **R** (1.85 g, 10.8 mmol) and alkyne **L** (238 mg, 1.80 mmol). After purification by flash chromatography [^{n}hexane:EtOAc 85:15] ynamide **1l** was isolated as a white solid (347 mg, 64%); mp: 94-96 °C; ^{1}H-NMR (300 MHz, $CDCl_3$): δ = 7.58 (d, *J* = 7.9 Hz, 2H), 7.52-7.30 (m, 5H), 6.85 (d, *J* = 8.8 Hz, 2H), 3.81 (s, 3H), 3.16 (s, 3H); ^{13}C-NMR (101 MHz, $CDCl_3$): δ = 159.9, 139.1, 133.7, 129.6, 128.4, 125.7, 114.3, 114.1, 80.8, 70.9, 55.4, 36.9; IR (Neat): ν_{max} 3083, 3019, 2926, 2243, 1606, 1590, 1513, 1485, 1455, 1366, 1247, 1163, 1080, 1023, 962, 931, 899, 827, 768, 714, 689 cm^{-1}; HR-MS (ES-TOF): *m/z*: calcd for $C_{16}H_{16}NO_3S$: 302.0851 found 302.0849 $[M+H]^+$.

N-((1-Acetyl-1H-indol-3-yl)ethynyl)-N-phenylmethanesulfonamide (1m)

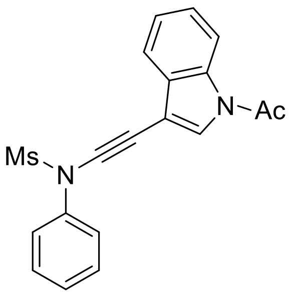

Following **GP7** using sulfonamide **R** (1.0 g, 6.0 mmol) and alkyne **AI** (180 mg, 1.0 mmol). After purification by flash chromatography [Toluene:EtOAc 95:5] ynamide **1m** was isolated as a white solid (96 mg, 27%); mp: 142-144 °C; ^{1}H-NMR (300 MHz, $CDCl_3$): δ = 8.42 (d, *J* = 8.0 Hz, 1H), 7.73-7.57 (m, 4H), 7.54-7.30 (m, 5H), 3.21 (s, 3H), 2.64 (s, 3H); ^{13}C-NMR (101 MHz, $CDCl_3$): δ = 168.3, 138.8, 135.1, 130.7, 129.8, 128.6, 128.5, 126.3, 125.7, 124.4, 120.0, 116.8, 104.3, 85.8, 62.8, 37.2, 24.0; IR (Neat): ν_{max} 3155, 3028, 2933, 1716, 1590, 1562, 1491, 1448, 1381, 1348, 1283, 1213, 1158, 1052, 996, 964, 901, 818, 742, 690; HR-MS (ES-TOF): *m/z*: calcd for $C_{19}H_{16}N_2O_3NaS$: 375.0779 found 375.0782 $[M+Na]^+$.

N-(Cyclopropylethynyl)-N-phenylmethanesulfonamide (1n)

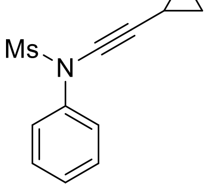

Following **GP6** using sulfonamide **R** (2.8 g, 16 mmol) and cyclopropylacetylene (270 µL, 3.2 mmol). After purification by flash chromatography [^{n}hexane:EtOAc 4:1] ynamide **1n** was isolated as a white solid (288 mg, 42%); mp: 62-64 °C; ^{1}H-NMR (300 MHz, $CDCl_3$): δ = 7.51-745 (m, 2H), 7.44-7.36 (m, 2H), 7.35-7.29 (m, 1H), 3.07 (s, 3H), 1.38 (tt, *J* = 8.0, 4.9 Hz, 1H), 0.87-0.70 (m, 4H); ^{13}C-NMR (101 MHz, $CDCl_3$): δ = 139.3, 129.5, 128.1, 125.6,

75.3, 68.8, 36.5, 9.0, -0.6; IR (Neat): ν_{max} 3157, 3014, 2933, 1715, 1562, 1490, 1448, 1381, 1348, 1283, 1214, 1157, 1052, 997, 965, 935, 900, 819, 779, 769, 742, 692 cm^{-1}; HR-MS (ES-TOF): *m/z*: calcd for $C_{12}H_{14}NO_2S$: 236.0745 found 236.0746 $[M+H]^+$.

***N*-Allyl-*N*-((4-methoxyphenyl)ethynyl)methanesulfonamide (5)**

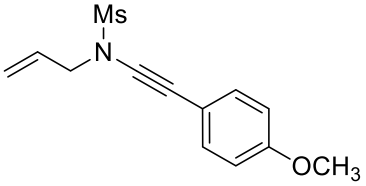

Following **GP5** using sulfonamide **Z** (82.4 mg, 0.610 mmol) and dibromoalkene **O** (265 mg, 0.908 mmol). After purification by flash chromatography [^{n}hexane:EtOAc 85:15] ynamide **5** was isolated as a pale yellow solid (124 mg, 76%); mp: 34-36 °C; ^{1}H-NMR (300 MHz, $CDCl_3$): δ = 7.36 (d, *J* = 8.9 Hz, 2H), 6.83 (d, *J* = 8.9 Hz, 2H), 6.00 (ddt, *J* = 16.7, 10.1, 6.4 Hz, 1H), 5.43 (app. dq, *J* = 16.7, 1.2 Hz, 1H), 5.36 (app. dq, *J* = 10.1, 1.2 Hz, 1H), 4.15 (app. dt, *J* = 6.4, 1.2 Hz, 2H), 3.80 (s, 3H), 3.13 (s, 3H); ^{13}C-NMR (101 MHz, $CDCl_3$): δ = 159.8, 133.7, 131.1, 120.6, 114.5, 114.1, 80.3, 70.9, 55.4, 54.5, 38.9; IR (Neat): ν = 3034, 2939, 2922, 2839, 2236, 1602, 1512, 1432, 1347, 1287, 1247, 1159, 1133, 1024, 965, 937, 840, 782 cm^{-1}; HR-MS (ES-TOF): m/z: calcd for $C_{13}H_{15}NO_3NaS$: 288.0670 found 288.0675 $[M+Na]^+$.

***N*-(4-Methoxybenzyl)-*N*-(phenylethynyl)methanesulfonamide (7a)**

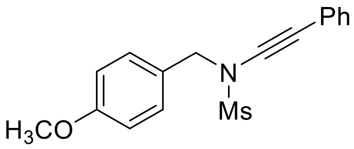

Following **GP8** using sulfonamide **X** (323 mg, 1.50 mmol) and bromophenylacetylene **AG** (326 mg, 1.80 mmol) for 18 h. After purification by gradient elution flash chromatography [^{n}hexane:EtOAc 4:1–7:3] ynamide **7a** was obtained as a pale yellow solid (658 mg, 94%); mp: 41–42 °C; ^{1}H NMR (300 MHz, $CDCl_3$) δ 7.44–7.23 (m, 7H), 6.90 (d, *J* = 8.6 Hz, 2H), 4.64 (s, 2H), 3.80 (s, 3H), 2.88 (s, 3H); ^{13}C NMR (101 MHz, $CDCl_3$) δ 160.0 (C), 131.4 (2CH), 130.5 (2CH), 128.3 (2CH), 128.0 (CH), 126.6 (C), 122.5 (C), 114.2 (2CH), 82.1 (C), 71.6 (C), 55.6 (CH_2), 55.3 (CH_3), 39.0 (CH_3); IR (neat): ν_{max} 2232, 1610, 1513, 1355, 1240, 1160, 1019, 958, 938, 920, 795, 751, 689 cm^{-1}; HR-MS (ES-TOF): *m/z* calcd for $C_{17}H_{18}NO_3S$: 316.1007 found 316.1010 $[M+H]^+$.

***N*-((4-Fluorophenyl)ethynyl)-*N*-(4-methoxybenzyl)methanesulfonamide (7b)**

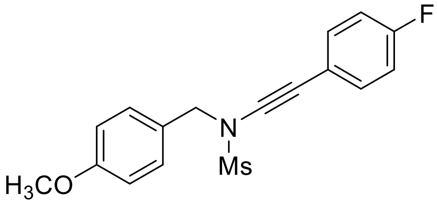

Following **GP5** using sulfonamide **X** (435 mg, 2.02 mmol) and dibromoalkene **Q** (825 mg, 2.95 mmol) with 1,4-dioxane as solvent for 18 h. After purification by flash chromatography [^{n}hexane:EtOAc 4:1] ynamide **7b** was obtained as a pale yellow solid (549 mg, 82%); mp: 54–55 °C; ^{1}H NMR (400 MHz,

$CDCl_3$) δ 7.43–7.38 (m, 2H), 7.37–7.31 (m, 2H), 7.02–6.96 (m, 2H), 6.95–6.90 (m, 2H), 4.65 (s, 2H), 3.82 (s, 3H), 2.91 (s, 3H); ^{13}C NMR (101 MHz, $CDCl_3$) δ 162.4 (d, *J* = 249.3 Hz), 160.0, 133.5 (d, *J* = 8.4 Hz), 130.5, 126.5, 118.5 (d, *J* = 3.2 Hz), 115.6 (d, *J* = 22.1 Hz), 114.2, 81.7, 70.5, 55.5, 55.3, 39.0; ^{19}F NMR (282 MHz, $CDCl_3$) δ -111.2; IR (neat): ν_{max} 3011, 2939, 2242, 1612, 1599, 1588, 1515, 1507, 1468, 1458, 1373, 1353, 1325, 1304, 1249, 1217, 1177, 1158, 1088, 1031, 1001, 968, 939, 924, 838, 819, 784, 756, 730, 710, 635 cm^{-1}; HR-MS (ES-TOF): *m/z* calcd for $C_{17}H_{16}FNO_3SNa$: 356.0733 found 356.0732 $[M+Na]^+$.

_N_-((3-Bromophenyl)ethynyl)-_N_-(4-methoxybenzyl)methanesulfonamide (7c)

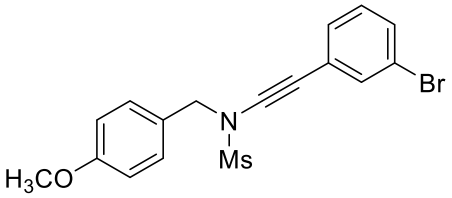

Following **GP5** using sulfonamide **X** (352 mg, 1.64 mmol) and dibromoalkene **F** (709 mg, 2.16 mmol) with 1,4-dioxane as solvent for 19 h. After purification by gradient elution flash chromatography [^{n}hexane:EtOAc 9:1–4:1] the ynamide **7c** was obtained as a viscous yellow oil (510 mg, 83%); ^{1}H NMR (400 MHz, $CDCl_3$) δ 7.48 (app. t, *J* = 1.7 Hz, 1H), 7.43–7.38 (m, 3H), 7.29–7.25 (m, 1H), 7.15 (app. t, *J* = 7.9 Hz, 1H), 6.96–6.91 (m, 2H), 4.65 (s, 2H), 3.83 (s, 3H), 2.91 (s, 3H); ^{13}C NMR (101 MHz, $CDCl_3$) δ 160.0, 133.9, 131.0, 130.5, 129.7, 129.7, 126.3, 124.6, 122.1, 114.2, 83.4, 70.5, 55.5, 55.3, 39.2; IR (neat) ν_{max}: 2933, 2837, 2233, 1612, 1590, 1553, 1513, 1476, 1464, 1441, 1408, 1354, 1323, 1304, 1248, 1159, 1109, 1084, 1068, 1030, 1009, 995, 961, 921, 784, 763, 753, 680 cm^{-1}; HR-MS (ES-TOF): *m/z* calcd. for $C_{17}H_{17}NO_3S^{79}Br$: 394.0113 found 394.0105 $[M+H]^+$.

Methyl 4-((_N_-(4-methoxybenzyl)methylsulfonamido)ethynyl)benzoate (7d)

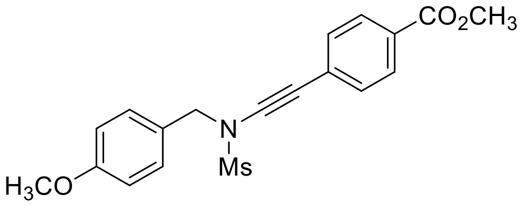

Following **GP5** using sulfonamide **X** (436 mg, 2.03 mmol) and dibromoalkene **D** (913 mg, 2.86 mmol) with 1,4-dioxane as solvent for 19 h. After purification by gradient elution flash chromatography [^{n}hexane:EtOAc 4:1–7:3] the ynamide **7d** was obtained as a viscous yellow oil (426 mg, 59%); ^{1}H NMR (400 MHz, $CDCl_3$) δ 7.98–7.93 (m, 2H), 7.44–7.35 (m, 4H), 6.96–6.90 (m, 2H), 4.67 (s, 2H), 3.91 (s, 3H), 3.82 (s, 3H), 2.93 (s, 3H); ^{13}C NMR (101 MHz, $CDCl_3$) δ 166.6, 160.1, 130.6, 130.5, 129.5, 128.9, 127.5, 126.3, 114.2, 85.2, 71.6, 55.6, 55.3, 52.2, 39.3; IR (neat) ν_{max}: 3016, 2941, 2236, 1712, 1599, 1514, 1488, 1464, 1435, 1351, 1326, 1255, 1162, 1088, 1027, 996, 963, 937, 914, 850, 818, 798, 781, 757, 742, 664 cm^{-1}; HR-MS (ESI-TOF): *m/z* calcd. for $C_{19}H_{20}NO_5S$: 374.1062 found 374.1046 $[M+H]^+$.

***N*-(3-Methoxybenzyl)-*N*-(phenylethynyl)methanesulfonamide (7e)**

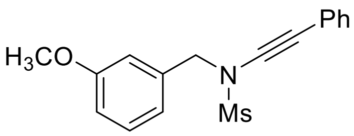

Following **GP8** using sulfonamide **Y** (245 mg, 1.14 mmol) and bromophenylacetylene **N** (258 mg, 1.43 mmol) for 24 h. After purification by gradient elution flash chromatography [^{n}hexane:EtOAc 9:1–4:1] ynamide **7e** was obtained as a pale yellow solid (345 mg, 96%); mp: 39–40 °C; ^{1}H NMR (300 MHz, $CDCl_3$) δ 7.42–7.27 (m, 6H), 7.10–7.02 (m, 2H), 6.92 (dd, *J* = 8.3, 1.9Hz, 1H), 4.69 (s, 2H), 3.82 (s, 3H), 2.95 (s, 3H); ^{13}C NMR (101 MHz, $CDCl_3$) δ 159.9 (C), 136.0 (C), 131.4 (2CH), 129.9 (CH), 128.3 (2CH), 128.0 (CH), 122.5 (C), 121.2 (CH), 114.4 (2CH), 82.0 (C), 71.6 (C), 55.8 (CH_2), 55.3 (CH_3), 39.0 (CH_3); IR (neat): ν_{max} 2237, 1599, 1493, 1469, 1352, 1327, 1261, 1159, 1034, 978, 964, 890, 853, 791, 758, 731, 688 cm^{-1}; HRMS (ES-TOF): *m/z* calcd for $C_{17}H_{18}NO_3S$: 316.1007 found 316.1009 $[M+H]^+$.

***N*-(4-Methylbenzyl)-*N*-(phenylethynyl)methanesulfonamide (7f)**

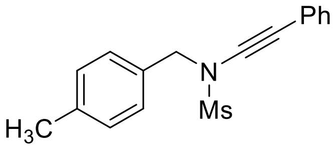

Following **GP8** using sulfonamide **W** (595 mg, 2.99 mmol) and bromophenylacetylene **N** (652 mg, 3.60 mmol) for 15 h. After purification by flash chromatography [^{n}hexane:EtOAc 9:1] ynamide **7f** was obtained as a pale orange solid (822 mg, 92%); mp: 79 °C; ^{1}H NMR (400 MHz, $CDCl_3$) δ 7.40–7.34 (m, 4H), 7.33–7.27 (m, 3H), 7.21 (d, *J* = 7.9 Hz, 2H), 4.68 (s, 2H), 2.92 (s, 3H), 2.37 (s, 3H); ^{13}C NMR (101 MHz, $CDCl_3$) δ 138.6, 131.5, 131.4, 129.5, 129.0, 128.3, 127.9, 122.6, 82.1, 71.5, 55.7, 39.0 21.2; IR (neat): ν_{max} 3022, 2929, 2236, 1724, 1513, 1491, 1465, 1450, 1345, 1323, 1232, 1161, 1120, 1055, 1020, 962, 922, 834, 806, 790, 776, 756, 744, 727, 693 cm^{-1}; HR-MS (ES-TOF): *m/z* calcd for $C_{17}H_{18}NO_2S$: 300.1058 found 300.1063 $[M+H]^+$.

***N*-(4-(Allyloxy)benzyl)-4-methyl-*N*-(phenylethynyl)benzenesulfonamide (7g)**

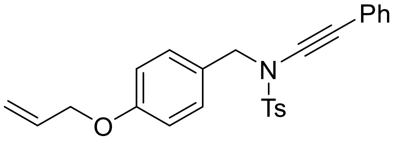

Degrades in $CDCl_3$ and upon standing, used immediately in catalysis following purification. Following **GP8** using **AA** (630 mg, 1.99 mmol) and bromophenylacetylene **N** (430 mg, 2.38 mmol) for 18 h, purified by flash chromatography [^{n}hexane:EtOAc 9:1]. **7g** was obtained as a yellow oil (639 mg, 77%); ^{1}H NMR (300 MHz, $CDCl_3$) δ 7.84–7.78 (m, 2H), 7.37–7.30 (m, 2H), 7.30–7.23 (m, 7H), 6.90–6.83 (m, 2H), 6.06 (ddt, *J* = 17.2, 10.5, 5.3 Hz, 1H), 5.42 (d app. q, *J* = 17.3, 1.6 Hz, 1H), 5.31 (d app. q, *J* = 10.5, 1.6 Hz, 1H), 4.55 (dt, *J* = 5.3 Hz, 1.5 Hz, 2H), 4.53 (s, 3H), 2.46 (s, 3H); ^{13}C NMR (101 MHz, $CDCl_3$) δ 158.9, 144.7,

134.9, 133.3, 131.3, 130.6, 129.8, 128.3, 127.9, 127.8, 126.8, 123.0, 117.9, 114.8, 82.9, 71.6, 69.0, 55.4, 21.8; IR (neat): ν_{max} 3031, 2924, 2238, 1697, 1611, 1597, 1511, 1496, 1455, 1426, 1409, 1351, 1305, 1243, 1223, 1164, 1116, 1087, 1017, 995, 926, 838, 812, 751, 731, 695, 667, 655 cm^{-1}; HR-MS (ESI-TOF): *m/z* calcd for $C_{26}H_{27}NO_4SNa$: 472.1558 found 472.1560 $[M+Na+MeOH]^+$.

N-(2-Methoxybenzyl)-4-methyl-N-(phenylethynyl)benzenesulfonamide (7h)

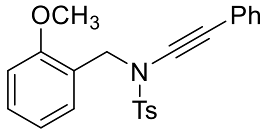

Following **GP8** using sulfonamide **AB** (588 mg, 2.02 mmol) and bromophenylacetylene **N** (432 mg, 2.39 mmol) for 21 h; purified by flash chromatography [^{n}hexane:EtOAc 9:1] before recrystallisation from a mixture of ^{n}hexane:EtOAc (4:1). **7h** was obtained as a colourless crystals (347 mg, 44%); mp: 98–99 °C; ^{1}H NMR (400 MHz, $CDCl_3$) δ 7.83 (d, *J* = 8.3 Hz, 2H), 7.35–7.31 (m, 1H), 7.33 (d, *J* = 8.3 Hz, 2H), 7.31–7.26 (m, 1H), 7.25–7.17 (m, 5H), 6.92 (app. td, *J* = 7.5, 0.8 Hz, 1H), 6.82 (d, *J* = 8.2 Hz, 1H), 4.66 (s, 2H), 3.72 (s, 3H), 2.45 (s, 3H); ^{13}C NMR (101 MHz, $CDCl_3$) δ 157.8, 144.5, 135.1, 131.1, 130.7, 129.8, 129.7, 128.3, 127.9, 127.5, 123.3, 122.8, 120.5, 110.5, 83.3, 70.9, 55.4, 50.8, 21.8; IR (neat): ν_{max} 2927, 2246, 1597, 1494, 1463, 1358, 1326, 1288, 1250, 1169, 1123, 1097, 1087, 1024, 992, 928, 799, 754, 735, 703, 695, 684 cm^{-1}; HR-MS (ESI-TOF): *m/z* calcd for $C_{23}H_{21}NO_3SNa$: 414.1140 found 414.1143 $[M+Na]^+$.

4-Methyl-N-(2-methylbenzyl)-N-(phenylethynyl)benzenesulfonamide (7i)

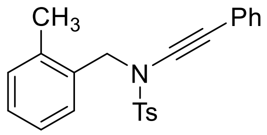

Following **GP8** using sulfonamide **AC** (826 mg, 3.00 mmol) and bromophenylacetylene **N** (749 mg, 4.14 mmol); purified by recrystallisation from EtOH. **7i** was obtained as an off white powder (270 mg, 24%); mp: 84 °C; ^{1}H NMR (300 MHz, $CDCl_3$) δ 7.85 (d, *J* = 8.3 Hz, 2H), 7.36 (d, *J* = 8.3 Hz, 2H), 7.28–7.11 (m, 9H), 4.55 (s, 2H), 2.47 (s, 3H), 2.39 (s, 3H); ^{13}C NMR (101 MHz, $CDCl_3$) δ 144.7, 137.6, 134.2, 132.0, 130.9, 130.5, 129.8, 128.7, 128.1, 127.8, 127.5, 125.9, 122.8, 82.5, 71.4, 53.5, 21.7, 19.1; IR (neat): ν_{max} 2226, 1595, 1360, 1345, 1320, 1307, 1188, 1164, 1120, 1099, 1085, 1018, 1005, 987, 934, 924, 913, 808, 760, 750, 725, 706, 692, 680, 652 cm^{-1}; HR-MS (ES+): *m/z* calcd for $C_{23}H_{21}NO_2NaS$: 398.1191 found 398.1195.

4-Methyl-N-(phenylethynyl)-N-(2,4,6-trimethylbenzyl)benzenesulfonamide (7j)

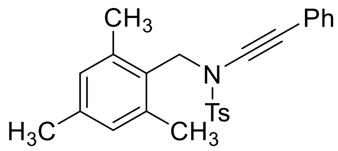

Following **GP8** using sulfonamide **AD** (817 mg, 2.02 mmol) and bromophenylacetylene **N** (465 mg, 2.57 mmol) for 20 h; purified

by recrystallisation from EtOH. **7j** was obtained as white needles (696 mg, 68%); mp: 130 °C; ^{1}H NMR (400 MHz, $CDCl_3$) δ 7.91 (d, *J* = 8.2 Hz, 2H), 7.39 (d, *J* = 8.2 Hz, 2H), 7.21–7.17 (m, 3H), 7.05–7.00 (m, 2H), 6.85 (s, 2H), 4.55 (s, 2H), 2.48 (s, 3H), 2.26 (s, 9H); ^{13}C NMR (101 MHz, $CDCl_3$) δ 144.7, 138.8, 138.4, 133.7, 130.4, 129.8, 129.1, 128.1, 127.9, 127.1, 126.5, 123.1, 82.1, 71.0, 48.6, 21.7, 21.0, 19.7; IR (neat): ν_{max} 2956, 2237, 1597, 1491, 1442, 1383, 1356, 1319, 1184, 1167, 1089, 1027, 997, 939, 888, 853, 809, 756, 730, 703, 680, 652 cm^{-1}; HR-MS (ES+): *m/z* calcd for $C_{25}H_{25}NO_2NaS$: 426.1504, found 426.1505

4-Methyl-*N*-(naphthalen-1-ylmethyl)-*N*-(phenylethynyl)benzenesulfonamide (7k)

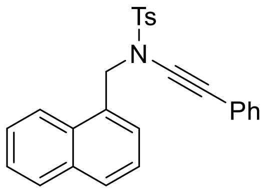

Synthesised using **GP8** using **AE** (923 mg, 2.96 mmol) and bromophenylacetylene **N** (670 mg, 3.70 mmol) for 19 h; purified by flash chromatography [^{n}hexane:EtOAc 9:1] before recrystallisation from a mixture of ^{n}hexane:EtOAc (4:1). **7k** was obtained as colourless crystals (670 mg, 55%); mp: 121–122 °C; ^{1}H NMR (400 MHz, $CDCl_3$) δ 8.22 (d, *J* = 8.4 Hz, 1H), 7.91–7.83 (m, 4H), 7.59–7.49 (m, 2H), 7.48–7.44 (m, 1H), 7.44–7.39 (m, 1H), 7.36 (d, *J* = 8.1 Hz, 2H), 7.22–7.16 (m, 3H), 7.13–7.06 (m, 2H), 4.99 (s, 2H), 2.46 (s, 3H); ^{13}C NMR (101 MHz, $CDCl_3$) δ 144.9, 134.3, 133.9, 131.9, 131.0, 129.9, 129.7, 129.6, 129.0, 128.8, 128.2, 128.0, 127.7, 126.9, 126.1, 125.1, 123.7, 122.9, 82.7, 72.0, 53.7, 21.8; **7k** was seen to degrade in $CDCl_3$. IR (neat): ν_{max} 3068, 2227, 1595, 1450, 1356, 1342, 1306, 1292, 1187, 1163, 1099, 1084, 1018, 993, 930, 917, 818, 794, 787, 775, 751, 738, 714, 691, 659 cm^{-1}; HR-MS (ESI-TOF): *m/z* calcd for $C_{26}H_{21}NO_2SNa$: 434.1191 found 434.1190 $[M+Na]^+$.

***N*-Benzyl-*N*-(phenylethynyl)methanesulfonamide (S1)**

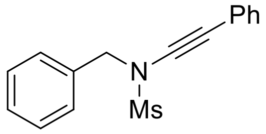

Following **GP8** using sulfonamide **AK** (0.93 g, 5.1 mmol.) and bromophenylacetylene **N** (1.1 g, 6.1 mmol) for 16 h. After purification by flash chromatography (^{n}hexane:EtOAc [9:1 - 7:3]) ynamide **SI** was obtained as a pale yellow solid (1.24 g, 87%); mp: 60 - 61 °C; ^{1}H NMR (300 MHz, $CDCl_3$) δ 7.53 – 7.27 (m, 10H), 4.72 (s, 2H), 2.94 (s, 3H); IR (neat): ν_{max} 2927, 2235, 1498, 1458, 1443, 1349, 1306, 1157, 1121, 1055, 966, 945, 791, 755, 748, 690 cm^{-1}.

Spectroscopic data in accordance with the literature.[34]

Reaction Optimisation

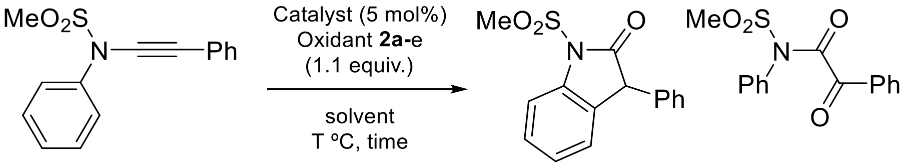

1a 3a 4a

Entry	Catalyst	**2** (1.1 eq.)	Solvent [M]	T/°C	Time/h	Yield (%)	
						**3a**	**4a** and **1a**
1	DTBPAuNTf$_2$	**2a**	CH_3NO_2 [0.025]	80	3.5	4	34
2	Ph$_3$PAuNTf$_2$	**2a**	CH_3NO_2 [0.025]	80	3	8	43
3	IPrAuNTf$_2$	**2a**	CH_3NO_2 [0.025]	80	3	29	21
4	**XPhosAuNTf$_2$**	**2a**	**CH_3NO_2 [0.025]**	**80**	**3**	**92 isolated**	**-**
5	XPhosAuNTf$_2$	**2b**	CH_3NO_2 [0.025]	80	3	8	83
6	XPhosAuNTf$_2$	**2c**	CH_3NO_2 [0.025]	80	3	31	14
7	XPhosAuNTf$_2$	**2d**	CH_3NO_2 [0.025]	80	3.5	38	47
8	XPhosAuNTf$_2$	**2e**	CH_3NO_2 [0.025]	80	3	94	-
9	XPhosAuNTf$_2$	**2f**	CH_3NO_2 [0.025]	80	3	60	32
10	XPhosAuNTf$_2$	**2a**	CH_3NO_2 [0.1]	80	3.5	74	11
11	XPhosAuNTf$_2$	**2a**	CH_3CN [0.025]	80	2	85	13
12	XPhosAuNTf$_2$	**2a**	DCE [0.025]	80	4	68	-
13	XPhosAuNTf$_2$	**2a**	PhMe [0.025]	80	4.5	52	27
14	XPhosAuNTf$_2$	**2a**	CH_3NO_2 [0.025]	rt	22/17	79/86	21/10
15	XPhosAuNTf$_2$	**2a**	CH_2Cl_2 [0.025]	rt	22/17	72	15

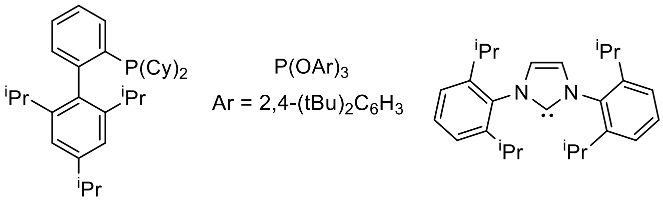

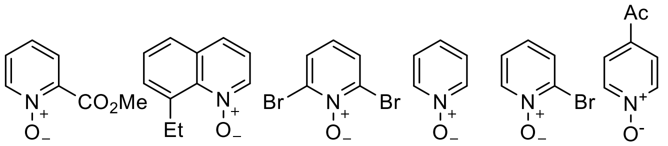

2a 2b 2c 2d 2e 2f

Yields calculated by ^{1}H NMR spectroscopy against a known quantity of 1,2,4,5-tetramethylbenzene as internal standard unless otherwise stated.

Products of the Oxidative Cyclisation Reactions

General Procedure 9 (GP9) General conditions for catalysis

2-(Methoxycarbonyl)pyridine *N*-oxide (**2a**) (1.1 eq.) was added to a solution of ynamide (1.0 eq.) and XPhosAuNTf$_2$ (5 mol%) in dry nitromethane (0.025 M). The reaction mixture was then stirred at 80 °C until consumption of the starting ynamide was observed by TLC or once appeared to progress no further by TLC. The reaction was filtered through a pipette sized column of silica gel, eluting with CH_2Cl_2 and then EtOAc. The filtrate was concentrated under reduced pressure. The residue was purified by flash chromatography to obtain isolated yields.

1-(Methylsulfonyl)-3-phenylindolin-2-one (3a)

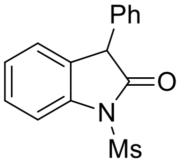

Following **GP9** using ynamide **1a** (27.1 mg, 0.100 mmol) for 3 h. After purification by flash chromatography [^{n}hexane:EtOAc 4:1] oxindole **3a** was isolated as a white solid (26.3 mg, 92%); mp: 122-124 °C; ^{1}H-NMR (300 MHz, $CDCl_3$): δ = 7.85 (d, *J* = 8.2 Hz, 1H), 7.43-7.30 (m, 4H), 7.24-7.13 (m, 4H), 4.79 (brs, 1H), 3.42 (s, 3H); ^{13}C-NMR (101 MHz, $CDCl_3$): δ = 175.1, 139.8, 135.5, 129.3, 129.3, 128.6, 128.4, 127.5, 125.8, 125.3, 113.9, 52.4, 41.8; IR (Neat): ν_{max} 3031, 3014, 2928, 1743, 1602, 1495, 1475, 1459, 1412, 1360, 1331, 1298, 1237, 1176, 1143, 1086, 1029, 958, 782, 757, 724, 695 cm^{-1}; HR-MS (EI-TOF): *m/z*: calcd for $C_{15}H_{13}NO_3S$: 287.0616 found 287.0617 $[M]^+$.

5-Methoxy-3-phenyl-1-tosylindolin-2-one (3b)

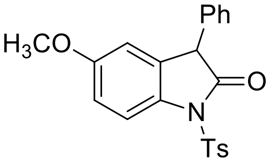

Following **GP9** using ynamide **1b** (37.7 mg, 0.100 mmol) for 5 h. After purification by flash chromatography [^{n}hexane:EtOAc 85:15] oxindole **3b** was isolated as a white solid (34.1 mg, 87%); mp: 118-120 °C; ^{1}H-NMR (300 MHz, $CDCl_3$): δ = 7.94 (d, *J* = 8.4 Hz, 2H), 7.91 (d, *J* = 9.0 Hz, 1H), 7.37-7.21 (m, 5H), 7.00-6.94 (m, 2H), 6.91 (ddd, *J* = 9.0, 2.7, 0.7 Hz, 1H), 6.66 (dd, *J* = 2.7, 1.0 Hz, 1H), 4.58 (brs, 1H), 3.74 (s, 3H), 2.41 (s, 3H); ^{13}C-NMR (101 MHz, $CDCl_3$): δ = 174.1, 157.5, 145.7, 135.8, 135.3, 133.2, 129.9, 129.2, 129.1, 128.5, 128.2, 128.1, 114.9, 114.2, 111.7, 55.8, 52.8, 21.8; IR (Neat): ν_{max} 3044, 2946, 2923, 2840, 1750, 1595, 1480, 1454, 1436, 1366, 1283, 1230, 1176, 1138, 1089, 1029, 932, 813, 799, 783, 727, 663 cm^{-1}; HR-MS (ES-TOF): *m/z*: calcd for $C_{22}H_{19}NO_4NaS$: 416.0932 found 416.0952 $[M+Na]^+$.

7-Phenyl-5-tosyl-5*H*-[1,3]dioxolo[4,5-*f*]indol-6(7*H*)-one (3c)

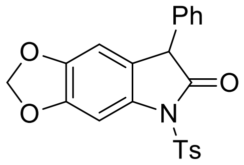

Following **GP9** using ynamide **1c** (78.3 mg, 0.200 mmol) for 3.5 h. After purification by flash chromatography [^{n}hexane:EtOAc 3:1] oxindole **3c** was isolated as a white solid (66.5 mg, 82%); mp: 184-186 °C; ^{1}H-NMR (300 MHz, $CDCl_3$): δ = 7.94 (d, *J* = 8.3 Hz, 2H), 7.62 (s, 1H), 7.40-7.19 (m, 5H), 7.02-6.88 (m, 2H), 6.55 (brs, 1H), 5.99 (s, 2H), 4.51 (brs, 1H), 2.42 (s, 3H); ^{13}C-NMR (101 MHz, $CDCl_3$): δ = 174.5, 148.2, 145.8, 145.3, 136.0, 135.2, 133.7, 129.9, 129.1, 128.4, 128.2, 128.1, 120.1, 106.0, 101.7, 97.5, 52.6, 21.8; IR (Neat): ν_{max} 3157, 3064, 3028, 2932, 1748, 1716, 1595, 1563, 1493, 1478, 1450, 1380, 1349, 1330, 1306, 1286, 1188, 1176, 1155, 1032, 965, 928, 848, 781, 744, 693 cm^{-1}; HR-MS (ES-TOF): *m/z*: calcd for $C_{22}H_{17}NO_5NaS$: 430.0725 found 430.0724 $[M+Na]^+$.

5-(Methylthio)-3-phenyl-1-tosylindolin-2-one (3d)

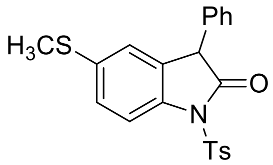

Following **GP9** using ynamide **1d** (39.4 mg, 0.100 mmol) for 3 h. After purification by flash chromatography [^{n}hexane:EtOAc 85:15] oxindole **3d** was isolated as a white solid (33.8 mg, 83%); mp: 58-60 °C; ^{1}H-NMR (300 MHz, $CDCl_3$): δ = 8.02-7.89 (m, 3H), 7.36-7.22 (m, 6H), 7.04-6.92 (m, 3H), 4.58 (brs, 1H), 2.48-2.36 (m, 6H); ^{13}C-NMR (101 MHz, $CDCl_3$): δ = 173.7, 145.9, 137.5, 135.5, 135.3, 135.2, 129.9, 129.2, 128.8, 128.5, 128.2, 128.1, 127.8, 124.4, 114.5, 52.4, 21.8, 16.7; IR (Neat): ν_{max} 3069, 3082, 2928, 1757, 1685, 1596, 1580, 1467, 1360, 1372, 1278, 1233, 1175, 1152, 1088, 1074, 952, 813, 781, 706, 663 cm^{-1}; HR-MS (ES-TOF): *m/z*: calcd for $C_{22}H_{19}NO_3NaS$: 432.0704 found 432.0689 $[M+Na]^+$.

Methyl 1-(methylsulfonyl)-2-oxo-3-phenylindoline-5-carboxylate (3e)

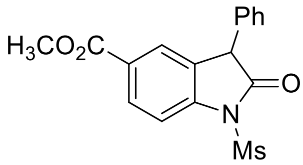

Following **GP9** using ynamide **1e** (32.9 mg, 0.100 mmol) for 5 h. After purification by flash chromatography [^{n}hexane:EtOAc 3:1] oxindole **3e** was isolated as a white solid (32.3 mg, 94%); mp: 164-166 °C; ^{1}H-NMR (300 MHz, $CDCl_3$): δ = 8.02 (ddd, *J* = 8.6, 1.8, 0.8 Hz, 1H), 7.85 (d, *J* = 8.6 Hz, 1H), 7.80 (dd, *J* = 1.8, 1.2 Hz, 1H), 7.41-7.33 (m, 3H), 7.21-7.14 (m, 2H), 4.75 (brs, 1H), 3.82 (s, 3H), 3.39 (s, 3H); ^{13}C-NMR (101 MHz, $CDCl_3$): δ = 174.8, 166.2, 143.3, 134.8, 131.3, 129.5, 128.7, 128.6, 127.8, 127.4, 127.1, 113.6, 52.4, 52.2, 42.1; IR (Neat): ν_{max} 3025, 2960, 2934, 1754, 1719, 1613, 1478, 1445, 1434, 1430, 1368, 1357, 1333, 1292, 1273, 1244, 1165, 1144, 1113, 1099, 967, 840, 766, 721, 693 cm^{-1}; HR-MS (ES-TOF): *m/z*: calcd for $C_{17}H_{15}NO_5NaS$: 368.0569 found 368.0584 $[M+Na]^+$.

7-Methyl-3-phenyl-1-tosylindolin-2-one (3f)

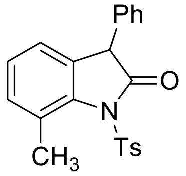

Oxidant **2a (**22.5 mg, 0.147 mmol) was added in portions (1.5 mg, 0.010 mmol, 0.1 eq) every 15 min to a solution of ynamide **1f** (36.1 mg, 0.100 mmol) and XPhosAuNTf$_2$ (4.8 mg, 5% mmol) in dry nitromethane (4 mL) at 80 °C. After the addition was completed (3.5 h) the reaction was stirred for further 30 min, filtered through a pad of silica gel, concentrated and purified by silica gel flash chromatography [^{n}hexane:EtOAc 9:1] affording oxindole **3f** as a white solid (17.9 mg, 47%); mp: 86-88 °C; ^{1}H-NMR (300 MHz, CDCl$_3$): δ = 7.85 (d, *J* = 8.4 Hz, 2H), 7.34-7.18 (m, 6H), 7.14 (t, *J* = 7.5 Hz, 1H), 7.10-7.02 (m, 2H), 6.98 (d, *J* = 7.5 Hz, 1H), 4.62 (brs, 1H), 2.55 (s, 3H), 2.39 (s, 3H); ^{13}C-NMR (101 MHz, CDCl$_3$): δ = 176.5, 145.2, 139.5, 135.7, 132.5, 129.7, 129.5, 129.0, 129.0, 128.6, 128.6, 128.0, 127.7, 125.8, 122.8, 52.9, 22.1, 21.8 IR (Neat): ν_{max} 3057, 2931, 1759, 1597, 1454, 1362, 1266, 1170, 1121, 1086, 1014, 932, 813, 768, 731, 696, 666 cm^{-1}; HR-MS (ES-TOF): *m/z*: calcd for $C_{22}H_{19}NO_3NaS$: 400.0983 found 400.0981 $[M+Na]^+$.

5-Tosyl-7-(4-(trifluoromethyl)phenyl)-5*H*-[1,3]dioxolo[4,5-*f*]indol-6(7*H*)-one (3g)

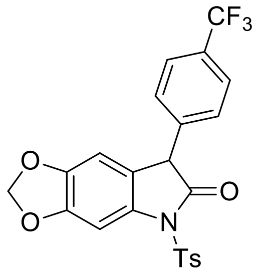

Following **GP9** using ynamide **1g** (45.9 mg, 0.100 mmol) for 3 h. After purification by flash chromatography [^{n}hexane:EtOAc 85:15] oxindole **3g** was isolated as a white solid (26.6 mg, 56%); mp: 176-178 °C; ^{1}H-NMR (300 MHz, CDCl$_3$): δ = 7.93 (d, *J* = 8.4 Hz, 2H), 7.64 (s, 1H), 7.51 (d, *J* = 8.4 Hz, 2H), 7.31 (d, *J* = 8.1 Hz, 2H), 7.09 (d, *J* = 8.1 Hz, 2H), 6.53 (brs, 1H), 6.01 (s, 2H), 4.58 (brs, 1H), 2.42 (s, 3H); ^{13}C-NMR (101 MHz, CDCl$_3$): δ = 173.7, 148.6, 146.0, 145.5, 139.8, 135.1, 133.9, 130.5 (q, *J* = 32.5 Hz), 130.0, 128.8, 128.1, 126.1 (q, *J* = 3.6 Hz), 124.0 (q, *J* = 270.0 Hz), 118.9, 105.8, 101.9, 97.7, 52.3, 21.8; IR (Neat): ν = 2915, 1744, 1619, 1598, 1476, 1458, 1371, 1325, 1158, 1067, 1036, 935, 837, 811, 739, 663 cm^{-1}; HR-MS (ES-TOF): *m/z*: calcd for $C_{23}H_{17}NO_5F_3S$: 476.0780 found 476.0781 $[M+H]^+$.

5-Iodo-1-(methylsulfonyl)-3-(4-(trifluoromethyl)phenyl)indolin-2-one (3h)

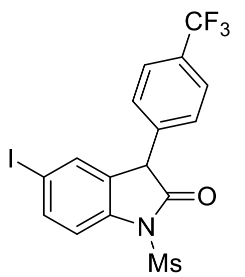

Following **GP9** using ynamide **1h** (46.5 mg, 0.100 mmol) for 2 h. After purification by flash chromatography [^{n}hexane:EtOAc 4:1] oxindole **3h** was isolated as a white solid (24.5 mg, 51%); mp: 212-214 °C; ^{1}H-NMR (300 MHz, CDCl$_3$): δ = 7.72 (dd, *J* = 8.7, 1.1 Hz, 1H), 7.69-7.61 (m, 3H), 7.49 (app. t, *J* = 1.1 Hz, 1H), 7.32 (d, *J* = 8.1 Hz, 2H), 4.83 (brs, 1H), 3.43 (s, 3H); ^{13}C-NMR (101 MHz, CDCl$_3$): δ = 173.4, 139.5, 138.6,

138.5, 134.5, 131.1 (q *J* = 32.4 Hz), 129.0, 128.8, 126.5 (q, *J* = 3.7 Hz), 123.9 (q, *J* = 272.3 Hz), 116.0, 88.9, 51.7, 42.0; IR (Neat): ν_{max} 3028, 2937, 1744, 1619, 1599, 1462, 1420, 1368, 1324, 1278, 1237, 1158, 1126, 1106, 1067, 1018, 967, 908, 837, 817, 769, 661 cm^{-1}; HR-MS (ES-TOF): *m/z*: calcd for $C_{16}H_{11}NO_3NaSF_3I$: 503.9354 found 503.9358 $[M+Na]^+$.

When the reaction was carried out adding the oxidant **2a** (0.12 mmol) in portions (0.01 mmol, 0.1 eq.) every 15 min, oxindole **3h** was isolated (32.8 mg, 68%).

Methyl 4-(1-(methylsulfonyl)-2-oxoindolin-3-yl)benzoate (3i)

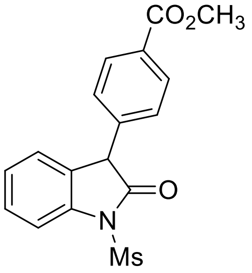

Following **GP9** using ynamide **1i** (32.9 mg, 0.100 mmol) for 2 h. After purification by flash chromatography [^{n}hexane:EtOAc 7:3] oxindole **3i** was isolated as a white solid (23.8 mg, 69%); mp: 178-180 °C; ^{1}H-NMR (300 MHz, $CDCl_3$): δ = 8.03 (d, *J* = 8.4 Hz, 2H), 7.86 (d, *J* = 8.2 Hz, 1H), 7.40 (app. td, *J* = 8.2, 1.1 Hz, 1H), 7.34-7.13 (m, 5H), 4.86 (brs, 1H), 3.91 (s, 3H), 3.42 (s, 3H); ^{13}C-NMR (101 MHz, $CDCl_3$): δ = 174.4, 166.6, 140.3, 139.8, 130.5, 130.3, 129.6, 128.7, 126.7, 125.7, 125.5, 114.1, 52.4, 52.3, 41.8; IR (Neat): ν_{max} 3032, 2955, 2936, 1739, 1723, 1608, 1462, 1435, 1364, 1282, 1241, 1169, 1154, 1110, 1020, 962, 852, 769, 740, 698, 683, 656 cm^{-1}; HR-MS (EI-TOF): *m/z*: calcd for $C_{17}H_{15}NO_5S$: 345.0671 found 345.0677 $[M]^+$.

Methyl 3-(1-(methylsulfonyl)-2-oxoindolin-3-yl)benzoate (3j)

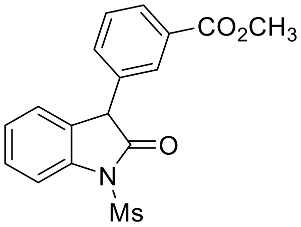

Following **GP9** using ynamide **1j** (32.9 mg, 0.100 mmol) for 45 min. After purification by flash chromatography [^{n}hexane:EtOAc 4:1] oxindole **3j** was isolated as a white solid (28.6 mg, 83%); mp: 139-141 °C; ^{1}H-NMR (400 MHz, $CDCl_3$): δ = 8.01 (app dt, *J* = 7.7, 1.3 Hz, 1H), 7.88 (app t, *J* = 1.3 Hz, 1H), 7.86 (d, *J* = 8.3 Hz, 1H), 7.45 (app t, *J* = 7.7 Hz, 1H), 7.42-7.34 (m, 2H), 7.22 (app td, *J* = 7.7, 0.7 Hz, 1H), 7.19-7.15 (m, 1H), 4.85 (brs, 1H), 3.90 (s, 3H), 3.42 (s, 3H); ^{13}C-NMR (101 MHz, $CDCl_3$): δ = 174.6, 166.6, 139.7, 135.8, 133.2, 131.2, 129.7, 129.6, 129.5, 129.5, 126.8, 125.7, 125.5, 114.0, 52.4, 52.1, 41.8; IR (Neat): ν_{max} 2936, 1752, 1723, 1610, 1467, 1446, 1430, 1352, 1298, 1237, 1173, 1142, 1082, 986, 968, 778 cm^{-1}; HR-MS (ES-TOF): *m/z*: calcd for $C_{17}H_{15}NO_5SNa$: 368.0569 found 368.0567 $[M+Na]^+$.

3-(2-Bromophenyl)-5-methoxy-1-tosylindolin-2-one (3k)

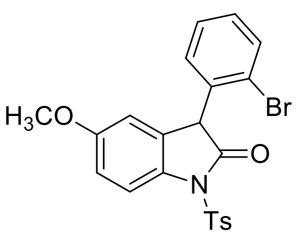

Oxidant **2a (**19.5 mg, 0.127 mmol) was added in portions (1.5 mg, 0.010 mmol, 0.1 eq) every 15 min to a solution of ynamide **1k** (45.6 mg, 0.100 mmol) and XPhosAuNTf$_2$ (4.8 mg, 5% mmol) in dry nitromethane (4 mL) at 80 °C. After 24 h the reaction was filtered and purified by flash chromatography [^{n}hexane:EtOAc 85:15] affording oxindole **3k** as a white solid (29.3 mg, 62%); mp: 104-106 °C; ^{1}H-NMR (300 MHz, CDCl$_3$, 23 °C): δ = 7.99 (d, *J* = 8.0 Hz, 2H), 7.91 (d, *J* = 8.9 Hz, 1H), 7.61 (brs, 1H), 7.44-7.27 (m, 3H), 7.23-6.99 (m, 1.5 H), 6.88 (dd, *J* = 8.9, 2.2 Hz, 1H), 6.69 (brs, 0.5 H), 6.58-6.27 (m, 1H), 5.32 (brs, 0.6 H), 4.68 (brs, 0.4 H), 3.72 (s, 3H), 2.42 (s, 3H); ^{1}H-NMR (400 MHz, C$_2$D$_2$Cl$_4$, 110 °C): δ = 8.01 (d, *J* = 8.3 Hz, 2H), 7.92 (d, *J* = 9.0 Hz, 1H), 7.60 (d, *J* = 7.6 Hz, 1H), 7.35 (d, *J* = 8.3 Hz, 2H), 7.27-7.13 (m, 2H), 6.95 (dd, *J* = 9.0, 2.5 Hz, 1H), 6.84 (brs, 1H), 6.65 (s, 1H), 5.12 (brs, 1H), 3.77 (s, 3H), 2.47 (s, 3H); ^{13}C-NMR (101 MHz C$_2$D$_2$Cl$_4$, 80 °C): δ = 172.9, 157.3, 145.6, 135.9, 135.0, 133.4, 132.9, 130.5, 129.6, 129.6, 128.7, 127.9, 127.8, 124.4, 114.5, 113.9, 111.2, 55.7, 53.1, 21.5; IR (Neat): ν_{max} 2928, 2867, 1749, 1594, 1479, 1436, 1359, 1282, 1227, 1139, 1088, 1030, 959, 907, 812, 778, 749, 732, 705, 664 cm^{-1}; HR-MS (ES-TOF): *m/z*: calcd for $C_{22}H_{18}NO_4S^{79}BrNa$: 494.0038 found 494.0037 [M+Na]$^+$.

3-(4-Methoxyphenyl)-1-(methylsulfonyl)indolin-2-one (3l)

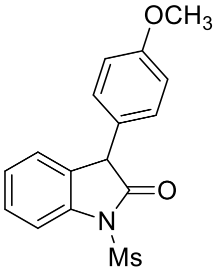

Oxidant **2a** (37.2 mg, 0.24 mmol) was added in portions (3.1 mg, 0.020 mmol, 0.1 eq;) every 10 min to a solution of ynamide **1l** (60.3 mg, 0.200 mmol) and XPhosAuNTf$_2$ (9.5 mg, 5% mmol) in dry nitromethane (8 mL) at 80 °C. After the addition was completed the reaction was stirred for further 10 min, filtered through a pad of silica gel, concentrated and purified by silica gel flash chromatography [Toluene:EtOAc 97:3] affording oxindole **3l** as a white solid (49.5 mg, 78%); mp: 154-156 °C; ^{1}H-NMR (300 MHz, CDCl$_3$): δ = 7.84 (d, *J* = 8.4 Hz, 1H), 7.42-7.31 (m, 1H), 7.22-7.17 (m, 2H), 7.11 (d, *J* = 8.7 Hz, 2H), 6.89 (d, *J* = 8.7 Hz, 2H), 4.74 (brs, 1H), 3.80 (s, 3H), 3.41 (s, 3H); ^{13}C-NMR (101 MHz, CDCl$_3$): δ = 175.4, 159.7, 139.6, 129.6, 129.1, 127.7, 127.4, 125.7, 125.2, 114.7, 113.8, 55.4, 51.6, 41.7; IR (Neat): ν_{max} 3026, 2930, 1741, 1605, 1360, 1327, 1251, 1237, 1176, 1143, 1085, 1028, 961, 830, 754, 633 cm^{-1}; HR-MS (ES-TOF): *m/z*: calcd for $C_{16}H_{16}NO_4S$: 318.0800 found 318.0799[M+H]$^+$.

When the reaction was carried out following **GP9** using ynamide **1l** (60.3 mg, 0.200 mmol) for 30 min, co-elution of overoxidation product and oxindole was observed on purification preventing isolated yields. By analysis of the ^{1}H-NMR of the crude reaction mixture against a known amount of internal standard (1,2,4,5-tetramethylbenzene) the yields were: 43% oxindole **3l**, 31% assigned as overoxidation product **4** with 23% of starting ynamide **1l**.

3-(1-Acetyl-1*H*-indol-3-yl)-1-(methylsulfonyl)indolin-2-one (3m)

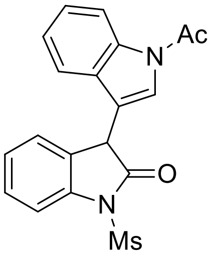

Following **GP9** using ynamide **1m** (35.2 mg, 0.100 mmol) at 0.020 M concentration (5 mL of dry nitromethane) for 2 h. After purification by flash chromatography [^{n}hexane:EtOAc 85:15] oxindole **3m** was isolated as a white solid (20.8 mg, 56%); mp: 198-200 °C; ^{1}H-NMR (400 MHz, $CDCl_3$): δ = 8.43 (d, *J* = 8.0 Hz, 1H), 7.91 (d, *J* = 8.3 Hz, 1H), 7.46-7.30 (m, 3H), 7.29-7.17 (m, 4H), 5.04 (brs, 1H), 3.45 (s, 3H), 2.62 (s, 3H); ^{13}C-NMR (101 MHz, $CDCl_3$): δ = 174.2, 168.5, 139.7, 136.3, 129.6, 128.7, 126.2, 126.1, 125.5, 125.4, 124.5, 124.1, 119.3, 117.0, 116.5, 114.1, 44.6, 41.9, 24.1; IR (Neat): ν = 3123, 2926, 2859, 1752, 1699, 1605, 1462, 1448, 1389, 1361, 1327, 1284, 1225, 1173, 1139, 1121, 1088, 1017, 972, 932, 759, 746 cm^{-1}; HR-MS (ES-TOF): *m/z*: calcd for $C_{19}H_{16}N_2O_4NaS$: 391.0728 found 391.0711 $[M+Na]^+$.

When the reaction was carried out using ynamide **1m** (35.2 mg, 0.100 mmol), $XPhosAuNTf_2$ (4.8 mg, 5% mol) at 0.0125 M concentration (8 mL of dry nitromethane), at 85 °C and adding the oxidant **2a** (18.0 mg, 0.118 mmol) in portions (3.0 mg, 0.020 mmol, 0.2 eq) every 30 min, oxindole **3m** was isolated in 81% (29.9 mg).

2-Cyclopropylidene-*N*-(methylsulfonyl)-*N*-phenylacetamide (4)

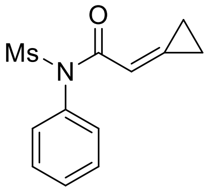

Following **GP9** using ynamide **1n** (47.1 mg, 0.200 mmol) and oxidant **2a** (36.8 mg, 0.240 mmol) for 48 h. After purification by flash chromatography [Toluene:EtOAc 9:1] methylenecyclopropane **4** was isolated as a white solid (15.8 mg, 31%); mp: 116-118 °C; ^{1}H-NMR (300 MHz, $CDCl_3$): δ = 7.52-7.43 (m, 3H), 7.37-7.30 (m, 2H), 5.62 (app. t, *J* = 1.2 Hz, 1H), 2.32-2.28 (m, 2H), 2.23-2.19 (m, 2H); ^{13}C-NMR (101 MHz, $CDCl_3$): δ = 162.2, 149.6, 139.9, 135.4, 130.6, 130.3, 129.7, 41.9, 30.4, 27.4; IR (Neat): ν_{max} 2941, 1673, 1661, 1585, 1488, 1342, 1315, 1284, 1245, 1157, 1125, 1107, 958, 906, 764, 735, 697 cm^{-1}; HR-MS (ES-TOF): *m/z*: calcd for $C_{12}H_{14}NO_3S$: 252.0694 found 252.0691 $[M+H]^+$.

1-(4-Methoxyphenyl)-3-(methylsulfonyl)-3-azabicyclo[3.1.0]hexan-2-one (6)

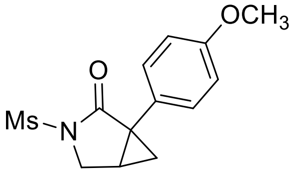

Following **GP9** using ynamide **5** (26.5 mg, 0.1 mmol) for 5 h. After purification by flash chromatography [^{n}hexane:EtOAc 65:35] **6** was isolated as a pale brown solid (23.8 mg, 85%); mp: 152-154 °C; ^{1}H-NMR (300 MHz, $CDCl_3$): δ = 7.31 (d, *J* = 8.7 Hz, 2H), 6.88 (d, *J* = 8.7 Hz, 2H), 4.02-3.92 (m, 2H), 3.80 (s, 3H), 3.26 (s, 3H), 2.41-2.31 (m, 1H), 1.57 (dd, *J* = 7.7, 5.0 Hz, 1H), 1.32 (app. t, *J* = 5.0 Hz, 1H); ^{13}C-NMR (101 MHz, $CDCl_3$): δ = 174.0, 159.5, 130.2, 126.0, 114.2, 55.5, 47.1, 40.6, 34.5, 20.3, 19.8; IR (neat): ν_{max} = 3037, 3023, 2942. 2925, 2845, 2240, 1650, 1604, 1514, 1366, 1347, 1291, 1250, 1067, 1023, 967, 938, 809 cm^{-1}; HR-MS (EI-TOF): *m/z* calcd for $C_{13}H_{15}NO_4S$: 281.0722 found 281.0729 $[M]^+$.

Reactions of *N*-benzyl-*N*-(phenylethynyl)methanesulfonamide

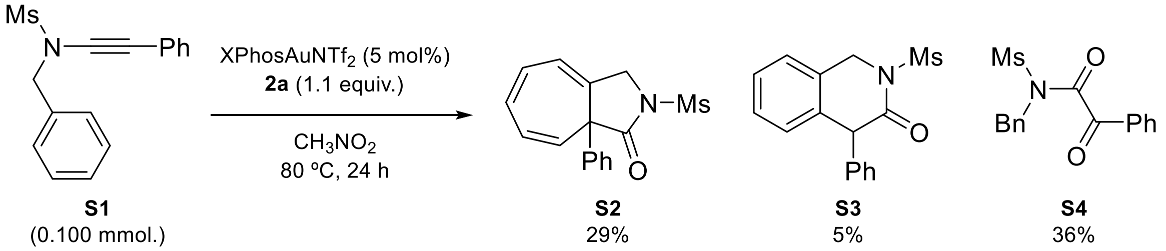

Exposure of *N*-benzyl substituted ynamide **S1**, featuring no electron donor group, yielded mixtures of cyclohepatriene **S2** and dihydroisoquinolinone **S3** and by-product **S4**. As isolated yields were compromised due to difficult separation from co-eluting impurities, the yields reported above were calculated from ^{1}H NMR spectra of the crude reaction mixtures using 1,2,4,5-tetramethylbenzene as an internal standard**.**

2-(Methylsulfonyl)-8a-phenyl-2,3-dihydrocyclohepta[*c*]pyrrol-1(8a*H*)-one (S2)

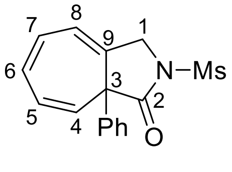

Following **GP9** using **S1** (116 mg, 0.405 mmol) for 16 h. Once complete the crude residue was purified by flash chromatography [^{n}hexane:EtOAc 7:3] to yield an off-white solid containing co-eluting impurities which was re-crystallised from CH_2Cl_2/^{n}hexane to yield **S2** as white crystals (15.6 mg, 13%); mp: 211 °C; ^{1}H NMR (400 MHz, $CDCl_3$) δ 7.26 – 7.19 (m, 5H, *Ph*), 6.51 (d, *J* = 6.3 Hz, 1H, H_8), 6.40 (dd, *J* = 9.6, 6.2 Hz, 1H, H_5), 6.35 (dd, *J* = 10.8, 6.3 Hz, 1H, H_7), 6.28 (dd, *J* = 10.8, 6.2 Hz, 1H, H_6), 5.72 (d, *J* = 9.6 Hz, 1H, H_4), 4.81 (d, *J* = 14.9 Hz, 1H, H_{1a}), 4.71 (d, *J* = 14.9 Hz, 1H, H_{1b}), 3.27 (s, 3H, *Ms*); ^{13}C NMR (101 MHz, $CDCl_3$) δ 175.0 (C, C_2), 136.9 (C, *Ph*), 129.9 (CH, C_6), 128.7 (CH, C_7), 128.0 (2CH, *Ph*), 127.8 (CH, *Ph*), 127.3 (CH, C_5), 126.9 (2CH, *Ph*), 124.5 (C, C_9), 122.2 (CH, C_8), 120.9 (CH, C_4), 56.1 (C, C_3), 49.8 (CH_2, C_1), 40.6 (CH_3, *Ms*); IR (neat): ν_{max} 3021, 2928, 1725, 1651, 1492, 1463, 1449, 1384, 1364, 1345,

1334, 1245, 1211, 1183, 1159, 1137, 1046, 967, 898, 849, 838, 775, 754 cm^{-1}, HR-MS (ES-TOF): *m/z* calcd for $C_{16}H_{16}NO_3S$: 302.0851 found 302.0862 $[M+H]^+$.

Spectroscopic data for compounds **S3** and **S4** were identical to those reported in literature.[37]

6-Methoxy-2-(methylsulfonyl)-8a-phenyl-3,8a-dihydrocyclohepta[*c*]pyrrol-1(*2H*)-one (8a)

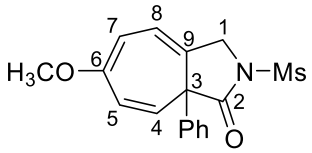

Following **GP9** using **7a** (63.1 mg, 0.200 mmol) for 6 h. After purification by flash chromatography [^{n}hexane:EtOAc 7:3] **8a** was isolated as a white solid (52.5 mg, 79%); mp: 164-65°C; ^{1}H NMR (400 MHz, $CDCl_3$) δ 7.27–7.19 (m, 5H, *Ph*), 6.38 (app. dt, *J* = 7.5, 1.9 Hz, 1H, H_8), 6.16 (dd, *J* = 10.4, 2.1 Hz, 1H, H_5), 5.80 (d, *J* = 10.4 Hz, 1H, H_4), 5.54 (d, *J* = 7.5 Hz, 1H, H_7), 4.73 (dd, *J* = 14.1, 1.9 Hz, 1H, H_1), 4.63 (dd, *J* = 14.1, 1.4 Hz, 1H, H_1), 3.44 (s, 3H, O*Me*), 3.25 (s, 3H, *Ms*); ^{13}C NMR (101 MHz, $CDCl_3$) δ 175.0 (C, C_2), 158.8 (C, C_6), 137.1 (C, *Ph*), 128.2 (2CH, *Ph*), 127.8 (CH, *Ph*), 126.9 (2CH, *Ph*), 123.2 (CH, C_5), 121.1 (CH, C_8), 121.1 (CH, C_4), 103.6 (CH, C_7), 54.7 (CH_3, O*Me*), 49.7 (CH_2, C_1), 40.5 (CH_3, *Ms*) (*2 quaternary carbons unaccounted*); IR (neat): ν_{max} 3014, 2934, 2840, 1695, 1615, 1503, 1459, 1341, 1322, 1279, 1239, 1107, 1032, 965, 909, 855, 752, 696, 594, 573 cm^{-1}; HR-MS (ESI-TOF): *m/z* calcd for $C_{17}H_{17}NO_4SNa$: 354.0776 found 354.0774 $[M+Na]^+$.

Severe broadening of the [5.3.0]-ring junction quaternary resonances in the ^{13}C NMR spectra was seen at ambient temperature, with resolution seen at 80 °C.[38] Identification of the 2 un-observed quaternary carbons, likely from norcaradiene–cycloheptatriene valence tautomerism, was achieved by recording the ^{13}C NMR spectra at 80 °C in 1,1,2,2-tetrachloroethane. Quaternary alkenyl carbon (C_9) was observed at 115.4 ppm and the quaternary alkyl carbon (C_3) was observed at 54.7 ppm. Recording ^{13}C NMR spectra at -50 °C in $CDCl_3$ resulted in alteration of the chemical shift and significant broadening for the ^{13}C signals which had appeared, when recorded at 22°C, at 158.8 (C_6), 137.1 (C, *Ph*), 126.9 (2CH, *Ph*), 123.2 (CH, C_5), 121.1 (CH, C_8), 121.1 (CH, C_4) and 103.6 (CH, C_7). The remaining resonances were unaffected, corresponding with changes in the environment of the carbon atoms in the cycloheptatriene and the phenyl substituent.

In $C_2Cl_4D_2$:

^{1}H NMR (300 MHz, $C_2Cl_4D_2$) δ 7.25 (m, 5H), 6.40 (d, *J* = 7.6 Hz, 1H), 6.15 (dd, *J* = 10.2, 1.7 Hz, 1H), 5.70 (d, *J* = 10.2 Hz, 1H), 5.58 (d, *J* = 7.6 Hz, 1H), 4.72 (d, *J* = 14.2 Hz, 1H), 4.62 (d, *J* = 14.2 Hz, 1H), 3.45 (s, 3H), 3.26 (s, 3H); ^{13}C NMR (101 MHz, $C_2Cl_4D_2$, 80 °C) δ

174.9 (C), 158.7 (C), 136.8 (C), 128.0 (2CH), 127.6 (CH), 126.9 (2CH), 122.5 (CH), 121.1 (CH), 119.1 (CH), 115.4 (C), 104.7 (CH), 54.8 (CH_3), 54.7 (C), 49.6 (CH_2), 40.5 (CH_3).

8a-(4-Fluorophenyl)-6-methoxy-2-(methylsulfonyl)-3,8a-dihydrocyclohepta[*c*]pyrrol-1(*2H*)-one (8b)

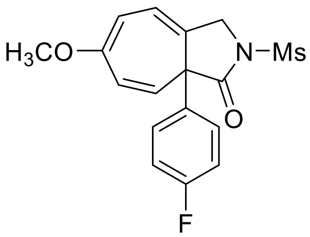

Following **GP9** using **7b** (66.7 mg, 0.20 mmol) for 6 h. After purification by gradient elution flash silica gel chromatography [^{n}hexane:EtOAc 7:3- 3:2] **8b** was obtained as an off white solid (51.0 mg, 73%); mp: 187–189 °C; ^{1}H NMR (400 MHz, $CDCl_3$) δ 7.23 (m, 2H), 6.94–6.88 (m, 2H), 6.40 (dt, *J* = 7.6, 1.8 Hz, 1H), 6.14 (dd, *J* = 10.2, 2.1 Hz, 1H), 5.73 (d, *J* = 10.2 Hz, 1H), 5.58 (dd, *J* = 7.6, 2.1 Hz, 1H), 4.71 (dd, *J* = 14.1, 1.8 Hz, 1H), 4.62 (dd, *J* = 14.1, 1.8 Hz, 1H), 3.46 (s, 3H), 3.26 (s, 3H); ^{13}C NMR (101 MHz, $CDCl_3$) δ 174.8, 162.3 (d, *J* = 246.8 Hz), 158.8, 132.5, 128.7 (d, *J* = 8.3 Hz), 122.6, 121.3, 119.5 (v. br.), 115.1 (d, *J* = 21.6 Hz), 104.2, 54.7, 49.7, 40.7 (*2 quaternary carbons unaccounted*); ^{19}F NMR (282 MHz, $CDCl_3$) δ -114.3; IR (neat): ν_{max} 3004, 2926, 2841, 1721, 1650, 1575, 1502, 1467, 1380, 1346, 1237, 1228, 1216, 1161, 1140, 1065, 1015, 973, 826, 810, 768, 712 cm^{-1}; HR-MS (ESI-TOF): *m/z* calcd for $C_{17}H_{16}FNO_4SNa$: 372.0682 found 372.0683 $[M+Na]^+$.

8a-(3-Bromophenyl)-6-methyl-2-(methylsulfonyl)-3,8a-dihydrocyclohepta[*c*]pyrrol-1(*2H*)-one (8c)

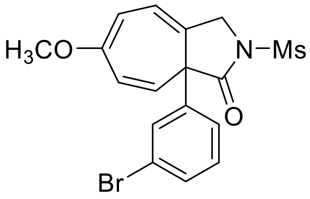

Following **GP9** using **7c** (76.4 mg, 0.20 mmol) for 18 h. After purification by gradient elution flash silica gel chromatography [^{n}hexane:EtOAc 7:3–3:2] **7c** was isolated as white crystals (49.6 mg, 62%); mp: 128–129 °C; ^{1}H NMR (400 MHz, $CDCl_3$) δ 7.39–7.35 (m, 2H), 7.27–7.23 (m, 1H), 7.13 (app. t, *J* = 8.0 Hz, 1H), 6.46 (dt, *J* = 7.5, 1.9 Hz, 1H), 6.20 (dd, *J* = 10.3, 1.9 Hz, 1H), 5.79 (d, *J* = 10.3 Hz, 1H), 5.63 (dd, *J* = 7.5, 1.3 Hz, 1H), 4.75 (dd, *J* = 14.2, 1.3 Hz, 1H), 4.65 (dd, *J* = 14.2, 1.3 Hz, 1H) , 3.50 (s, 3H), 3.29 (s, 3H); ^{13}C NMR (101 MHz, $CDCl_3$) δ 174.4, 158.8, 139.2, 131.1, 130.0, 129.7, 125.8, 123.4, 122.2, 121.6, 121.6, 104.0, 54.8, 49.6, 40.6 (*2 quaternary carbons unaccounted*); IR (neat): ν_{max} 1725, 1564, 1470, 1471, 1352, 1325, 1281, 1236, 1218, 1167, 1140, 1061, 1015, 967, 911, 812, 764, 729 cm^{-1}; HR-MS (ES-TOF): *m/z* calcd. for $C_{17}H_{17}NO_4S^{79}Br$: 410.0062 found 410.0052 $[M+H]^+$.

Methyl 4-(6-methoxy-2-(methylsulfonyl)-3-oxo-2,3-dihydrocyclohepta[*c*]pyrrol-3a(1*H*)-yl)benzoate (7d)

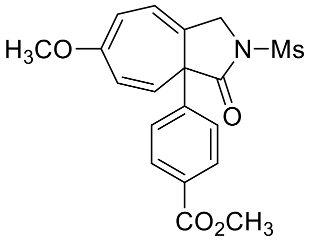

Following **GP9** using **7d** (72.3 mg, 0.200 mmol) for 18 h. After purification by gradient elution flash silica gel chromatography [^{n}hexane:EtOAc 7:3–3:2] followed by re-crystallisation from Et_2O to remove trace co-eluting impurities, **8d** was obtained as white crystals (24.3 mg, 32%)**;** mp: 158–160 °C; ^{1}H NMR (400 MHz, $CDCl_3$) δ 7.91 (d, *J* = 8.4 Hz, 2H), 7.36 (d, *J* = 8.4 Hz, 2H), 6.45 (d, *J* = 7.5 Hz, 1H), 6.21 (dd, *J* = 10.3, 1.9 Hz, 1H), 5.83 (d, *J* = 10.2 Hz, 1H), 5.59 (d, *J* = 7.4 Hz, 1H), 4.78 (d, *J* = 14.3 Hz, 1H), 4.68 (d, *J* = 14.3 Hz, 1H), 3.90 (s, 3H), 3.45 (s, 3H), 3.28 (s, 3H); ^{13}C NMR (101 MHz, $CDCl_3$) δ 174.3, 166.7, 158.9, 142.1, 129.7, 129.4, 127.1, 123.5, 121.5, 103.8, 54.7, 52.1, 49.7, 40.6 (*2 quaternary carbons unaccounted*); IR (neat): ν_{max} 2951, 1729, 1716, 1562, 1469, 1435, 1409, 1378, 1338, 1277, 1243, 1223, 1162, 1137, 1116, 1015, 974, 861, 808, 781, 771, 754, 735, 713 cm^{-1}; HR-MS (ESI-TOF): *m/z* calcd for $C_{19}H_{20}NO_6S$: 390.1011 found 390.1010 [M+H]$^{+}$.

6-Methyl-2-(methylsulfonyl)-8a-phenyl-3,8a-dihydrocyclohepta[*c*]pyrrol-1(*2H*)-one (8f)

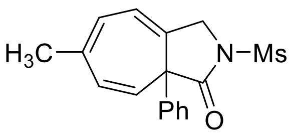

Following **GP9** using **7f** (59.9 mg, 0.200 mmol) for 20 h. After purification by gradient elution flash silica gel chromatography [^{n}hexane:EtOAc 9:1–7:3] **8f** was obtained as fine white crystals (39.4 mg, 62%); mp: 208–209 °C; ^{1}H NMR (400 MHz, $CDCl_3$) δ 7.23–7.18 (m, 5H), 6.35 (d, *J* = 6.7 Hz, 1H), 6.20 (d, *J* = 9.6 Hz, 1H), 6.09 (d, *J* = 6.7 Hz, 1H), 5.51 (d, *J* = 9.6 Hz, 1H), 4.74 (d, *J* = 14.4 Hz, 1H), 4.64 (d, *J* = 14.4 Hz, 1H), 3.27 (s, 3H), 1.81 (s, 3H); ^{13}C NMR (101 MHz, $CDCl_3$) δ 175.2, 138.8, 137.0, 129.5, 128.0, 127.7, 127.1, 126.3, 122.0, 115.4, 49.8, 40.6, 24.0 (*2 quaternary carbons unaccounted*); IR (neat): ν_{max} 3026, 2926, 1724, 1491, 1465, 1450, 1375, 1345, 1333, 1323, 1232, 1186, 1167, 1160, 1146, 1062, 1039, 1023, 971, 834, 775, 760, 743, 727, 718, 695 cm^{-1}; HR-MS (ESI-TOF): *m/z* calcd for $C_{17}H_{18}NO_3S$: 316.1007 found 316.1001 [M+H]$^{+}$.

6-(Allyloxy)-8a-phenyl-2-tosyl-3,8a-dihydrocyclohepta[*c*]pyrrol-1(*2H*)-one (8g)

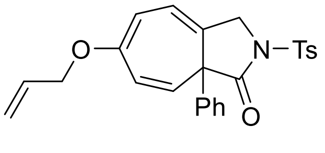

Following **GP9** using **7g** (42.7 mg, 0.102 mmol) for 8 h, purified by flash chromatography [^{n}hexane:EtOAc 9:1–17:3]. **8g** was obtained as colourless crystals (23.7 mg, 53%); mp: 154–155 °C; ^{1}H NMR (400 MHz, $CDCl_3$) δ 7.90 (d, *J* = 8.3 Hz, 2H), 7.29 (d, *J* = 8.3 Hz, 2H), 7.20–7.08

(m, 5H), 6.32 (d, J = 7.5 Hz, 1H), 6.13 (dd, J = 10.4, 1.9 Hz, 1H), 5.81–5.70 (m, 1H), 5.65 (d, J = 10.4 Hz, 1H), 5.55 (d, J = 7.5 Hz, 1H), 5.15–5.10 (m, 1H), 5.08 (s, 1H), 4.79 (d, J = 14.3 Hz, 1H), 4.63 (d, J = 14.3 Hz, 1H), 4.18–4.05 (m, 2H), 2.41 (s, 3H); ^{13}C NMR (101 MHz, $CDCl_3$) δ 173.9, 157.6, 145.5, 137.2, 134.8, 132.6, 129.8, 128.3, 128.1, 127.7, 127.0, 123.4, 121.4 (br.), 120.8, 117.6, 115.5, 105.5, 68.4, 55.4, 50.6, 21.8 *(peaks at 55.4 and 115.5 are of low intensity and could be the 2 normally missing quaternary carbons, HMBC suggests 55.4 is likely to be one)*; IR (neat): ν_{max} 2924, 1739, 1596, 1561, 1467, 1361, 1326, 1233, 1200, 1173, 1130, 1088, 1059, 1005, 943, 925, 910, 876, 829, 812, 776, 752, 735, 707, 694, 660 cm^{-1}; HR-MS (ESI-TOF): *m/z* calcd for $C_{25}H_{24}NO_4S$: 434.1426 found 434.1427 [M+H]$^+$.

4-Methoxy-8a-phenyl-2-tosyl-3,8a-dihydrocyclohepta[*c*]pyrrol-1(*2H*)-one (8h)

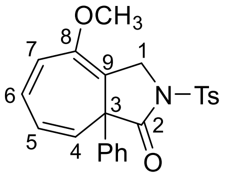

Following **GP9** using **7h** (39.1 mg, 0.100 mmol) for 4 h; purified by flash chromatography [^{n}hexane:EtOAc 17:3–8:2]. **8h** was obtained as colourless crystals (23.9 mg, 59%); mp: 143–144 °C; ^{1}H NMR (400 MHz, $CDCl_3$) δ 7.93 (d, J = 8.2 Hz, 2H, *Ts*), 7.31 (d, J = 8.2 Hz, 2H, *Ts*), 7.19–7.14 (m, 3H, *Ph*), 7.03–6.98 (m, 2H, *Ph*), 5.82 (dd, J = 8.9, 7.4 Hz, 1H, H_6), 5.73 (dd, J = 8.9, 5.5 Hz, 1H, H_5), 5.18 (d, J = 7.4 Hz, 1H, H_7), 4.63 (d, J = 11.1 Hz, 1H, H_1), 4.09 (d, J = 11.1 Hz, 1H, H_1), 3.69 (s, 3H, O*Me*), 3.15 (br. s, 1H, H_4), 2.43 (s, 3H, *Ts*); ^{13}C NMR (101 MHz, $CDCl_3$) δ 173.7, 159.1, 152.3, 145.2, 135.2, 131.2, 130.6, 129.8, 128.4, 128.0, 128.0, 126.7, 116.3, 99.8 (br.), 56.3, 48.1, 21.8 (*2 quaternary carbons unaccounted*); IR (neat): ν_{max} 3053, 2943, 1726, 1642, 1597, 1568, 1500, 1477, 1447, 1404, 1355, 1270, 1232, 1191, 1172, 1163, 1133, 1089, 1054, 1029, 1011, 995, 936, 914, 815, 737, 699, 689, 679, 659 cm^{-1}; HR-MS (ESI-TOF): *m/z* calcd for $C_{23}H_{22}NO_4S$: 408.1270 found 408.1274 [M+H]$^+$.

4-Methyl-8a-phenyl-2-tosyl-3,8a-dihydrocyclohepta[*c*]pyrrol-1(*2H*)-one (8i)

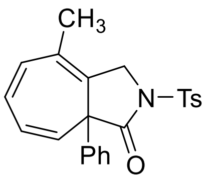

Following **GP9** using **7i** (75.3 mg, 0.201 mmol) for 18 h; purified by flash chromatography [^{n}hexane:EtOAc 9:1–17:3]. **8i** was obtained as a colourless solid (36.5 mg, 46%); mp: 183–184 °C; ^{1}H NMR (400 MHz, $CDCl_3$) δ 7.92 (d, J = 8.3 Hz, 2H), 7.29 (d, J = 8.3 Hz, 2H), 7.14–7.07 (m, 3H), 7.07–7.01 (m, 2H), 6.25 (ddd, J = 9.5, 5.8, 0.6 Hz, 1H), 6.19 (d, J = 11.0 Hz, 1H), 6.14 (dd, J = 11.0, 5.8 Hz, 1H), 5.58 (d, J = 9.5 Hz, 1H), 4.77 (d, J = 15.0 Hz, 1H), 4.62 (d, J = 15.0 Hz, 1H), 2.42 (s, 3H), 2.00 (s, 3H); ^{13}C NMR (101 MHz, $CDCl_3$) δ 174.4, 145.5, 137.6, 134.9, 133.2, 129.8, 129.5, 129.0, 128.3, 127.8, 127.6, 127.1, 126.6, 122.2, 55.7, 50.4, 21.8, 18.9 (*1 quaternary was unaccounted*); IR (neat): ν_{max} 1736, 1592, 1473, 1448, 1343, 1253,

1201, 1191, 1159, 1139, 1088, 1061, 1030, 1019, 1011, 849, 817, 791, 752, 731, 718, 698, 693, 662 cm^{-1}; HR-MS (ESI-TOF): *m/z* calcd for $C_{23}H_{21}NO_3SNa$: 414.1140 found 414.1147 $[M+Na]^+$.

4,6,8-Trimethyl-8a-phenyl-2-tosyl-3,8a-dihydrocyclohepta[*c*]pyrrol-1(*2H*)-one (8j)

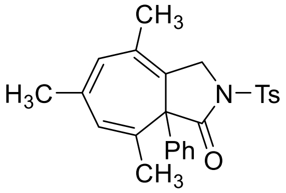

Following **GP9** using **7j** (40.5 mg, 0.100 mmol) for 5 h; purified by flash chromatography [^{n}hexane:EtOAc 9:1–17:3]. **8j** was obtained as a colourless solid (11.8 mg, 28%); mp: 191–192 °C; ^{1}H NMR (400 MHz, $CDCl_3$) δ 7.94 (d, *J* = 8.3 Hz, 2H), 7.29 (d, *J* = 8.3 Hz, 2H), 7.09–7.00 (m, 3H), 6.94–6.89 (m, 2H), 5.90 (s, 1H), 5.89 (s, 1H), 4.80 (d, *J* = 14.9 Hz, 1H), 4.64 (d, *J* = 14.9 Hz, 1H), 2.42 (s, 3H), 1.91 (s, 3H), 1.85 (d, *J* = 0.8 Hz, 3H), 1.62 (s, 3H); ^{13}C NMR (101 MHz, $CDCl_3$) δ 173.2, 145.5, 138.6, 138.1, 135.1, 131.6, 129.7, 129.4, 128.4, 128.2, 127.4, 127.1, 126.9, 126.7, 120.3, 60.2, 50.3, 23.8, 22.5, 21.8, 18.3; IR (neat): ν_{max} 1738, 1596, 1494, 1478, 1446, 1347, 1231, 1182, 1166, 1143, 1091, 1021, 909, 840, 823, 810, 760, 728, 711, 703, 693, 681, 659 cm^{-1}; HR-MS (ESI-TOF): *m/z* calcd for $C_{25}H_{25}NO_3SNa$: 442.1453 found 442.1457 $[M+Na]^+$.

3a-Phenyl-2-tosyl-1,2,3a,3b-tetrahydro-3*H*-naphtho[1',2':1,3]cyclopropa[1,2-*c*]pyrrol-3-one (8k)

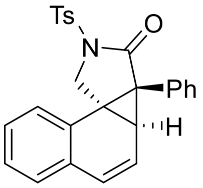

Following **GP9** using **7k** (41.2 mg, 0.100 mmol) for 4 h, purified by flash chromatography [^{n}hexane:EtOAc 95:5–4:1]. **8k** was obtained as a colourless solid (32.1 mg, 75%); mp: 108–109 °C; ^{1}H NMR (400 MHz, $CDCl_3$) δ 8.00 (d, *J* = 8.2 Hz, 2H), 7.63 (d, *J* = 7.6 Hz, 1H), 7.39 (app. td, *J* = 7.6, 1.1 Hz, 1H), 7.35 (d, *J* = 8.2 Hz, 2H), 7.24 (app. td, *J* = 7.6, 1.1 Hz, 1H), 7.10–7.04 (m, 1H), 7.04–6.95 (m, 3H), 6.73–6.69 (m, 2H), 6.23 (d, *J* = 9.7 Hz, 1H), 6.05 (dd, *J* = 9.7, 5.0 Hz, 1H), 4.78 (d, *J* = 10.4 Hz, 1H), 4.19 (d, *J* = 10.4 Hz, 1H), 2.65 (d, *J* = 5.0 Hz, 1H), 2.45 (s, 3H); ^{13}C NMR (101 MHz, $CDCl_3$) δ 173.3, 145.4, 135.2, 132.5, 132.0, 130.0, 129.1, 129.1, 128.7, 128.5, 128.4, 128.2, 127.9, 127.9, 127.7, 125.7, 121.8, 49.3, 36.1, 33.8, 31.8, 21.9; IR (neat): ν_{max} 3029, 1726, 1597, 1493, 1447, 1358, 1330, 1275, 1188, 1168, 1141, 1125, 1089, 1069, 1035, 1013, 996, 933, 810, 789, 770, 749, 719, 710, 697, 676, 658 cm^{-1}; HR-MS (ESI-TOF): *m/z* calcd for $C_{26}H_{21}NO_3SNa$: 450.1140 found 450.1137 $[M+Na]^+$.

(3bS,4S,10S,10aR)-12-Methoxy-2-(methylsulfonyl)-3a,7-diphenyl-1,2,3b,4-tetrahydro-6H,10H-4,10-ethenopyrrolo[3',4':1,3]cyclopropa[1,2-d][1,2,4]triazolo[1,2-a]pyridazine-3,6,8(3aH,7H)-trione (9)

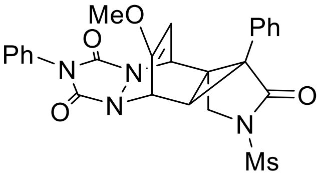

8a (33.3 mg, 0.100 mmol) was dissolved in dry CH_2Cl_2 (5 mL) at 0 °C before the dropwise addition of PTAD (16.4 mg, 0.0936 mmol) as a solution in CH_2Cl_2 (10 mL). The reaction was then stirred at 0 °C for 15 mins and r.t. for 15 mins. ^{n}Hexane was then added slowly until the solution became cloudy, the solvent was then partially removed (*ca.* 60–70%) and the solid collected by vacuum filtration to give **9** as a pale pink powder (40.4 mg, 80%); mp: 219–220 °C; ^{1}H NMR (400 MHz, DMSO) δ 7.52–7.40 (m, 3H), 7.37–7.19 (m, 7H), 5.55 (d, *J* = 6.8 Hz, 1H), 5.35 (dd, *J* = 4.4, 2.5 Hz, 1H), 4.60 (dd, *J* = 6.8, 2.5 Hz, 1H), 4.50 (d, *J* = 9.4 Hz, 1H), 4.04 (d, *J* = 9.3 Hz, 1H), 2.25 (d, *J* = 4.6 Hz, 1H); ^{13}C NMR (101 MHz, DMSO) δ 170.8, 156.6, 155.9, 153.3, 131.3, 130.9, 130.8, 130.7, 129.2, 128.7, 128.1, 127.7, 127.3, 126.2, 93.5, 55.5, 55.4, 54.3, 48.0, 45.0, 31.0, 22.2; IR (neat): ν_{max} 3017, 2927, 1768, 1732, 1709, 1642, 1498, 1401, 1366, 1304, 1265, 1243, 1215, 1173, 1141, 1010, 963, 834, 819, 774, 760, 746, 726, 704, 691 cm^{-1}; HR-MS (ESI-TOF): *m/z* calcd for $C_{25}H_{23}N_4O_6S$: 507.1338 found 507.1335 $[M+H]^+$.

7-Methoxy-2-(methylsulfonyl)-4-phenyl-1,4-dihydroisoquinolin-3(*2H*)-one (10)

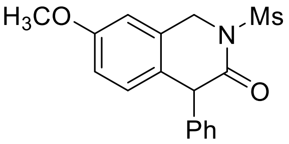

Following **GP9** using **7e** (63.1 mg, 0.200 mmol) for 6 h. After purification by gradient elution flash silica gel chromatography [^{n}hexane:EtOAc 4:1–7:3] **10** was obtained as colourless crystals (40.3 mg, 61%); mp: 99–100 °C; ^{1}H NMR (400 MHz, $CDCl_3$) δ 7.32–7.22 (m, 3H), 7.11–7.06 (m, 3H), 6.90 (dd, *J* = 8.4, 2.5 Hz, 1H), 6.85 (d, *J* = 2.5 Hz, 1H), 4.95 (d, *J* = 15.4 Hz, 1H), 4.94 (s, 1H), 4.49 (d, *J* = 15.4 Hz, 1H), 3.82 (s, 3H), 3.32 (s, 3H); ^{13}C NMR (101 MHz, $CDCl_3$) δ 171.5, 159.3, 134.9, 133.0, 129.6, 129.0, 127.9, 127.6, 126.0, 114.5, 111.6, 55.5, 54.7, 47.4, 41.9; IR (neat): ν_{max} 3014, 2934, 1695, 1615, 1503, 1459, 1432, 1342, 1323, 1279, 1239, 1159, 1107, 1032, 964, 753, 730, 696, 594, 572 cm^{-1}; HR-MS (ESI-TOF): *m/z* calcd for $C_{17}H_{17}NO_4SNa$: 354.0776 found 354.0766 $[M+Na]^+$.

7-Methyl-3-phenyl-indolin-2-one

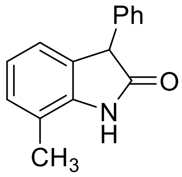

Following a modified literature method:[39] A solution of 7-methyl-3-phenyl-1-tosyl indolin-2-one (27.3 mg, 0.072 mmol) in 1,2-DME (0.7 mL) was cooled to -78 °C before sodium napthalenide solution (1.7 mL, 0.36 M in 1,2-DME) was added dropwise. Orange colouration develops on addition of up to ~0.5 mL after which time the dark green colour was sustained. The solution was stirred at -78 °C for 1 hour and then quenched by the addition of water (~ 3 mL). Extraction with EtOAc (2 × 5 mL) was followed by washing the combined organic layers with brine (5 mL), drying over Na_2SO_4, filtering and removing the solvent under reduced pressure. The residue was purified by silica gel flash chromatography [^{n}hexane:EtOAc 8:2 to 7:3; loading in the eluent] affording the oxindole as a white solid (11.0 mg, 69%); ^{1}H-NMR (400 MHz, $CDCl_3$): δ = 9.03 (br, 1H), 7.37-7.27 (m, 3H), 7.25-7.22 (m, 2H), 7.08-7.05 (m, 1H), 6.99-6.92 (m, 2H), 4.66 (s, 1H), 2.29 (s, 3H); ^{13}C NMR (101 MHz, $CDCl_3$) δ 179.0, 140.6, 136.8, 129.8, 129.3, 129.0, 128.6, 127.7, 122.8, 119.5, 53.1, 16.7 (quaternary carbon not observed); IR (Neat): ν_{max} 3055, 3029, 2922, 2849, 1695, 1624, 1599, 1454, 1326, 1302, 1210, 749, 701 cm^{-1}; HR-MS (ES-TOF): *m/z*: calcd for $C_{15}H_{14}NO$: 224.1075 found 224.1077 $[M+H]^+$.

References

[1] (a) A. K. Buzas, F. M. Istrate and F. Gagosz, *Org. Lett.*, 2007, **9**, 985; (b) N. Mézailles, L. Ricard and F. Gagosz, *Org. Lett.*, 2005, **7**, 4133; (c) Y. Odabachian, X. F. Le Goff and F. Gagosz, *Chem. Eur. J.*, 2009, **15**, 8966; (d) A. Zhdanko, M. Ströbele and M. E. Maier, *Chem. Eur. J.*, 2012, **18**, 14732.

[2] N. Nishina and Y. Yamamoto, *Synlett*, 2007, **2007**, 1767.

[3] C. Nieto-Oberhuber, S. López and A. M. Echavarren, *J. Am. Chem. Soc.*, 2005, **127**, 6178.

[4] A. González-Gómez, L. Añorbe, A. Poblador, G. Domínguez and J. Pérez-Castells, *Eur. J. Org. Chem.*, 2008, 1370.

[5] K. Born and S. Doye, *Eur. J. Org. Chem.*, 2012, 764.

[6] H. Hu, J. S. Mendoza, C. T. Lowden, L. M. Ballas and W. P. Janzen, *Bioorg. Med. Chem.*, 1997, **5**, 1873.

[7] B. R. Rosen, J. C. Ruble, T. J. Beauchamp and A. Navarro, *Org. Lett.*, 2011, **13**, 2564.

[8] H. Xu and L.-l. Fan, *Eur. J. Med. Chem.*, 2011, **46**, 364.

[9] D. H. Huh, J. S. Jeong, H. B. Lee, H. Ryu and Y. G. Kim, *Tetrahedron*, 2002, **58**, 9925.

[10] A. V. Shastin, V. N. Korotchenko, V. G. Nenajdenko and E.S. Balenkova, *Synthesis*, 2001, 2081.

[11] H. Horibe, Y. Fukuda, K. Kondo, H. Okuno, Y. Murakami and T. Aoyama, *Tetrahedron*, 2004, **60**, 10701.

[12] B. Witulski, N. Buschmann and U. Bergsträβer, *Tetrahedron*, 2000, **56**, 8473.

[13] J. S. Oakdale and D. L. Boger, *Org. Lett.*, 2010, **12**, 1132.

[14] T. Shimada, K. Mukaide, A. Shinohara, J. W. Han and T. Hayashi, *J. Am. Chem. Soc.*, 2002, **124**, 1584.

[15] T. M. Fasina, J. C. Collings, J. M. Burke, A. S. Batsanov, R. M. Ward, D. Albesa-Jove, L. Porres, A. Beeby, J. A. K. Howard, A. J. Scott, W. Clegg, S. W. Watt, C. Viney and T. B. Marder, *J. Mater. Chem.*, 2005, **15**, 690.

[16] G. T. Crisp and P. D. Turner, *Tetrahedron*, 2000, **56**, 407.

[17] A. Odedra, C.-J. Wu, T. B. Pratap, C.-W. Huang, Y.-F. Ran and R.-S. Liu, *J. Am. Chem. Soc.*, 2005, **127**, 3406.

[18] C. Körner, P. Starkov and T. D. Sheppard, *J. Am. Chem. Soc.*, 2010, **132**, 5968.

[19] R. B. Dateer, B. S. Shaibu and R.-S. Liu, *Angew. Chem Int. Ed.*, 2012, **51**, 113.

[20] A. Elangovan, Y.-H. Wang and T.-I. Ho, *Org. Lett.*, 2011, **11**, 1841.

[21] A.Torrado, S. Lopez, R. Alvarez and A. R. de Lera, *Synthesis*, 1995, 285.

[22] D. L. Usanov and H. Yamamoto, *J. Am. Chem. Soc.*, 2011, **133**, 1286.

[23] M. L.N. Rao, D. N. Jadhav and P. Dasgupta, *Org. Lett.*, 2010, **12**, 2048.

[24] D. C. Johnson II and T. S. Widlanski, *J. Org. Chem.*, 2003, **68**, 5300.

[25] Q. Xu, Q. Li, X. Zhu and J. Chen, *Adv. Synth. Catal.*, 2013, **355**, 73.

[26] J. E. Ellis, J. H. Fried, I. T. Harrison, E. Rapp and C. H. Ross, *J. Org. Chem.*, 1977, **42**, 2891.

[27] W. B. Jennings and C. J. Lovely, *Tetrahedron*, 1991, **47**, 5561.

[28] X. Cui, F. Shi, Y. Zhang and Y. Deng, *Tetrahedron Lett.*, 2010, **51**, 2048.

[29] K. Müther, J. Mohr and M. Oestreich, *Organometallics*, 2013, **32**, 6643-6646.

[30] E. J. Corey and P. L. Fuchs, *Tetrahedron Letters*, 1972, **13**, 3769.

[31] E. Alonso, D. J. Ramón and M. Yus, *Tetrahedron*, 1997, **53**, 14355.

[32] A. Coste, G. Karthikeyan, F. Couty and G. Evano, *Angew. Chem. Int. Ed.*, 2009, **48**, 4381.

[33] T. Hamada, X. Ye and S. S. Stahl, *J. Am. Chem. Soc.*, 2008, **130**, 833.

[34] Y. Zhang, R. P. Hsung, M. R. Tracey, K. C. M. Kurtz and E. L. Vera, *Org. Lett.*, 2004, **6**, 1151.

[35] P. W. Davies, A. Cremonesi and L. Dumitrescu, *Angew. Chem. Int. Ed.*, 2011, **50**, 8931.

[36] E.-I. Negishi, M. Hata and C. Xu, *Org. Lett.*, 2000, **2**, 3687.

[37] L. Li, B. Zhou, Y.-H. Wang, C. Shu, Y.-F. Pan, X. Lu, L.-W. Ye, *Angew. Chem. Int. Ed.*, 2015, **54**. 8245.

[38] A. R. Maguire, P. O'Leary, F. Harrington, S. E. Lawrence and A. J. Blake, *J. Org. Chem.*, 2001, **66**, 7166.

[39] C. P. Seath, J. W. B. Fyfe, J. J. Molloy, A. J. B. Watson, *Synthesis*, 2017, **49**, 891.

^{1}H and ^{13}C NMR Spectra of Novel Ynamides and Precursors

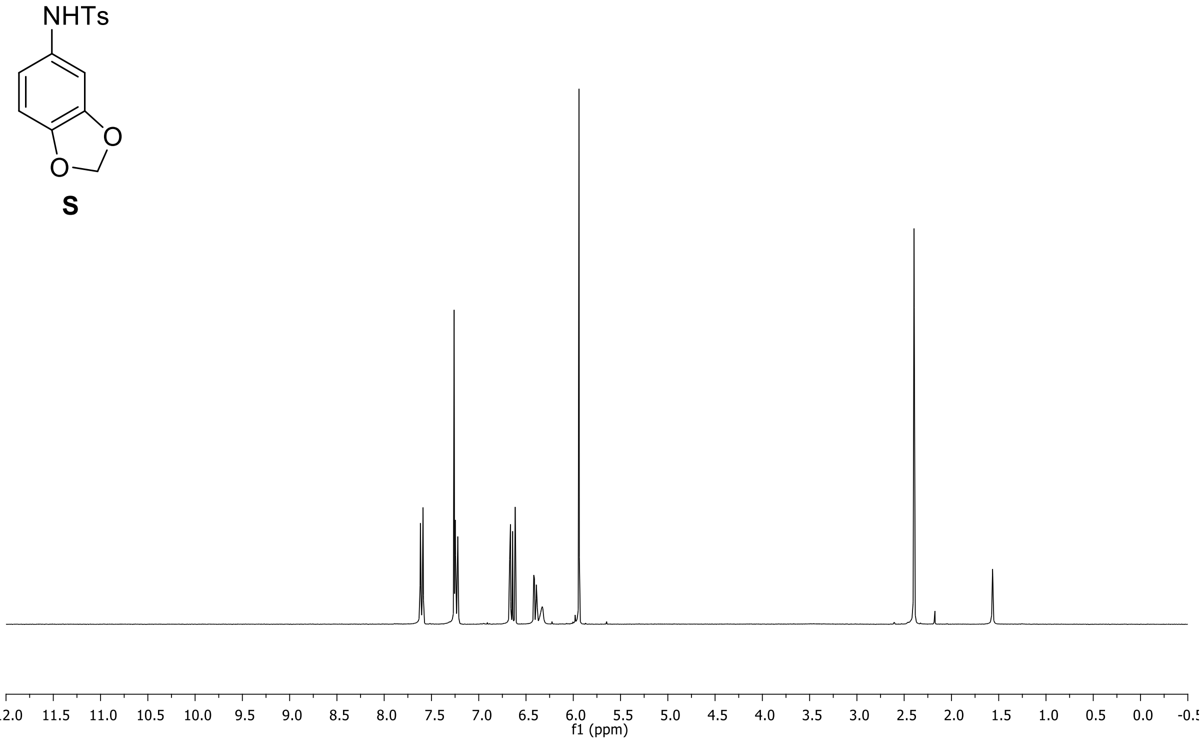

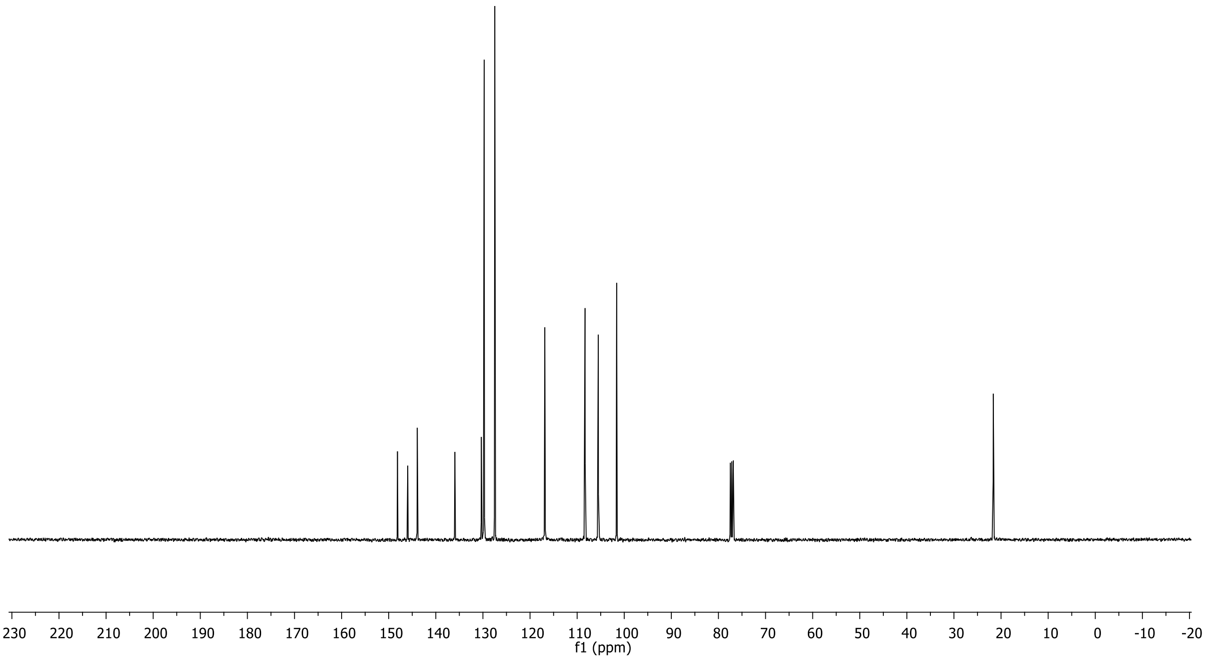

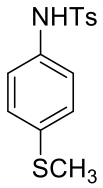
NHTs
SCH$_3$

T

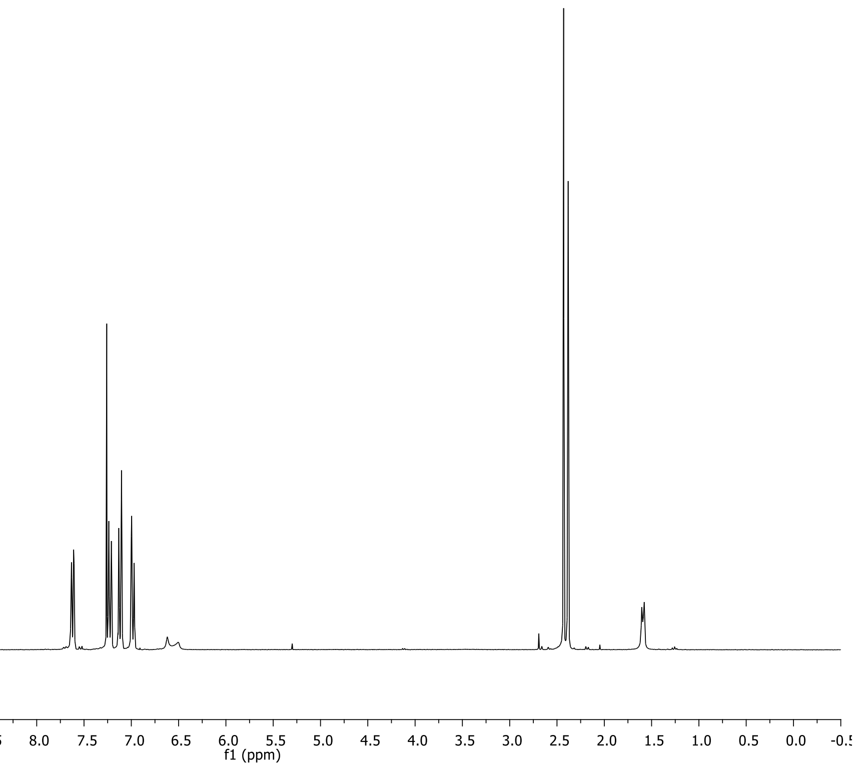
12.0 11.5 11.0 10.5 10.0 9.5 9.0 8.5 8.0 7.5 7.0 6.5 6.0 5.5 5.0 4.5 4.0 3.5 3.0 2.5 2.0 1.5 1.0 0.5 0.0
f1 (ppm)

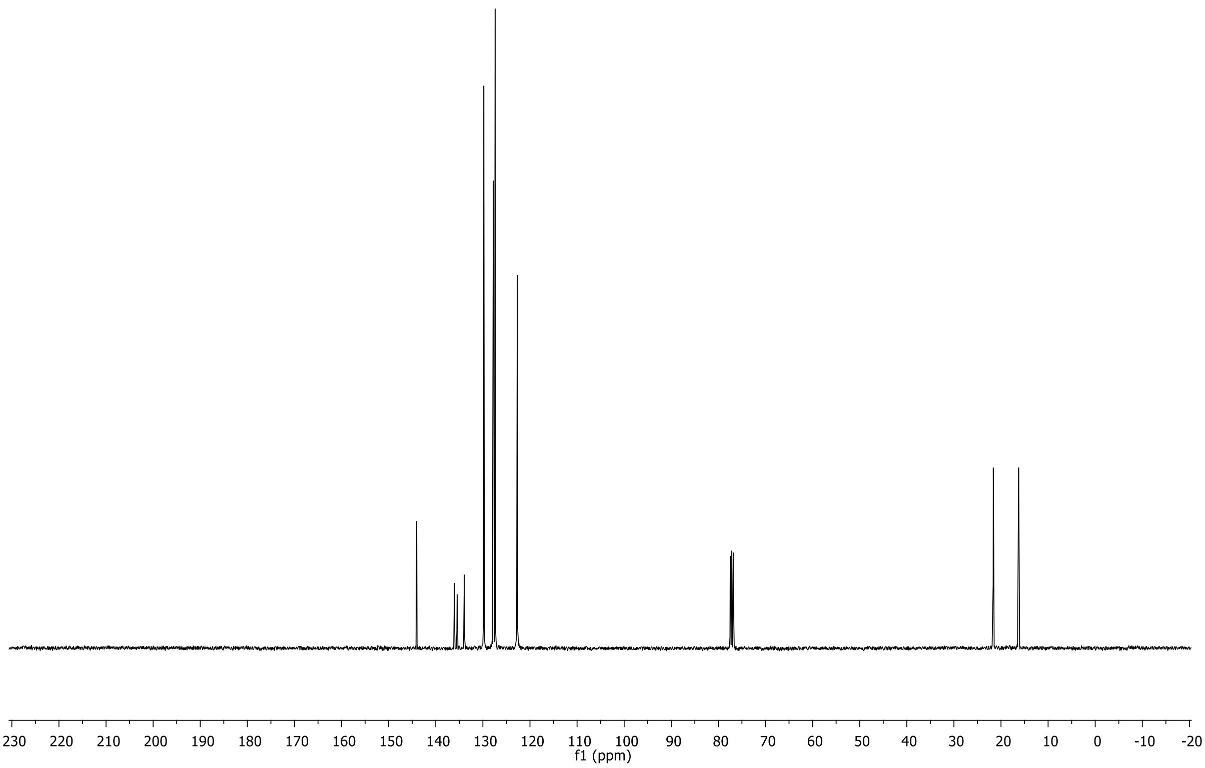
230 220 210 200 190 180 170 160 150 140 130 120 110 100 90 80 70 60 50 40 30 20 10 0 -10 -20
f1 (ppm)

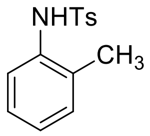

U

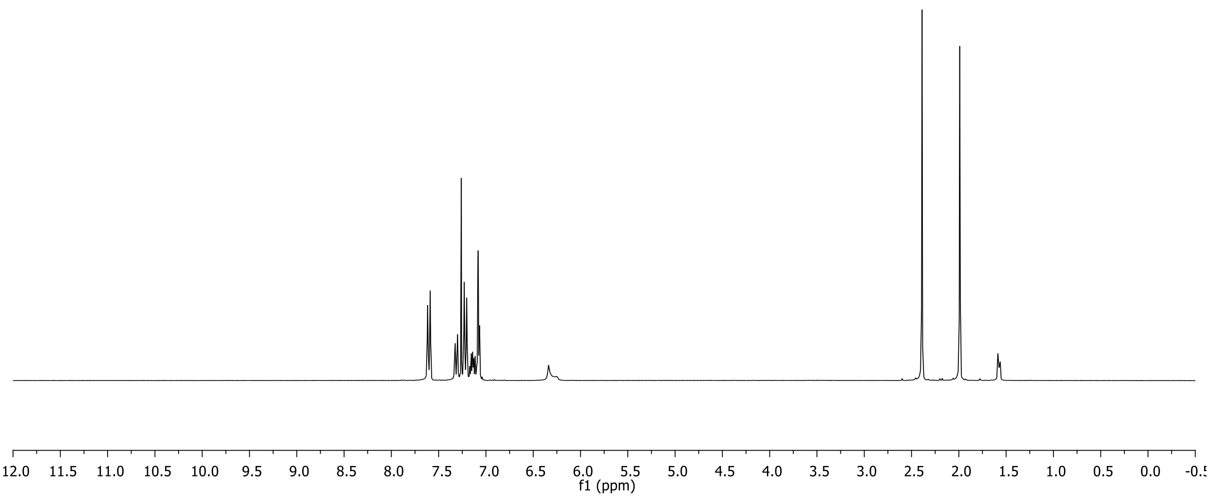

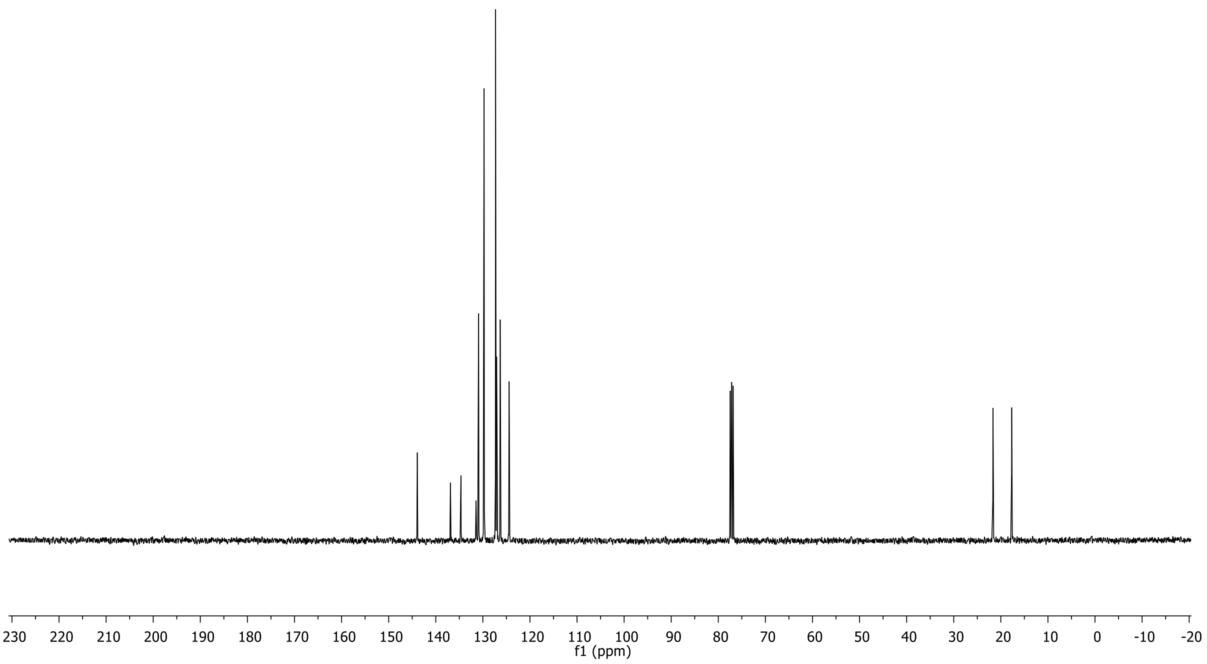

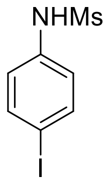
NHMs
I

V

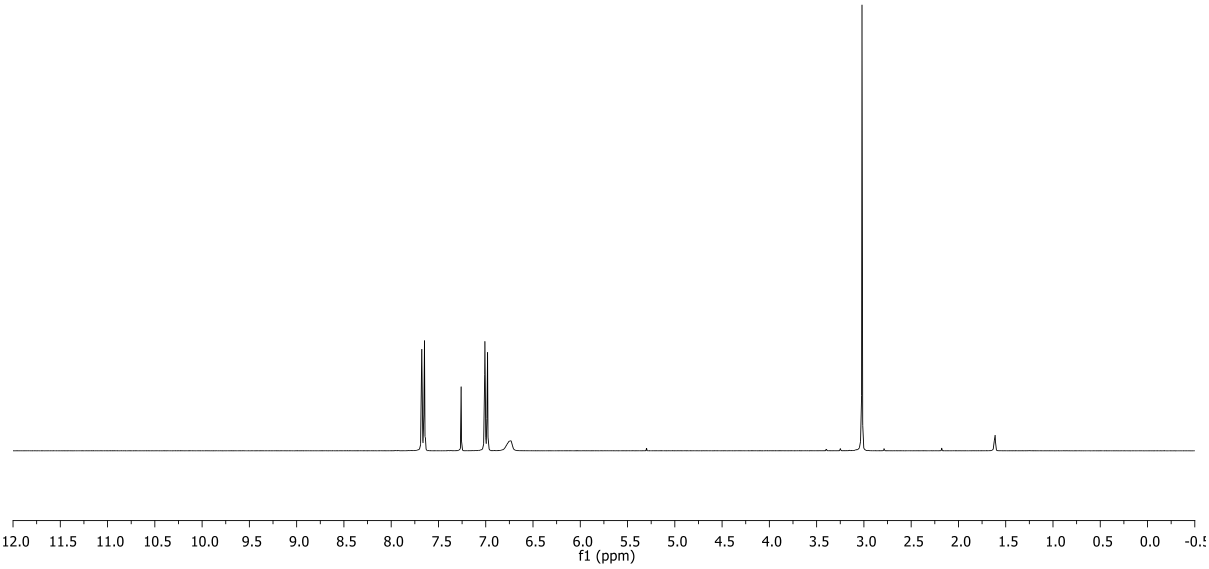
12.0 11.5 11.0 10.5 10.0 9.5 9.0 8.5 8.0 7.5 7.0 6.5 6.0 5.5 5.0 4.5 4.0 3.5 3.0 2.5 2.0 1.5 1.0 0.5 0.0
f1 (ppm)

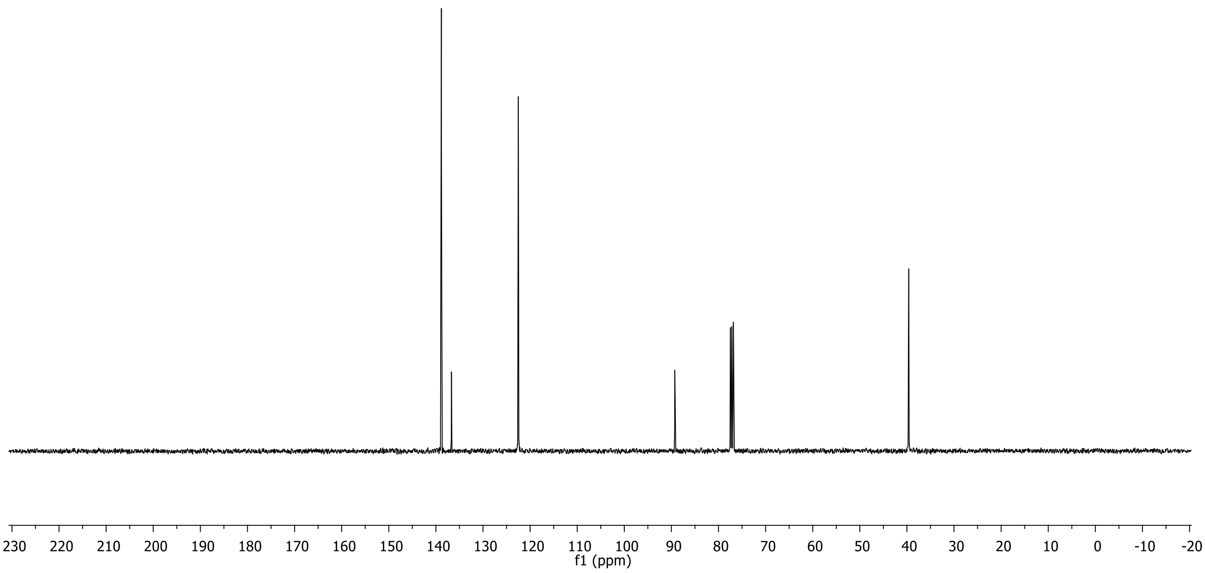
230 220 210 200 190 180 170 160 150 140 130 120 110 100 90 80 70 60 50 40 30 20 10 0 -10 -20
f1 (ppm)

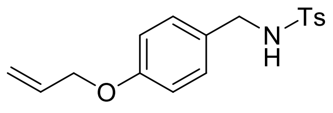

Ts
N
H
O

AA

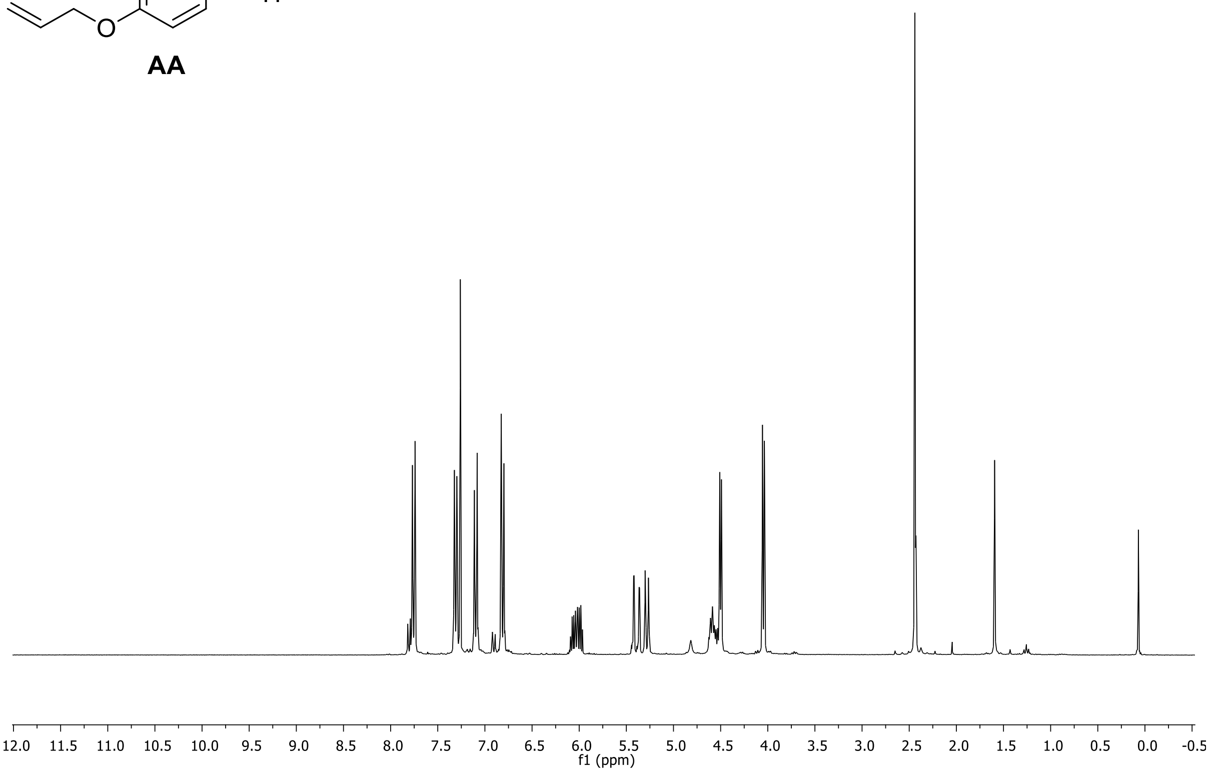

12.0 11.5 11.0 10.5 10.0 9.5 9.0 8.5 8.0 7.5 7.0 6.5 6.0 5.5 5.0 4.5 4.0 3.5 3.0 2.5 2.0 1.5 1.0 0.5 0.0 -0.5
f1 (ppm)

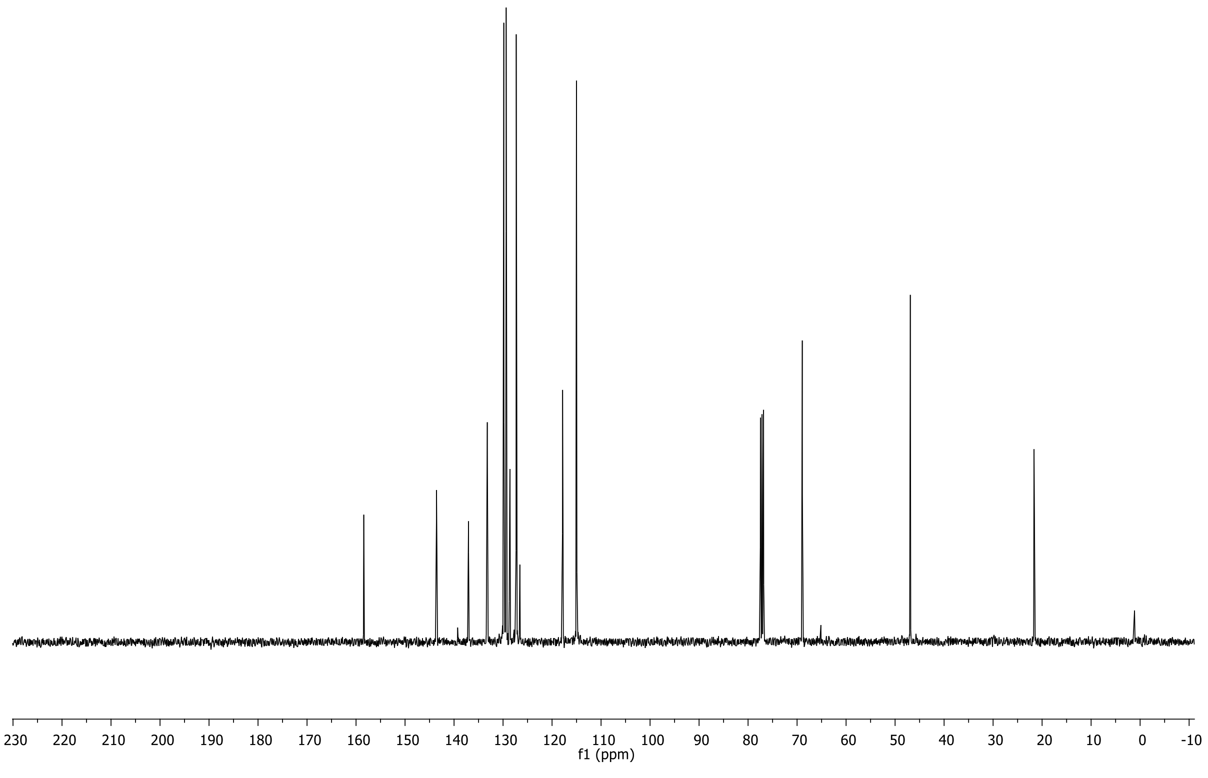

230 220 210 200 190 180 170 160 150 140 130 120 110 100 90 80 70 60 50 40 30 20 10 0 -10
f1 (ppm)

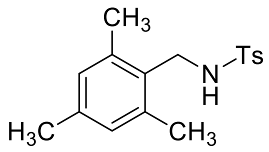

AD

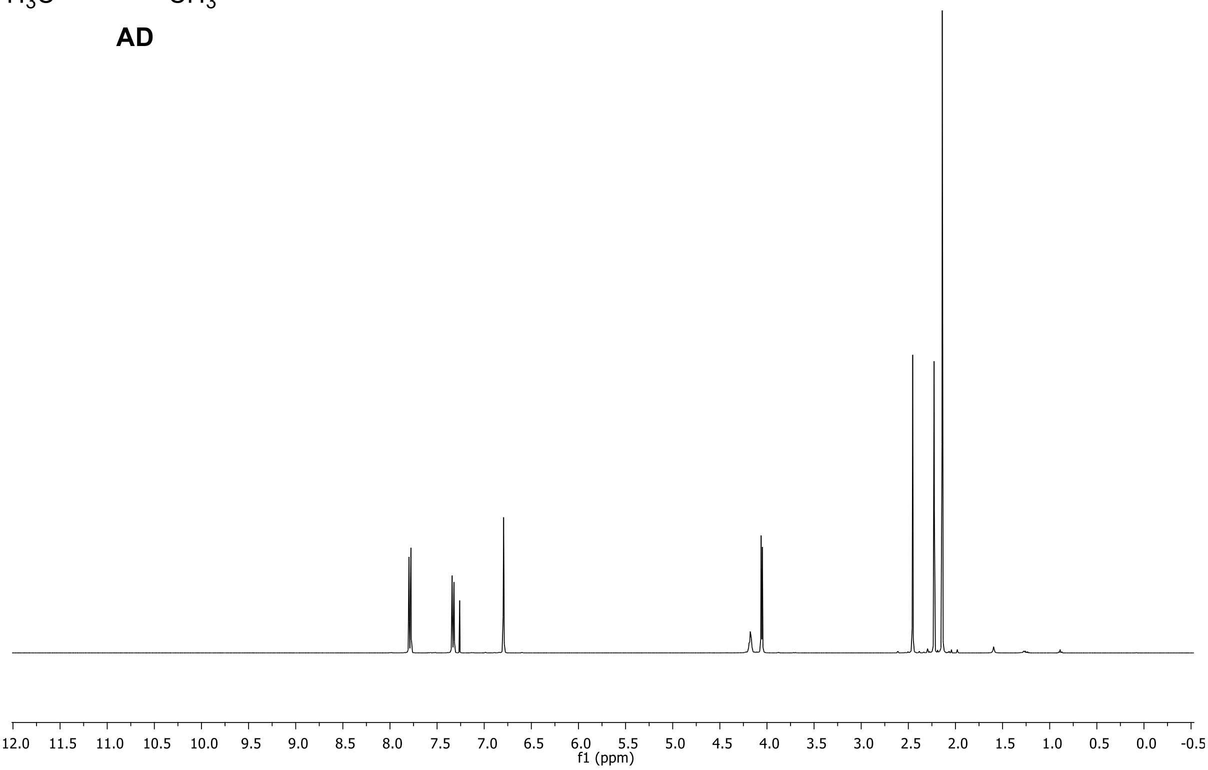
12.0 11.5 11.0 10.5 10.0 9.5 9.0 8.5 8.0 7.5 7.0 6.5 6.0 5.5 5.0 4.5 4.0 3.5 3.0 2.5 2.0 1.5 1.0 0.5 0.0 -0.5
f1 (ppm)

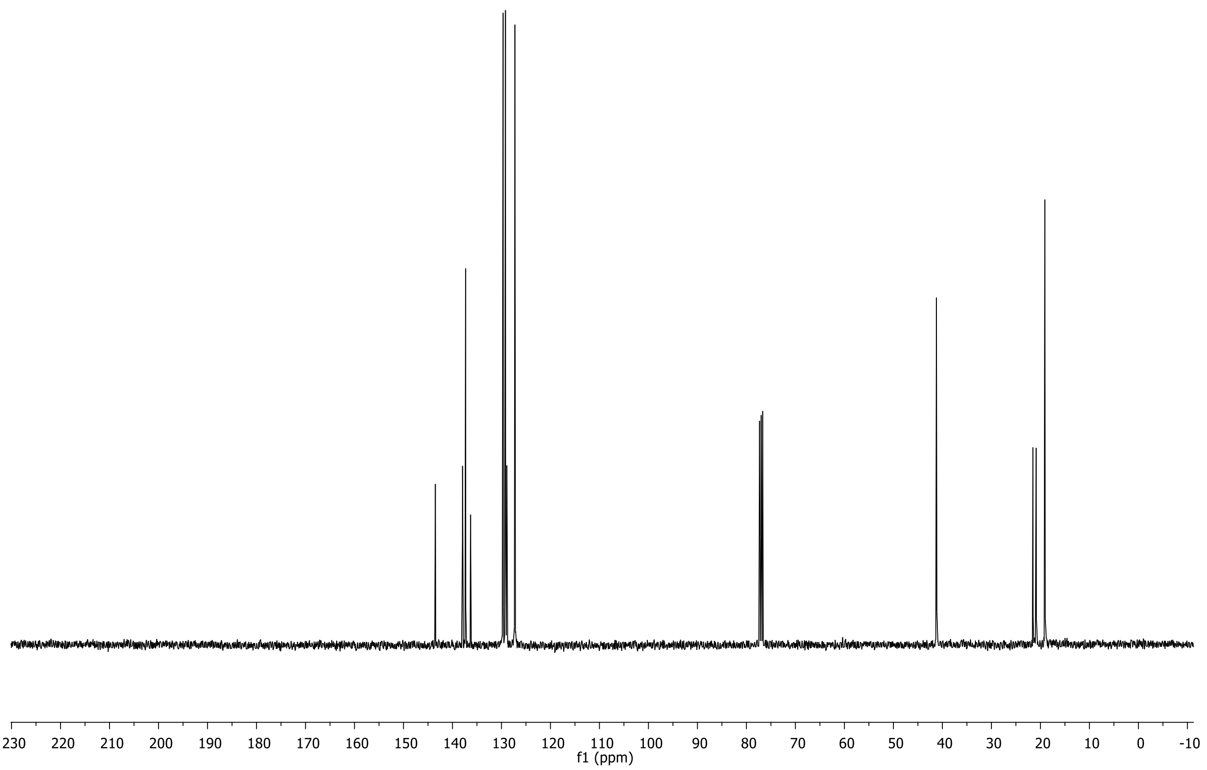
230 220 210 200 190 180 170 160 150 140 130 120 110 100 90 80 70 60 50 40 30 20 10 0 -10
f1 (ppm)

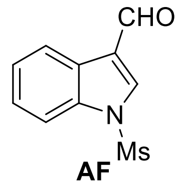
CHO
N
Ms

AF

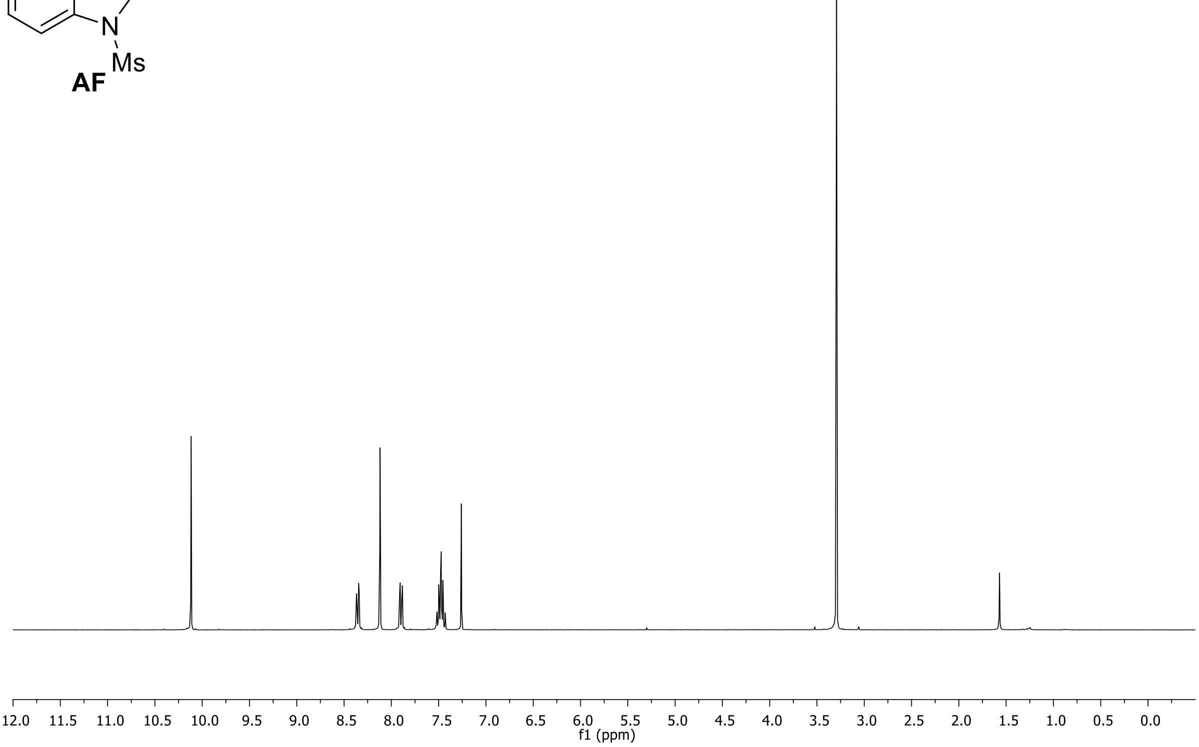
12.0 11.5 11.0 10.5 10.0 9.5 9.0 8.5 8.0 7.5 7.0 6.5 6.0 5.5 5.0 4.5 4.0 3.5 3.0 2.5 2.0 1.5 1.0 0.5 0.0
f1 (ppm)

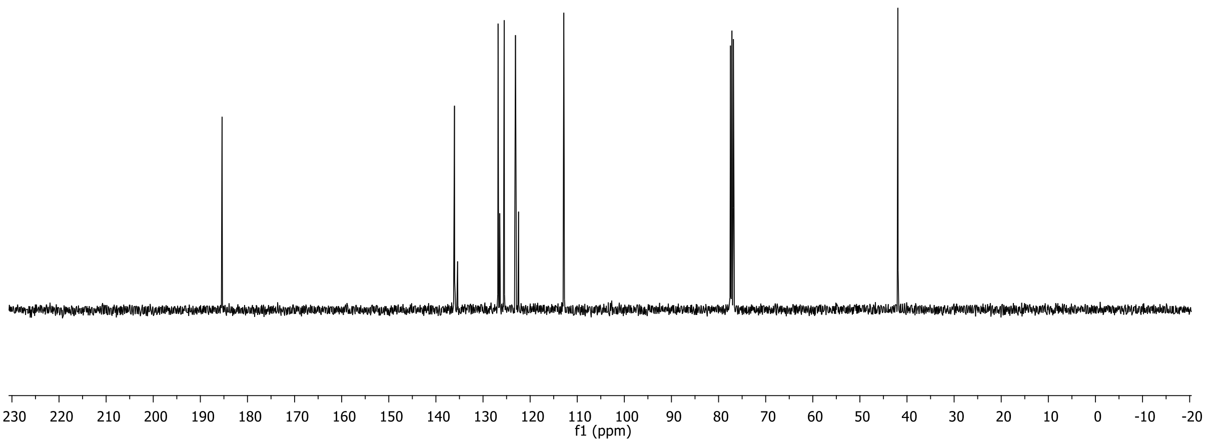
230 220 210 200 190 180 170 160 150 140 130 120 110 100 90 80 70 60 50 40 30 20 10 0 -10 -20
f1 (ppm)

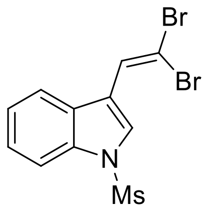
Br
Br
N
Ms

AG

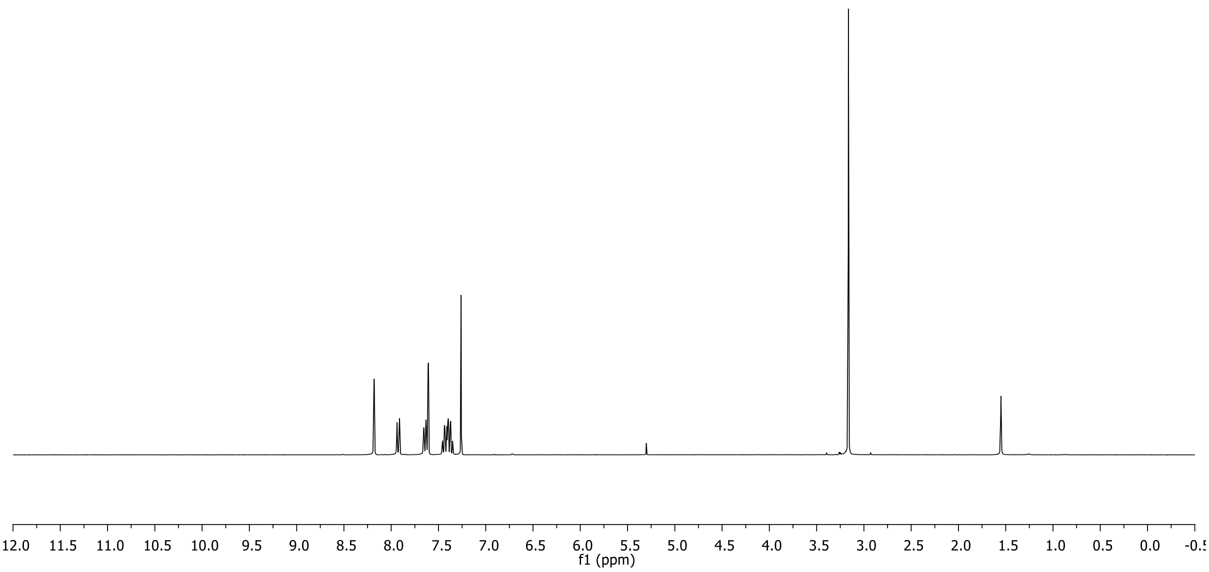
12.0 11.5 11.0 10.5 10.0 9.5 9.0 8.5 8.0 7.5 7.0 6.5 6.0 5.5 5.0 4.5 4.0 3.5 3.0 2.5 2.0 1.5 1.0 0.5 0.0
f1 (ppm)

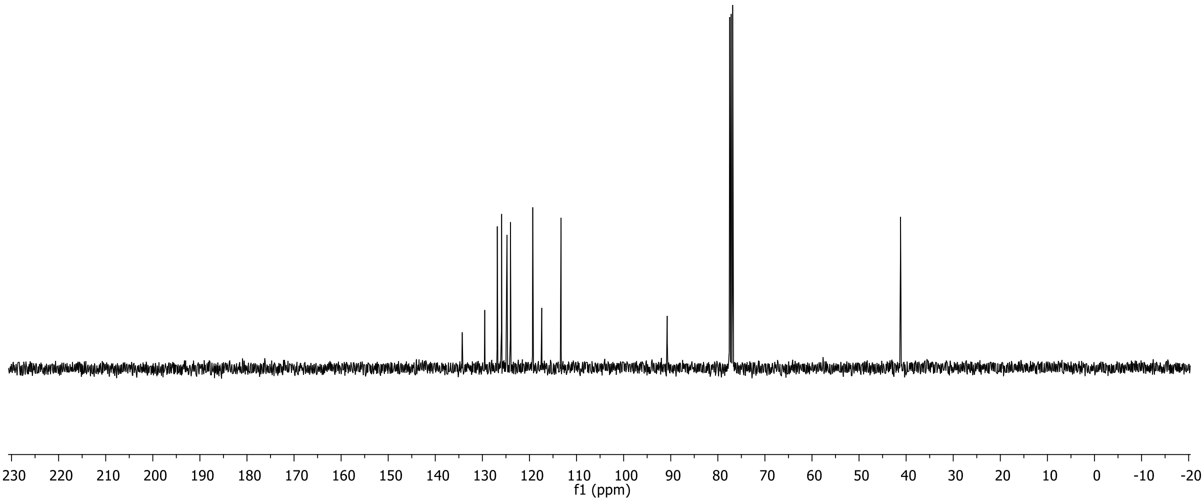
230 220 210 200 190 180 170 160 150 140 130 120 110 100 90 80 70 60 50 40 30 20 10 0 -10 -20
f1 (ppm)

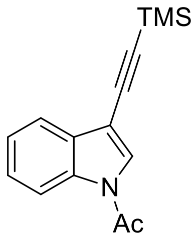
TMS
N
Ac

AH

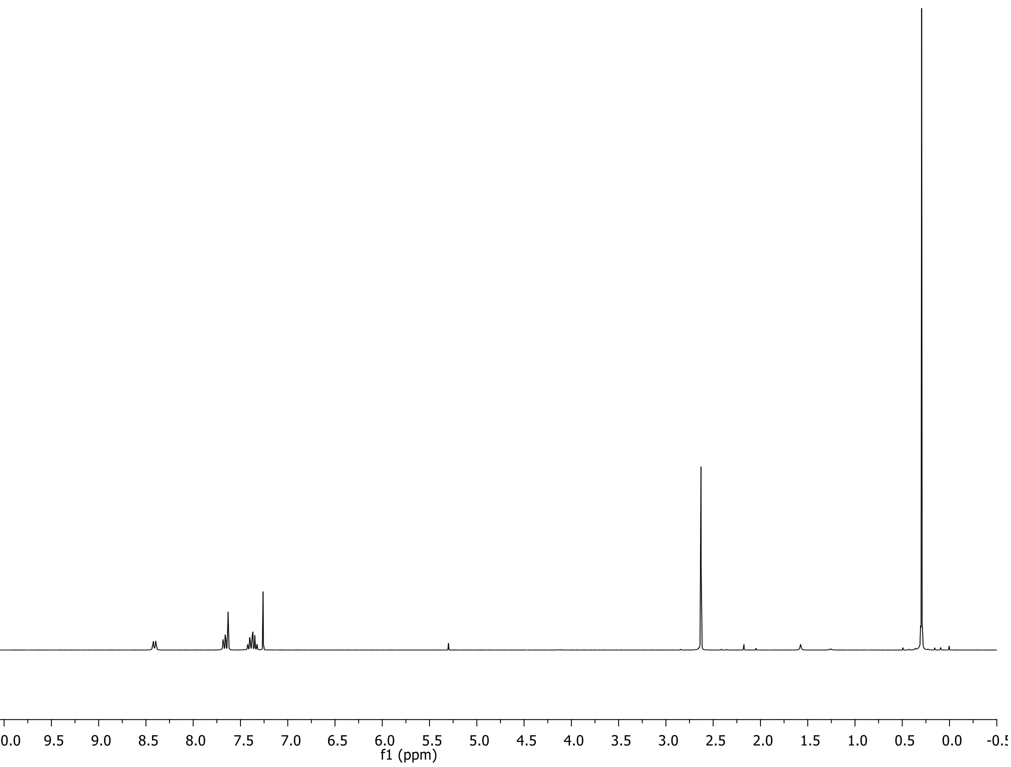
12.0 11.5 11.0 10.5 10.0 9.5 9.0 8.5 8.0 7.5 7.0 6.5 6.0 5.5 5.0 4.5 4.0 3.5 3.0 2.5 2.0 1.5 1.0 0.5 0.0
f1 (ppm)

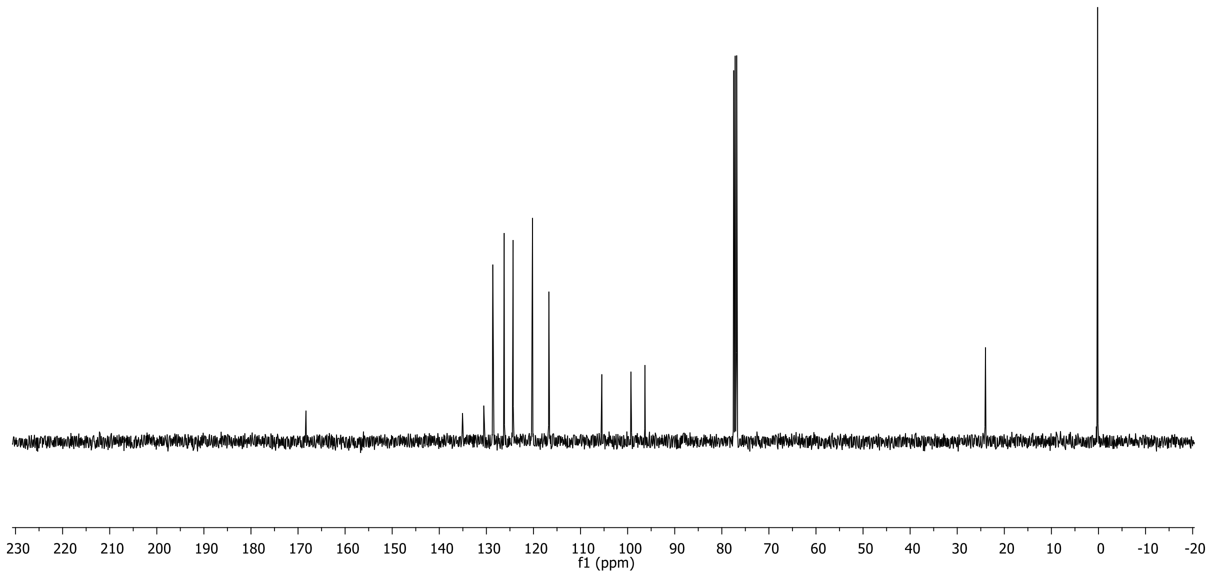
230 220 210 200 190 180 170 160 150 140 130 120 110 100 90 80 70 60 50 40 30 20 10 0 -10 -20
f1 (ppm)

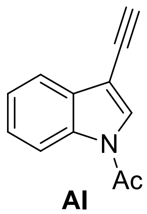
N
Ac

AI

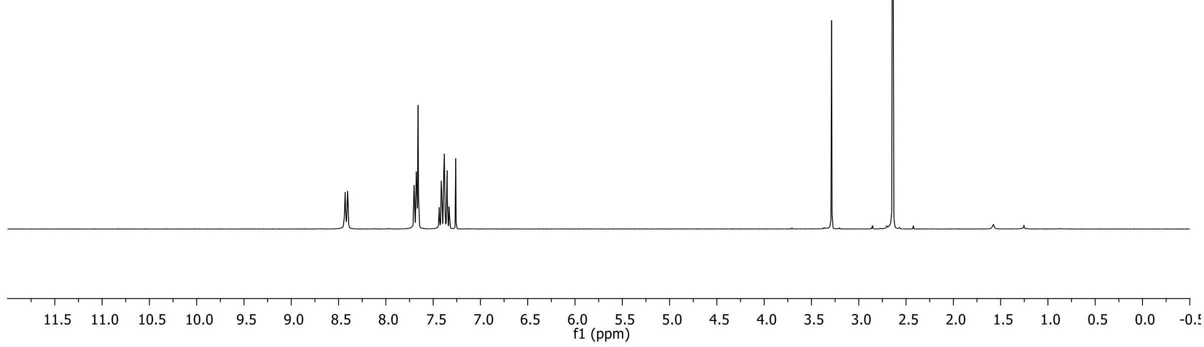
11.5 11.0 10.5 10.0 9.5 9.0 8.5 8.0 7.5 7.0 6.5 6.0 5.5 5.0 4.5 4.0 3.5 3.0 2.5 2.0 1.5 1.0 0.5 0.0 -0.
f1 (ppm)

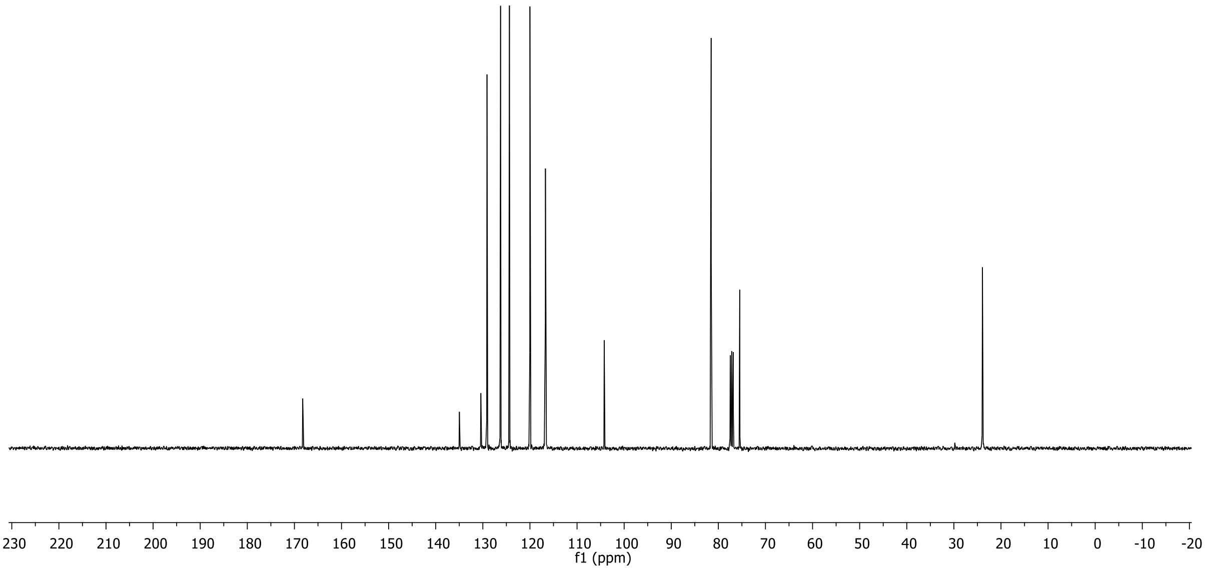
230 220 210 200 190 180 170 160 150 140 130 120 110 100 90 80 70 60 50 40 30 20 10 0 -10 -20
f1 (ppm)

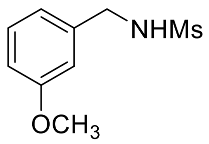
NHMs
OCH$_3$

AN

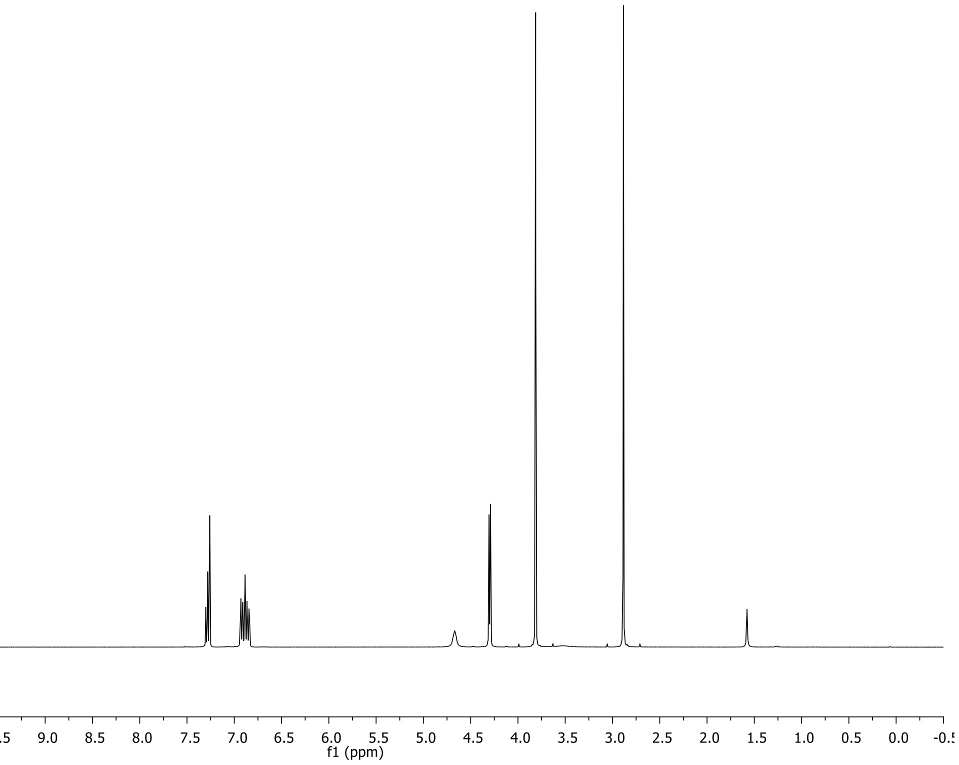
12.0 11.5 11.0 10.5 10.0 9.5 9.0 8.5 8.0 7.5 7.0 6.5 6.0 5.5 5.0 4.5 4.0 3.5 3.0 2.5 2.0 1.5 1.0 0.5 0.0 -0.[illegible]
f1 (ppm)

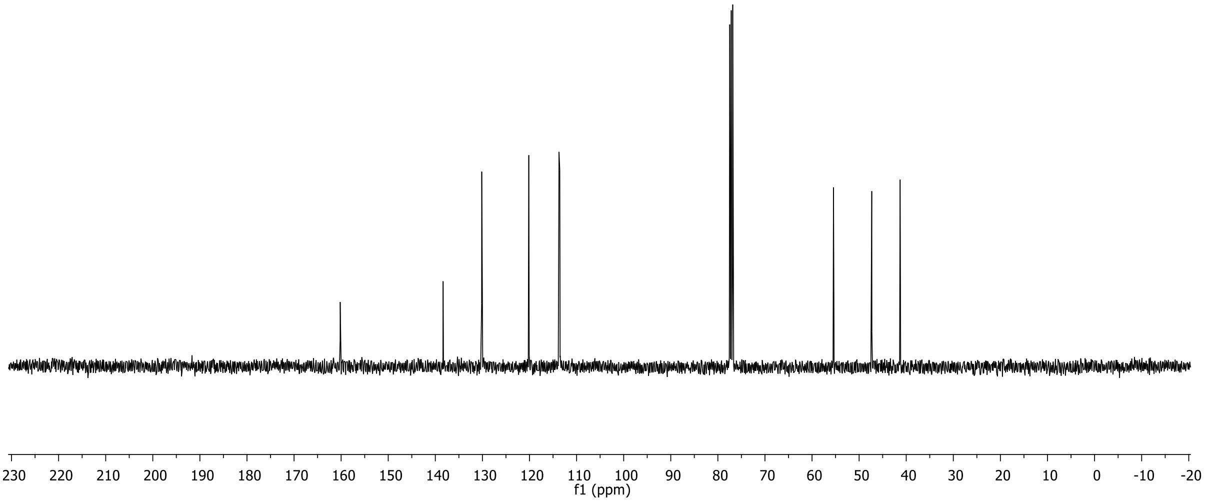
230 220 210 200 190 180 170 160 150 140 130 120 110 100 90 80 70 60 50 40 30 20 10 0 -10 -20
f1 (ppm)

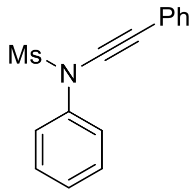
Ph
Ms
N

1a

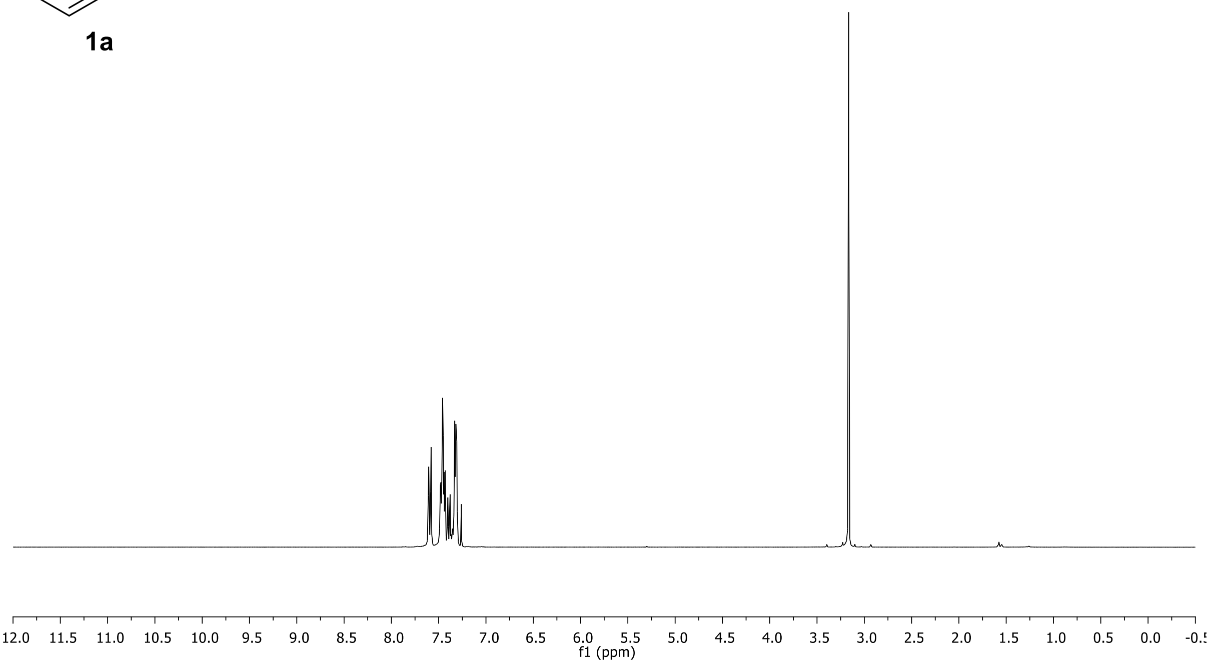
12.0 11.5 11.0 10.5 10.0 9.5 9.0 8.5 8.0 7.5 7.0 6.5 6.0 5.5 5.0 4.5 4.0 3.5 3.0 2.5 2.0 1.5 1.0 0.5 0.0
f1 (ppm)

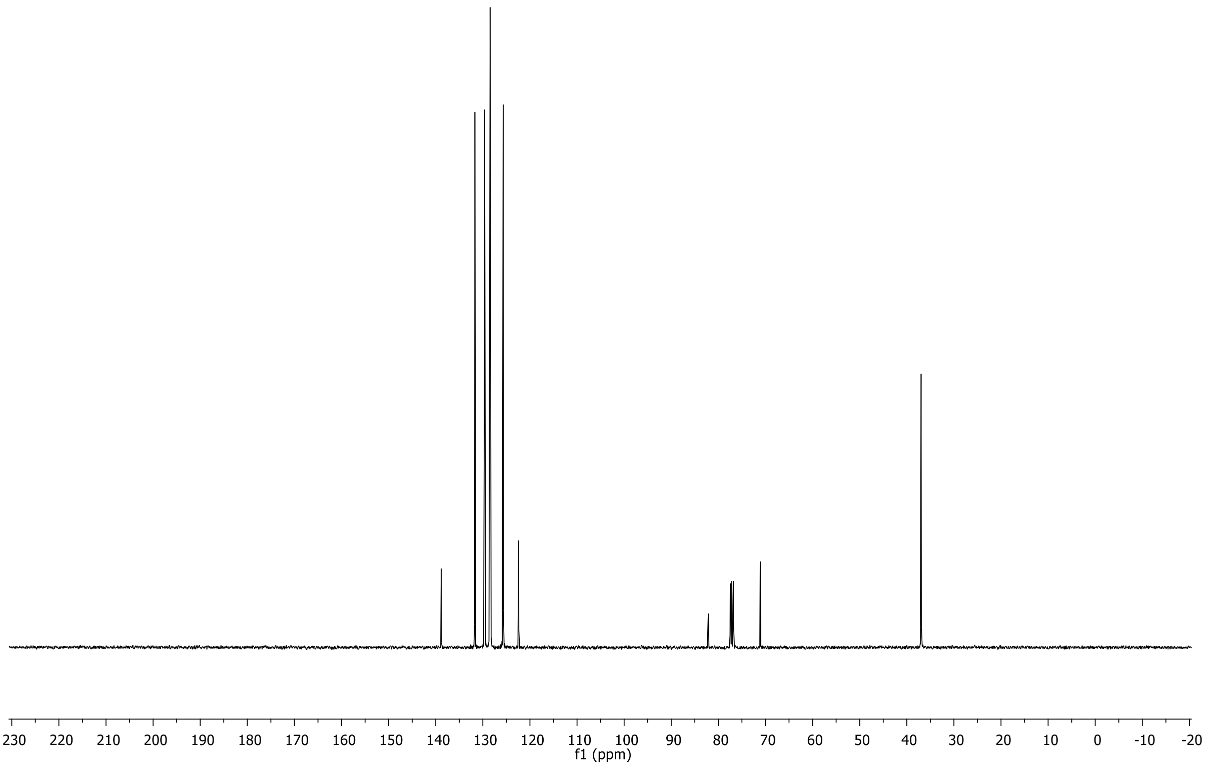
230 220 210 200 190 180 170 160 150 140 130 120 110 100 90 80 70 60 50 40 30 20 10 0 -10 -20
f1 (ppm)

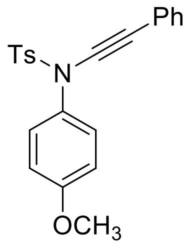
Ph
Ts
N
OCH$_3$

1b

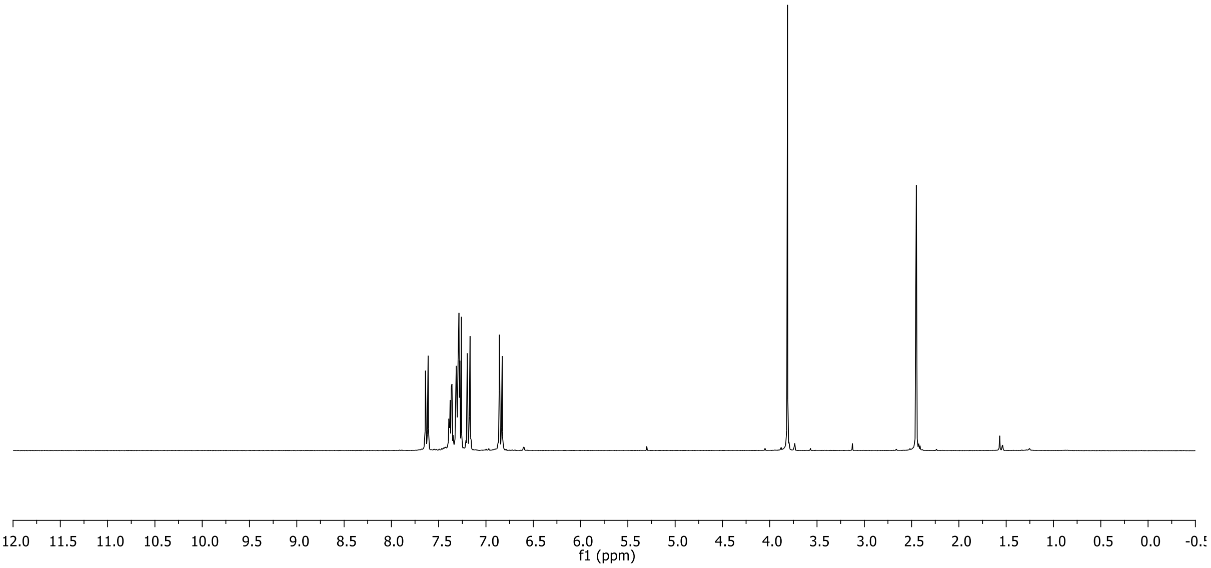
12.0 11.5 11.0 10.5 10.0 9.5 9.0 8.5 8.0 7.5 7.0 6.5 6.0 5.5 5.0 4.5 4.0 3.5 3.0 2.5 2.0 1.5 1.0 0.5 0.0
f1 (ppm)

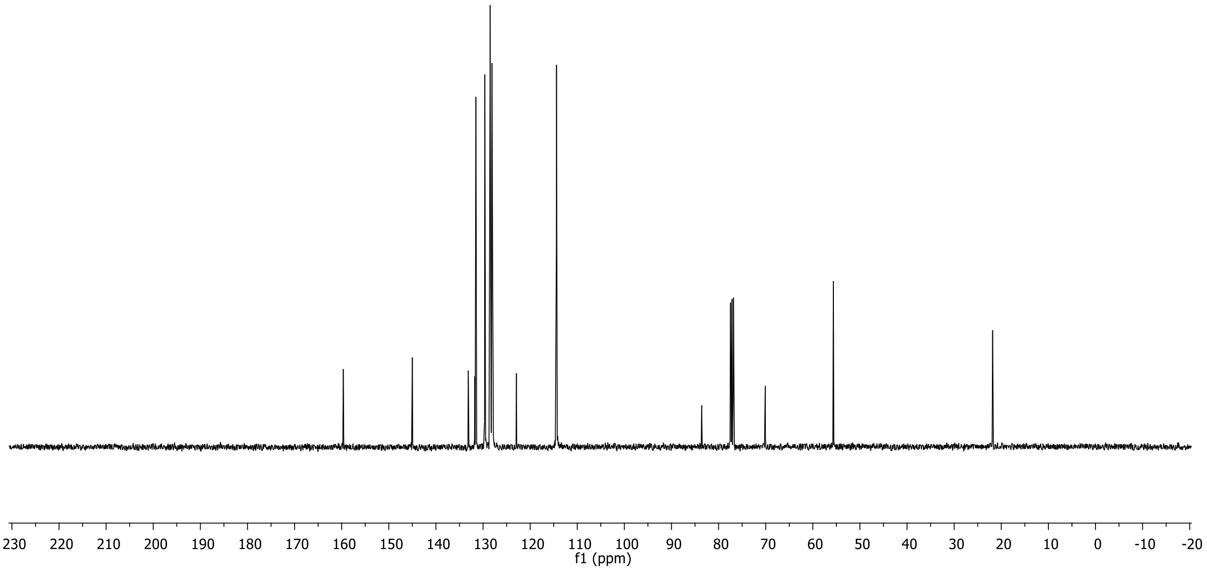
230 220 210 200 190 180 170 160 150 140 130 120 110 100 90 80 70 60 50 40 30 20 10 0 -10 -20
f1 (ppm)

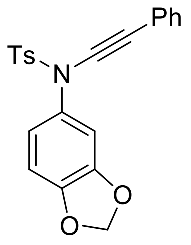
Ph
Ts
N
O
O

1c

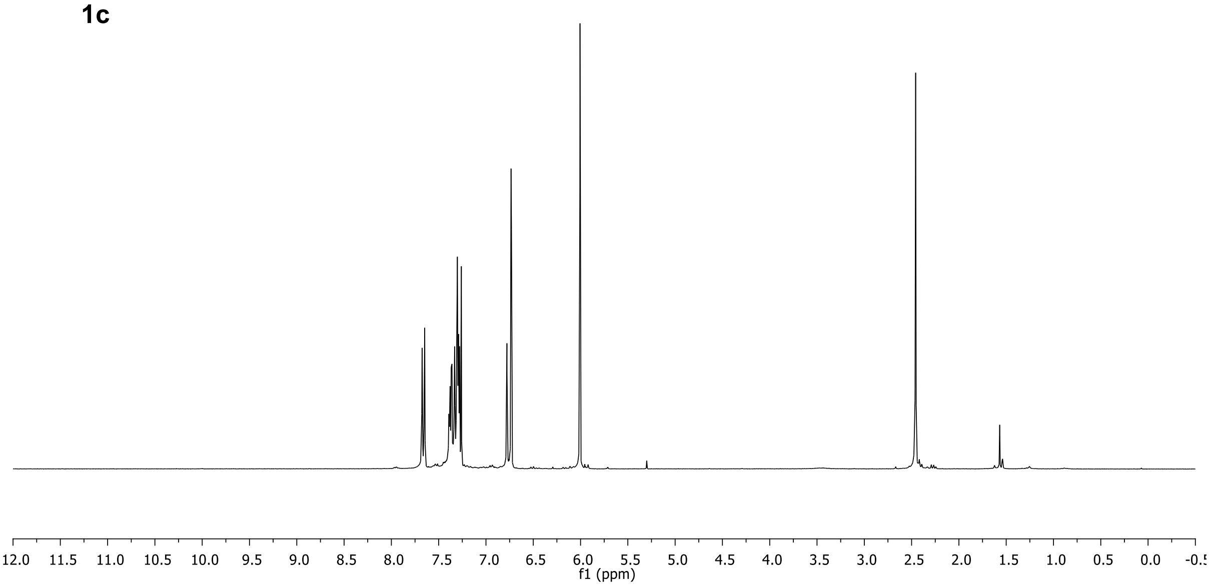
12.0 11.5 11.0 10.5 10.0 9.5 9.0 8.5 8.0 7.5 7.0 6.5 6.0 5.5 5.0 4.5 4.0 3.5 3.0 2.5 2.0 1.5 1.0 0.5 0.0
f1 (ppm)

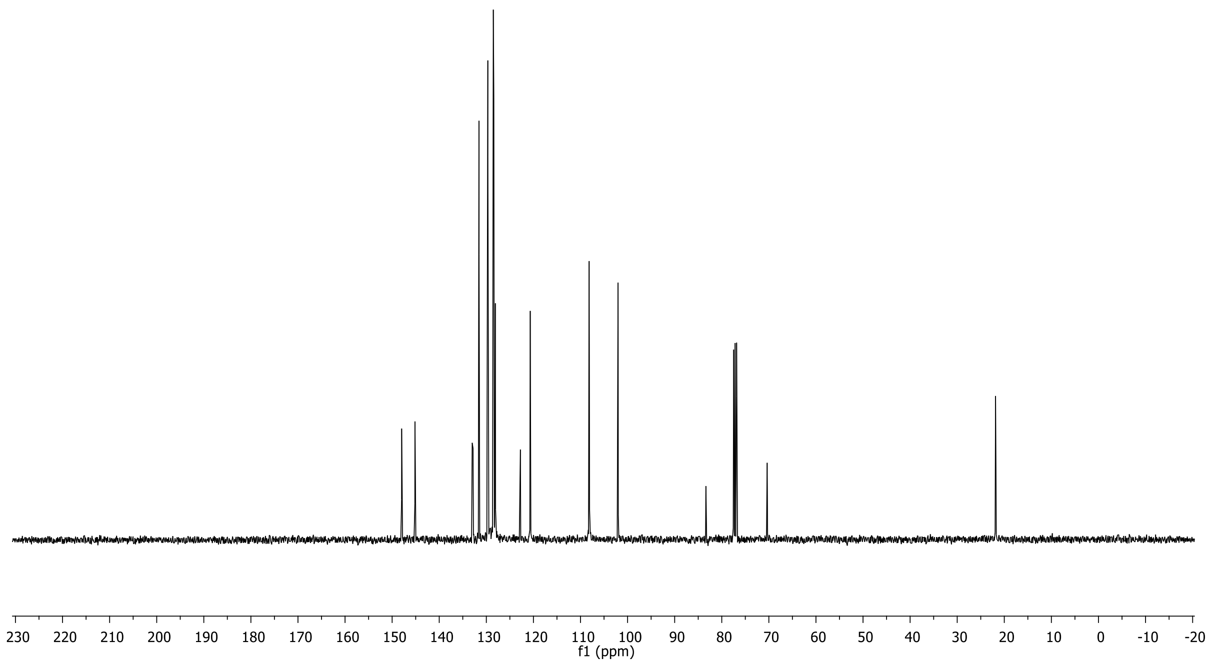
230 220 210 200 190 180 170 160 150 140 130 120 110 100 90 80 70 60 50 40 30 20 10 0 -10 -20
f1 (ppm)

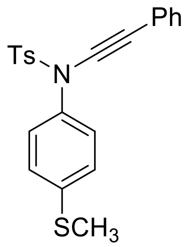
Ph
Ts
N
SCH3

1d

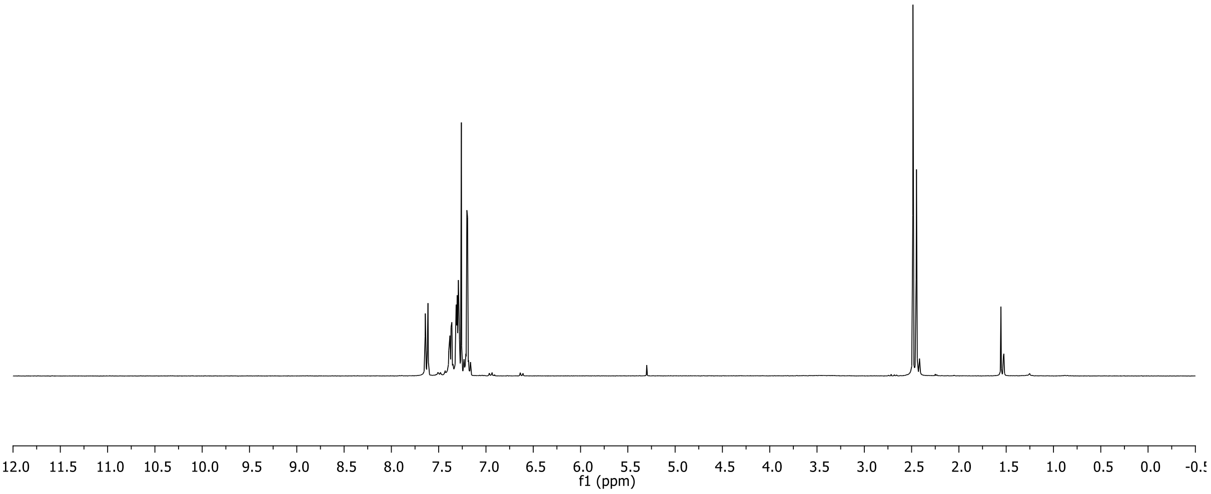
12.0 11.5 11.0 10.5 10.0 9.5 9.0 8.5 8.0 7.5 7.0 6.5 6.0 5.5 5.0 4.5 4.0 3.5 3.0 2.5 2.0 1.5 1.0 0.5 0.0
f1 (ppm)

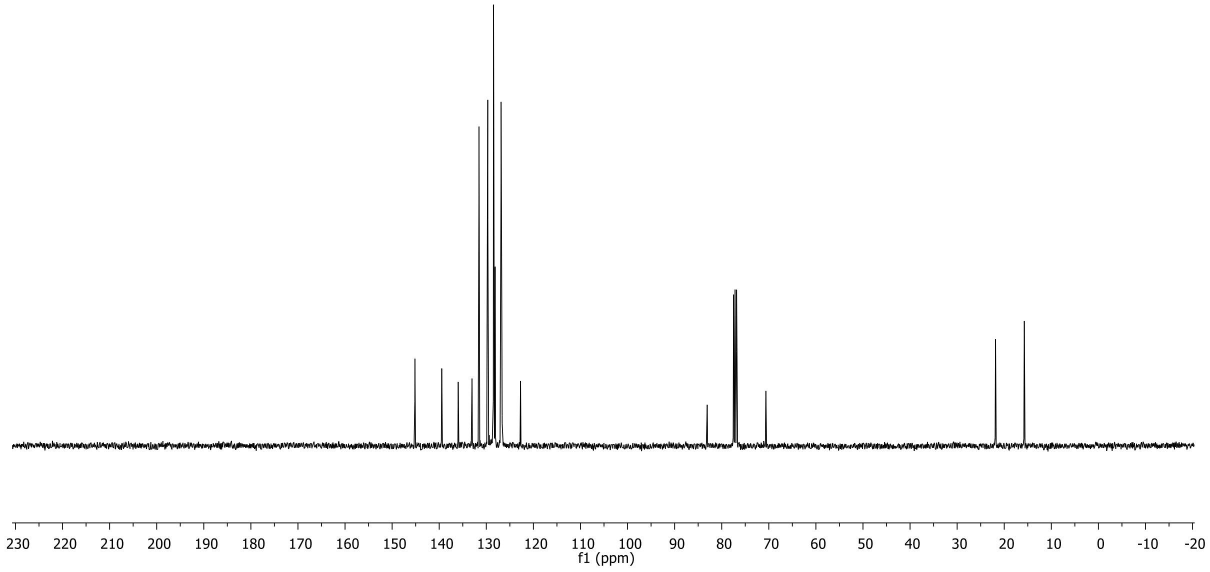
230 220 210 200 190 180 170 160 150 140 130 120 110 100 90 80 70 60 50 40 30 20 10 0 -10 -20
f1 (ppm)

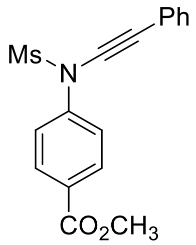
Ph
Ms
N
CO2CH3

1e

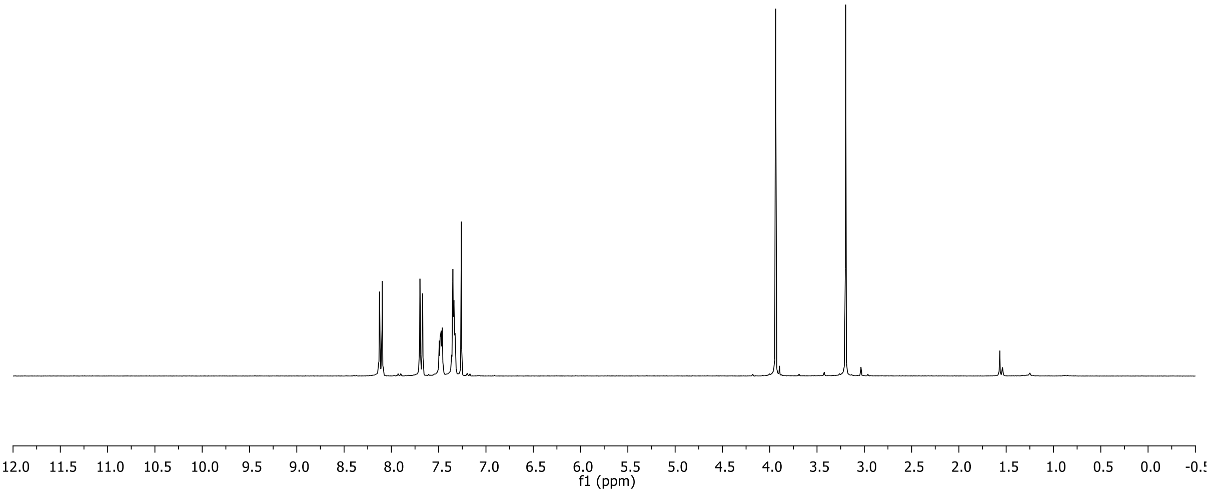
12.0 11.5 11.0 10.5 10.0 9.5 9.0 8.5 8.0 7.5 7.0 6.5 6.0 5.5 5.0 4.5 4.0 3.5 3.0 2.5 2.0 1.5 1.0 0.5 0.0
f1 (ppm)

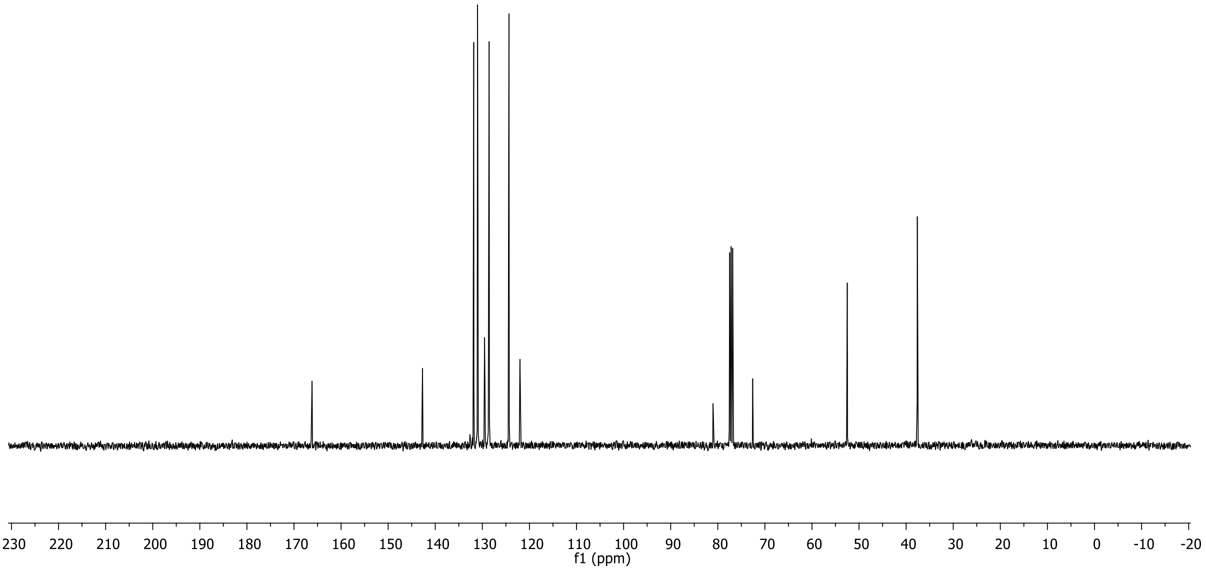
230 220 210 200 190 180 170 160 150 140 130 120 110 100 90 80 70 60 50 40 30 20 10 0 -10 -20
f1 (ppm)

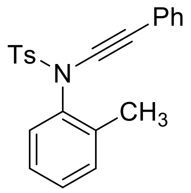

1f

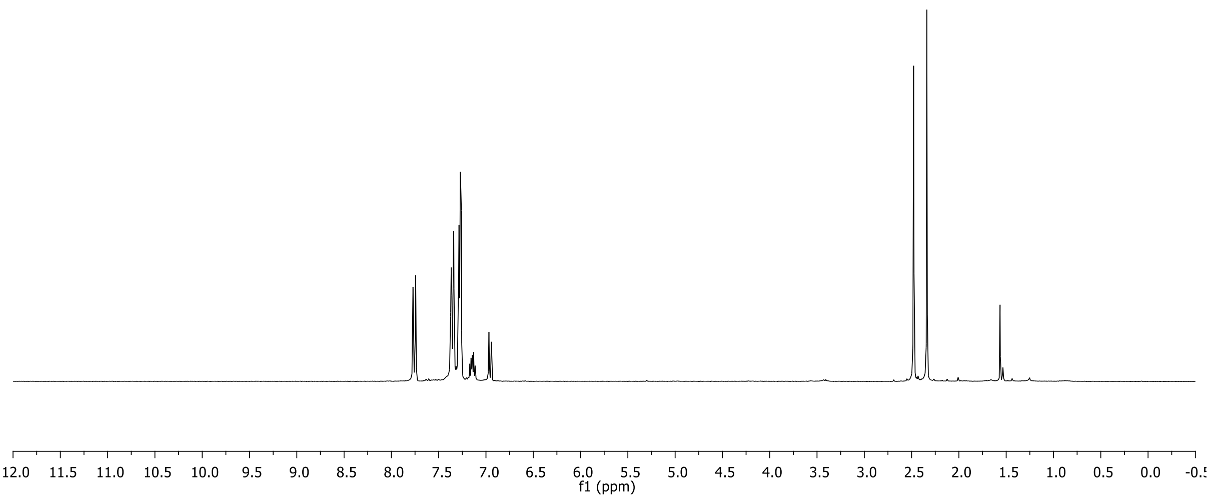
12.0 11.5 11.0 10.5 10.0 9.5 9.0 8.5 8.0 7.5 7.0 6.5 6.0 5.5 5.0 4.5 4.0 3.5 3.0 2.5 2.0 1.5 1.0 0.5 0.0
f1 (ppm)

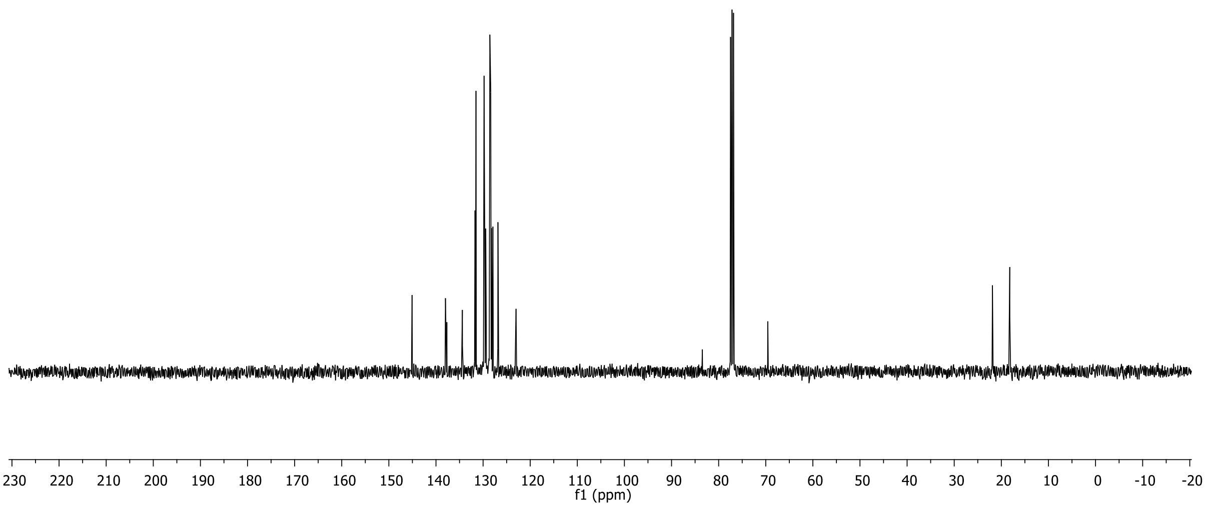
230 220 210 200 190 180 170 160 150 140 130 120 110 100 90 80 70 60 50 40 30 20 10 0 -10 -20
f1 (ppm)

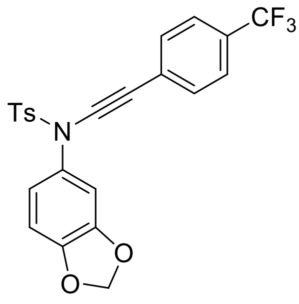

CF3
Ts
N
O
O

1g

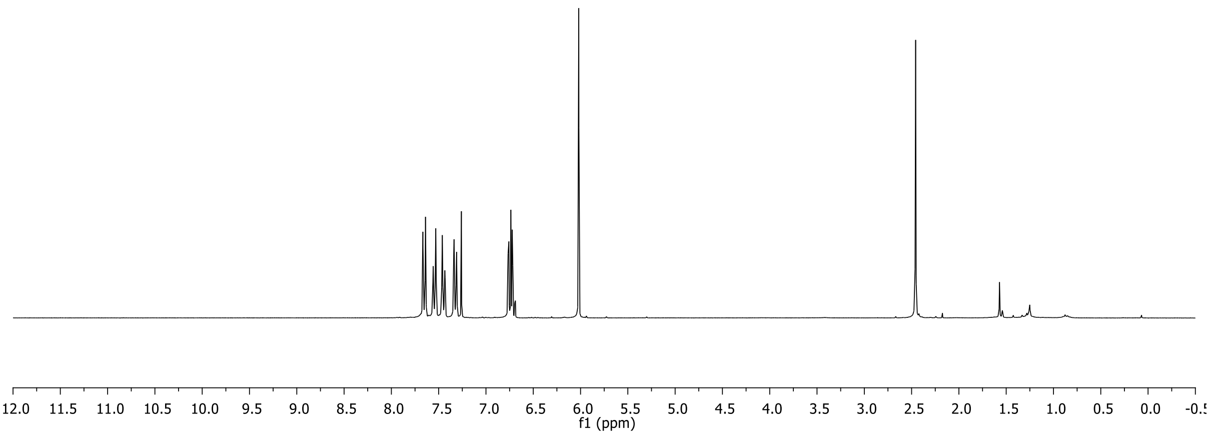

12.0 11.5 11.0 10.5 10.0 9.5 9.0 8.5 8.0 7.5 7.0 6.5 6.0 5.5 5.0 4.5 4.0 3.5 3.0 2.5 2.0 1.5 1.0 0.5 0.0
f1 (ppm)

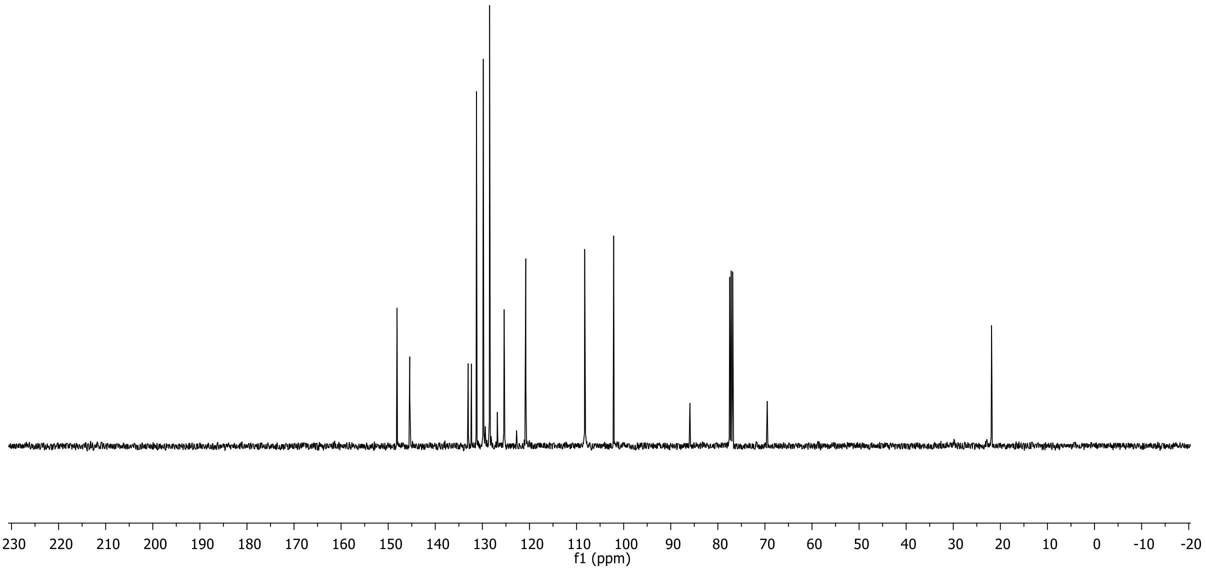

230 220 210 200 190 180 170 160 150 140 130 120 110 100 90 80 70 60 50 40 30 20 10 0 -10 -20
f1 (ppm)

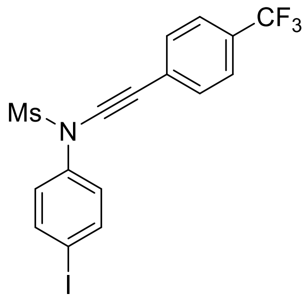
CF3
Ms
N
I

1h

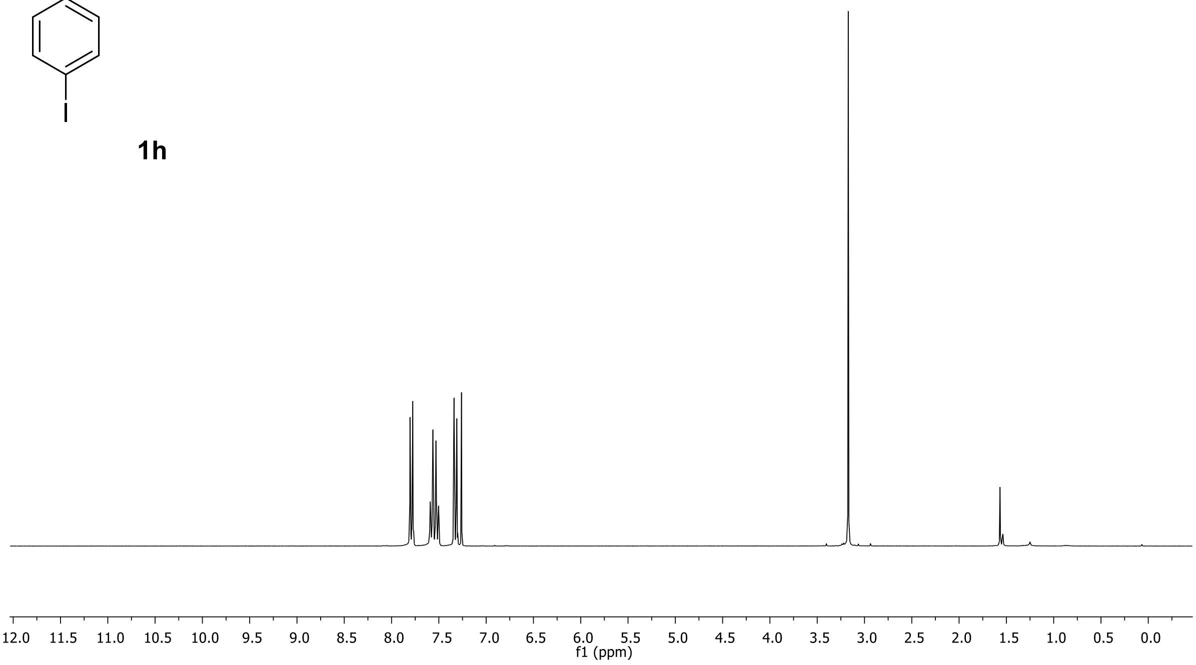
12.0 11.5 11.0 10.5 10.0 9.5 9.0 8.5 8.0 7.5 7.0 6.5 6.0 5.5 5.0 4.5 4.0 3.5 3.0 2.5 2.0 1.5 1.0 0.5 0.0
f1 (ppm)

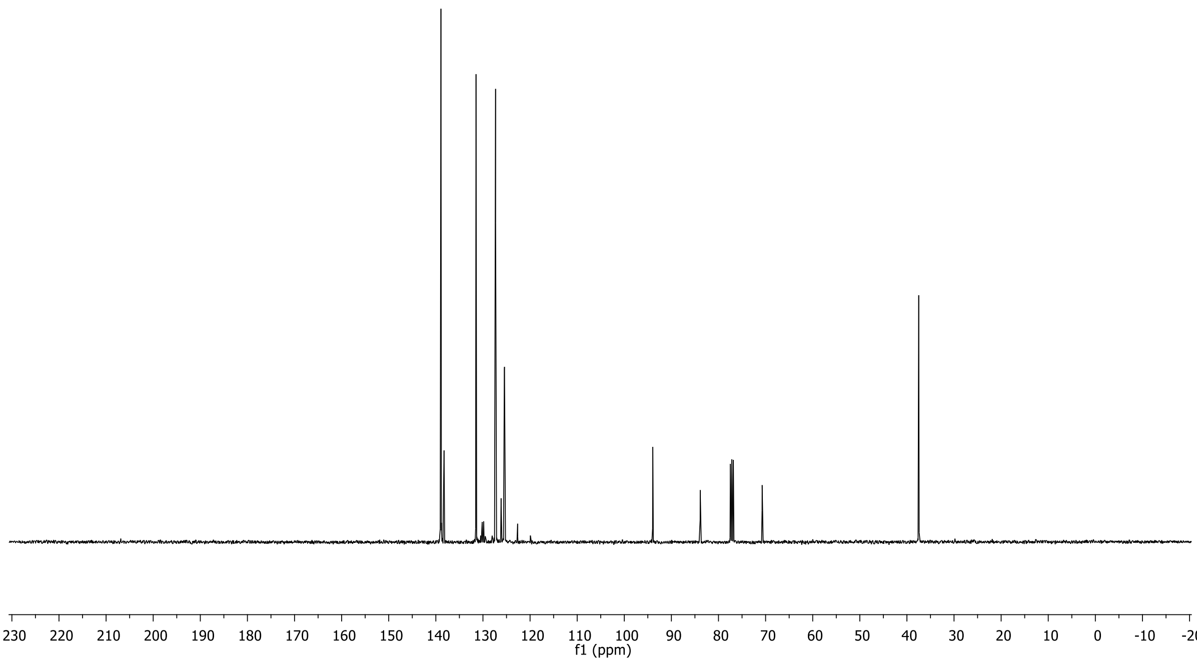
230 220 210 200 190 180 170 160 150 140 130 120 110 100 90 80 70 60 50 40 30 20 10 0 -10 -20
f1 (ppm)

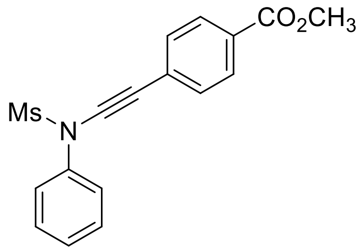

1i

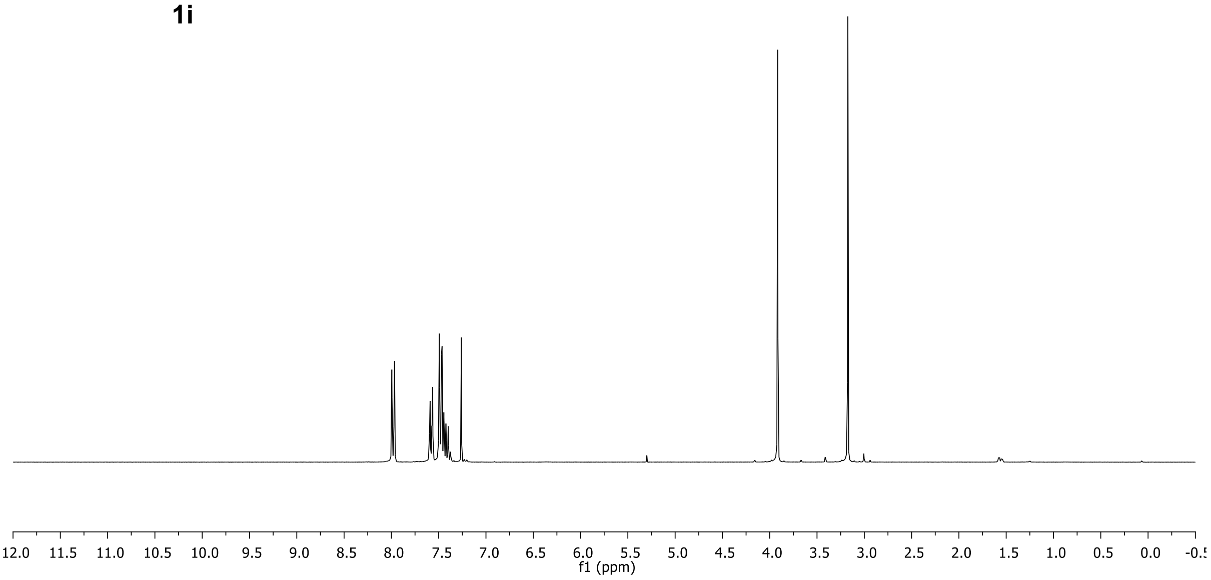

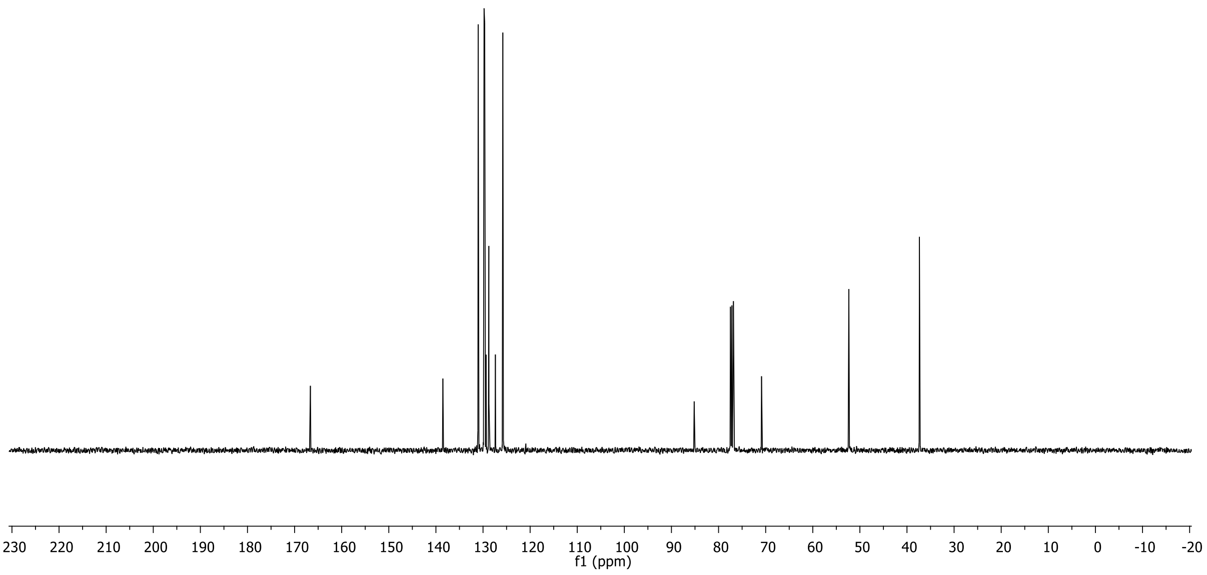

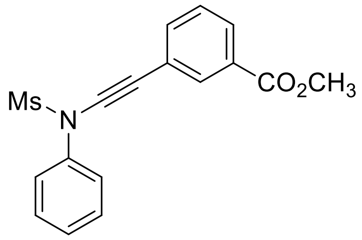
Ms
N
CO2CH3

1j

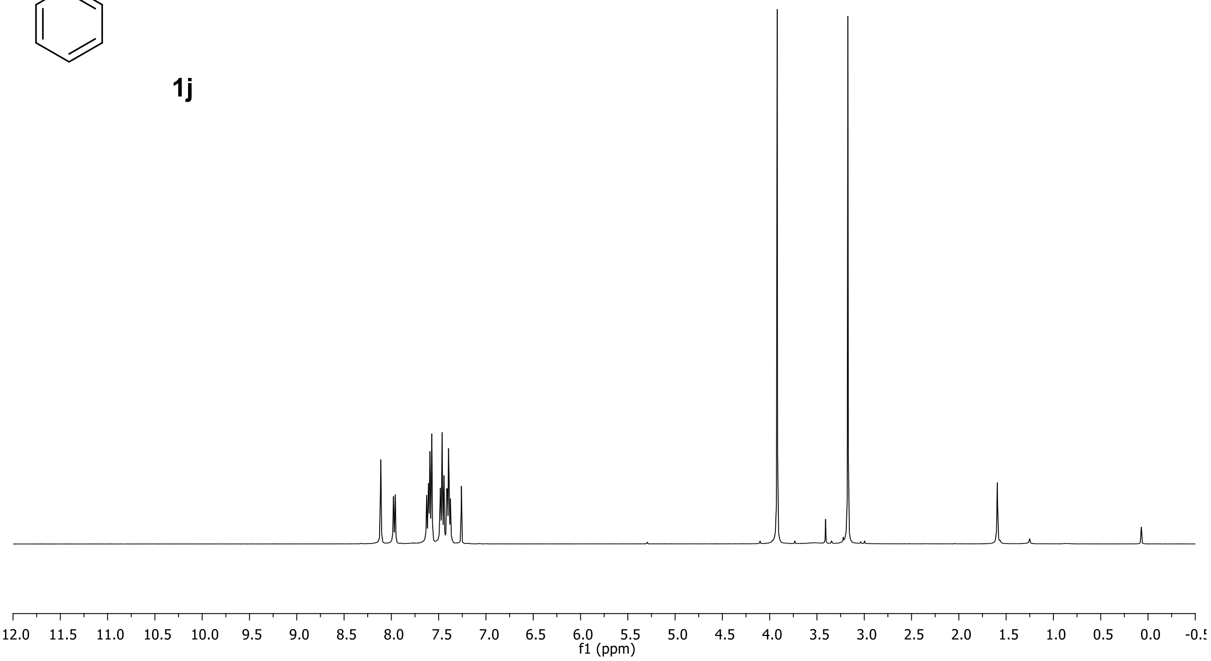
12.0 11.5 11.0 10.5 10.0 9.5 9.0 8.5 8.0 7.5 7.0 6.5 6.0 5.5 5.0 4.5 4.0 3.5 3.0 2.5 2.0 1.5 1.0 0.5 0.0 -0.5
f1 (ppm)

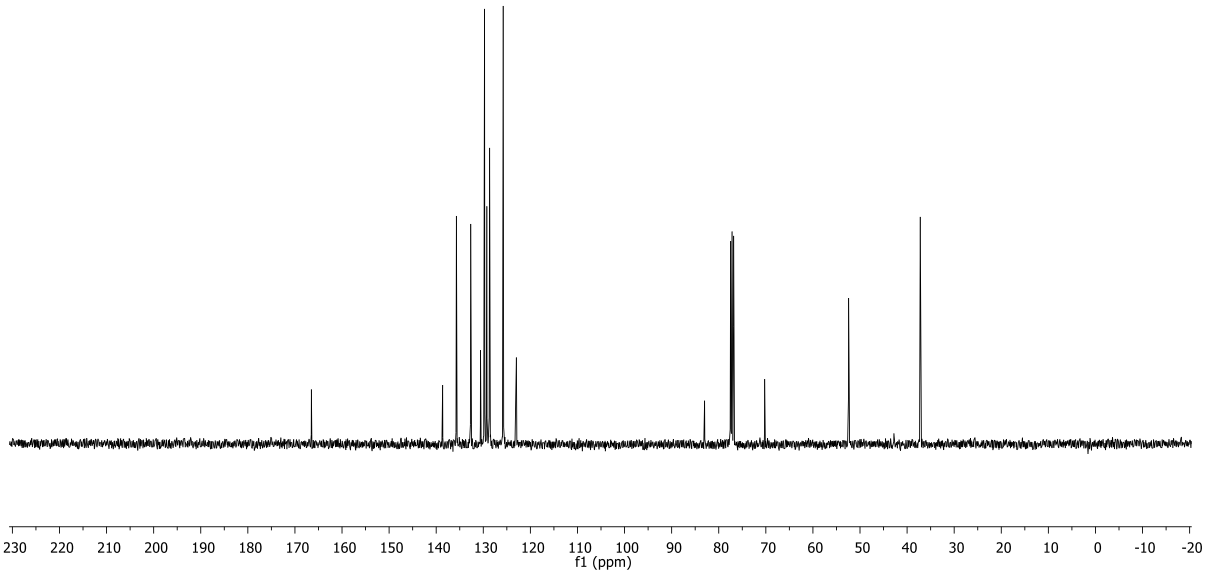
230 220 210 200 190 180 170 160 150 140 130 120 110 100 90 80 70 60 50 40 30 20 10 0 -10 -20
f1 (ppm)

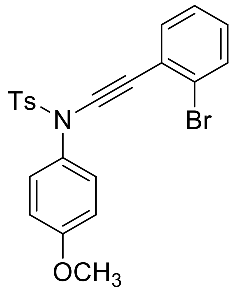
Ts
N
Br
OCH3

1k

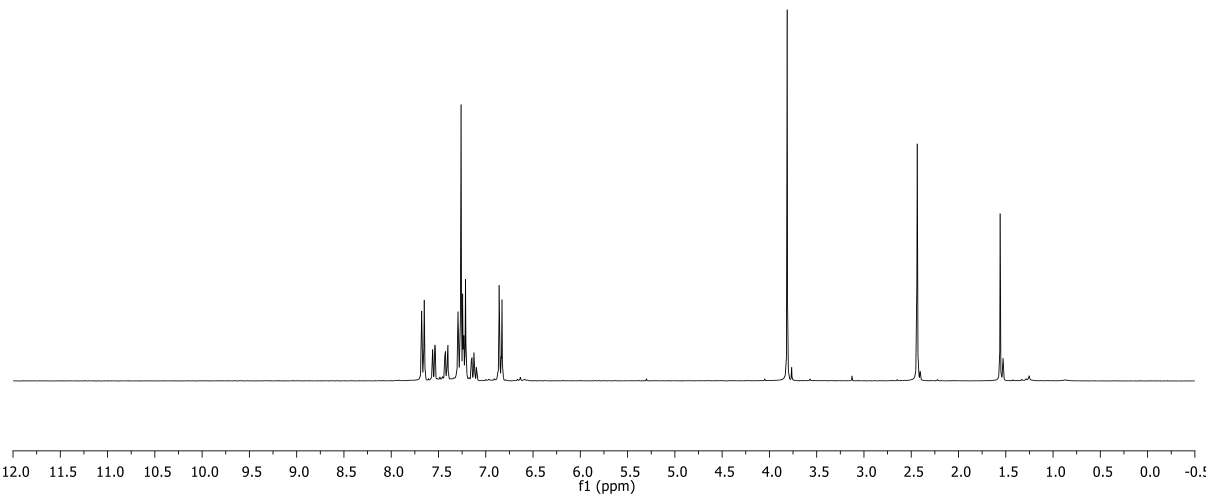
12.0 11.5 11.0 10.5 10.0 9.5 9.0 8.5 8.0 7.5 7.0 6.5 6.0 5.5 5.0 4.5 4.0 3.5 3.0 2.5 2.0 1.5 1.0 0.5 0.0
f1 (ppm)

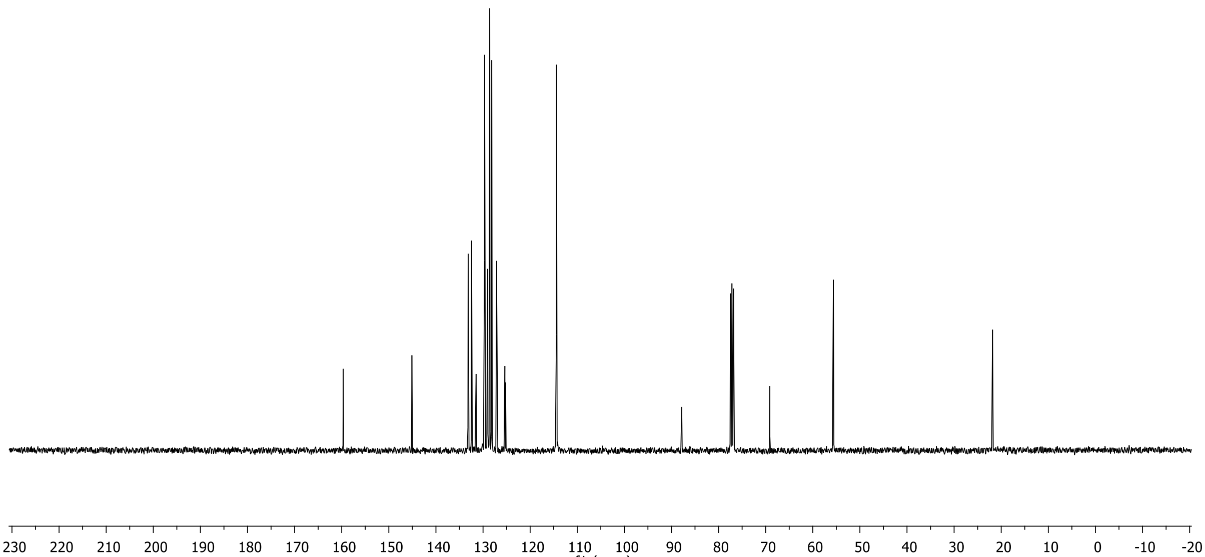
230 220 210 200 190 180 170 160 150 140 130 120 110 100 90 80 70 60 50 40 30 20 10 0 -10 -20
f1 (ppm)

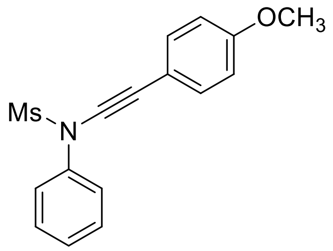
OCH3
Ms
N

1l

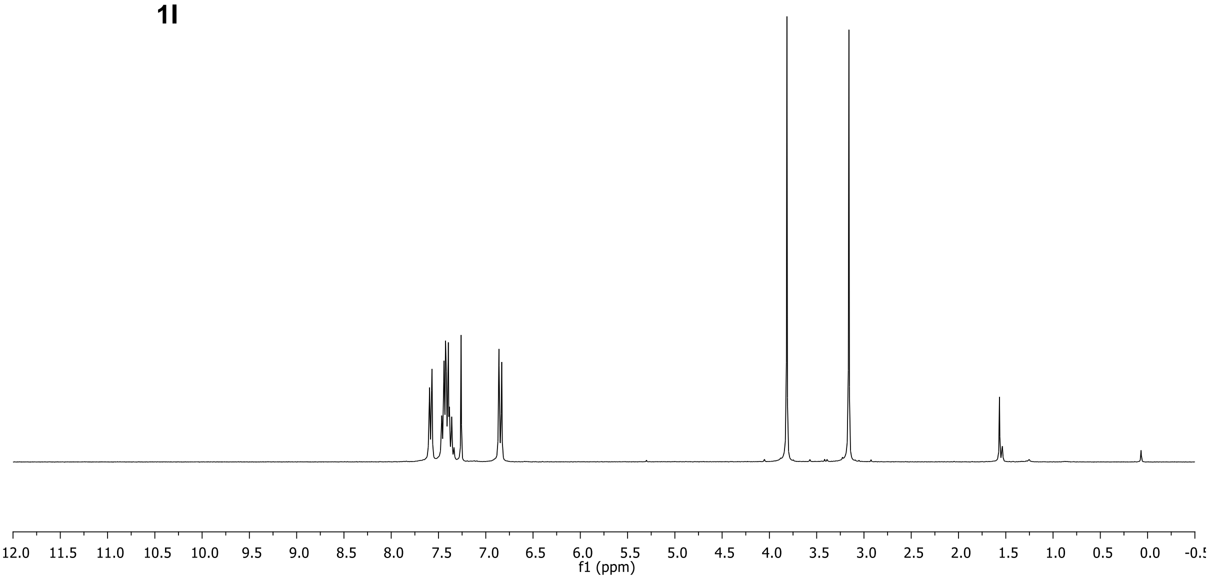
12.0 11.5 11.0 10.5 10.0 9.5 9.0 8.5 8.0 7.5 7.0 6.5 6.0 5.5 5.0 4.5 4.0 3.5 3.0 2.5 2.0 1.5 1.0 0.5 0.0
f1 (ppm)

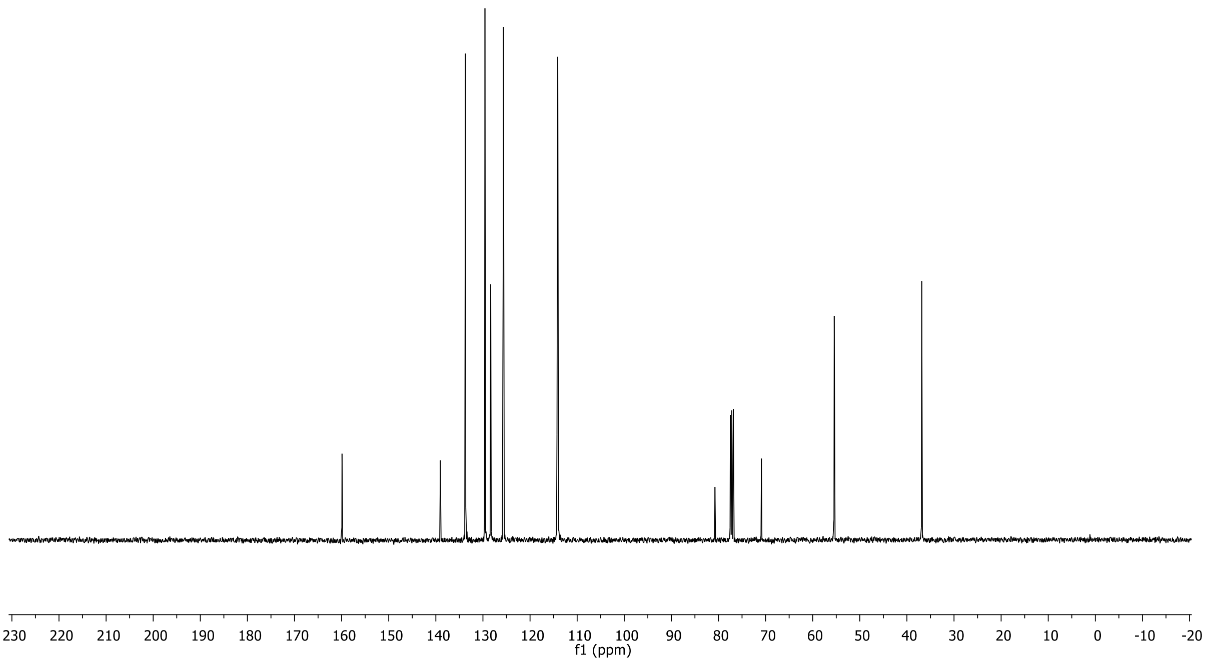
230 220 210 200 190 180 170 160 150 140 130 120 110 100 90 80 70 60 50 40 30 20 10 0 -10 -20
f1 (ppm)

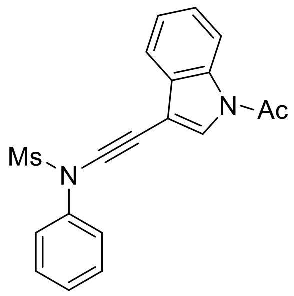
Ms
N
N
Ac

1m

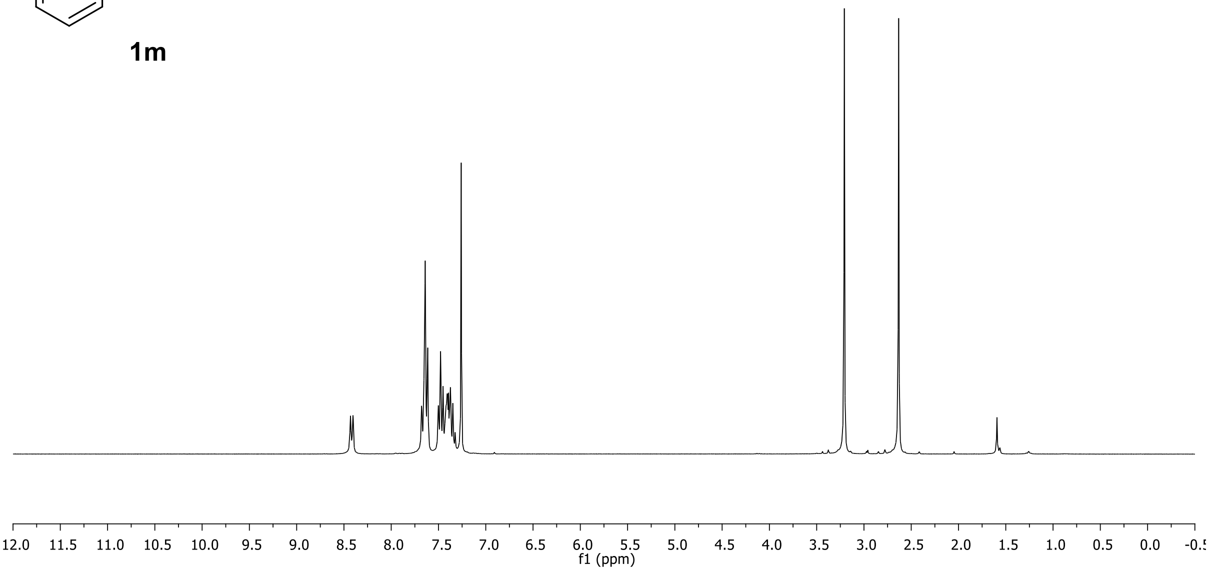
12.0 11.5 11.0 10.5 10.0 9.5 9.0 8.5 8.0 7.5 7.0 6.5 6.0 5.5 5.0 4.5 4.0 3.5 3.0 2.5 2.0 1.5 1.0 0.5 0.0 -0.5
f1 (ppm)

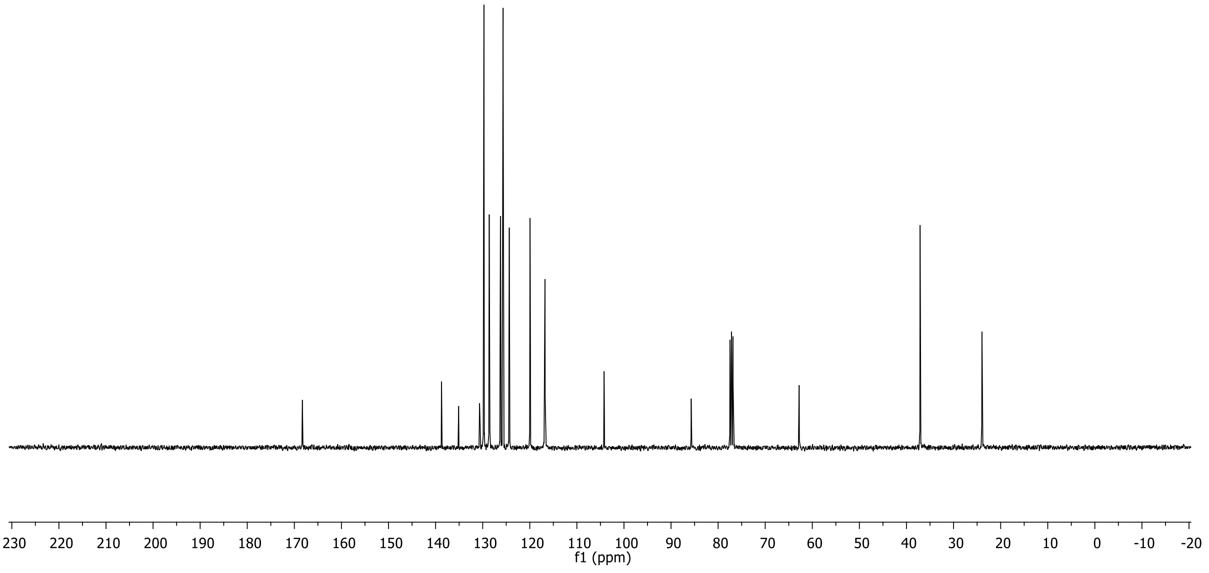
230 220 210 200 190 180 170 160 150 140 130 120 110 100 90 80 70 60 50 40 30 20 10 0 -10 -20
f1 (ppm)

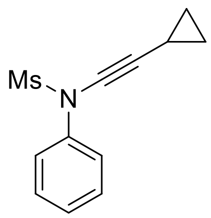
Ms
N

1n

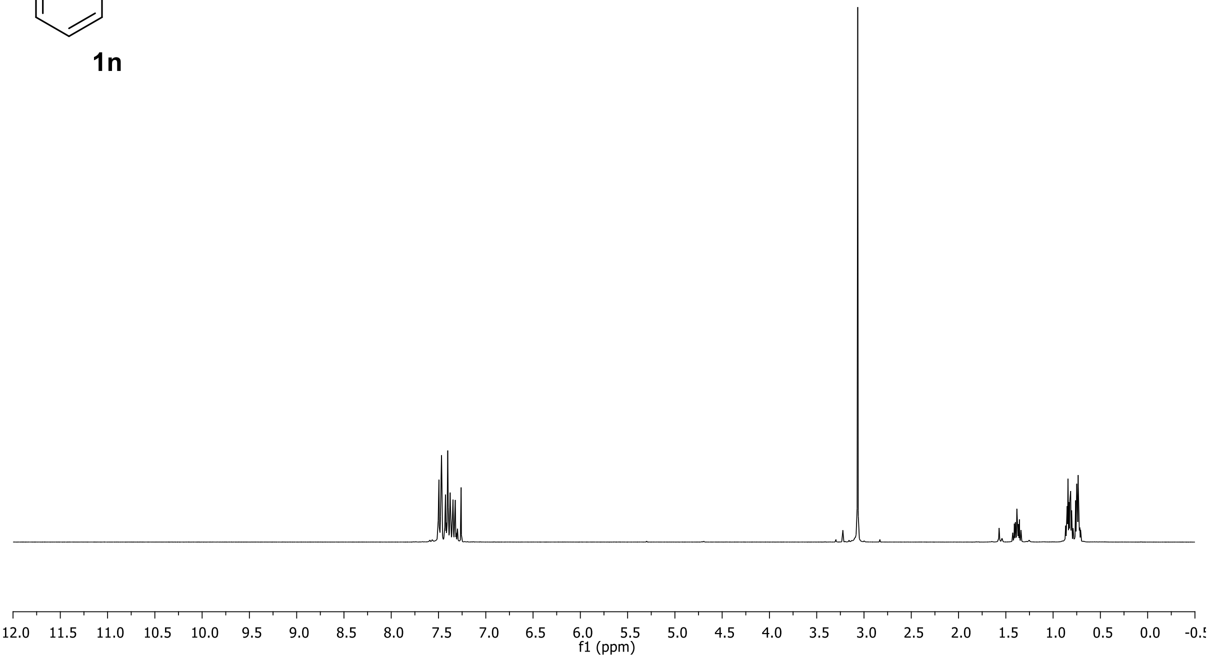
12.0 11.5 11.0 10.5 10.0 9.5 9.0 8.5 8.0 7.5 7.0 6.5 6.0 5.5 5.0 4.5 4.0 3.5 3.0 2.5 2.0 1.5 1.0 0.5 0.0
f1 (ppm)

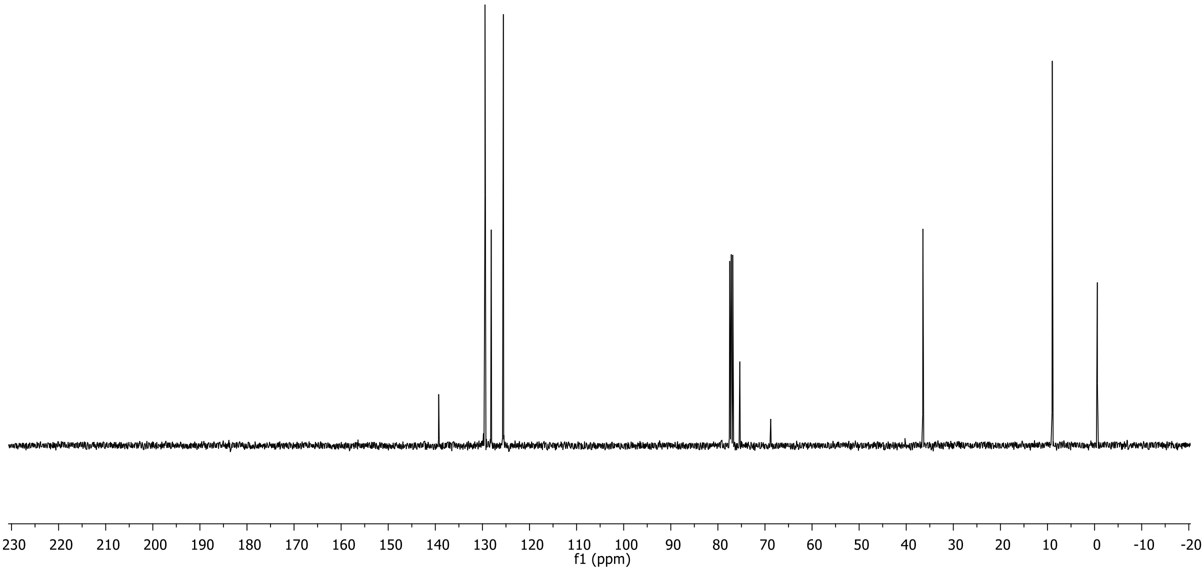
230 220 210 200 190 180 170 160 150 140 130 120 110 100 90 80 70 60 50 40 30 20 10 0 -10 -20
f1 (ppm)

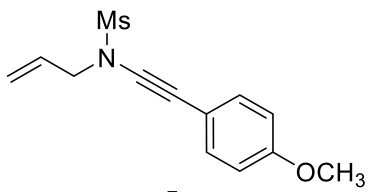
Ms
N
OCH3

5

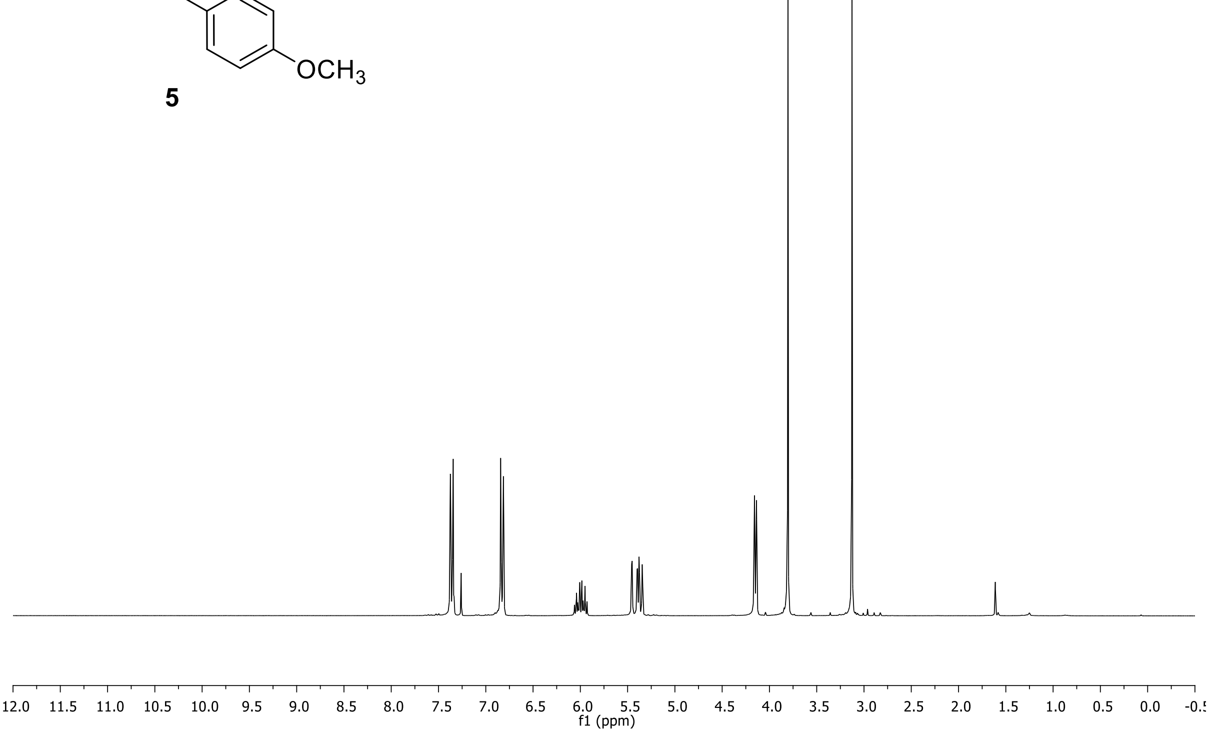
12.0 11.5 11.0 10.5 10.0 9.5 9.0 8.5 8.0 7.5 7.0 6.5 6.0 5.5 5.0 4.5 4.0 3.5 3.0 2.5 2.0 1.5 1.0 0.5 0.0
f1 (ppm)

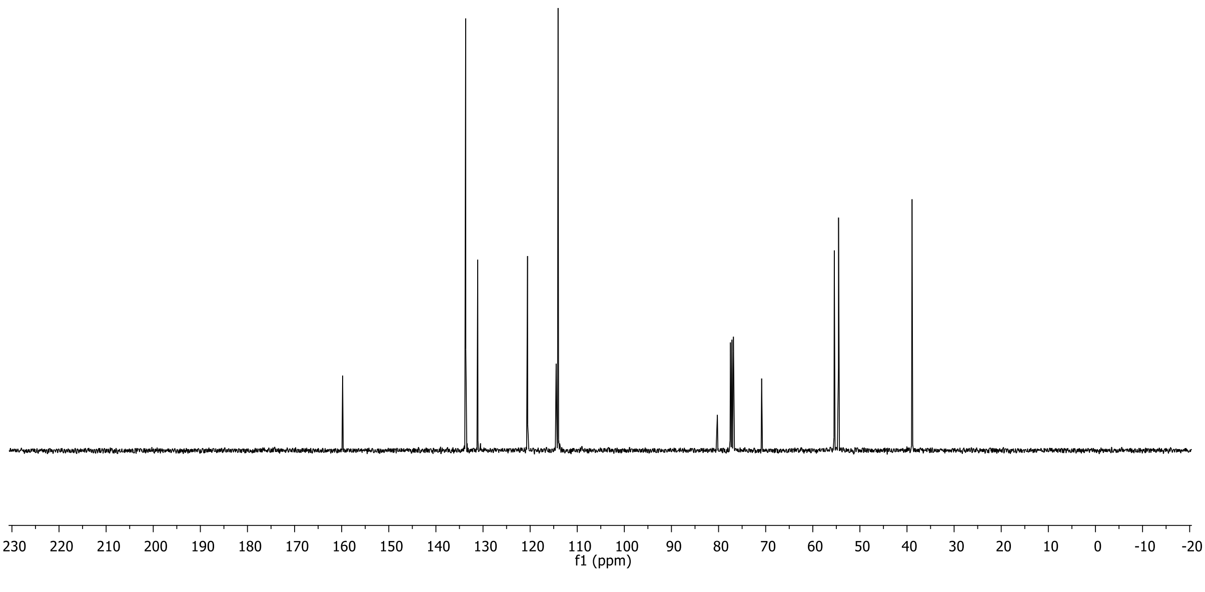
230 220 210 200 190 180 170 160 150 140 130 120 110 100 90 80 70 60 50 40 30 20 10 0 -10 -20
f1 (ppm)

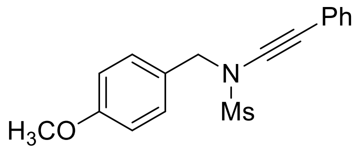
Ph
N
Ms
H3CO

7a

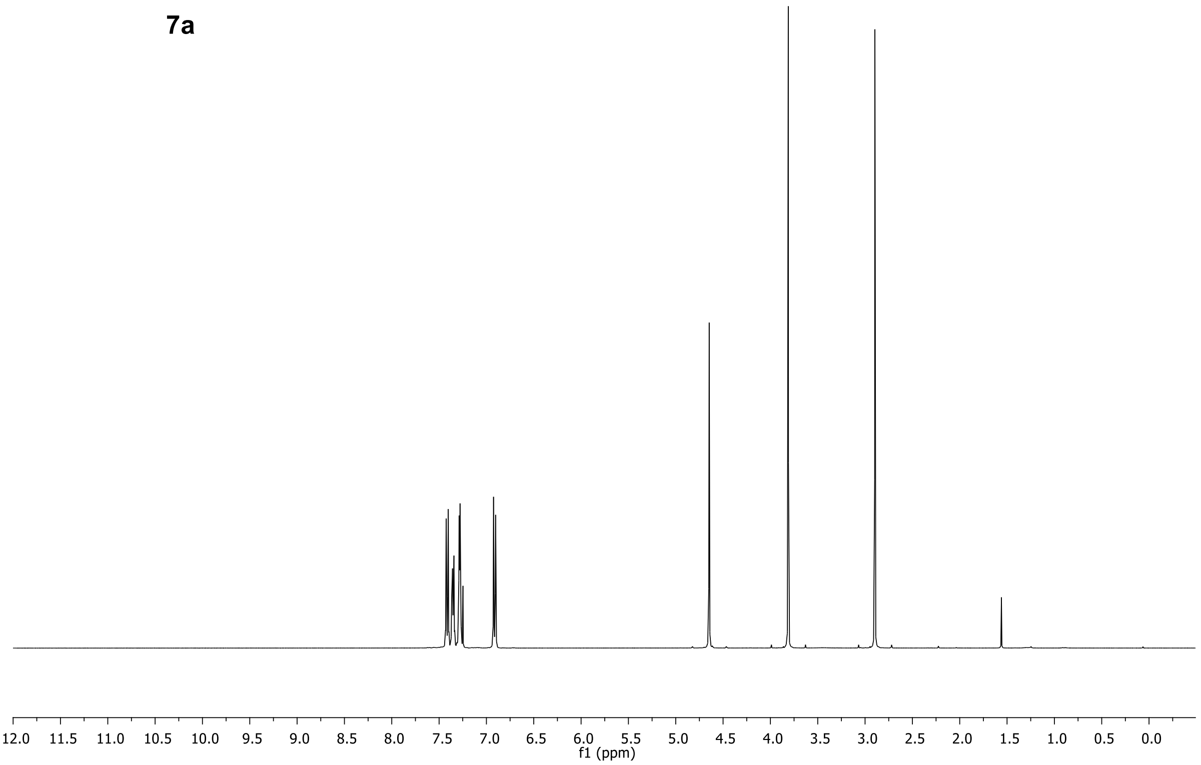
12.0 11.5 11.0 10.5 10.0 9.5 9.0 8.5 8.0 7.5 7.0 6.5 6.0 5.5 5.0 4.5 4.0 3.5 3.0 2.5 2.0 1.5 1.0 0.5 0.0
f1 (ppm)

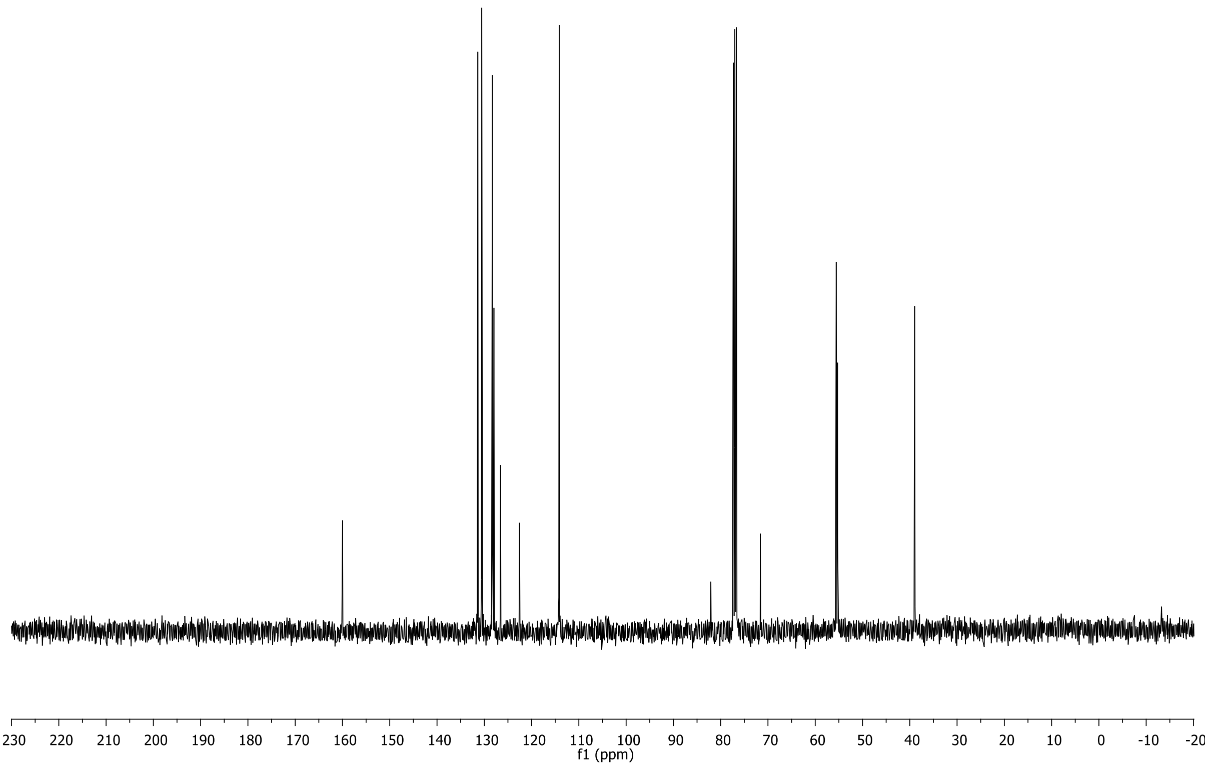
230 220 210 200 190 180 170 160 150 140 130 120 110 100 90 80 70 60 50 40 30 20 10 0 -10 -20
f1 (ppm)

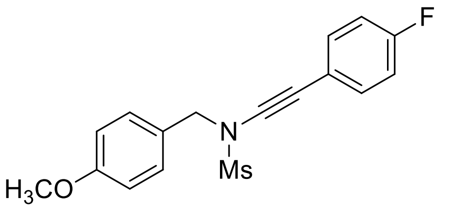

7b

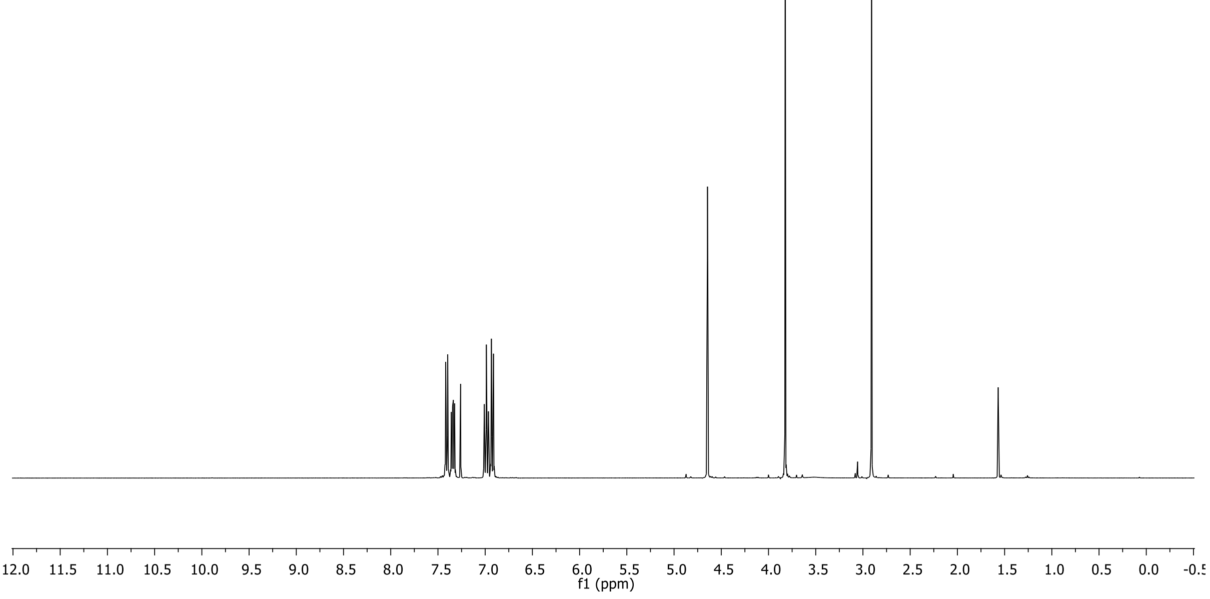

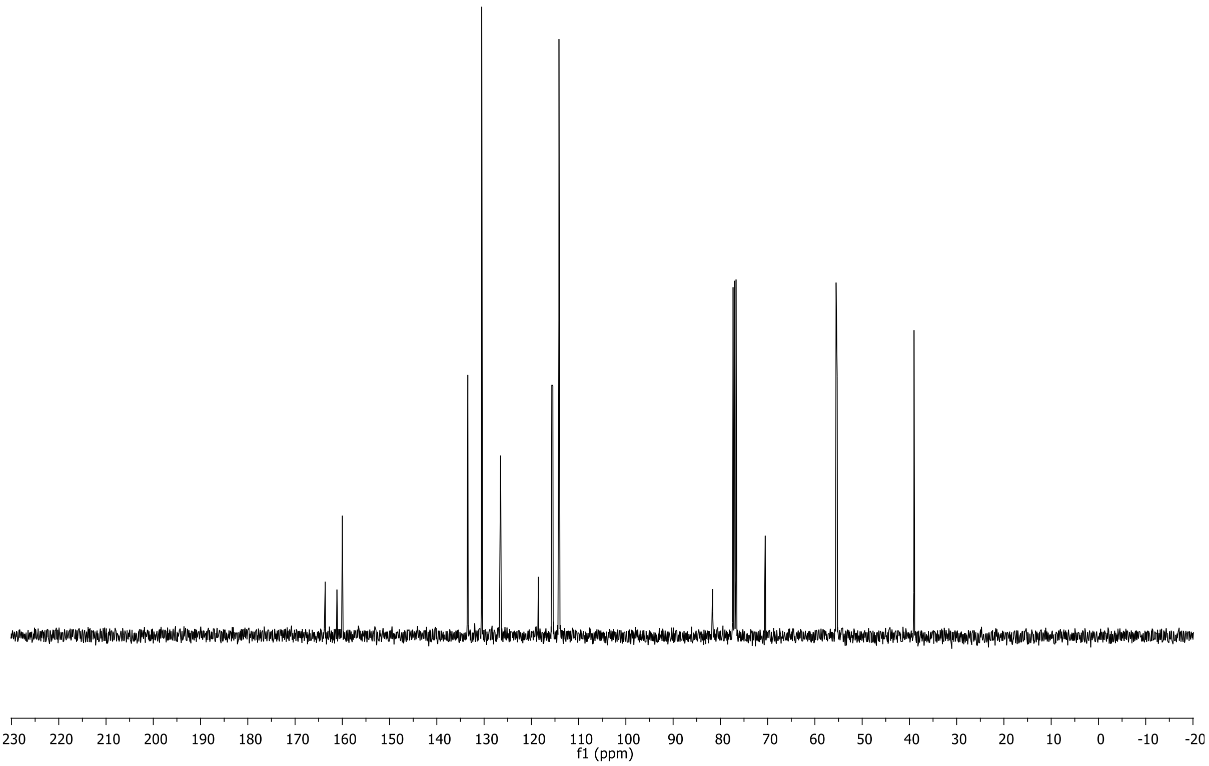

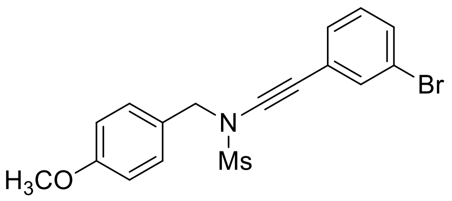
Br
N
Ms
H3CO

7c

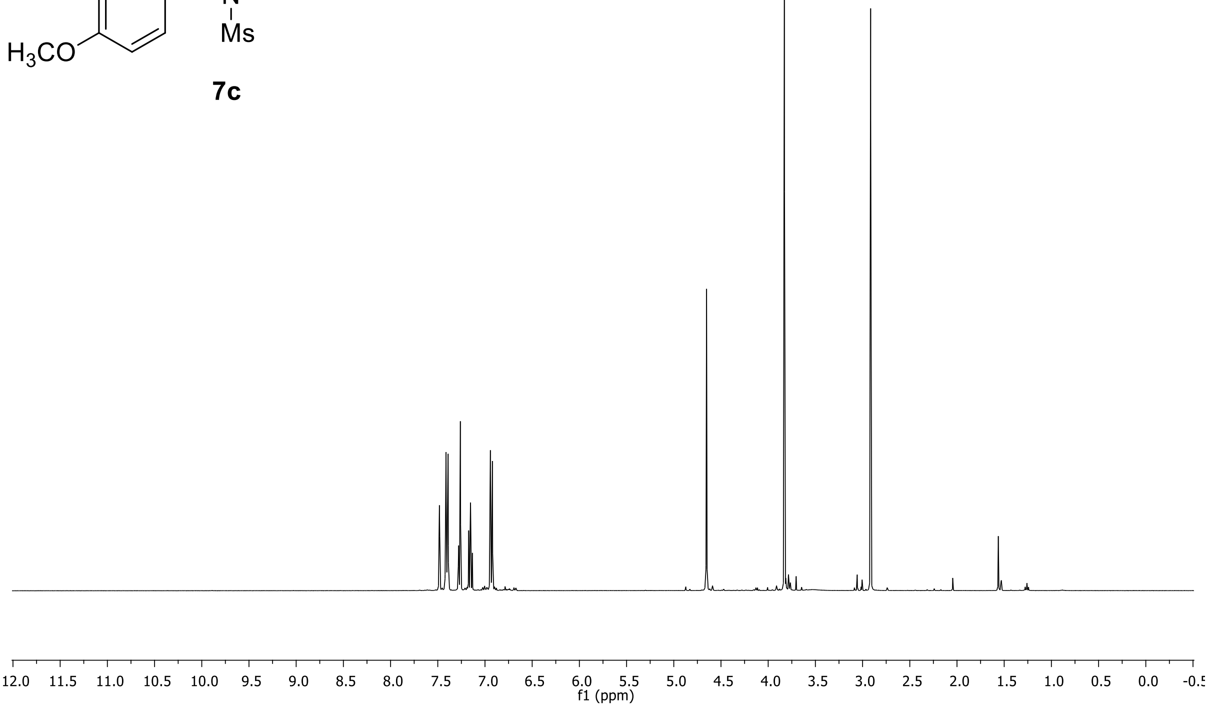
12.0 11.5 11.0 10.5 10.0 9.5 9.0 8.5 8.0 7.5 7.0 6.5 6.0 5.5 5.0 4.5 4.0 3.5 3.0 2.5 2.0 1.5 1.0 0.5 0.0 -0.5
f1 (ppm)

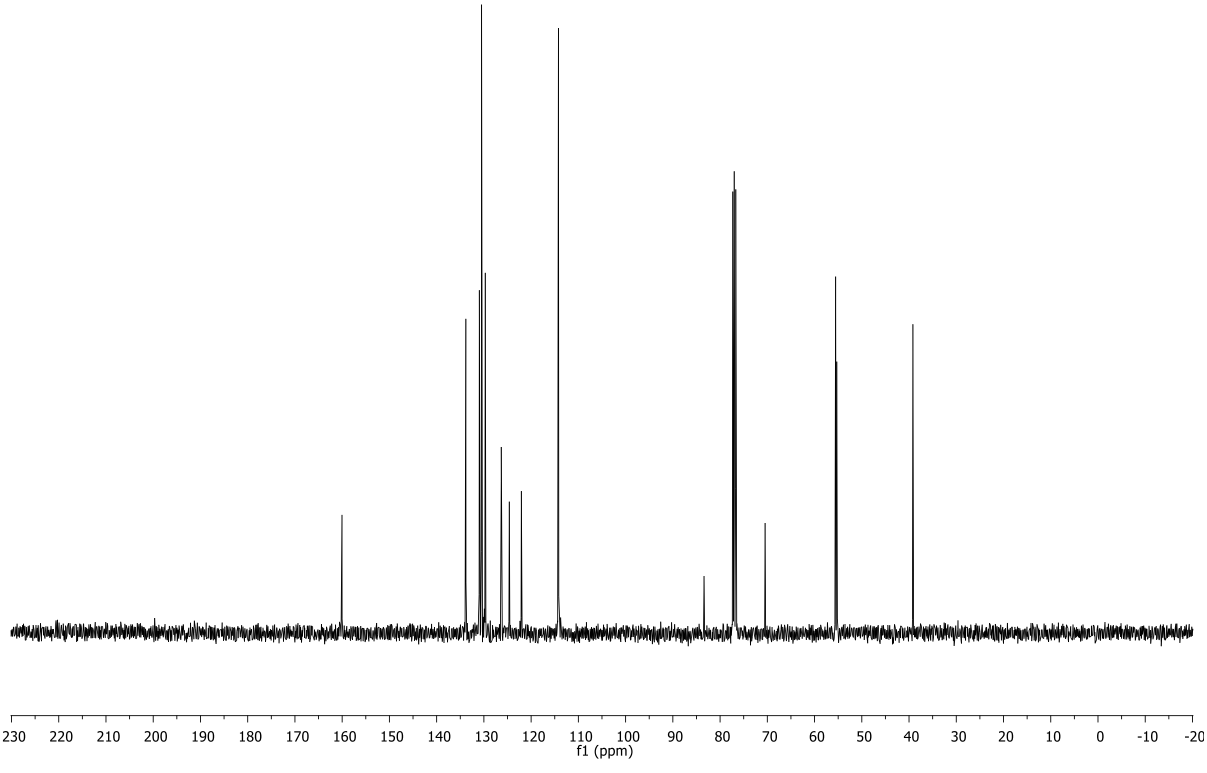
230 220 210 200 190 180 170 160 150 140 130 120 110 100 90 80 70 60 50 40 30 20 10 0 -10 -20
f1 (ppm)

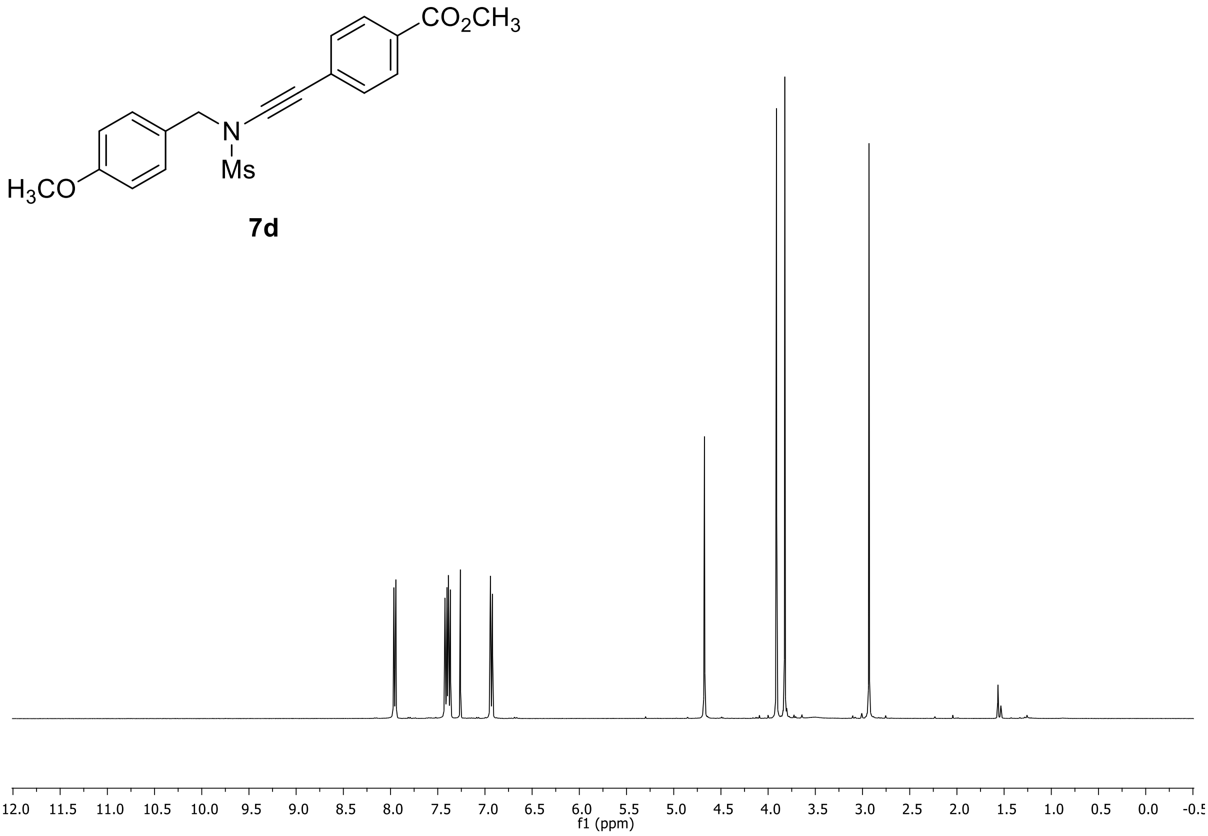
CO2CH3
N
Ms
H3CO
7d
12.0 11.5 11.0 10.5 10.0 9.5 9.0 8.5 8.0 7.5 7.0 6.5 6.0 5.5 5.0 4.5 4.0 3.5 3.0 2.5 2.0 1.5 1.0 0.5 0.0
f1 (ppm)

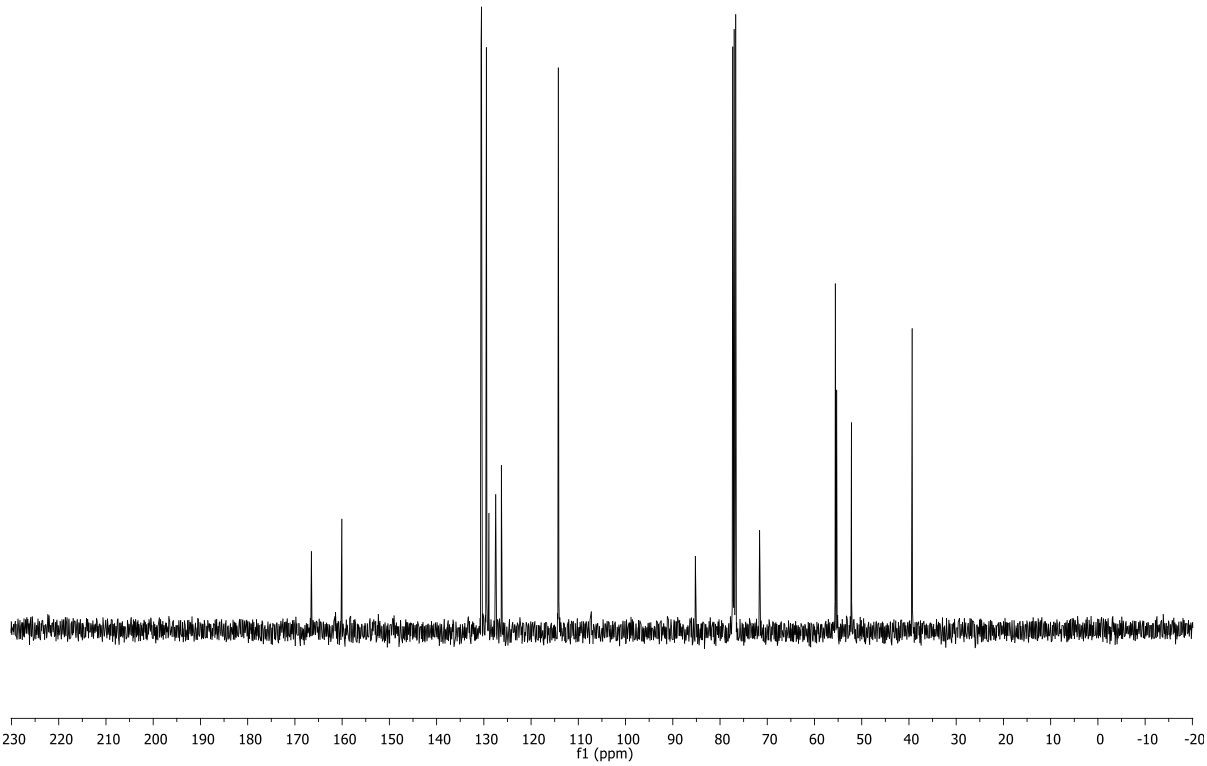
230 220 210 200 190 180 170 160 150 140 130 120 110 100 90 80 70 60 50 40 30 20 10 0 -10 -20
f1 (ppm)

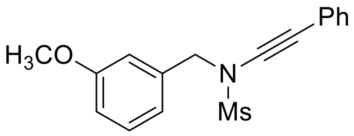
Ph
H3CO
N
Ms

7e

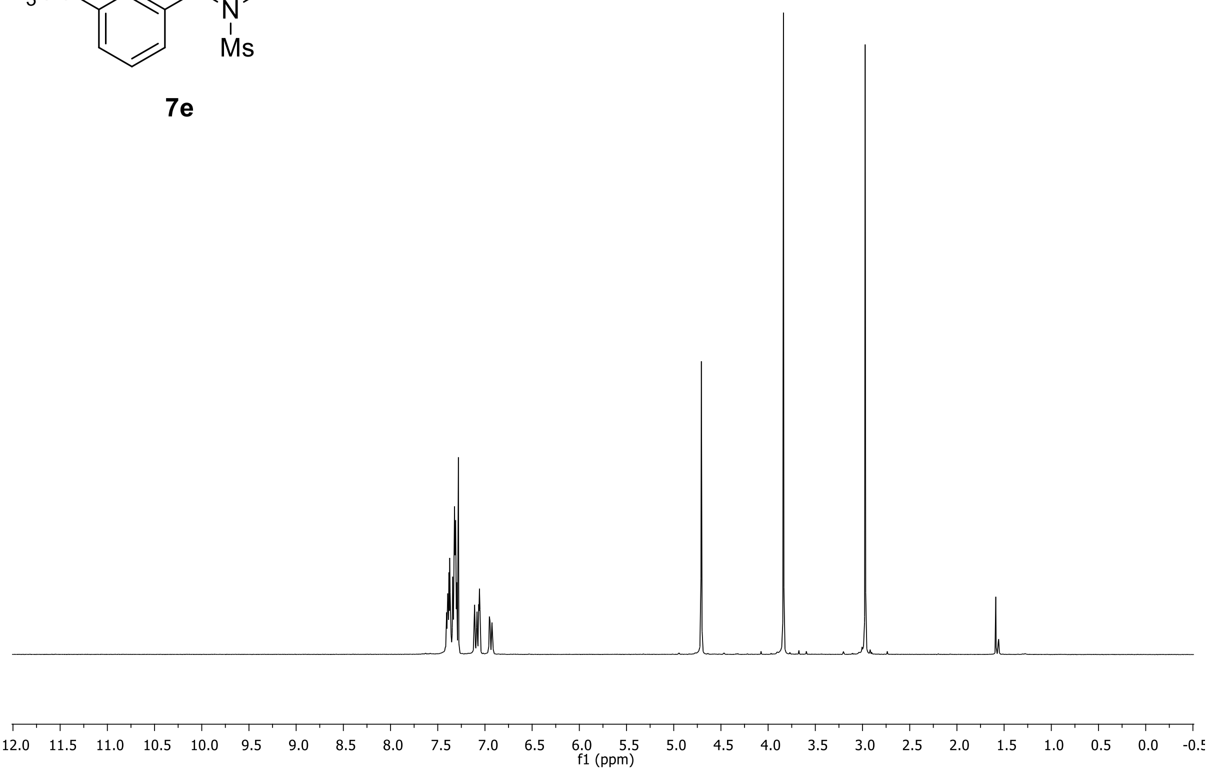
12.0 11.5 11.0 10.5 10.0 9.5 9.0 8.5 8.0 7.5 7.0 6.5 6.0 5.5 5.0 4.5 4.0 3.5 3.0 2.5 2.0 1.5 1.0 0.5 0.0
f1 (ppm)

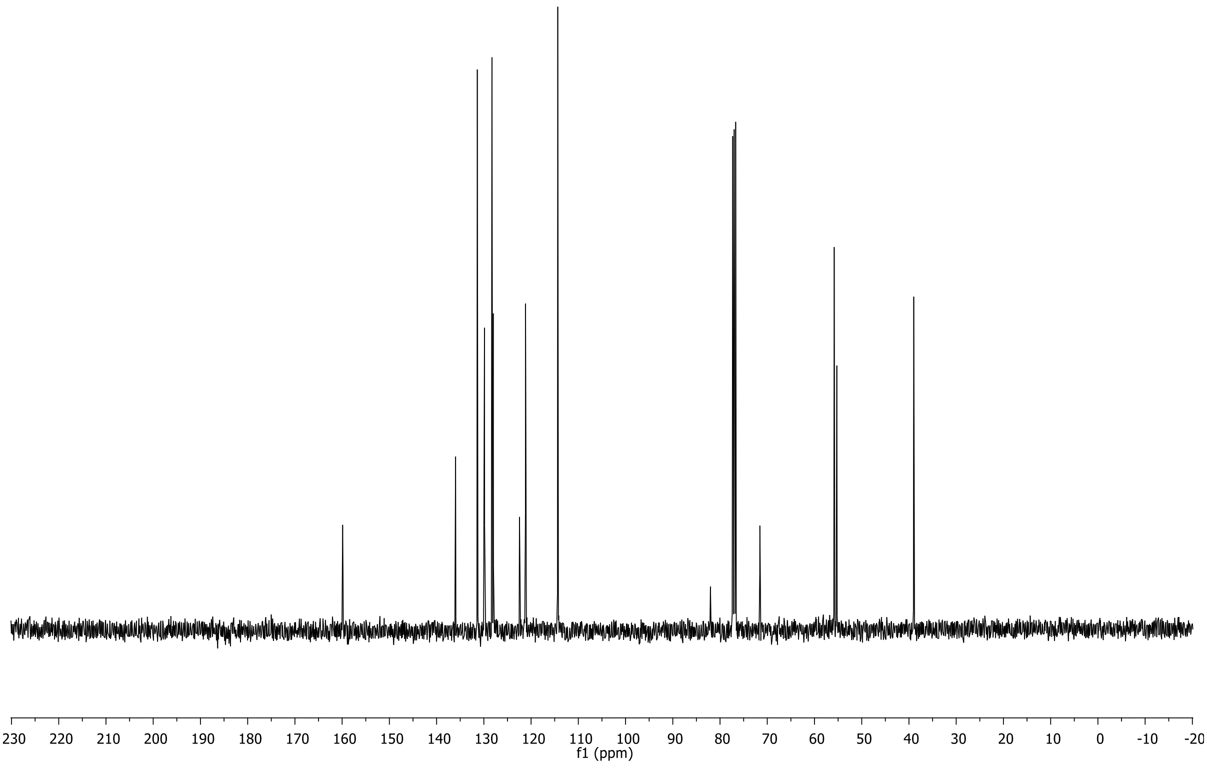
230 220 210 200 190 180 170 160 150 140 130 120 110 100 90 80 70 60 50 40 30 20 10 0 -10 -20
f1 (ppm)

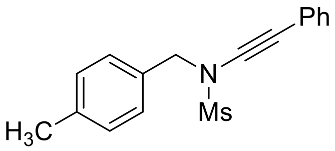
Ph
N
Ms
H3C

7f

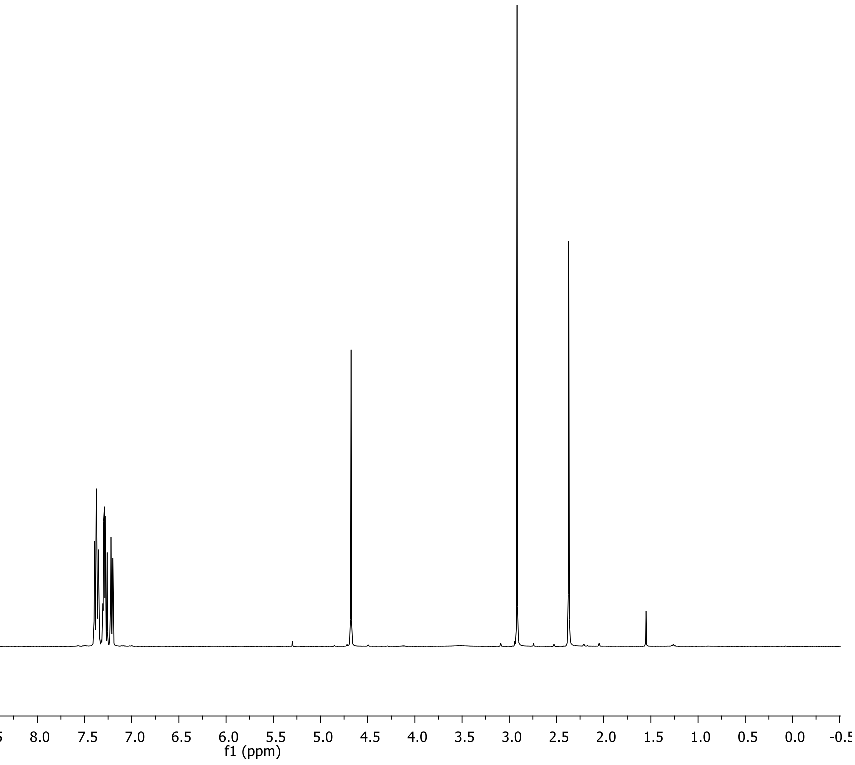
12.0 11.5 11.0 10.5 10.0 9.5 9.0 8.5 8.0 7.5 7.0 6.5 6.0 5.5 5.0 4.5 4.0 3.5 3.0 2.5 2.0 1.5 1.0 0.5 0.0
f1 (ppm)

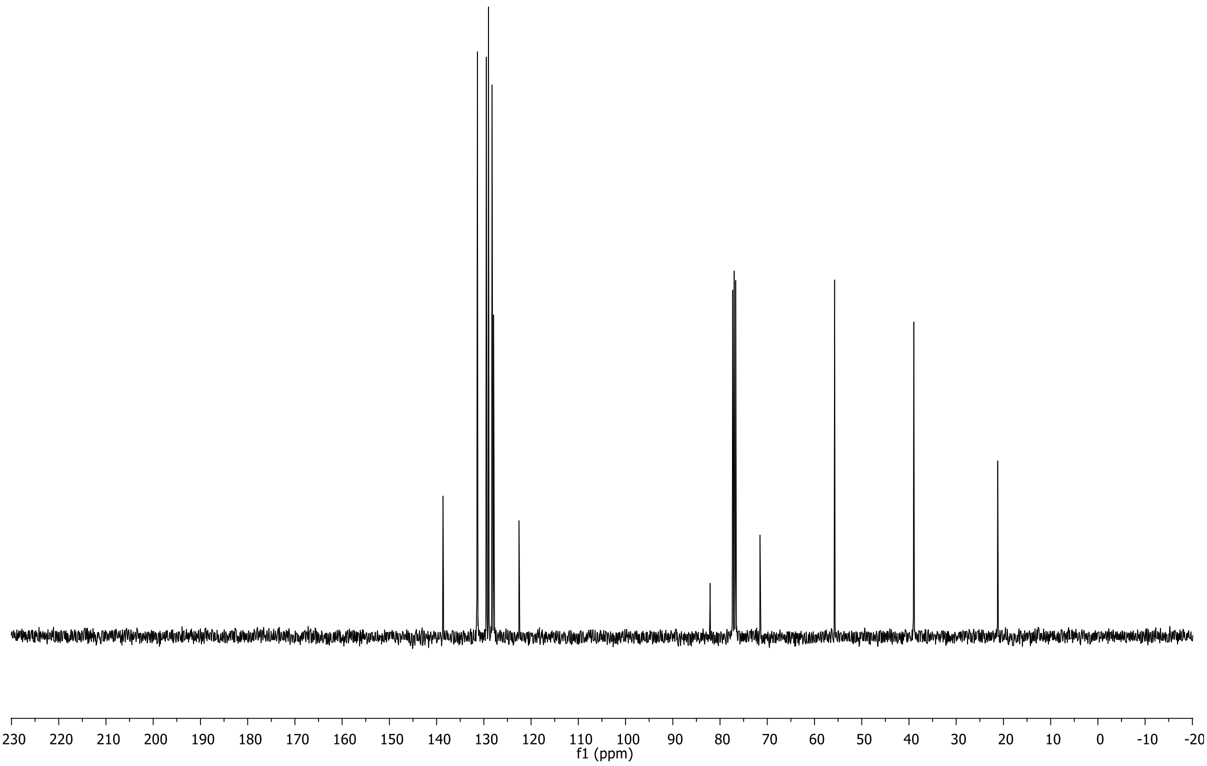
230 220 210 200 190 180 170 160 150 140 130 120 110 100 90 80 70 60 50 40 30 20 10 0 -10 -20
f1 (ppm)

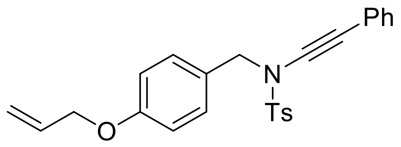
Ph
N
Ts
O

7g

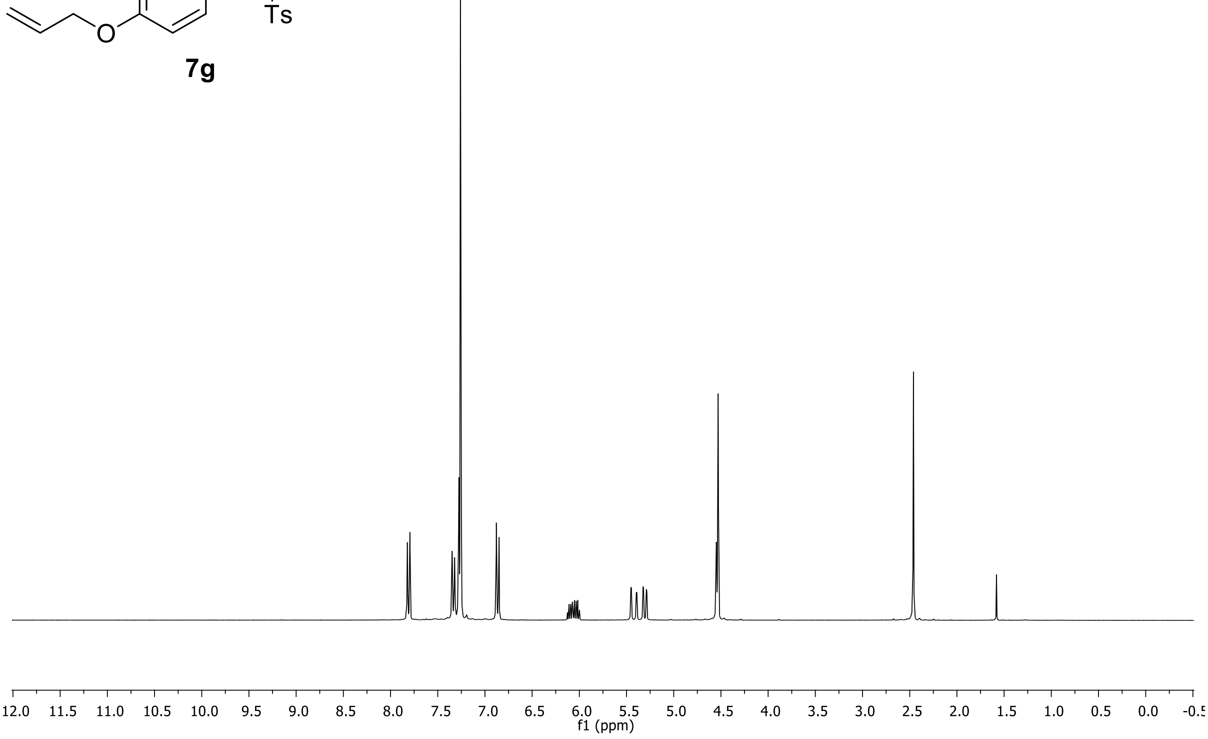
12.0 11.5 11.0 10.5 10.0 9.5 9.0 8.5 8.0 7.5 7.0 6.5 6.0 5.5 5.0 4.5 4.0 3.5 3.0 2.5 2.0 1.5 1.0 0.5 0.0 -0.5
f1 (ppm)

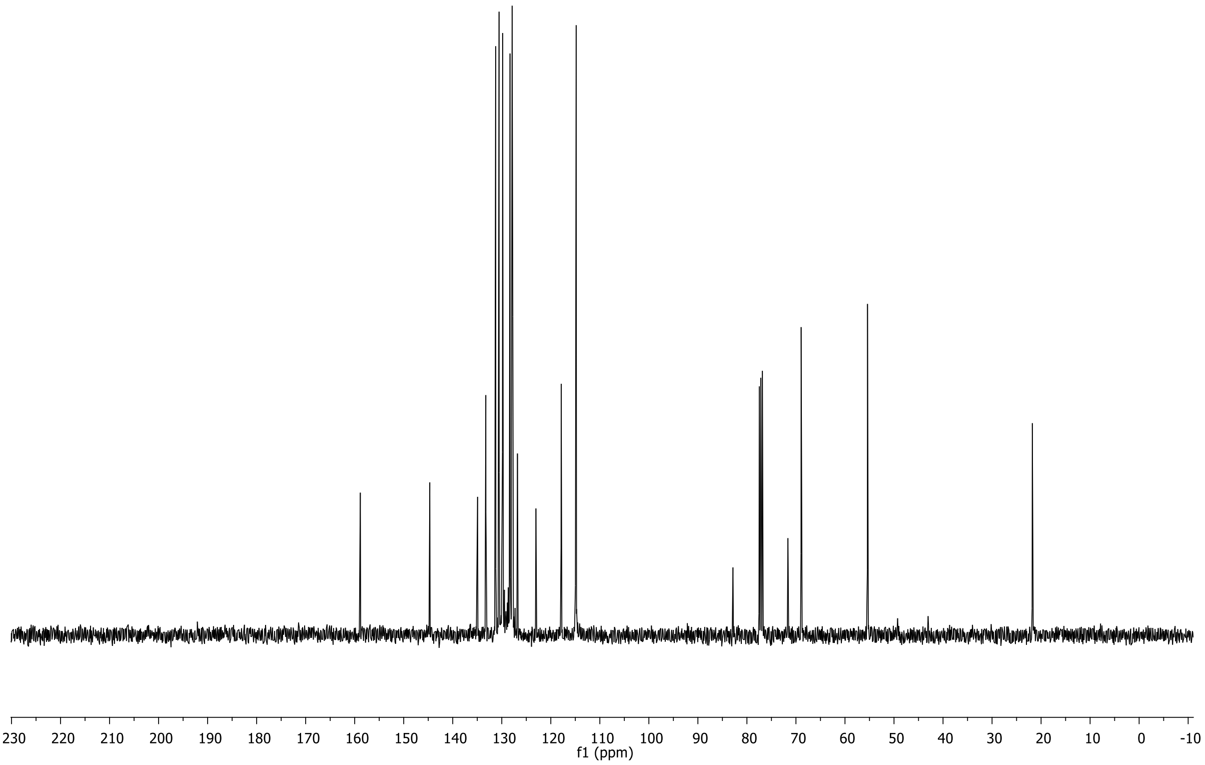
230 220 210 200 190 180 170 160 150 140 130 120 110 100 90 80 70 60 50 40 30 20 10 0 -10
f1 (ppm)

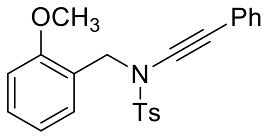

7h

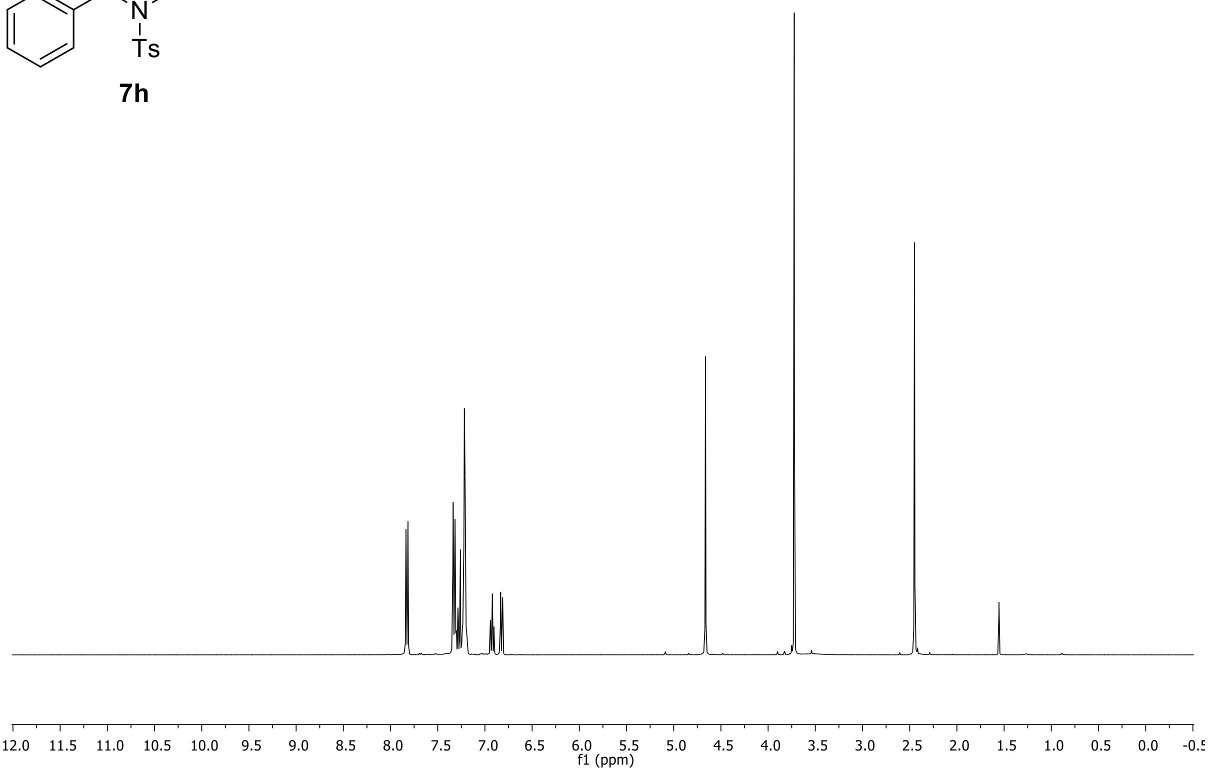

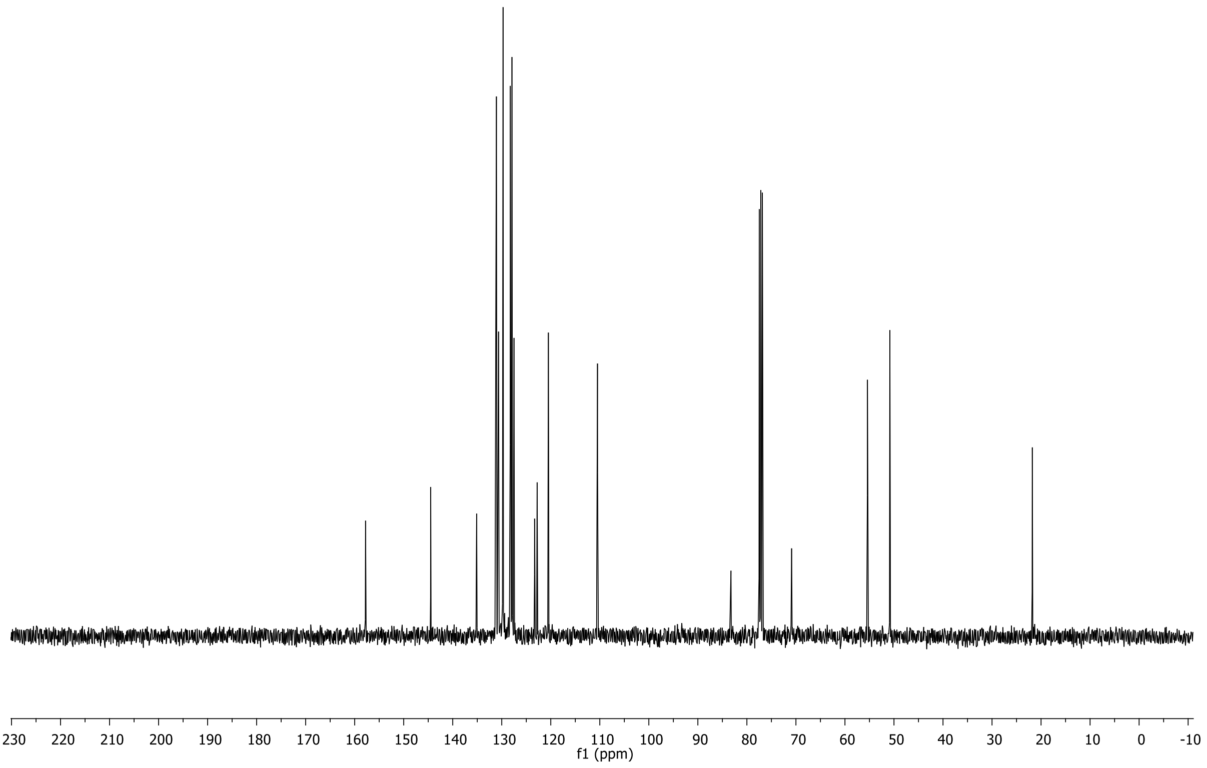

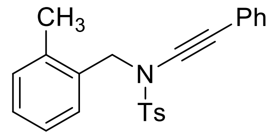
CH3
Ph
N
Ts

7i

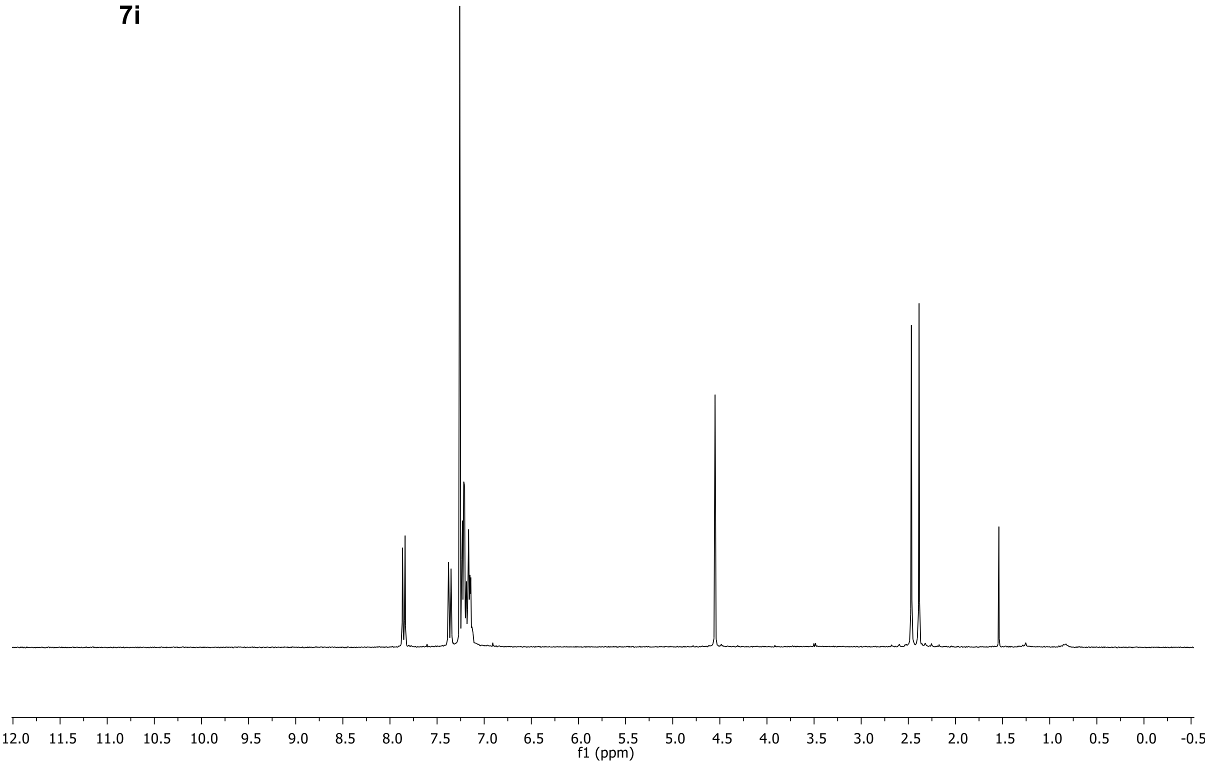
12.0 11.5 11.0 10.5 10.0 9.5 9.0 8.5 8.0 7.5 7.0 6.5 6.0 5.5 5.0 4.5 4.0 3.5 3.0 2.5 2.0 1.5 1.0 0.5 0.0 -0.5
f1 (ppm)

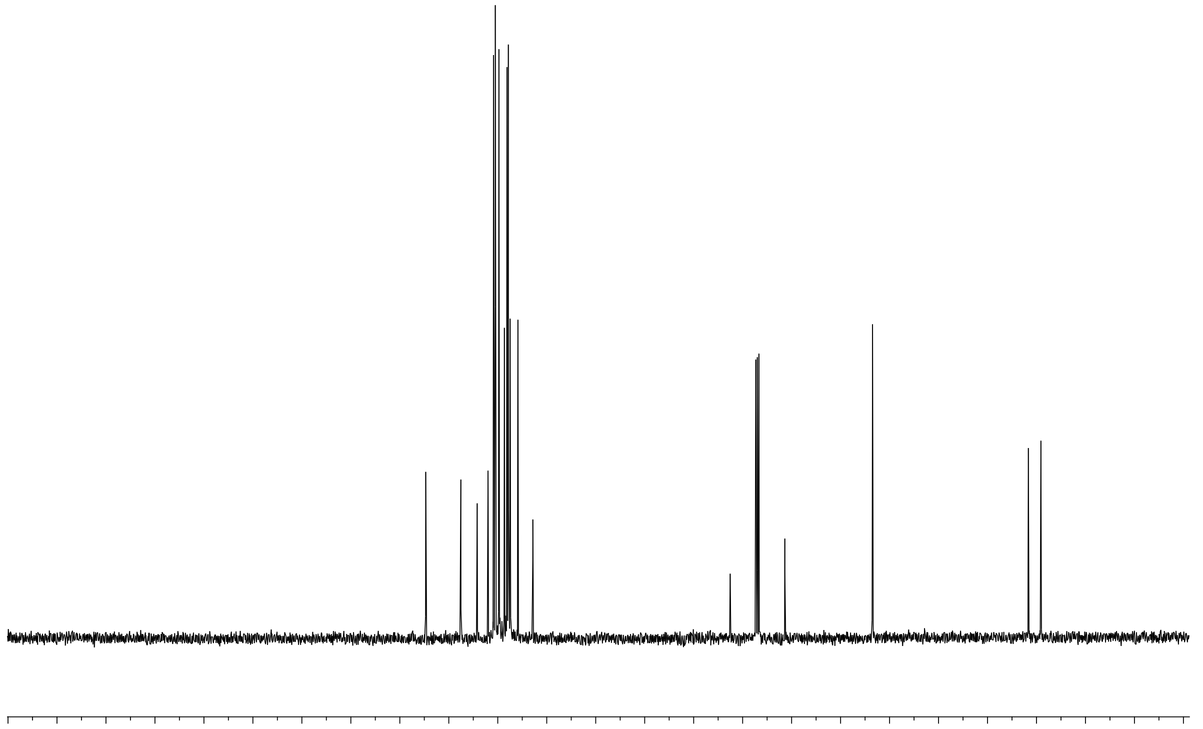
230 220 210 200 190 180 170 160 150 140 130 120 110 100 90 80 70 60 50 40 30 20 10 0 -10
f1 (ppm)

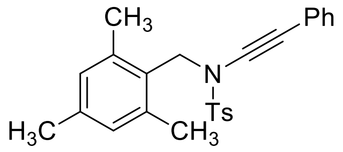
CH3
Ph
N
Ts
H3C
CH3

7j

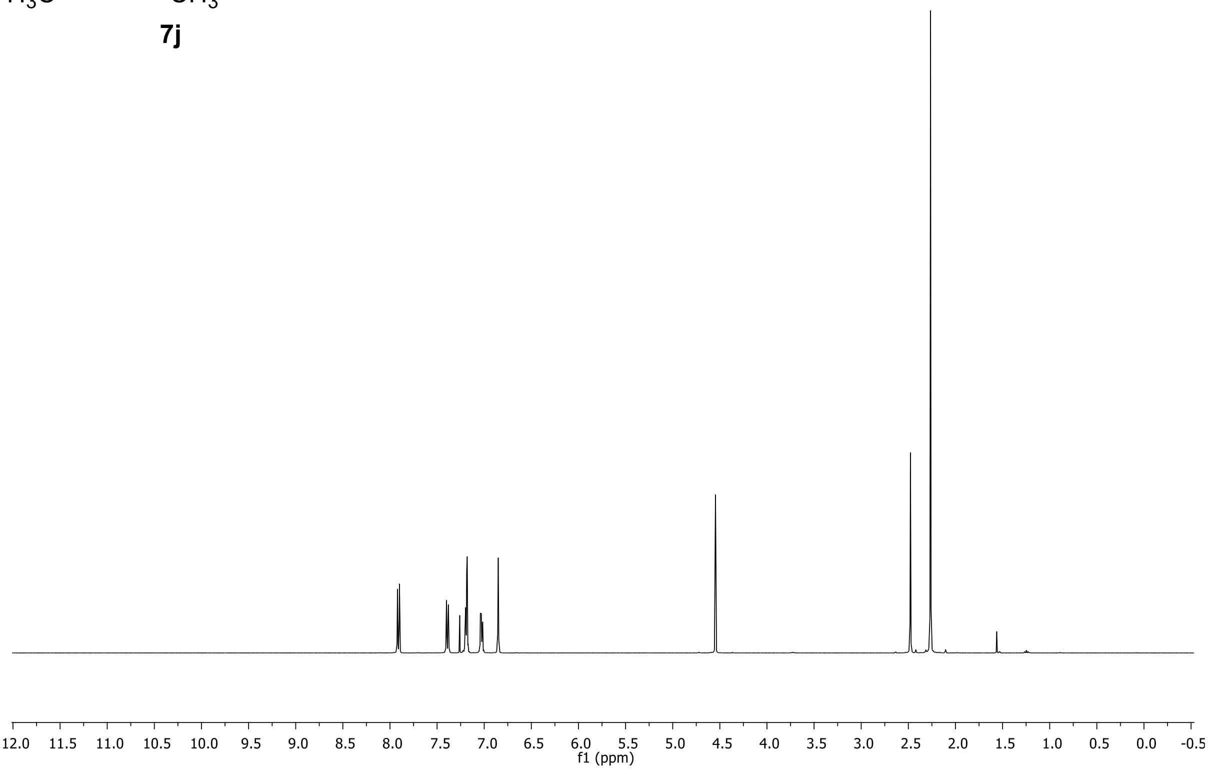
12.0 11.5 11.0 10.5 10.0 9.5 9.0 8.5 8.0 7.5 7.0 6.5 6.0 5.5 5.0 4.5 4.0 3.5 3.0 2.5 2.0 1.5 1.0 0.5 0.0 -0.5
f1 (ppm)

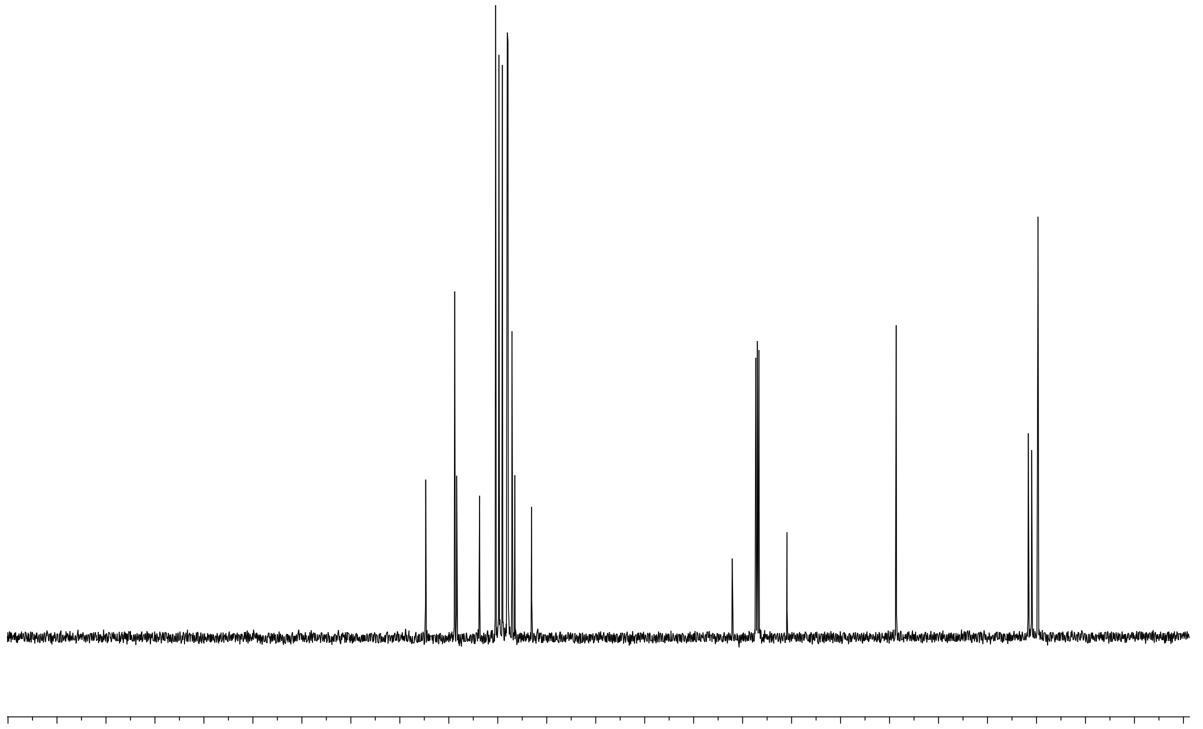
230 220 210 200 190 180 170 160 150 140 130 120 110 100 90 80 70 60 50 40 30 20 10 0 -10
f1 (ppm)

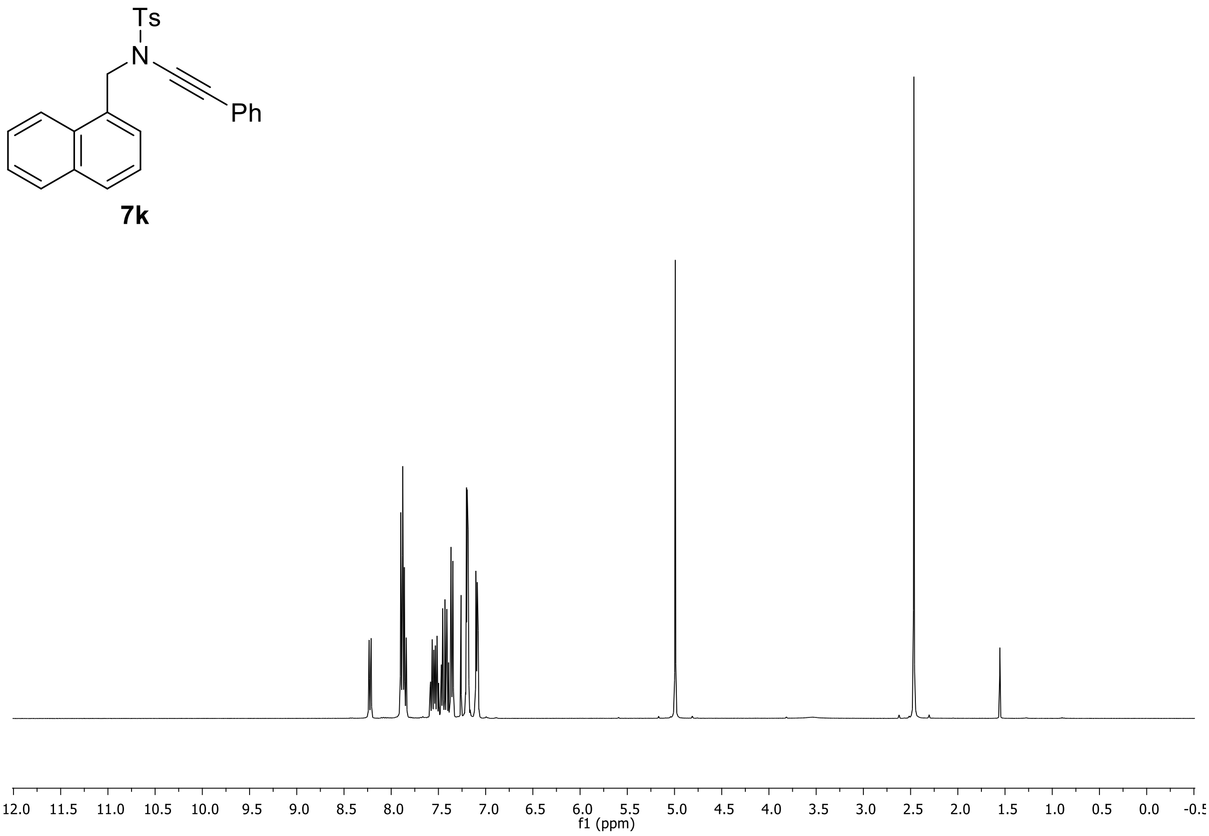
Ts
N
Ph

7k

12.0 11.5 11.0 10.5 10.0 9.5 9.0 8.5 8.0 7.5 7.0 6.5 6.0 5.5 5.0 4.5 4.0 3.5 3.0 2.5 2.0 1.5 1.0 0.5 0.0
f1 (ppm)

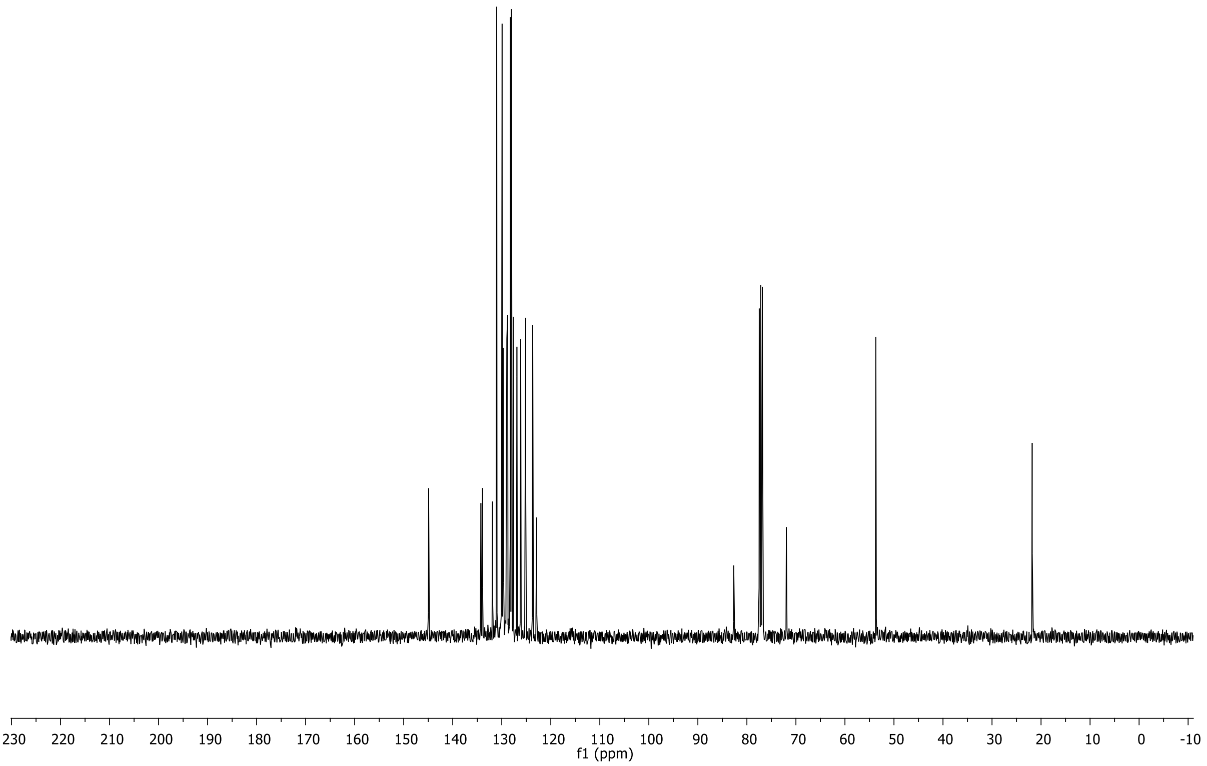
230 220 210 200 190 180 170 160 150 140 130 120 110 100 90 80 70 60 50 40 30 20 10 0 -10
f1 (ppm)

^{1}H and ^{13}C NMR Spectra of Novel Catalysis Products

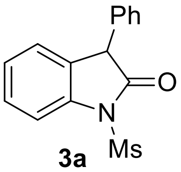

3a

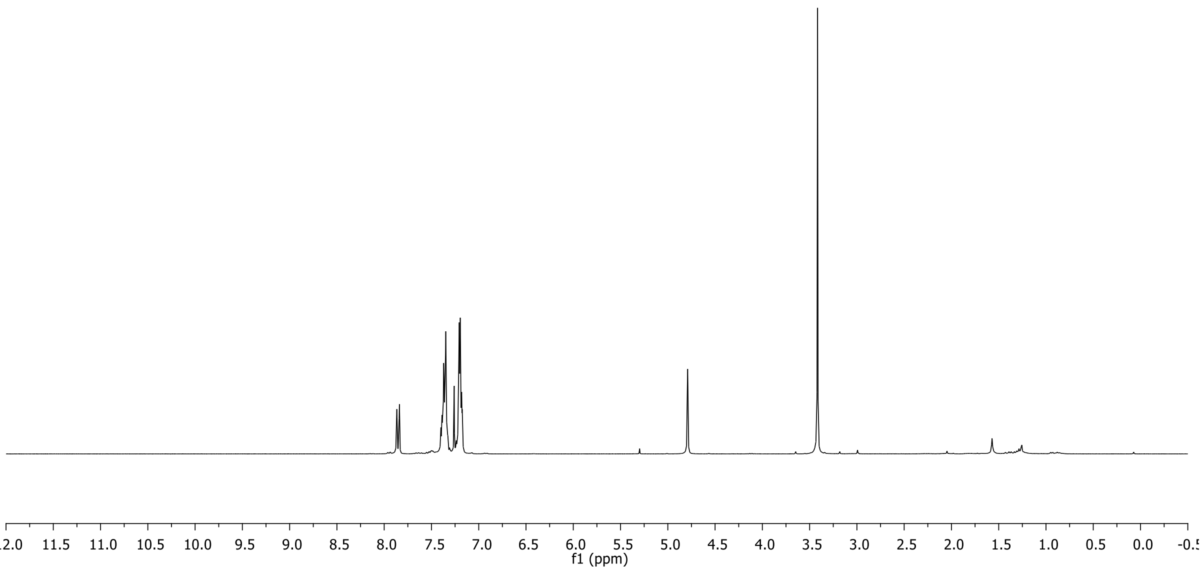

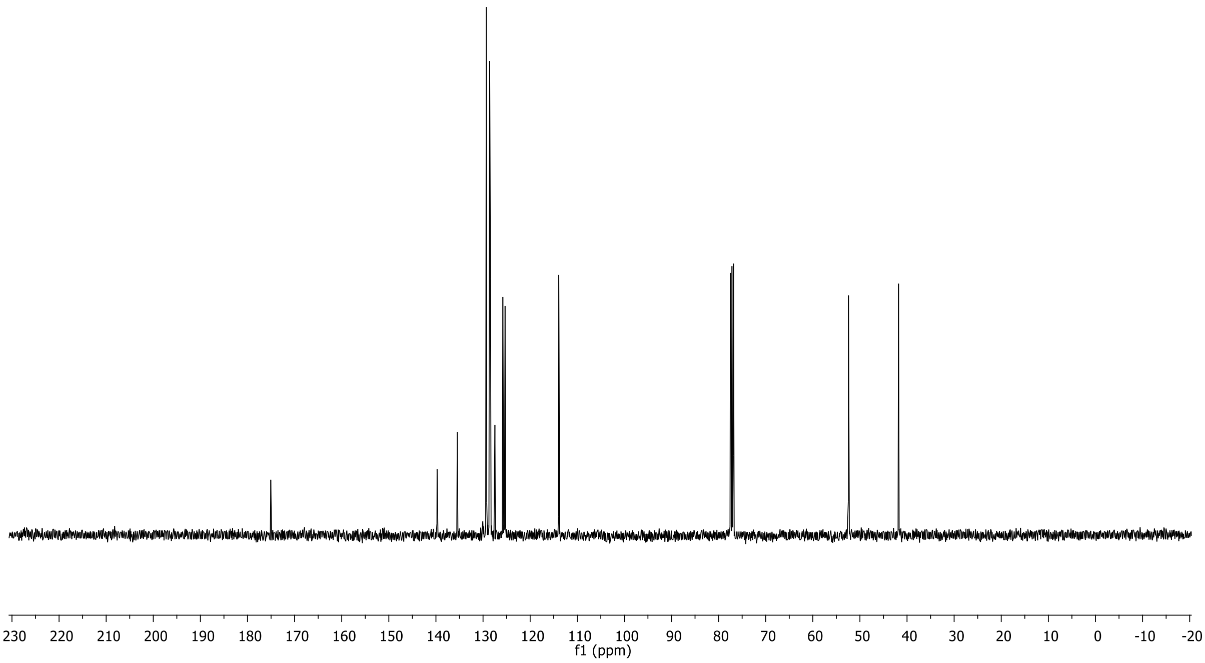

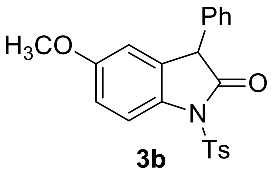
Ph
H3CO
O
N
Ts

3b

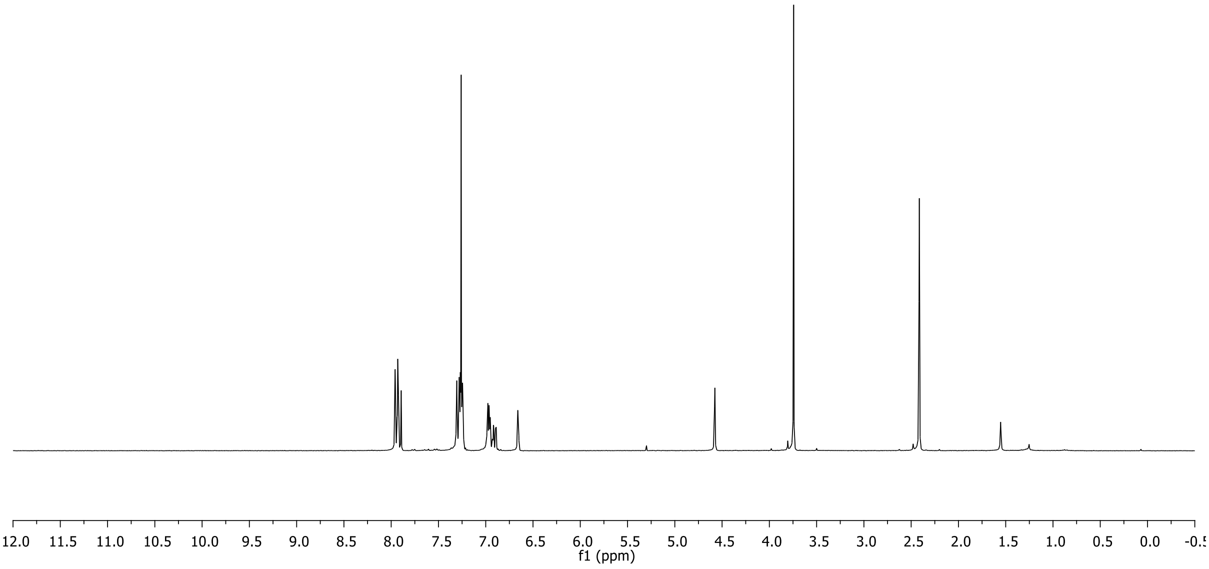
12.0 11.5 11.0 10.5 10.0 9.5 9.0 8.5 8.0 7.5 7.0 6.5 6.0 5.5 5.0 4.5 4.0 3.5 3.0 2.5 2.0 1.5 1.0 0.5 0.0
f1 (ppm)

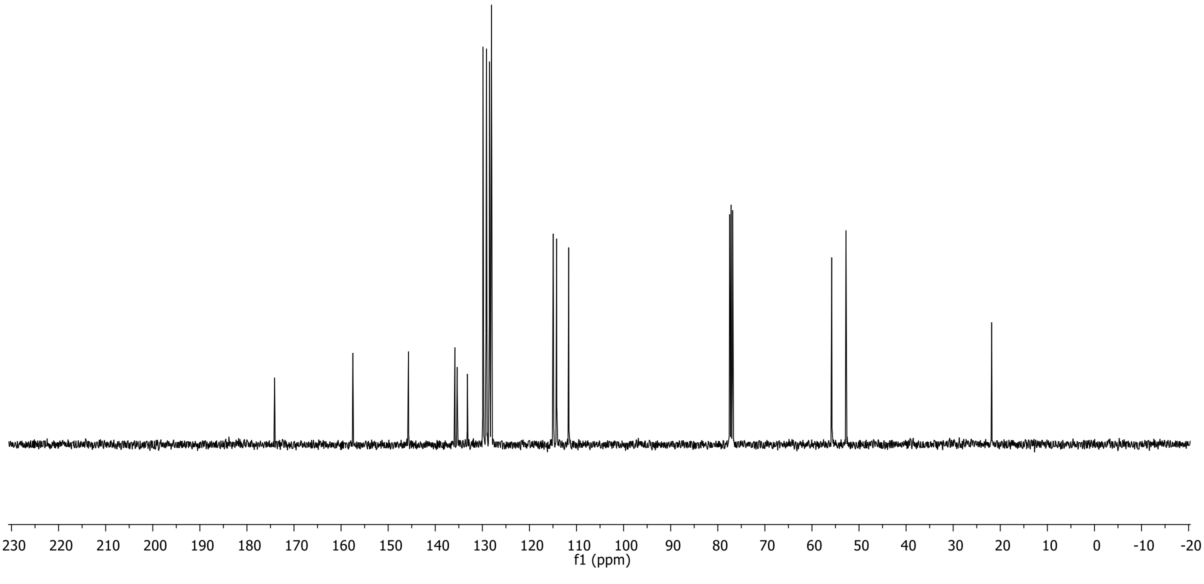
230 220 210 200 190 180 170 160 150 140 130 120 110 100 90 80 70 60 50 40 30 20 10 0 -10 -20
f1 (ppm)

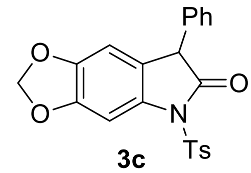
Ph
O
O
N
O
Ts

3c

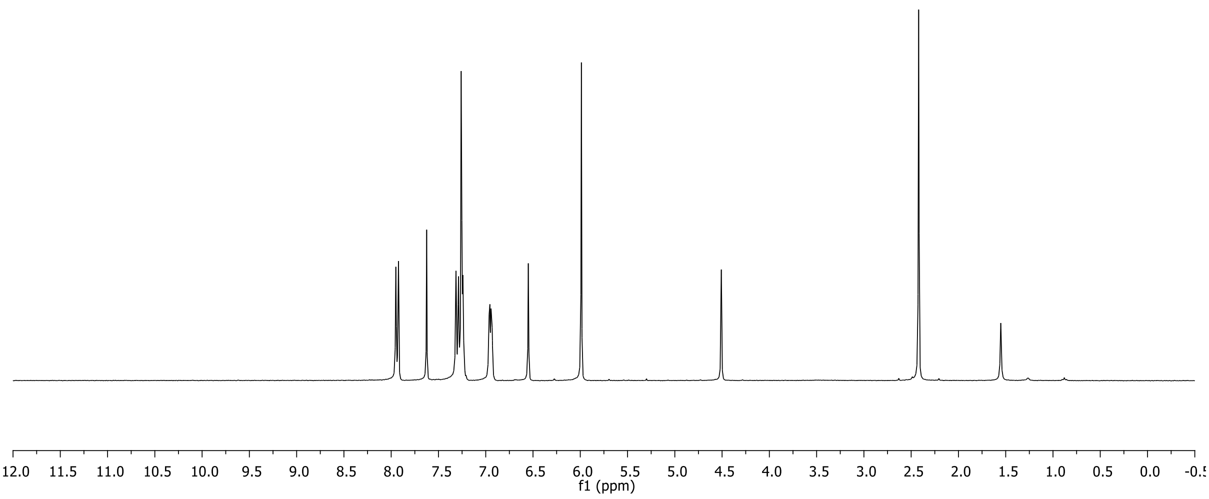
12.0 11.5 11.0 10.5 10.0 9.5 9.0 8.5 8.0 7.5 7.0 6.5 6.0 5.5 5.0 4.5 4.0 3.5 3.0 2.5 2.0 1.5 1.0 0.5 0.0 -0.5
f1 (ppm)

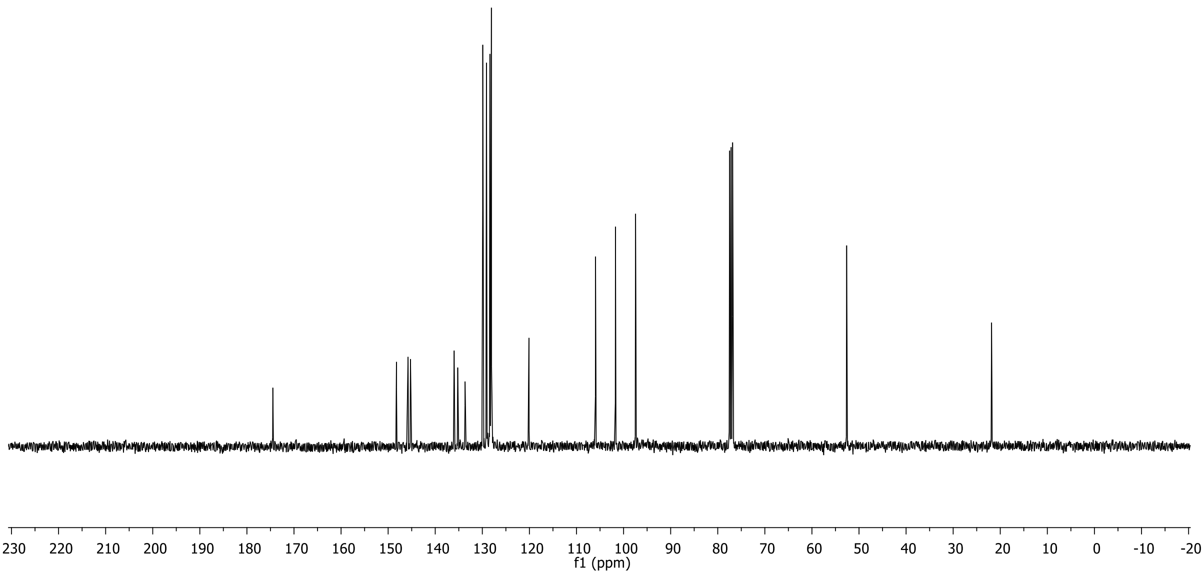
230 220 210 200 190 180 170 160 150 140 130 120 110 100 90 80 70 60 50 40 30 20 10 0 -10 -20
f1 (ppm)

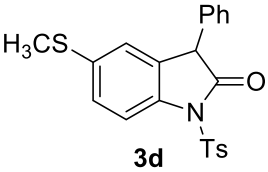
Ph
H3CS
O
N
Ts

3d

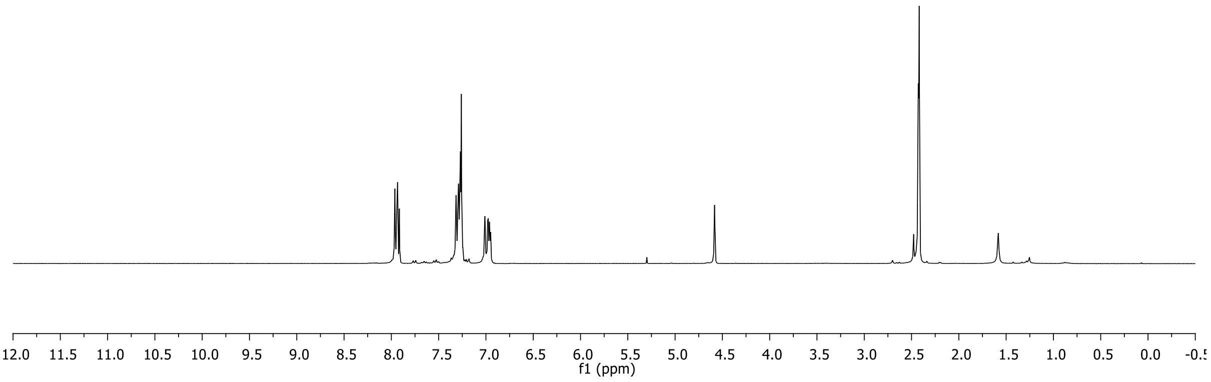
12.0 11.5 11.0 10.5 10.0 9.5 9.0 8.5 8.0 7.5 7.0 6.5 6.0 5.5 5.0 4.5 4.0 3.5 3.0 2.5 2.0 1.5 1.0 0.5 0.0 -0.
f1 (ppm)

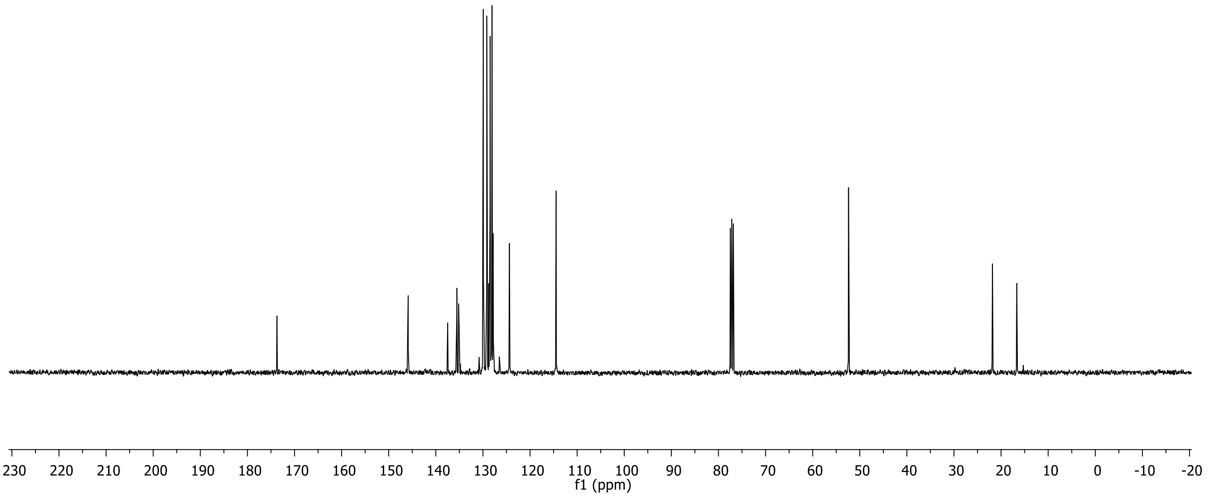
230 220 210 200 190 180 170 160 150 140 130 120 110 100 90 80 70 60 50 40 30 20 10 0 -10 -20
f1 (ppm)

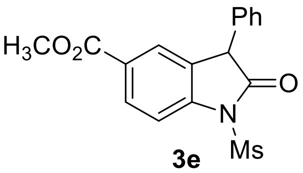
Ph
H3CO2C
O
N
Ms

3e

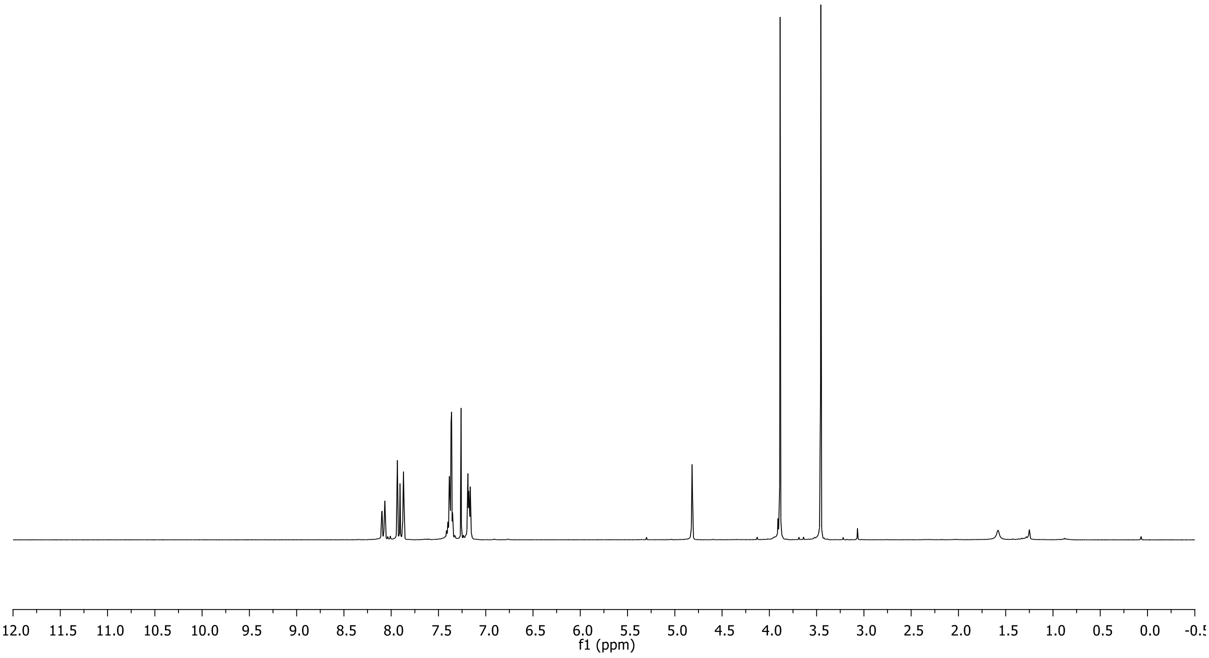
12.0 11.5 11.0 10.5 10.0 9.5 9.0 8.5 8.0 7.5 7.0 6.5 6.0 5.5 5.0 4.5 4.0 3.5 3.0 2.5 2.0 1.5 1.0 0.5 0.0
f1 (ppm)

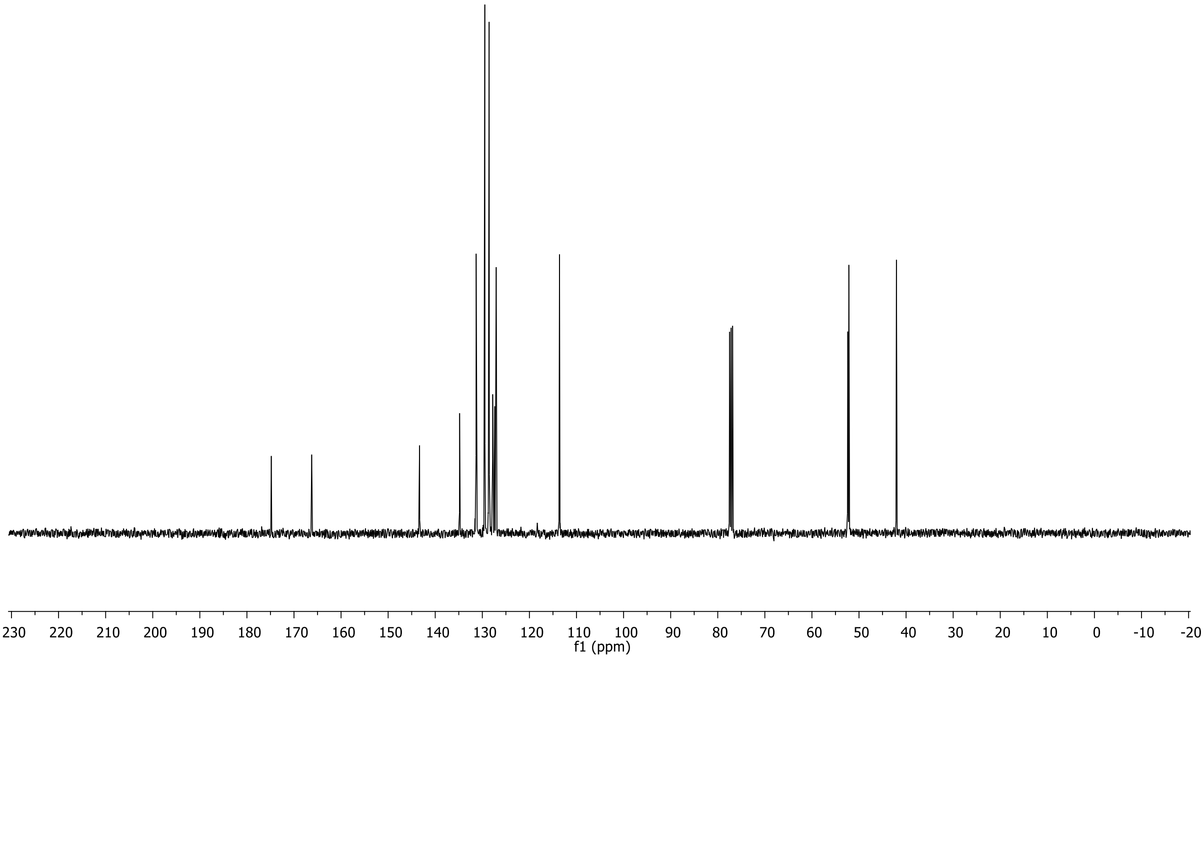
230 220 210 200 190 180 170 160 150 140 130 120 110 100 90 80 70 60 50 40 30 20 10 0 -10 -20
f1 (ppm)

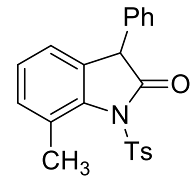
Ph
O
N
Ts
CH3

3f

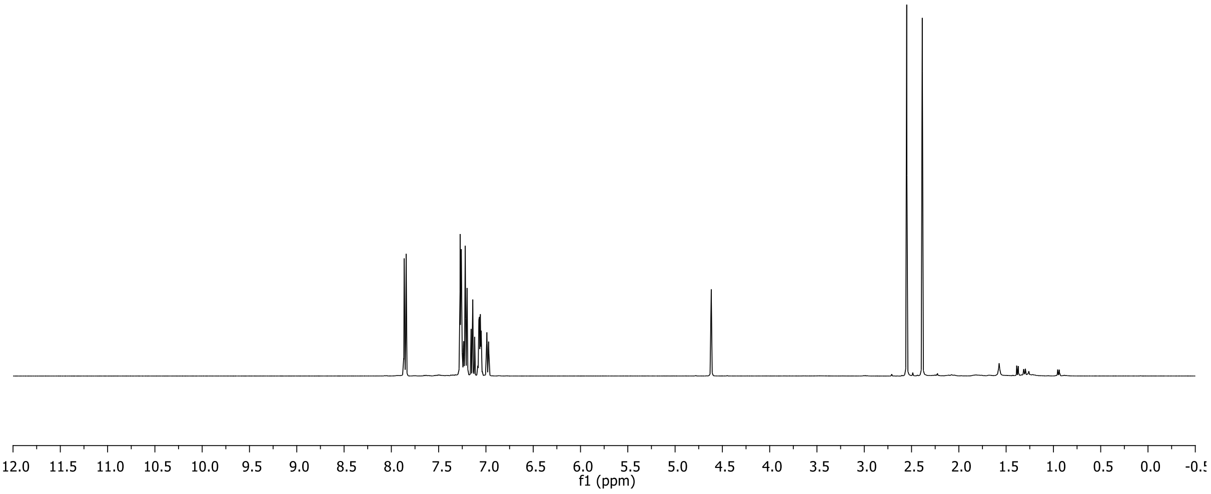
12.0 11.5 11.0 10.5 10.0 9.5 9.0 8.5 8.0 7.5 7.0 6.5 6.0 5.5 5.0 4.5 4.0 3.5 3.0 2.5 2.0 1.5 1.0 0.5 0.0
f1 (ppm)

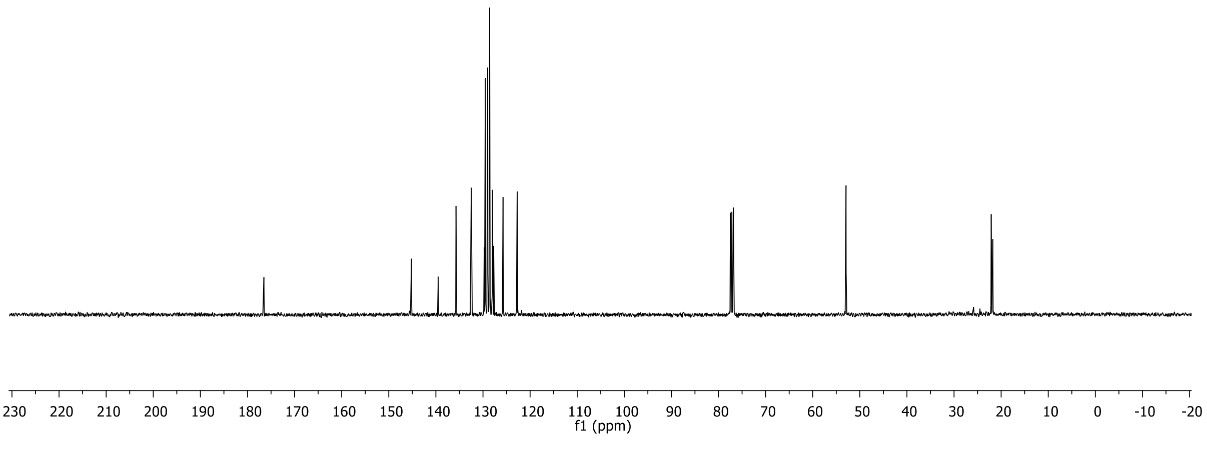
230 220 210 200 190 180 170 160 150 140 130 120 110 100 90 80 70 60 50 40 30 20 10 0 -10 -20
f1 (ppm)

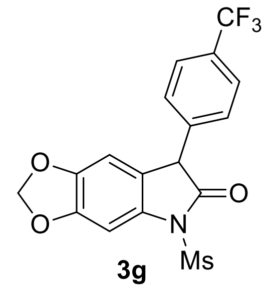
CF3
O
O
O
N
Ms

3g

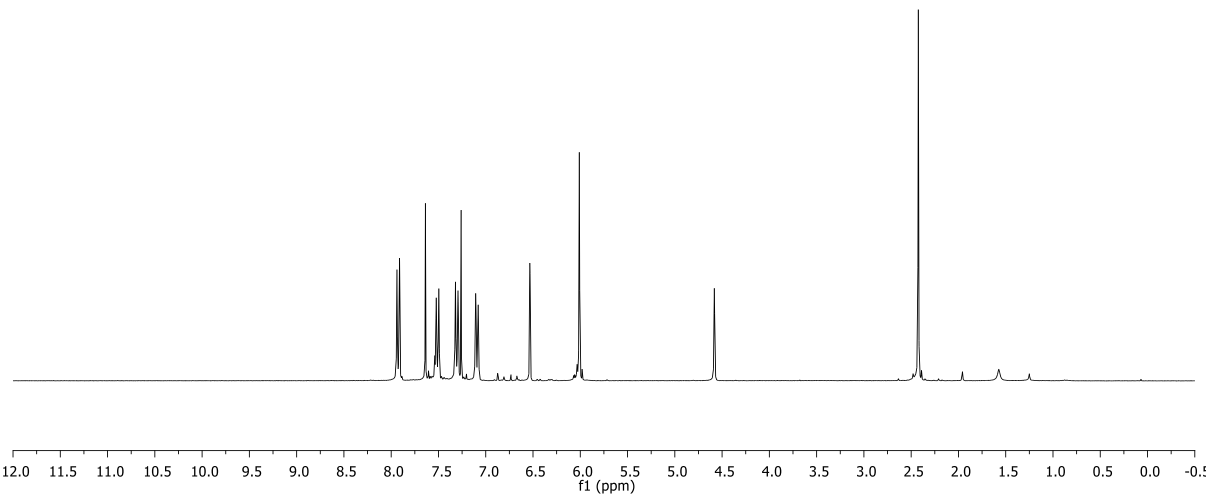
12.0 11.5 11.0 10.5 10.0 9.5 9.0 8.5 8.0 7.5 7.0 6.5 6.0 5.5 5.0 4.5 4.0 3.5 3.0 2.5 2.0 1.5 1.0 0.5 0.0
f1 (ppm)

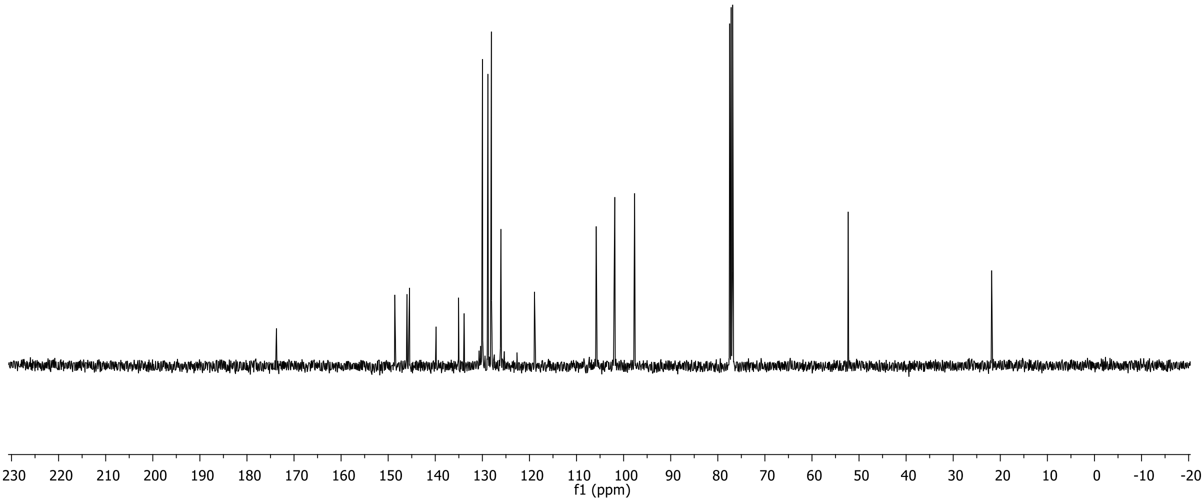
230 220 210 200 190 180 170 160 150 140 130 120 110 100 90 80 70 60 50 40 30 20 10 0 -10 -20
f1 (ppm)

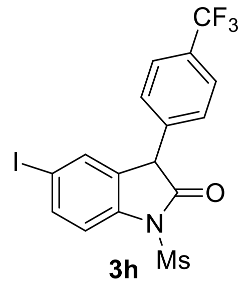
CF_3
I
O
N
Ms

3h

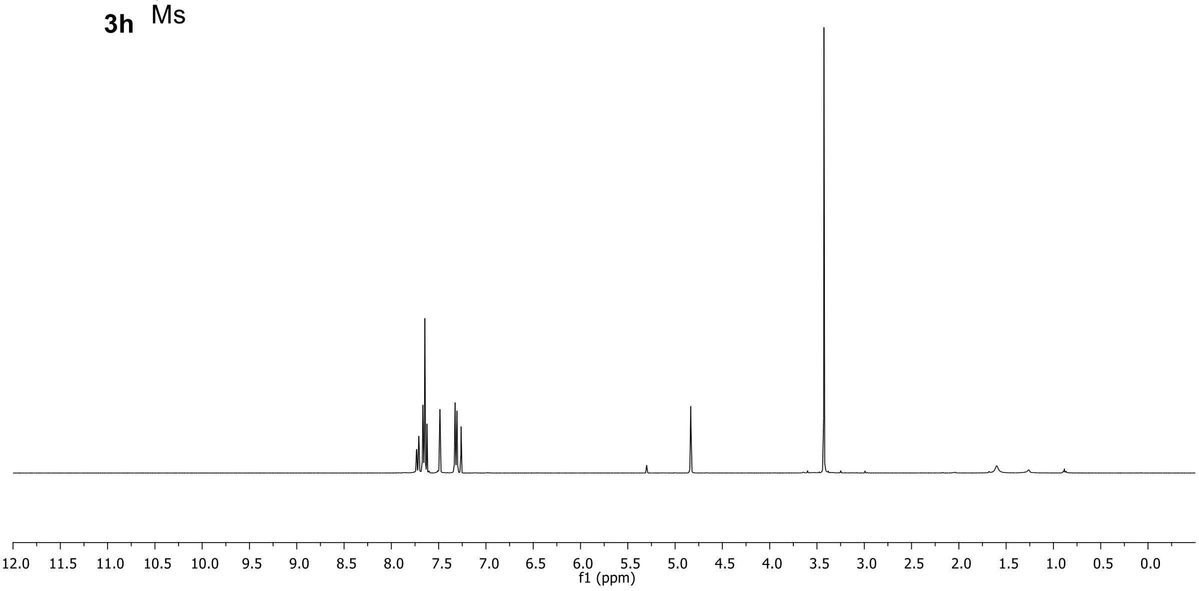
12.0 11.5 11.0 10.5 10.0 9.5 9.0 8.5 8.0 7.5 7.0 6.5 6.0 5.5 5.0 4.5 4.0 3.5 3.0 2.5 2.0 1.5 1.0 0.5 0.0
f1 (ppm)

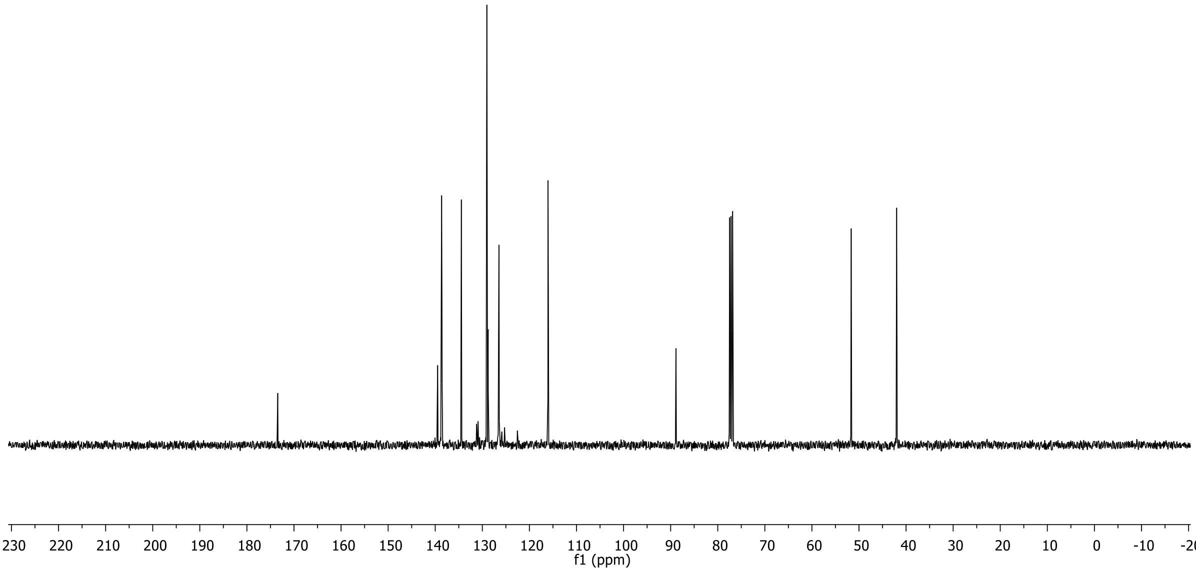
230 220 210 200 190 180 170 160 150 140 130 120 110 100 90 80 70 60 50 40 30 20 10 0 -10 -20
f1 (ppm)

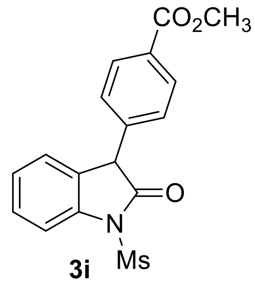

CO2CH3
O
N
Ms

3i

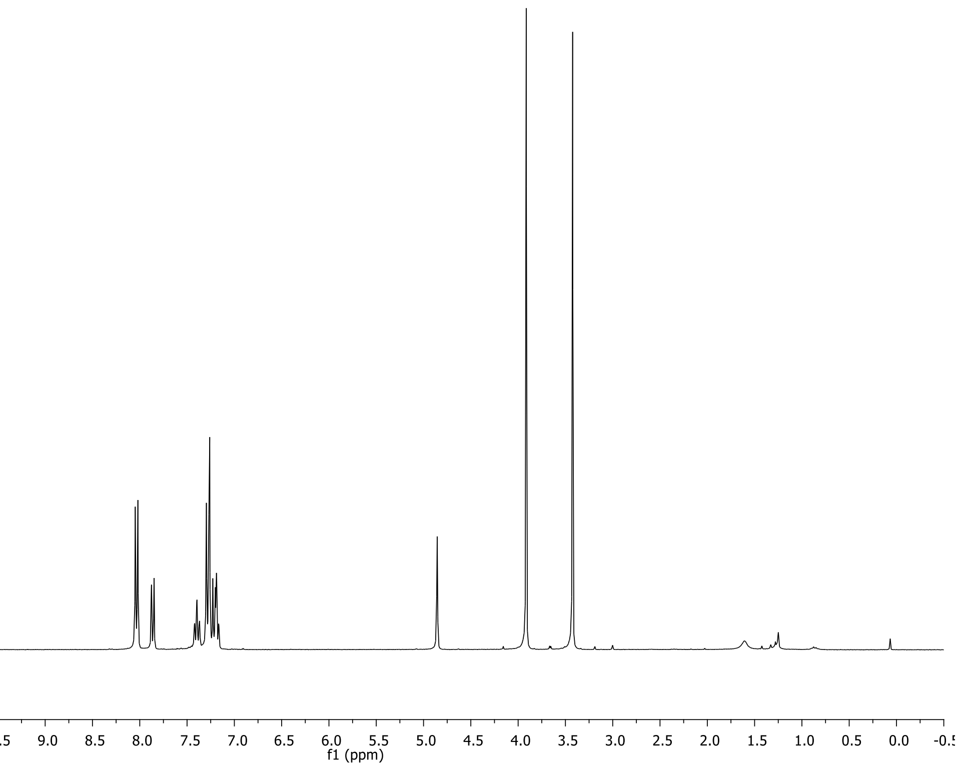

12.0 11.5 11.0 10.5 10.0 9.5 9.0 8.5 8.0 7.5 7.0 6.5 6.0 5.5 5.0 4.5 4.0 3.5 3.0 2.5 2.0 1.5 1.0 0.5 0.0
f1 (ppm)

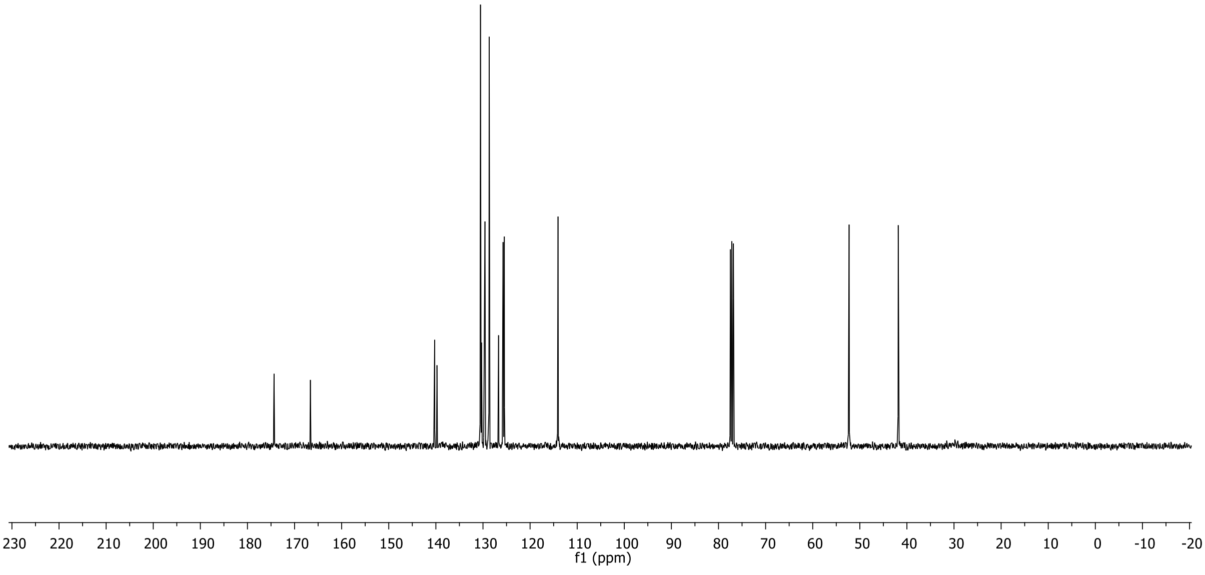

230 220 210 200 190 180 170 160 150 140 130 120 110 100 90 80 70 60 50 40 30 20 10 0 -10 -20
f1 (ppm)

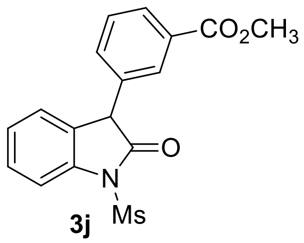
CO2CH3
O
N
Ms

3j

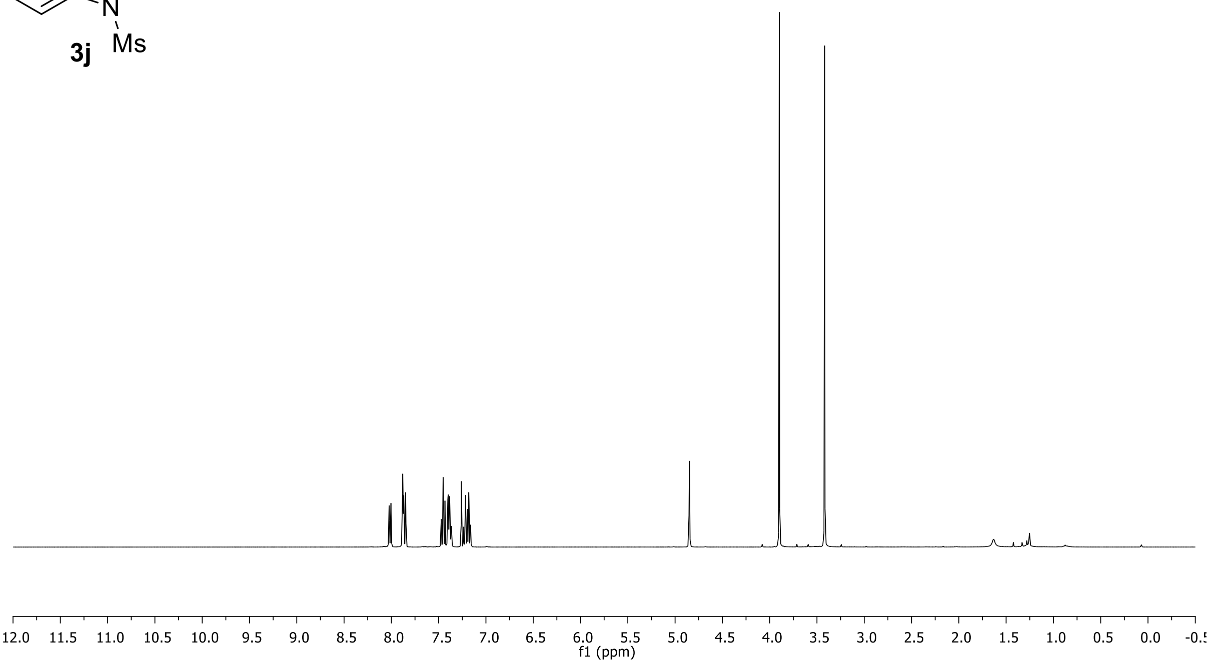
12.0 11.5 11.0 10.5 10.0 9.5 9.0 8.5 8.0 7.5 7.0 6.5 6.0 5.5 5.0 4.5 4.0 3.5 3.0 2.5 2.0 1.5 1.0 0.5 0.0
f1 (ppm)

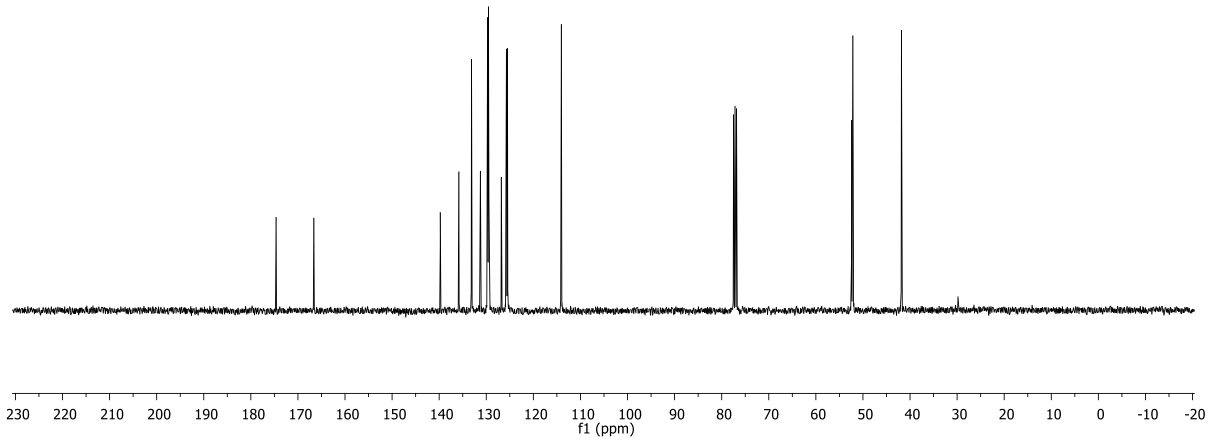
230 220 210 200 190 180 170 160 150 140 130 120 110 100 90 80 70 60 50 40 30 20 10 0 -10 -20
f1 (ppm)

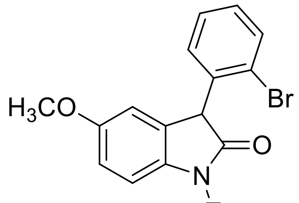

3k

$CDCl_3$, 23 °C

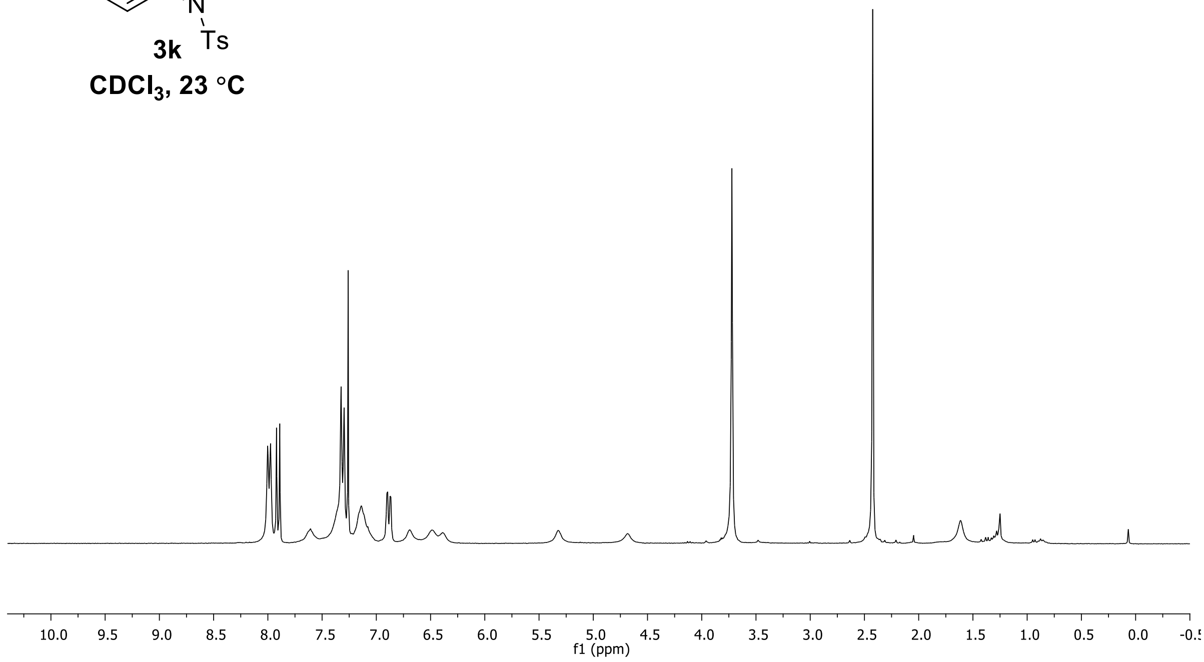

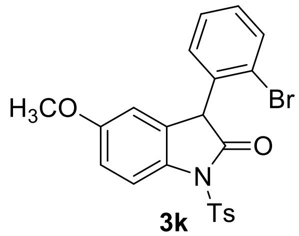

3k

$C_2Cl_4D_2$, 110 °C

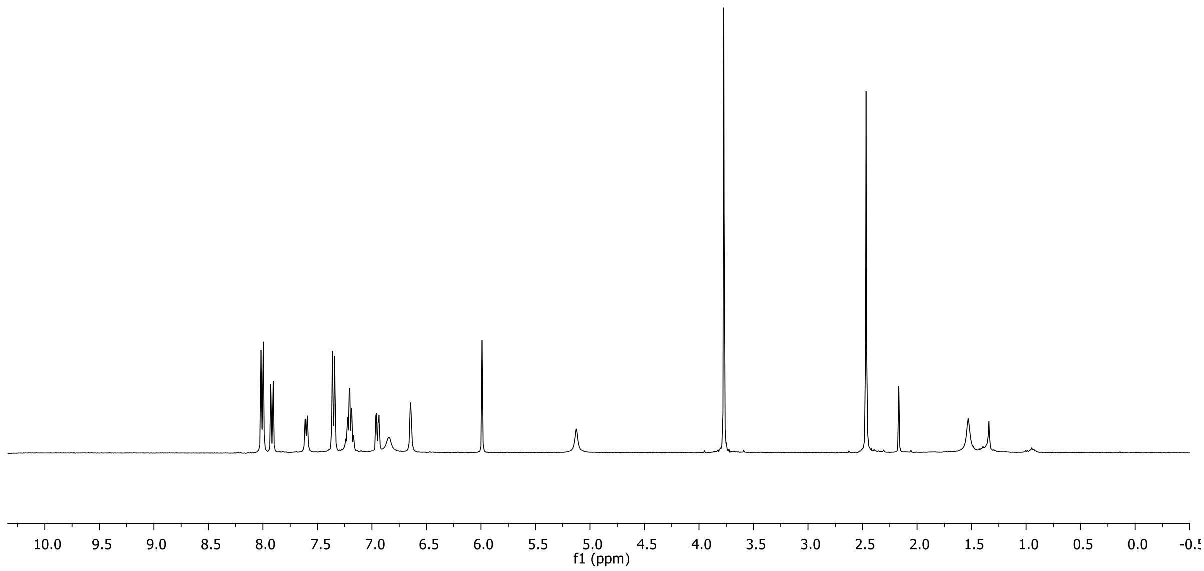

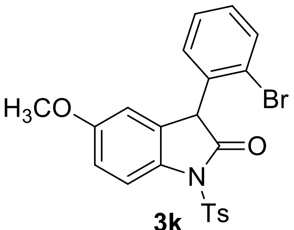

3k

$CDCl_3$, 23 °C

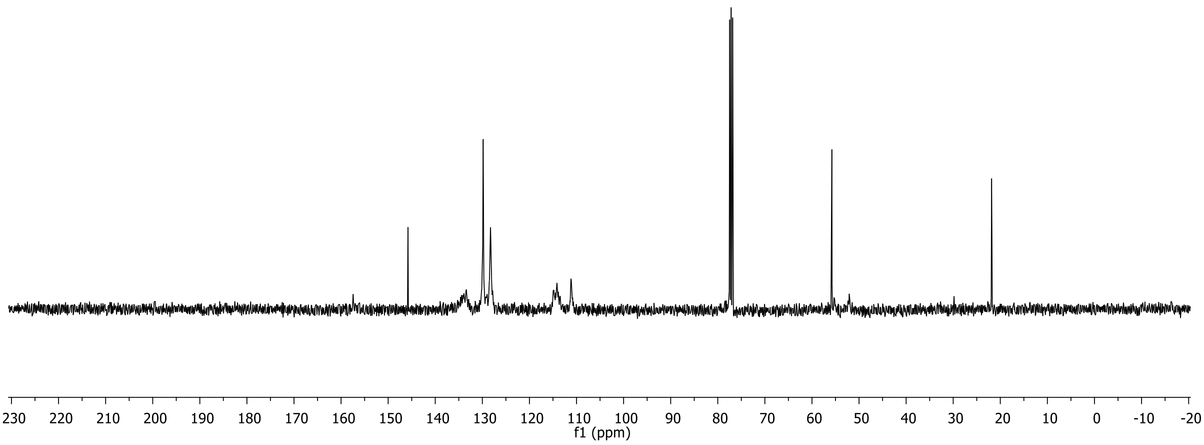

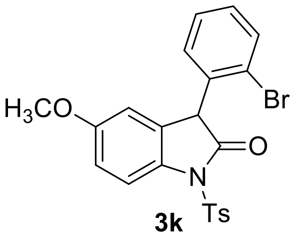

3k

$C_2Cl_4D_2$, 80 °C

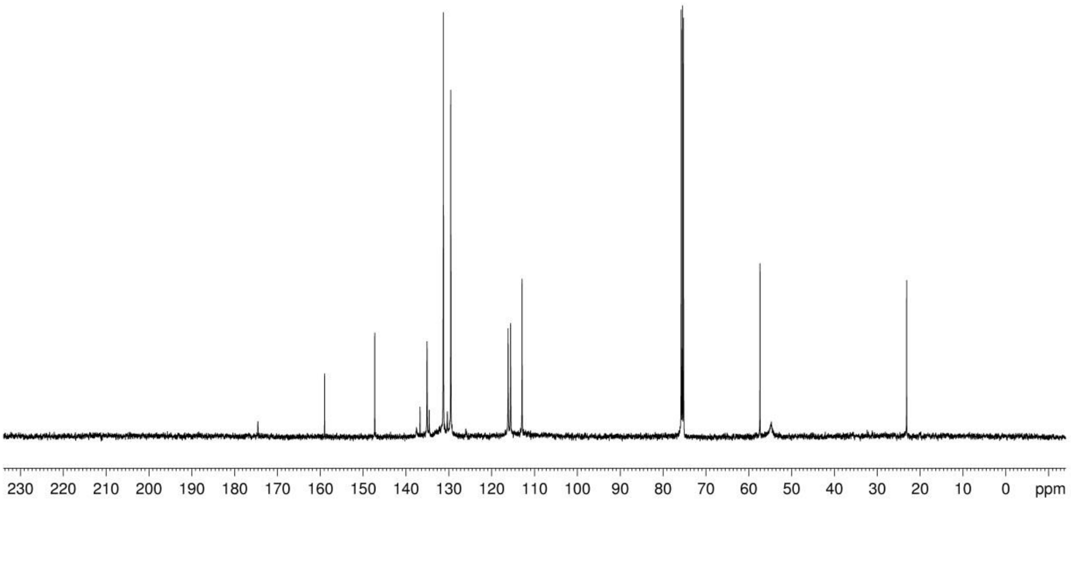

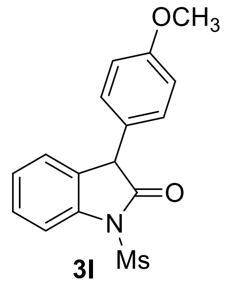
OCH3
O
N
Ms

3l

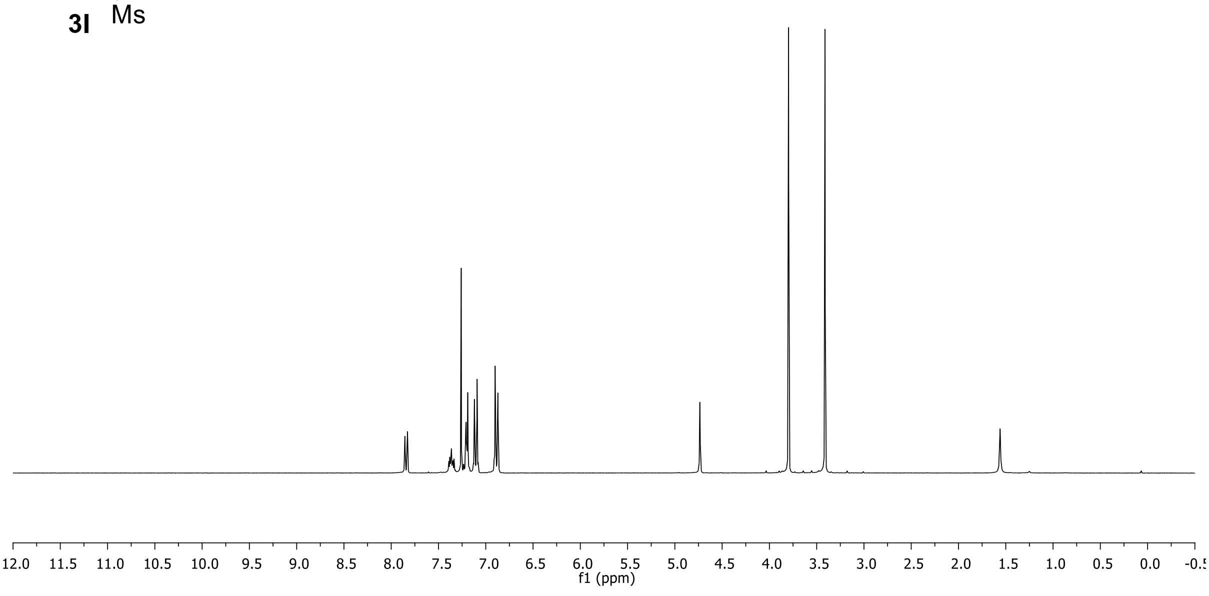
12.0 11.5 11.0 10.5 10.0 9.5 9.0 8.5 8.0 7.5 7.0 6.5 6.0 5.5 5.0 4.5 4.0 3.5 3.0 2.5 2.0 1.5 1.0 0.5 0.0
f1 (ppm)

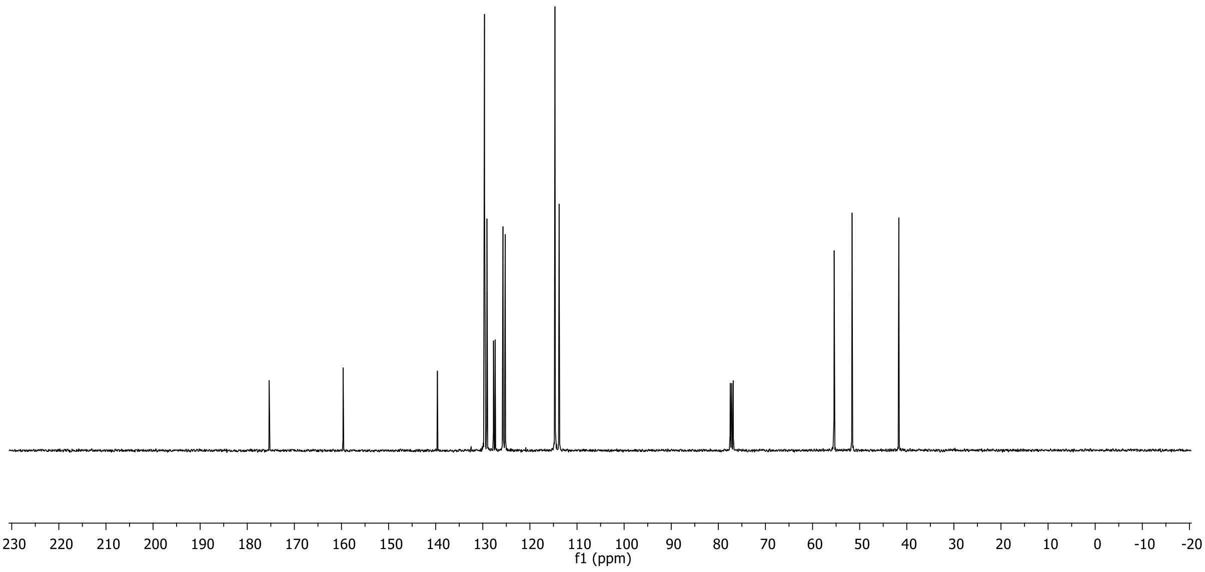
230 220 210 200 190 180 170 160 150 140 130 120 110 100 90 80 70 60 50 40 30 20 10 0 -10 -20
f1 (ppm)

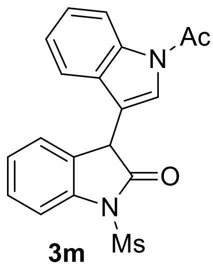
Ac
O
N
Ms

3m

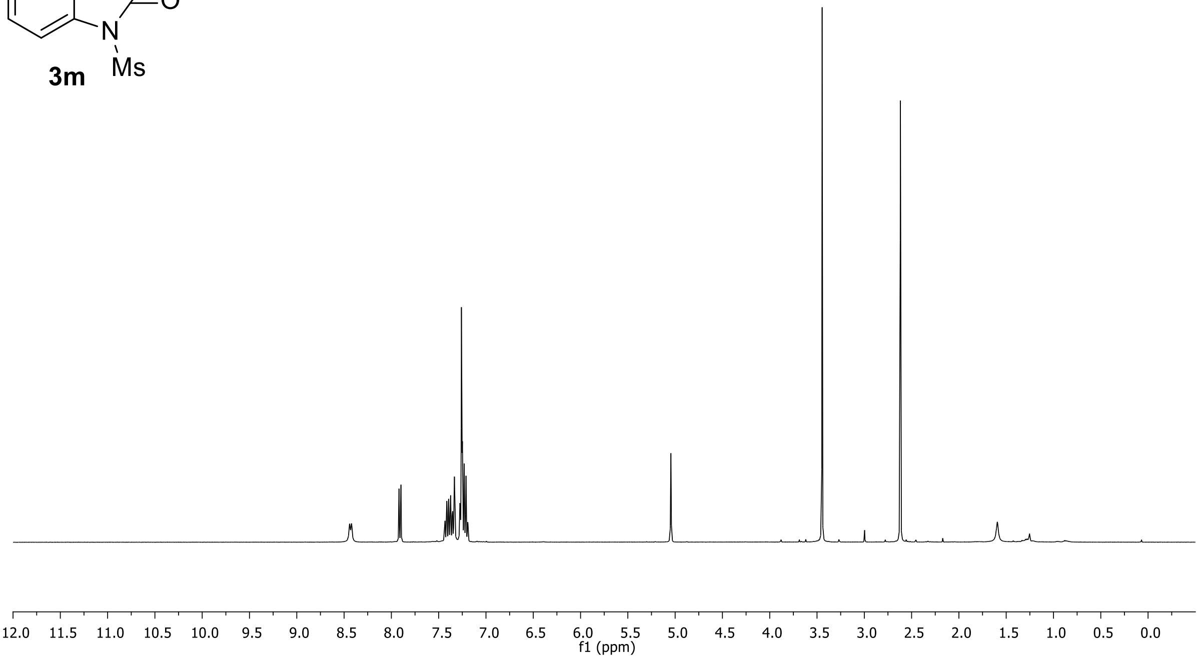
12.0 11.5 11.0 10.5 10.0 9.5 9.0 8.5 8.0 7.5 7.0 6.5 6.0 5.5 5.0 4.5 4.0 3.5 3.0 2.5 2.0 1.5 1.0 0.5 0.0
f1 (ppm)

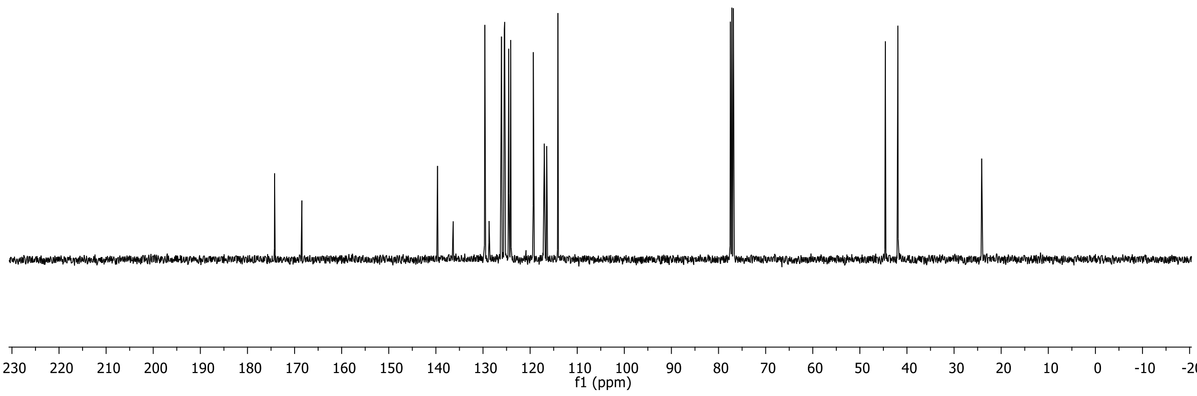
230 220 210 200 190 180 170 160 150 140 130 120 110 100 90 80 70 60 50 40 30 20 10 0 -10 -20
f1 (ppm)

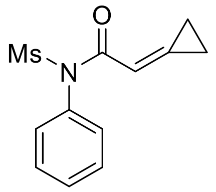
O
Ms
N

4

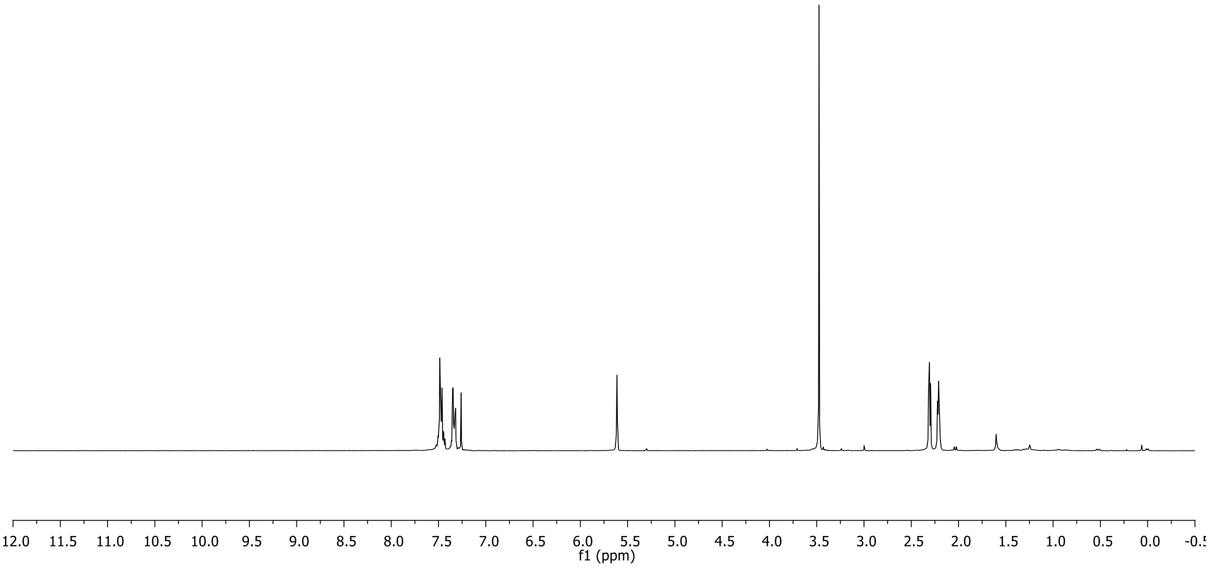
12.0 11.5 11.0 10.5 10.0 9.5 9.0 8.5 8.0 7.5 7.0 6.5 6.0 5.5 5.0 4.5 4.0 3.5 3.0 2.5 2.0 1.5 1.0 0.5 0.0
f1 (ppm)

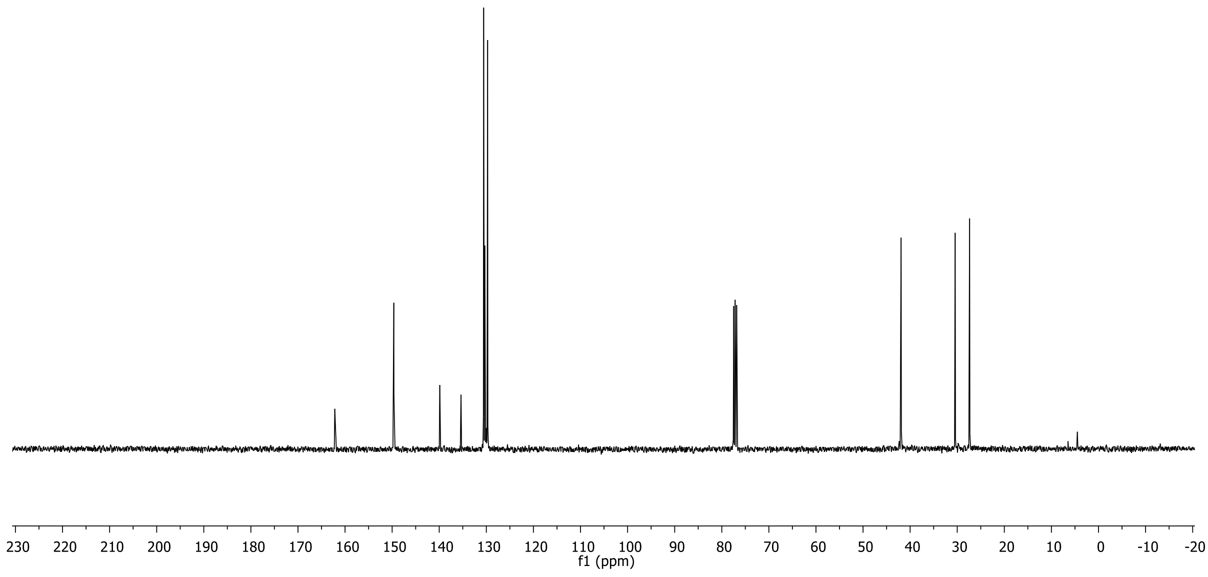
230 220 210 200 190 180 170 160 150 140 130 120 110 100 90 80 70 60 50 40 30 20 10 0 -10 -20
f1 (ppm)

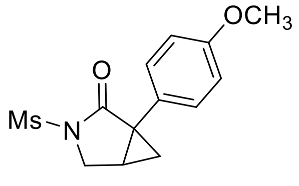

OCH3
O
Ms
N

6

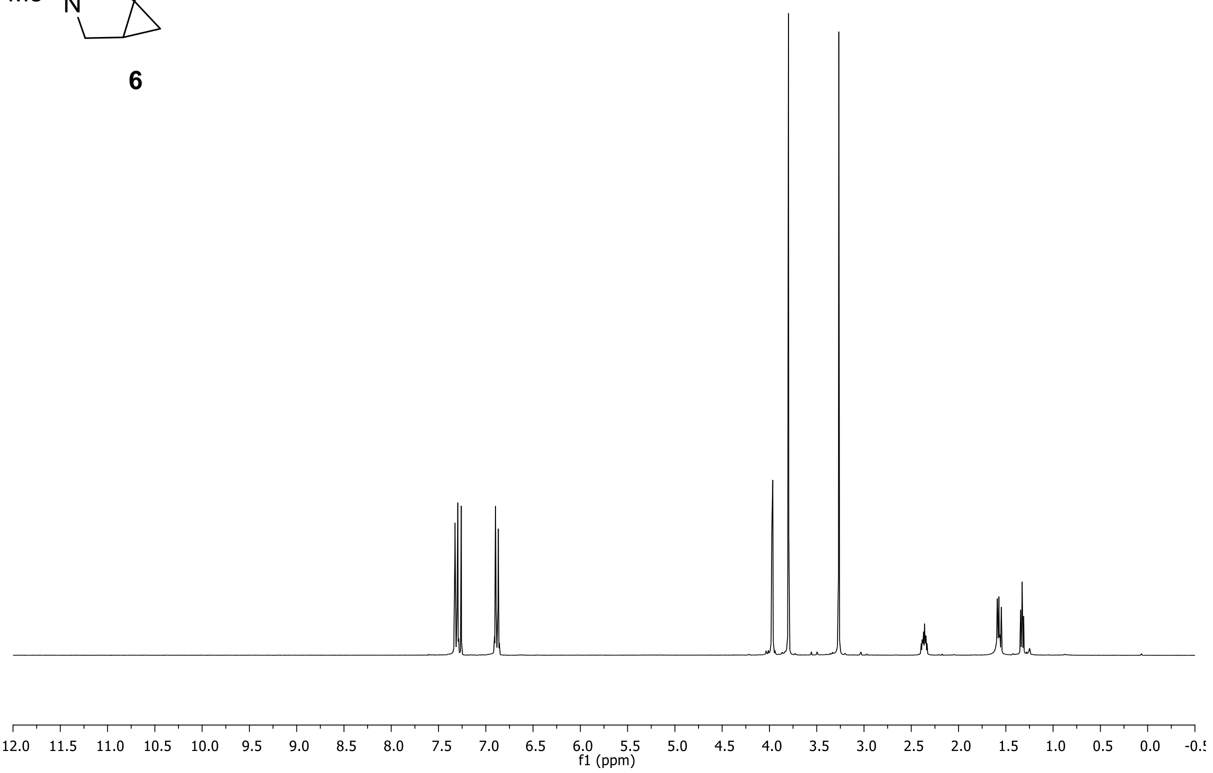

12.0 11.5 11.0 10.5 10.0 9.5 9.0 8.5 8.0 7.5 7.0 6.5 6.0 5.5 5.0 4.5 4.0 3.5 3.0 2.5 2.0 1.5 1.0 0.5 0.0
f1 (ppm)

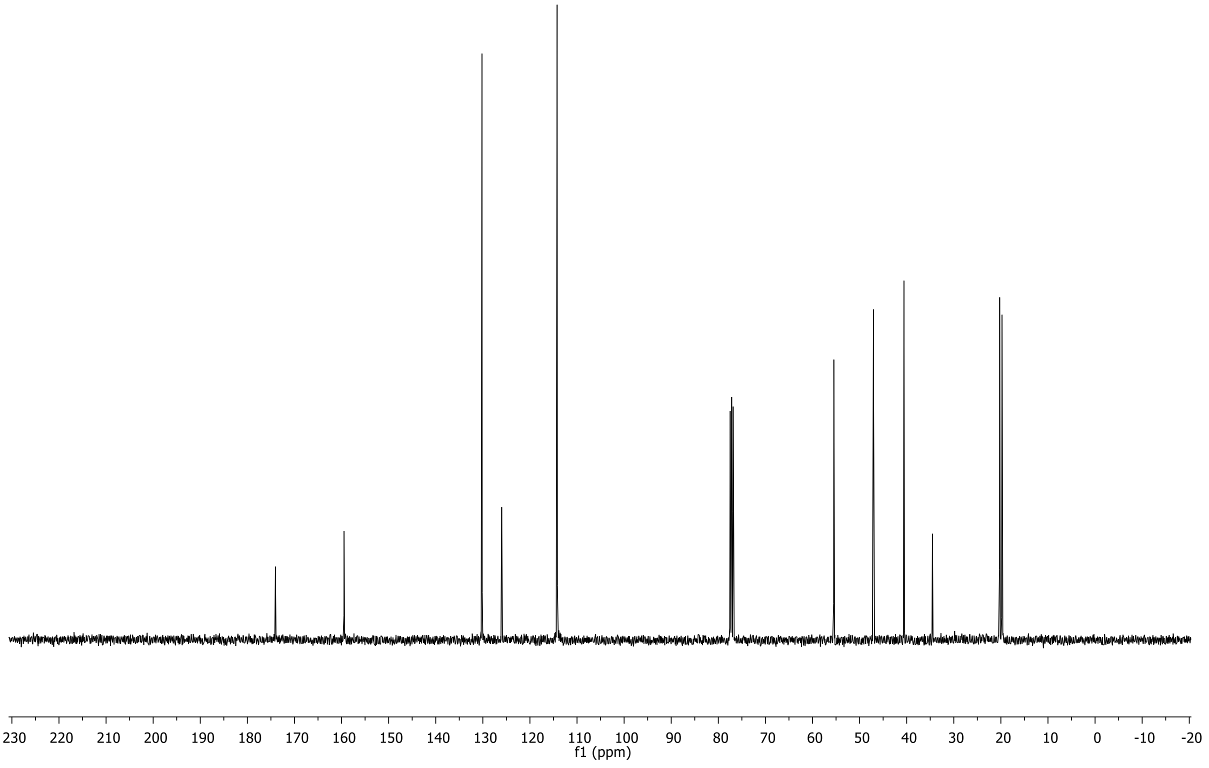

230 220 210 200 190 180 170 160 150 140 130 120 110 100 90 80 70 60 50 40 30 20 10 0 -10 -20
f1 (ppm)

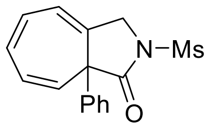
N−Ms
Ph
O

S1

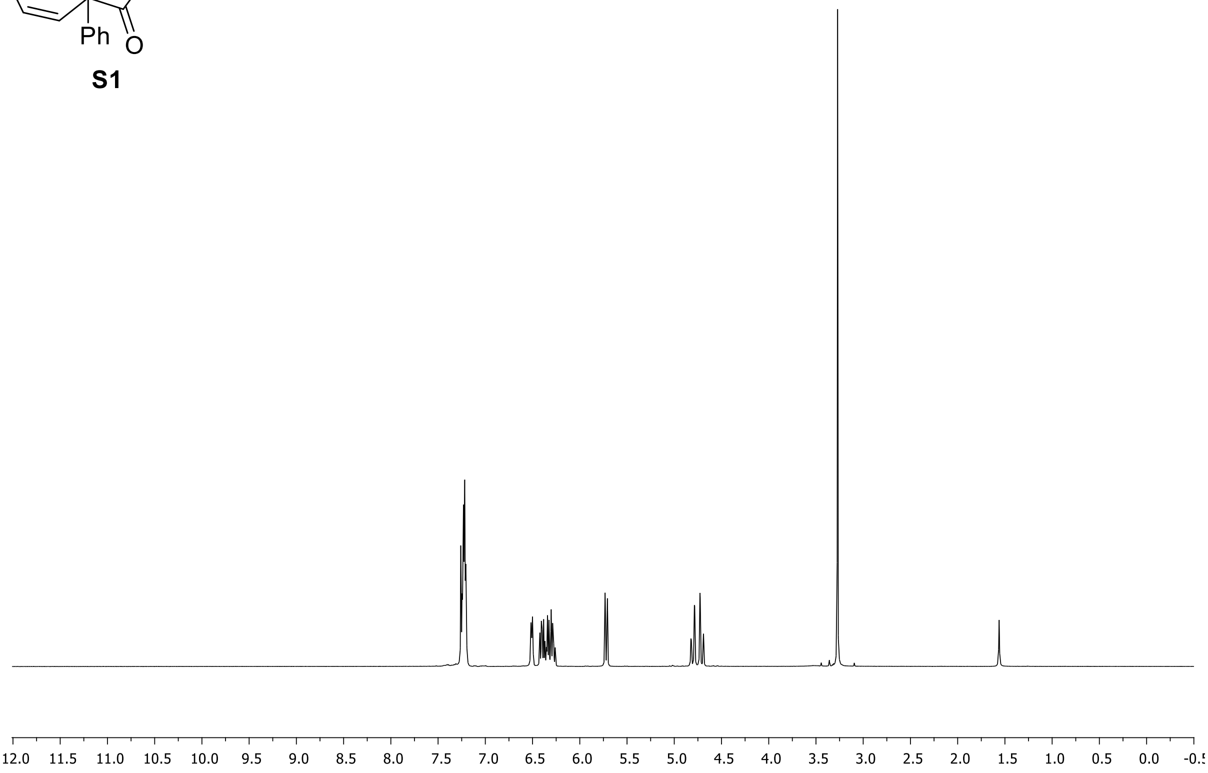
12.0 11.5 11.0 10.5 10.0 9.5 9.0 8.5 8.0 7.5 7.0 6.5 6.0 5.5 5.0 4.5 4.0 3.5 3.0 2.5 2.0 1.5 1.0 0.5 0.0 -0.5

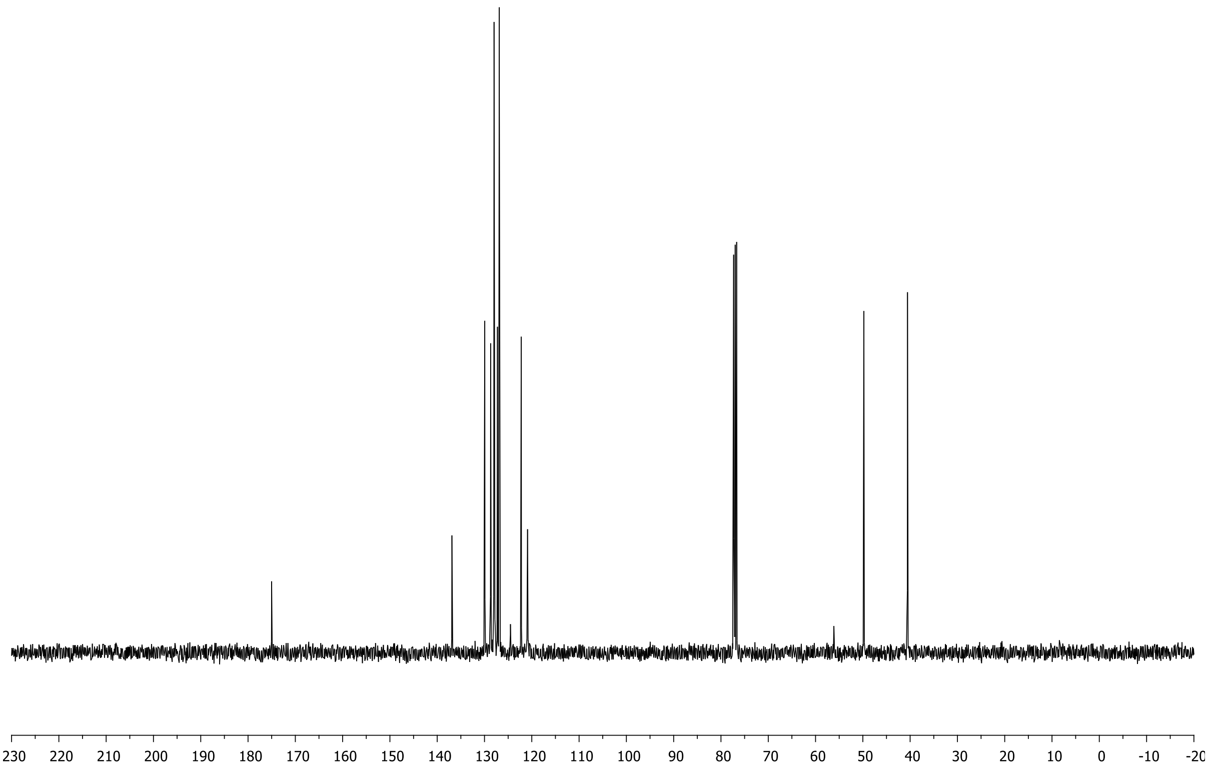
230 220 210 200 190 180 170 160 150 140 130 120 110 100 90 80 70 60 50 40 30 20 10 0 -10 -20

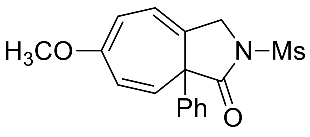

8a
$CDCl_3$, 22 °C

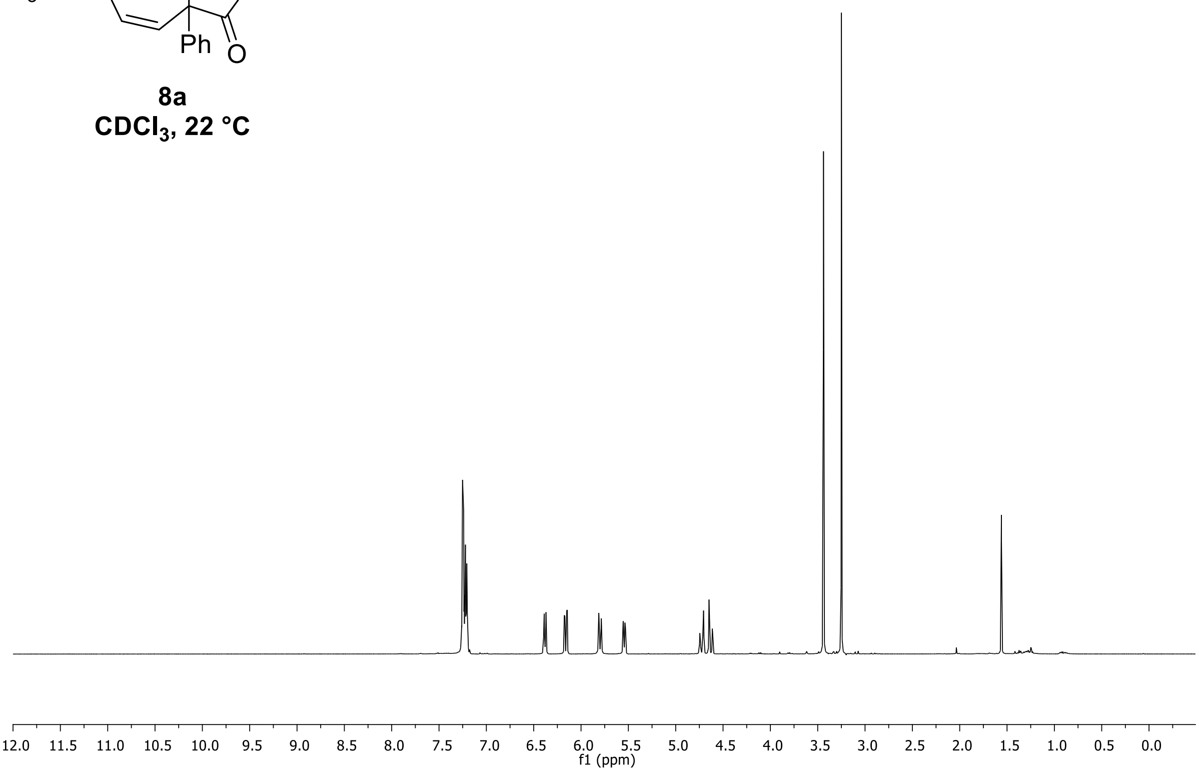

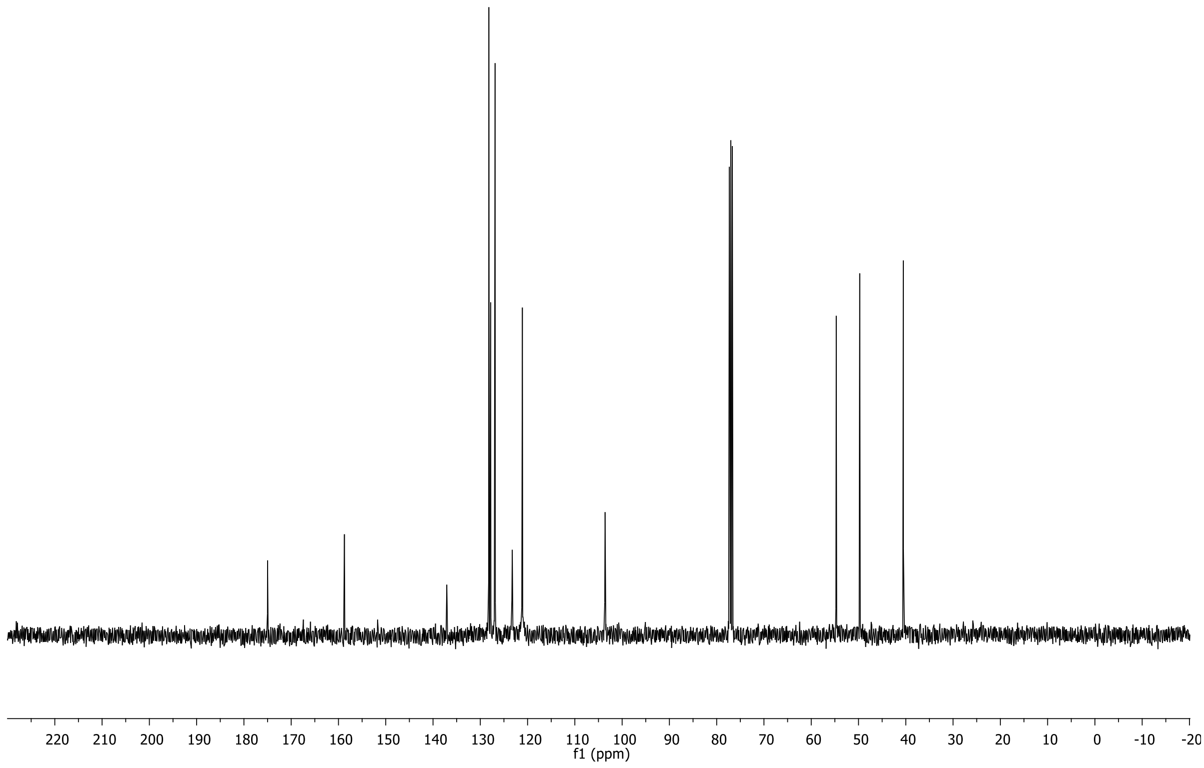

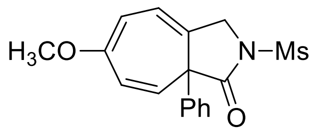

8a
$C_2Cl_4D_2$, 18 °C

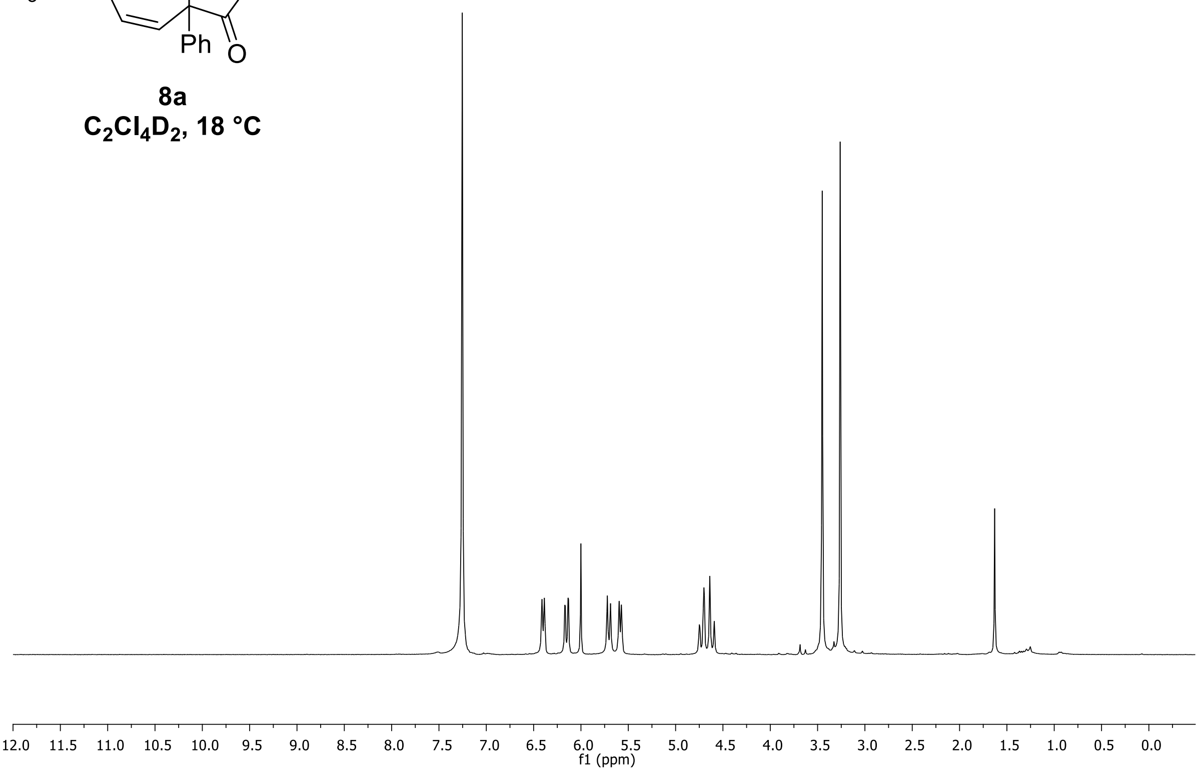

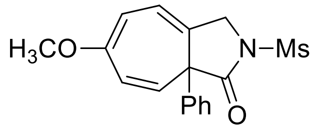

8a
$C_2Cl_4D_2$, 80 °C

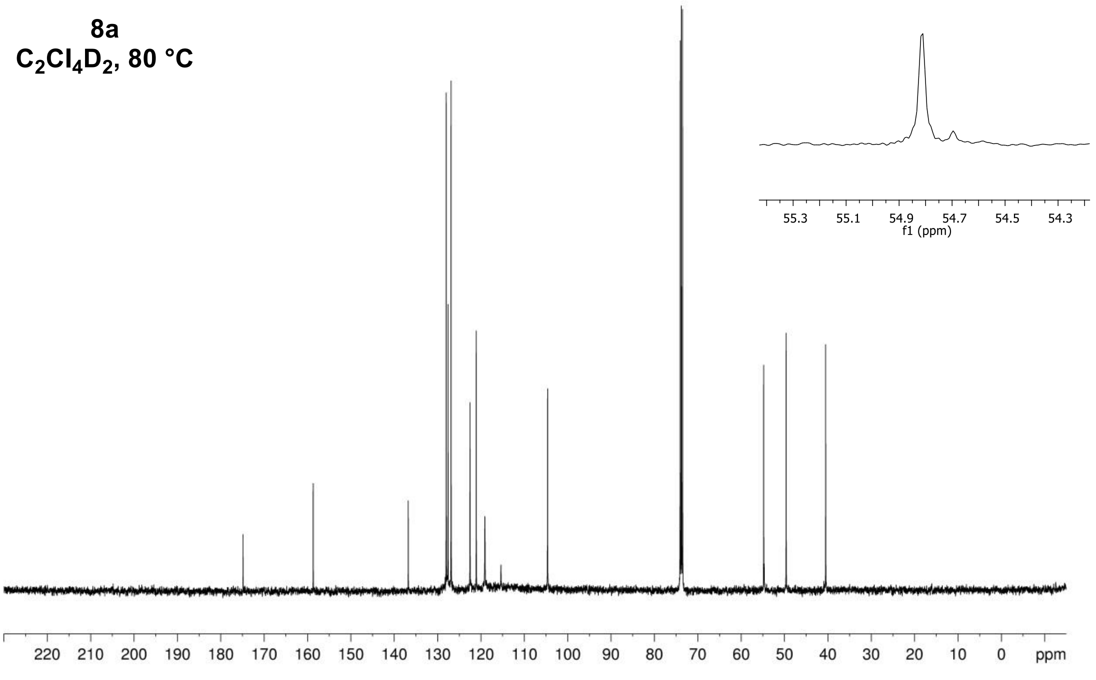

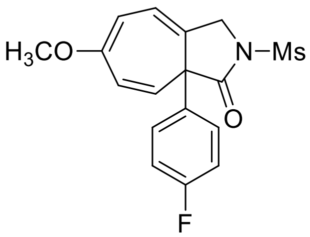
H3CO
N–Ms
O
F

8b

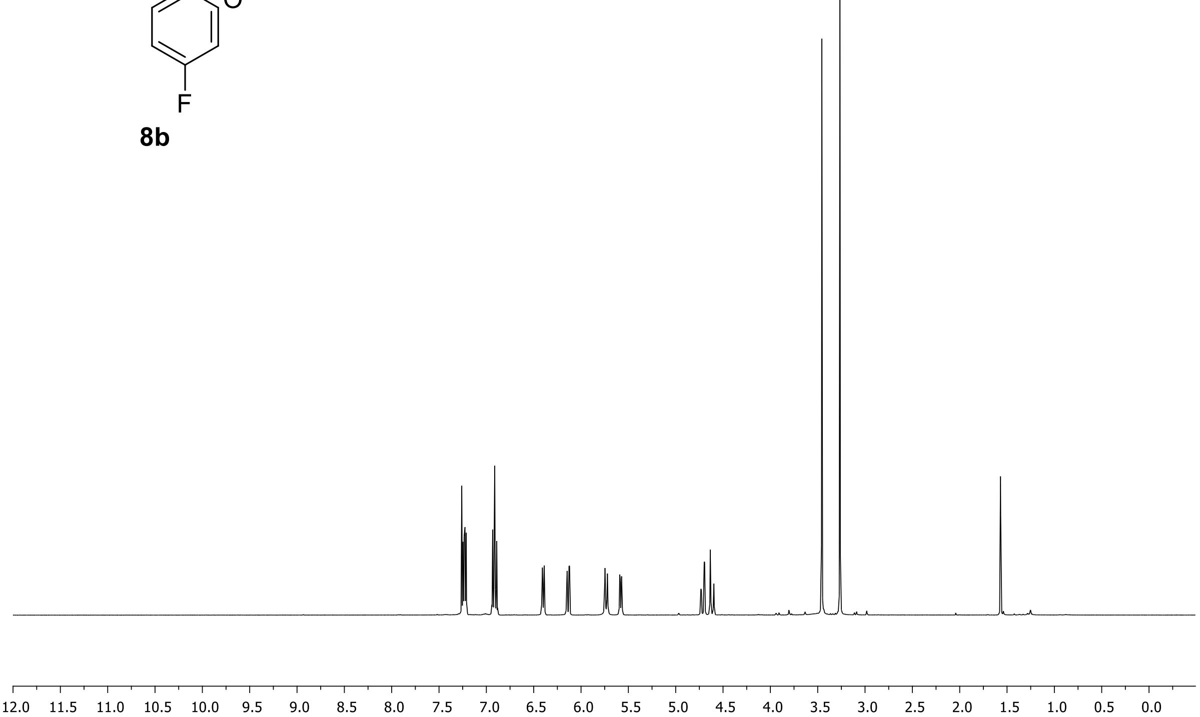
12.0 11.5 11.0 10.5 10.0 9.5 9.0 8.5 8.0 7.5 7.0 6.5 6.0 5.5 5.0 4.5 4.0 3.5 3.0 2.5 2.0 1.5 1.0 0.5 0.0

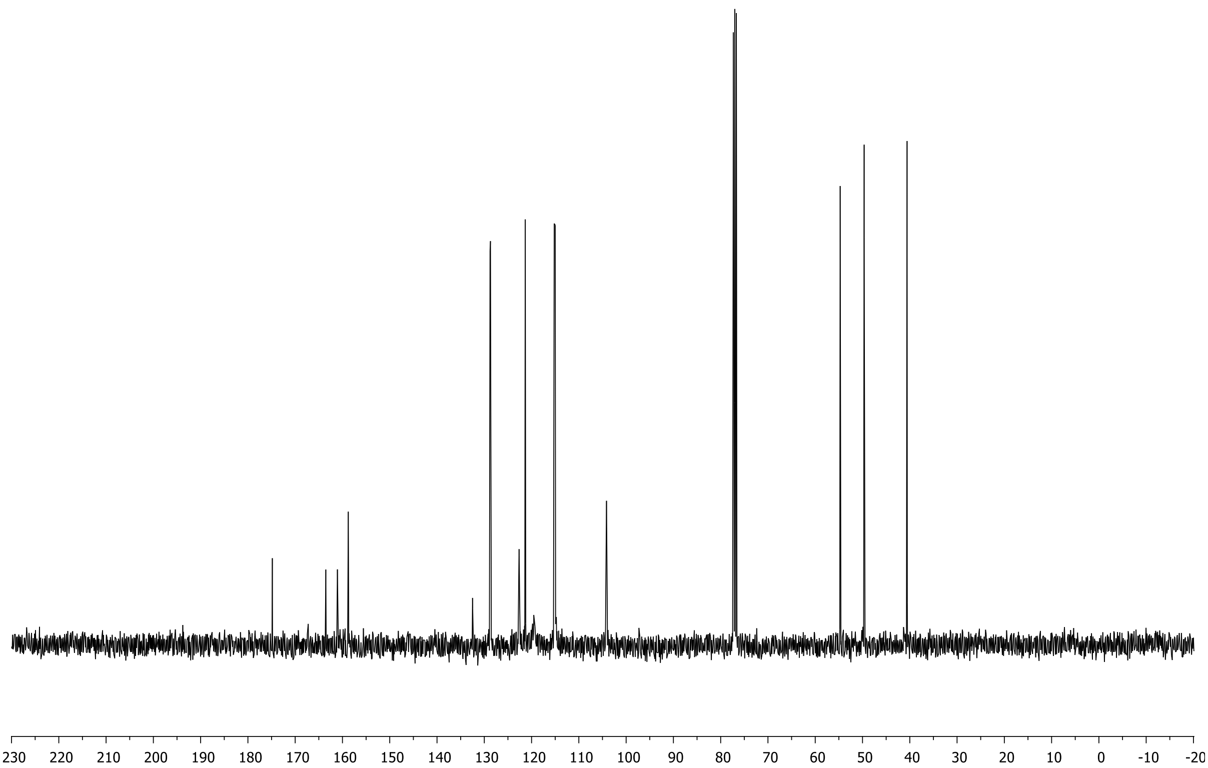
230 220 210 200 190 180 170 160 150 140 130 120 110 100 90 80 70 60 50 40 30 20 10 0 -10 -20

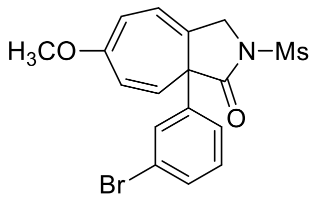
H3CO
N–Ms
O
Br

8c

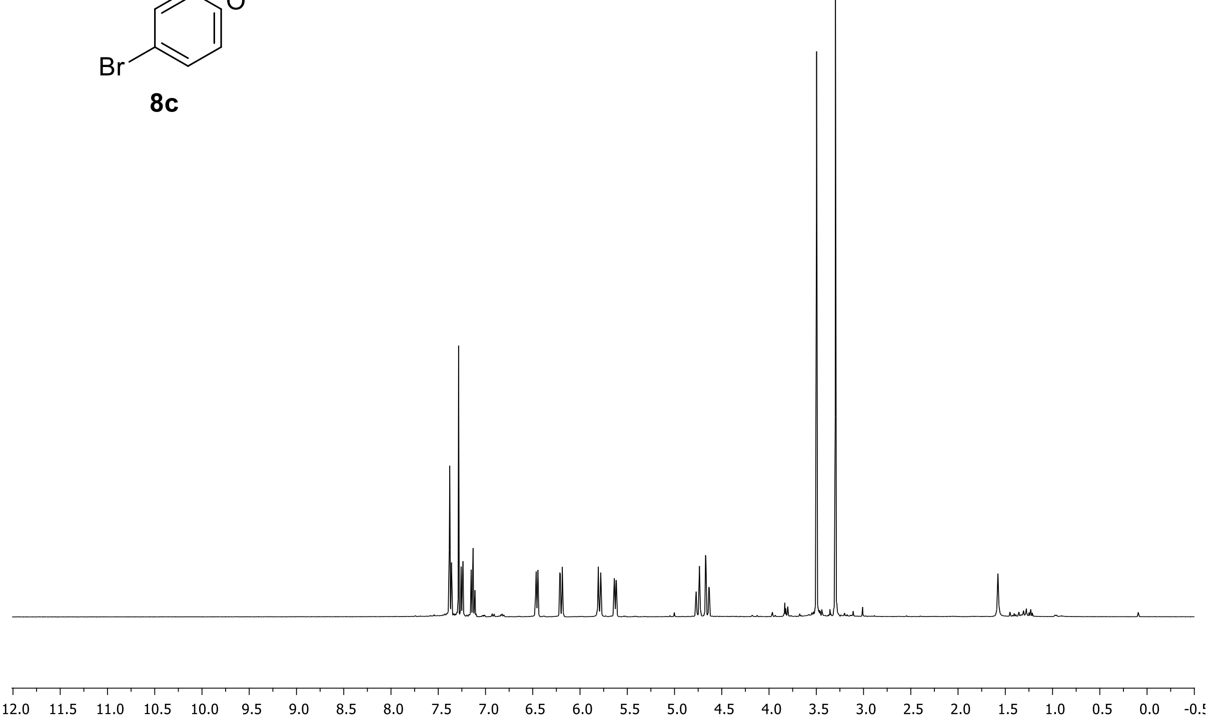
12.0 11.5 11.0 10.5 10.0 9.5 9.0 8.5 8.0 7.5 7.0 6.5 6.0 5.5 5.0 4.5 4.0 3.5 3.0 2.5 2.0 1.5 1.0 0.5 0.0 -0.5

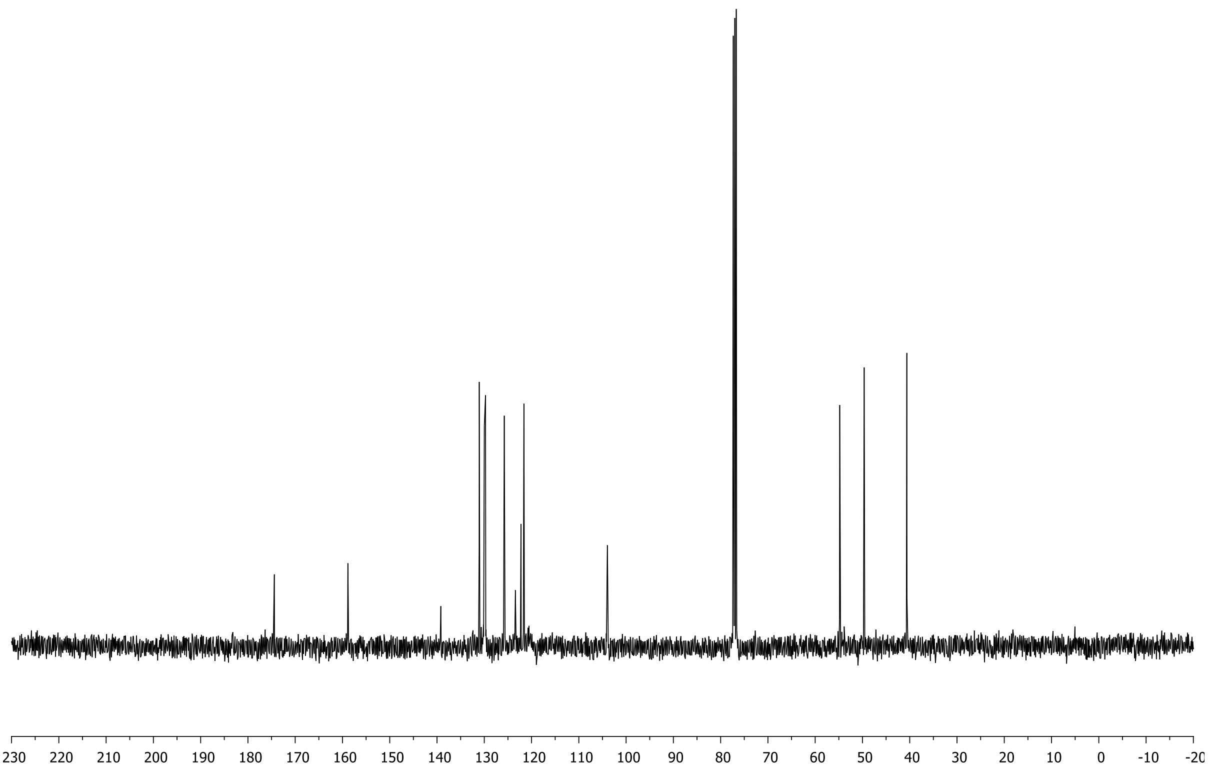
230 220 210 200 190 180 170 160 150 140 130 120 110 100 90 80 70 60 50 40 30 20 10 0 -10 -20

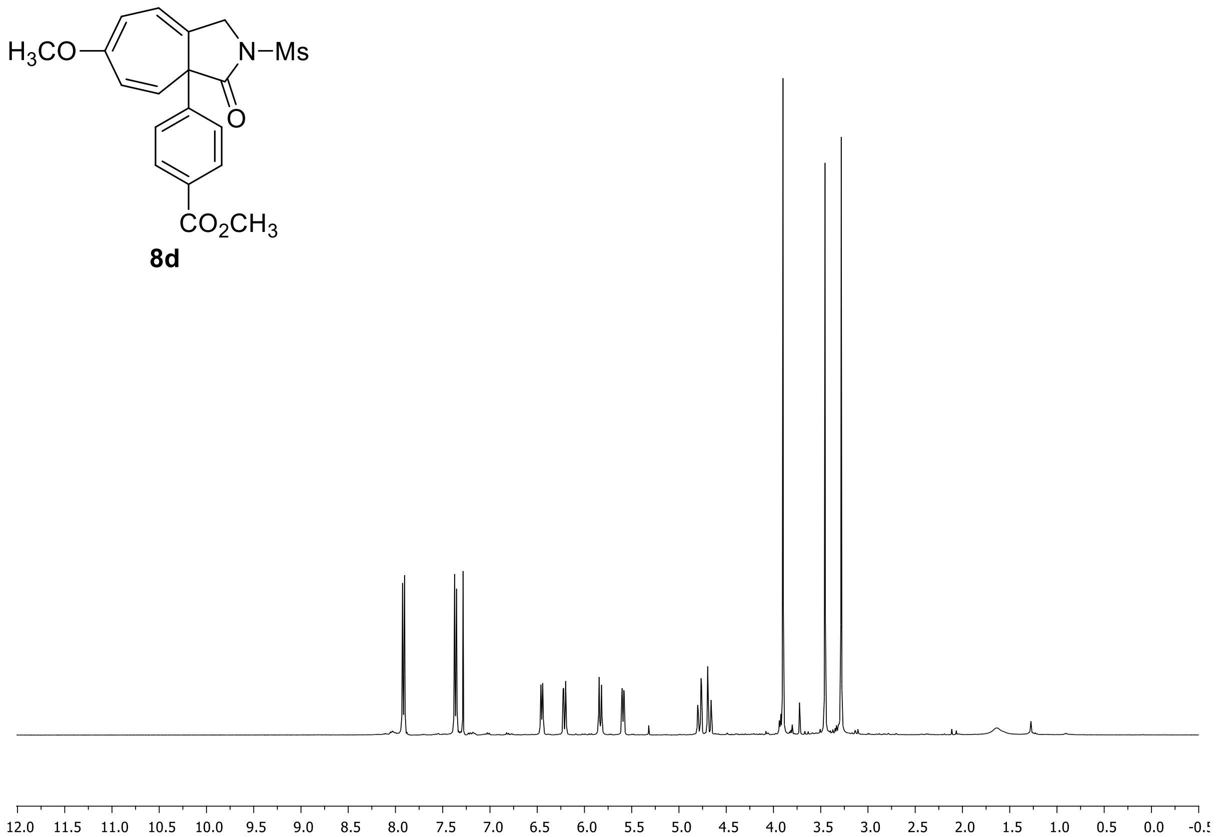
H3CO
N−Ms
O
CO2CH3

8d

12.0 11.5 11.0 10.5 10.0 9.5 9.0 8.5 8.0 7.5 7.0 6.5 6.0 5.5 5.0 4.5 4.0 3.5 3.0 2.5 2.0 1.5 1.0 0.5 0.0 -0.5

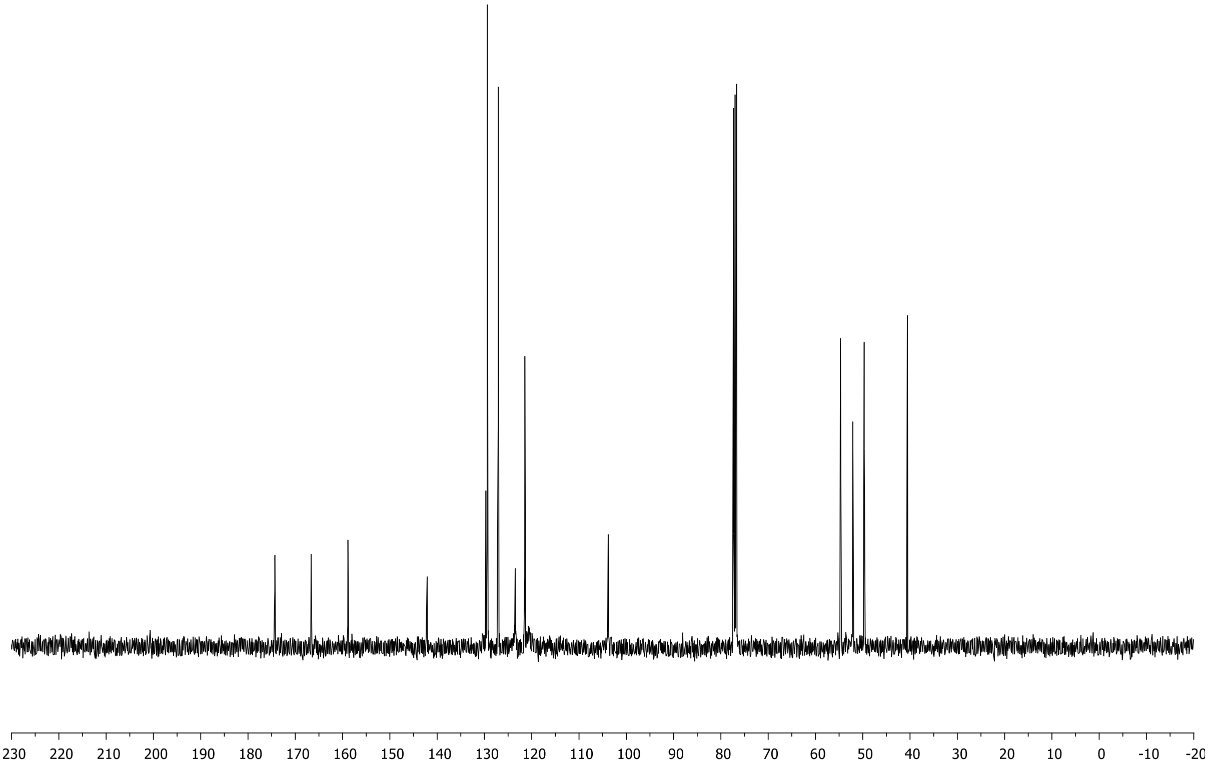
230 220 210 200 190 180 170 160 150 140 130 120 110 100 90 80 70 60 50 40 30 20 10 0 -10 -20

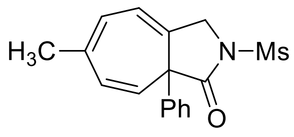
H3C
N–Ms
Ph
O

8f

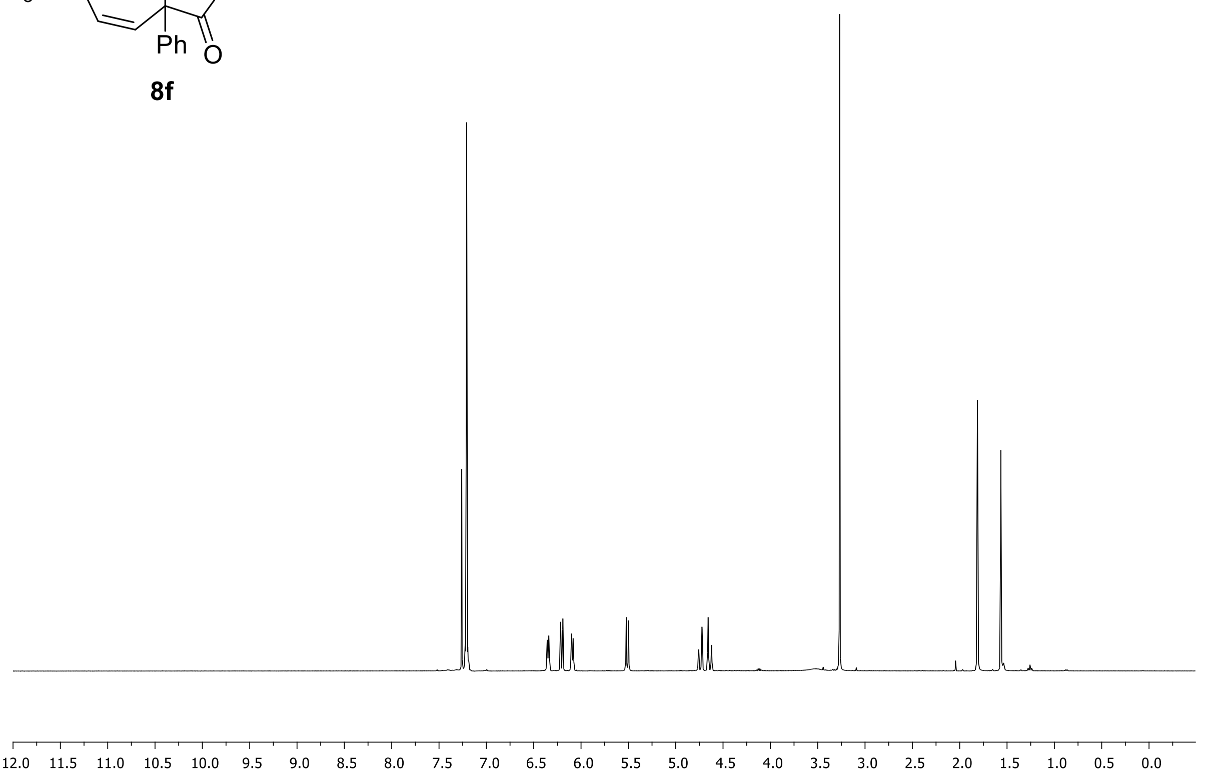
12.0 11.5 11.0 10.5 10.0 9.5 9.0 8.5 8.0 7.5 7.0 6.5 6.0 5.5 5.0 4.5 4.0 3.5 3.0 2.5 2.0 1.5 1.0 0.5 0.0

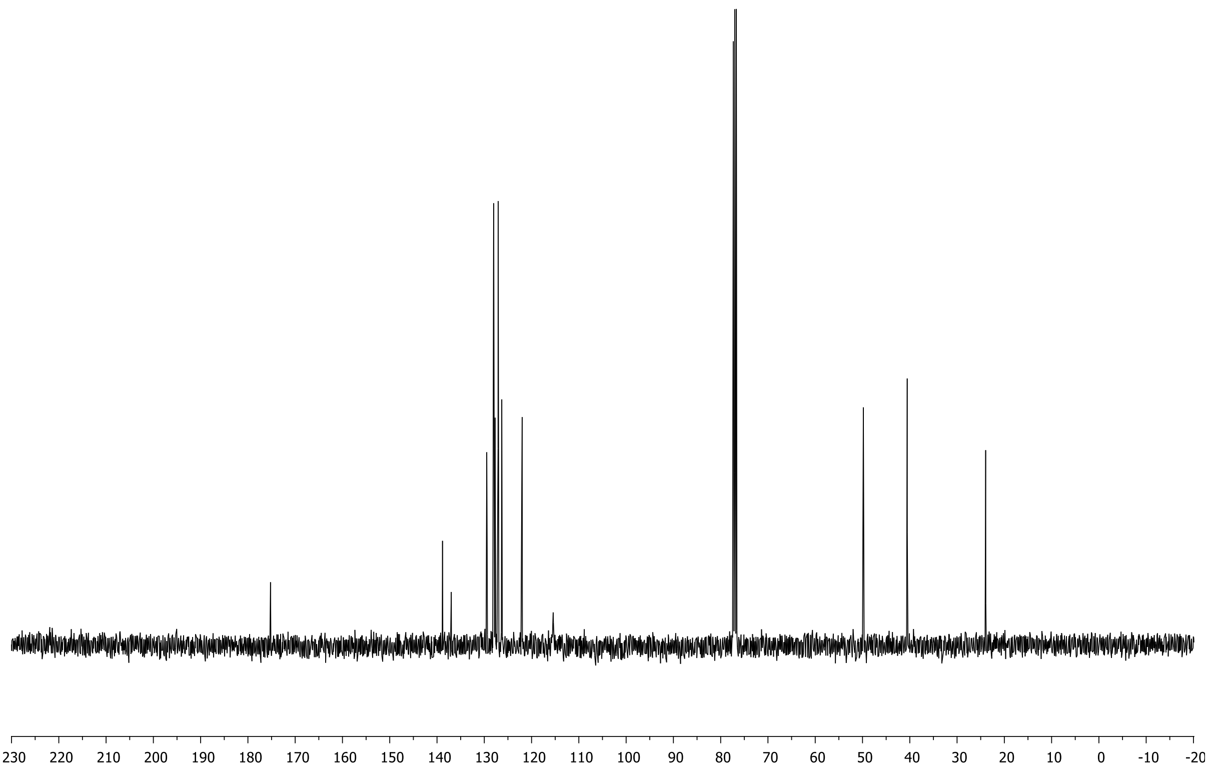
230 220 210 200 190 180 170 160 150 140 130 120 110 100 90 80 70 60 50 40 30 20 10 0 -10 -20

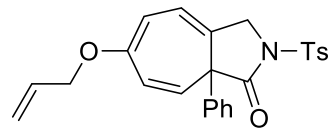
N–Ts
O
Ph
O

8g

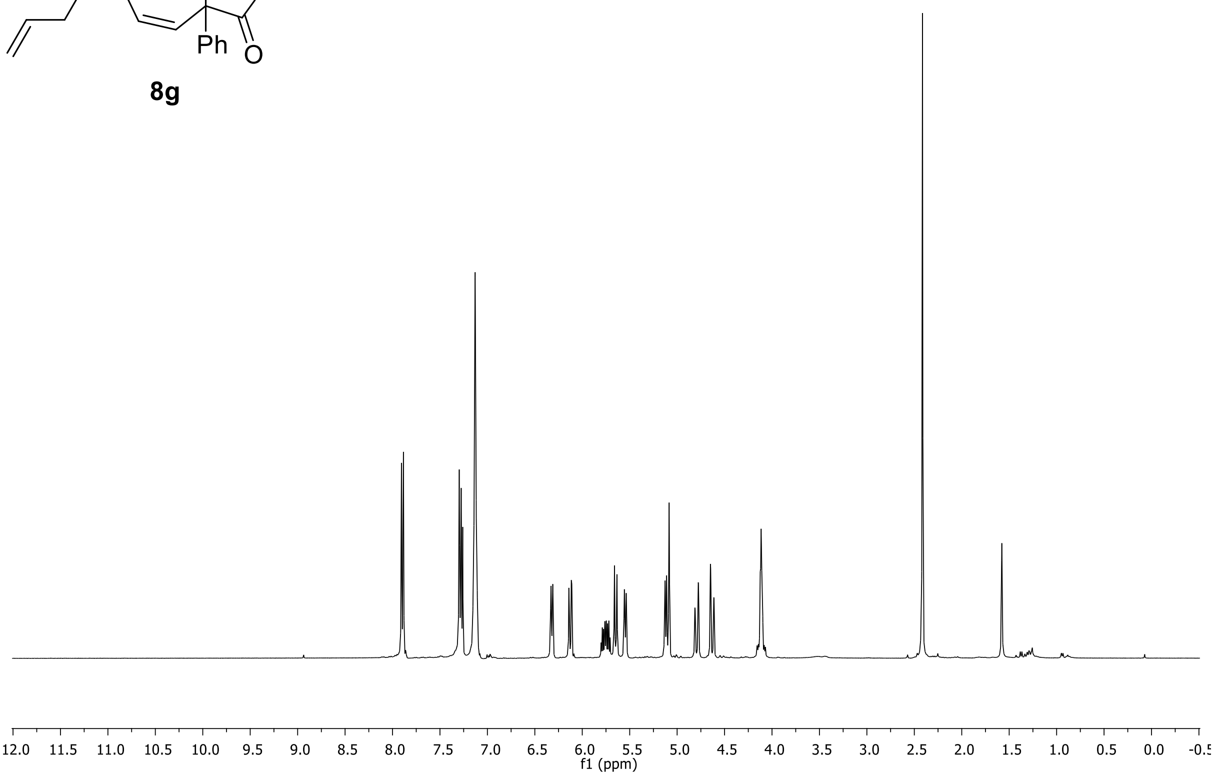
12.0 11.5 11.0 10.5 10.0 9.5 9.0 8.5 8.0 7.5 7.0 6.5 6.0 5.5 5.0 4.5 4.0 3.5 3.0 2.5 2.0 1.5 1.0 0.5 0.0 -0.5
f1 (ppm)

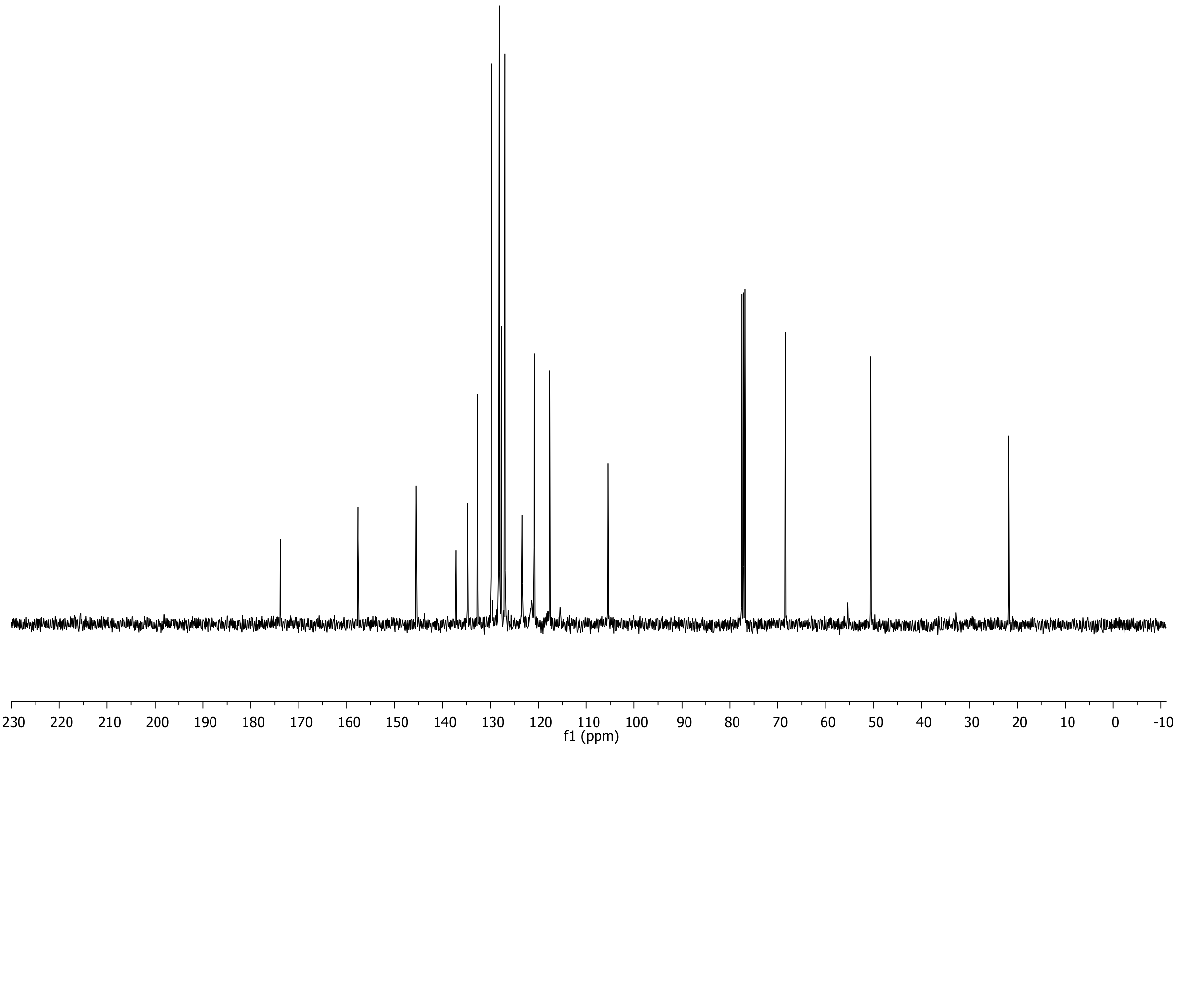
230 220 210 200 190 180 170 160 150 140 130 120 110 100 90 80 70 60 50 40 30 20 10 0 -10
f1 (ppm)

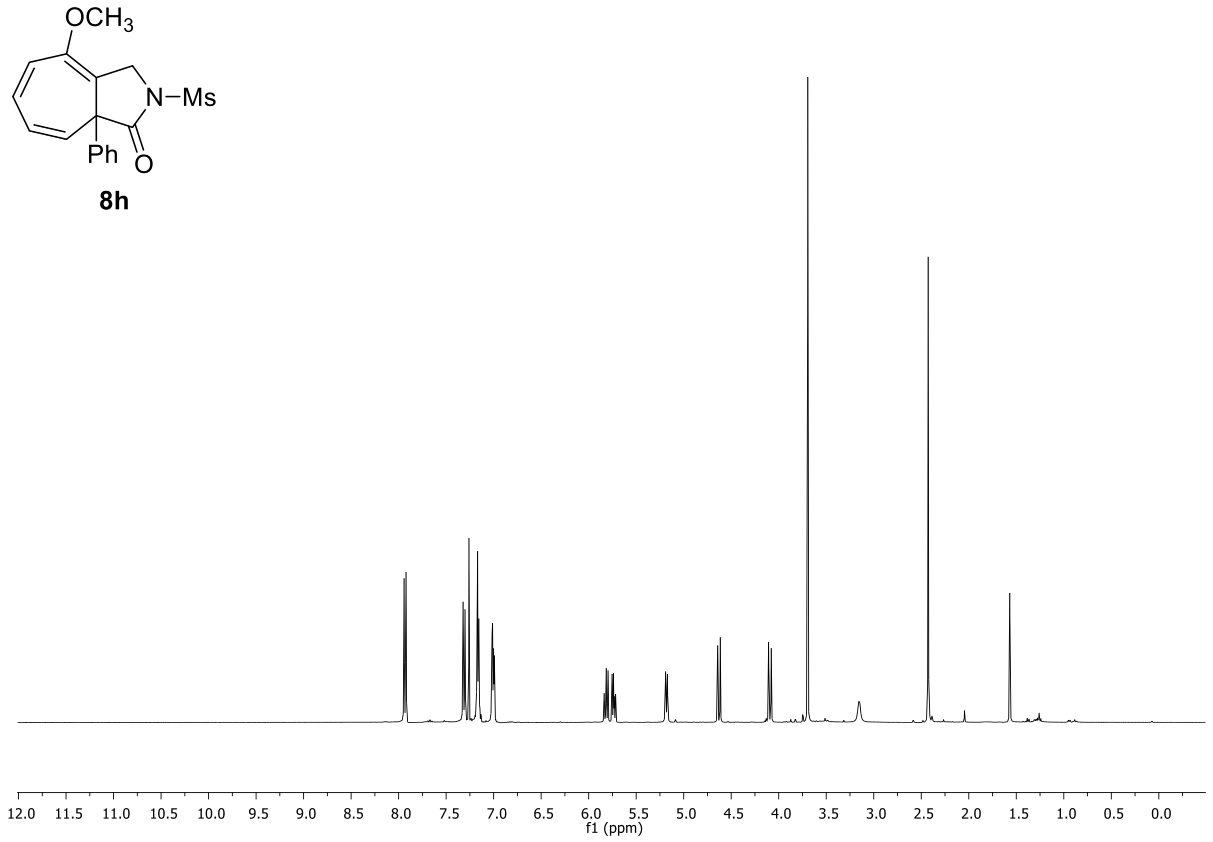
OCH3
N−Ms
Ph
O
8h
12.0 11.5 11.0 10.5 10.0 9.5 9.0 8.5 8.0 7.5 7.0 6.5 6.0 5.5 5.0 4.5 4.0 3.5 3.0 2.5 2.0 1.5 1.0 0.5 0.0
f1 (ppm)

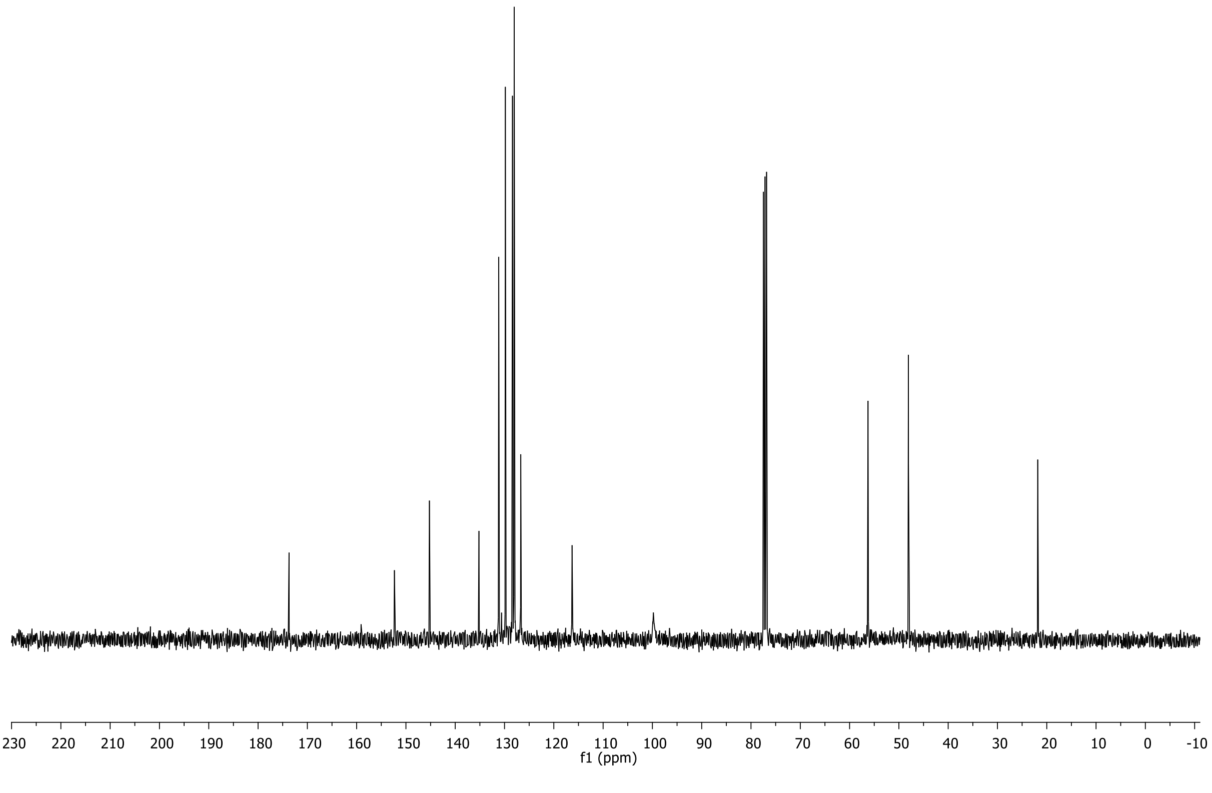
230 220 210 200 190 180 170 160 150 140 130 120 110 100 90 80 70 60 50 40 30 20 10 0 -10
f1 (ppm)

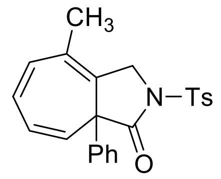
CH3
N−Ts
Ph
O

8i

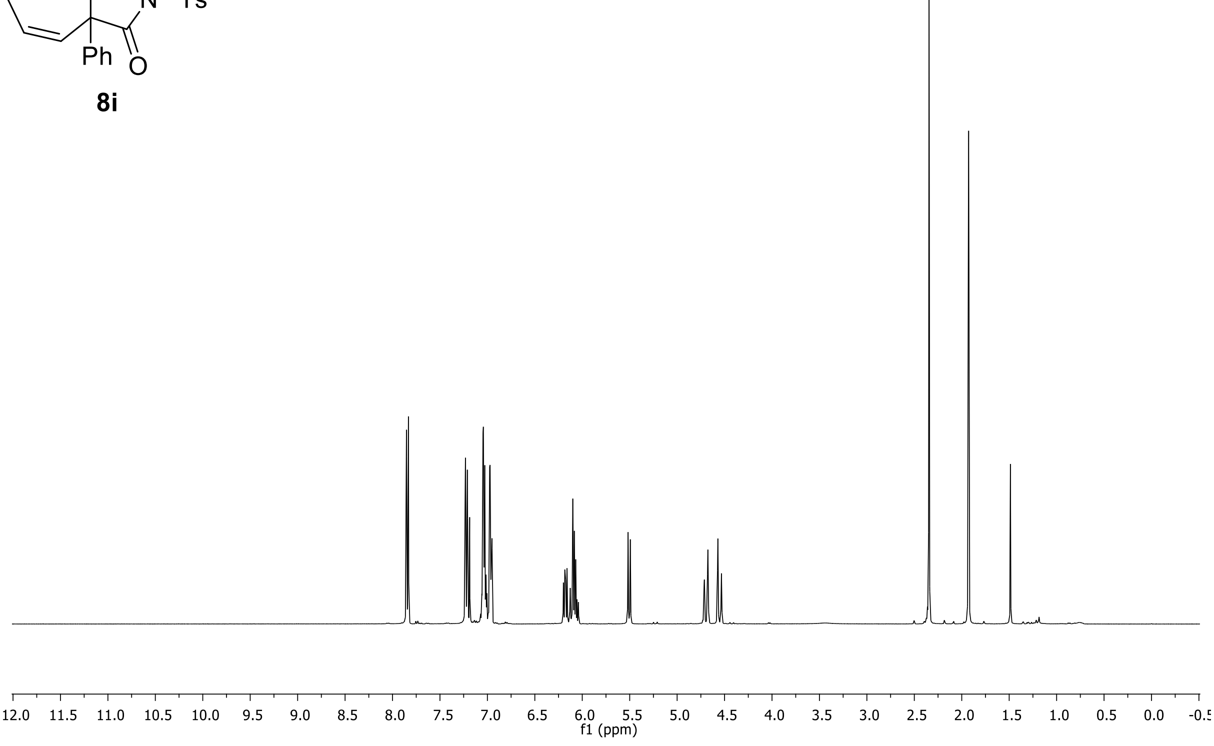
12.0 11.5 11.0 10.5 10.0 9.5 9.0 8.5 8.0 7.5 7.0 6.5 6.0 5.5 5.0 4.5 4.0 3.5 3.0 2.5 2.0 1.5 1.0 0.5 0.0
f1 (ppm)

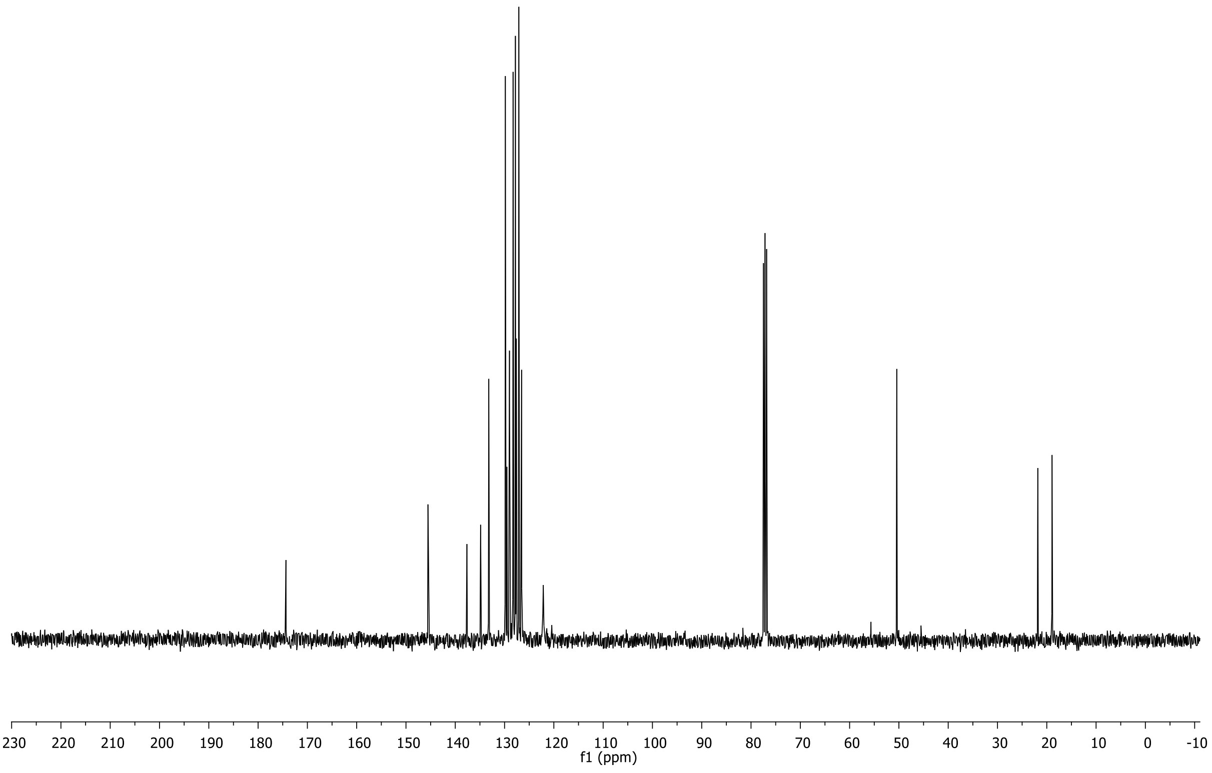
230 220 210 200 190 180 170 160 150 140 130 120 110 100 90 80 70 60 50 40 30 20 10 0 -10
f1 (ppm)

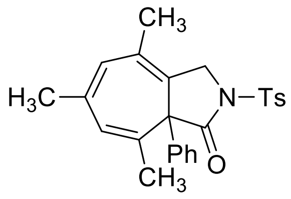
CH3
H3C
N−Ts
Ph
CH3
O

8j

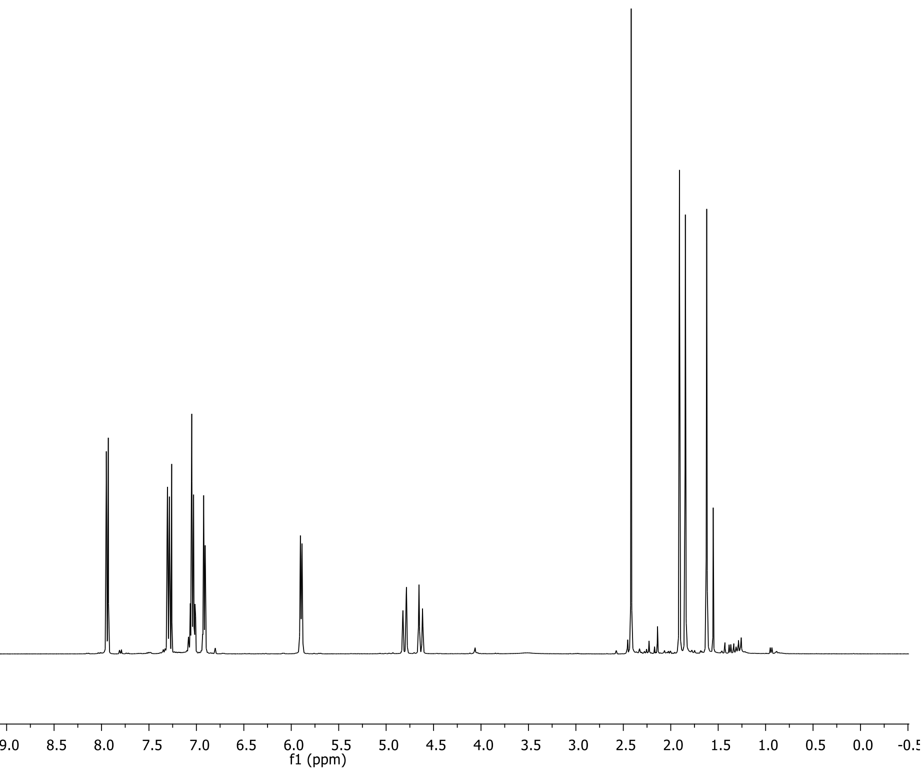
12.0 11.5 11.0 10.5 10.0 9.5 9.0 8.5 8.0 7.5 7.0 6.5 6.0 5.5 5.0 4.5 4.0 3.5 3.0 2.5 2.0 1.5 1.0 0.5 0.0
f1 (ppm)

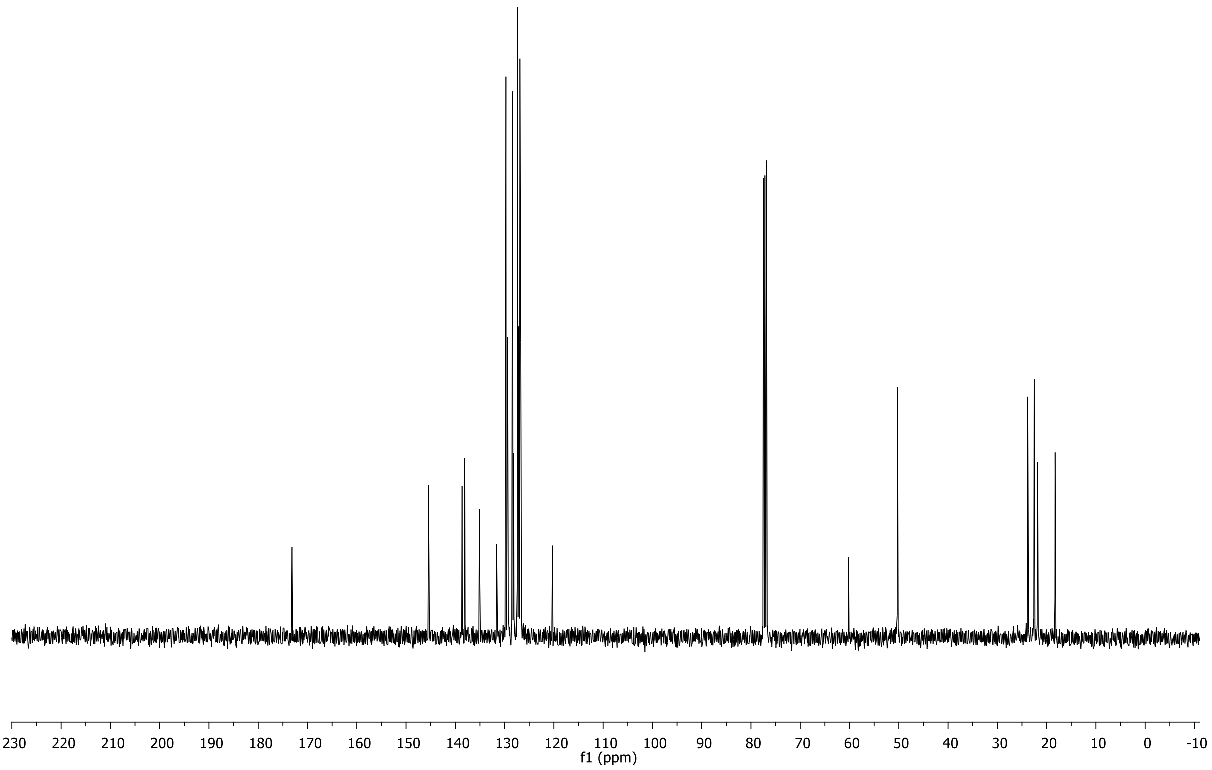
230 220 210 200 190 180 170 160 150 140 130 120 110 100 90 80 70 60 50 40 30 20 10 0 -10
f1 (ppm)

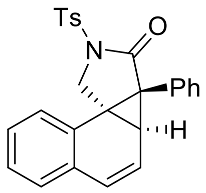
Ts
N
O
Ph
H

8k

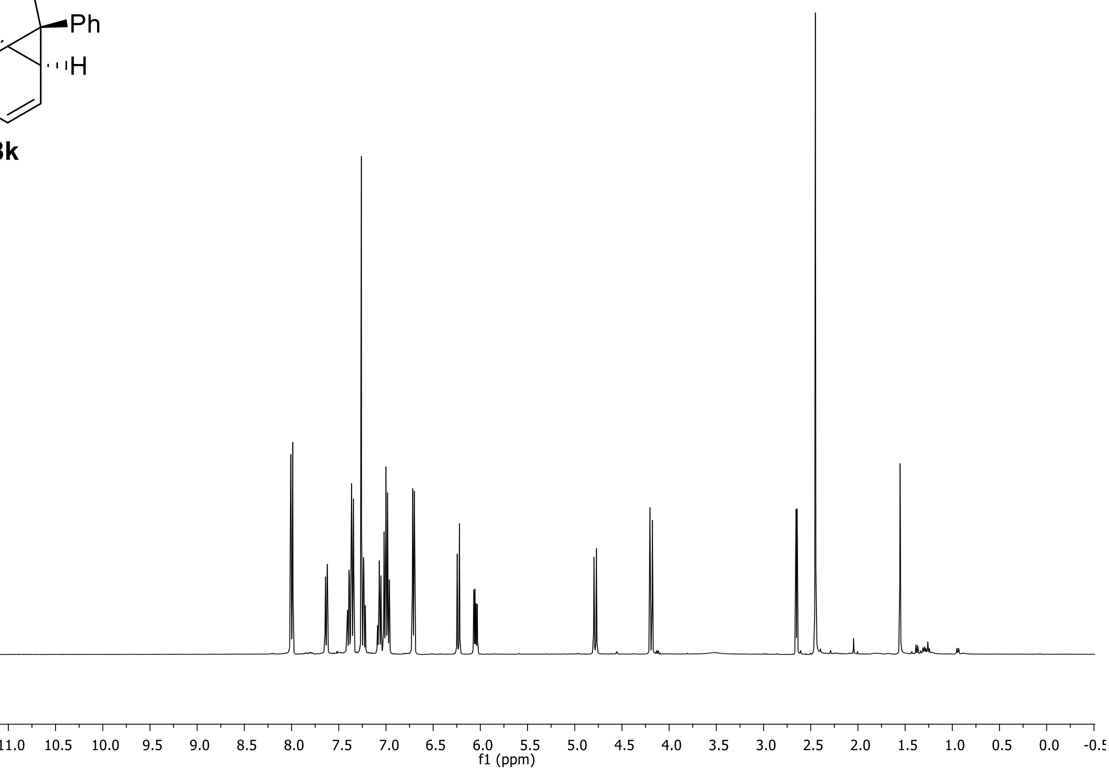
12.0 11.5 11.0 10.5 10.0 9.5 9.0 8.5 8.0 7.5 7.0 6.5 6.0 5.5 5.0 4.5 4.0 3.5 3.0 2.5 2.0 1.5 1.0 0.5 0.0
f1 (ppm)

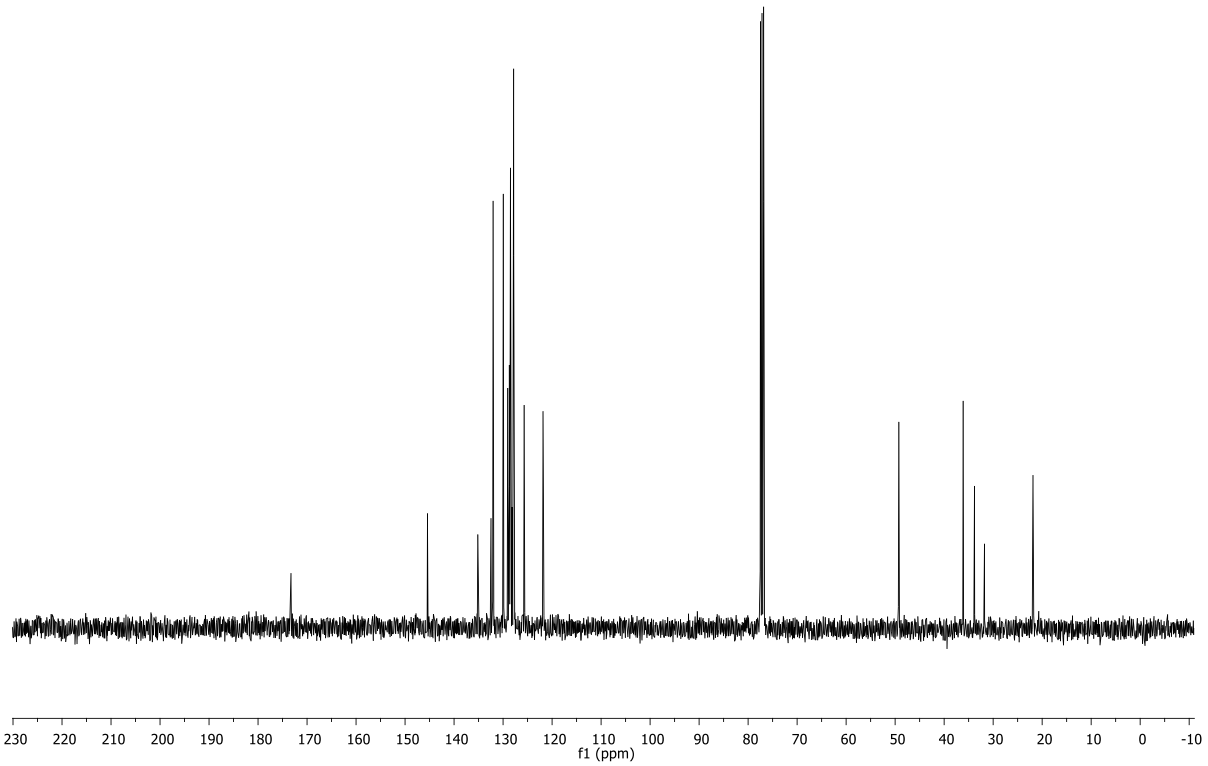
230 220 210 200 190 180 170 160 150 140 130 120 110 100 90 80 70 60 50 40 30 20 10 0 -10
f1 (ppm)

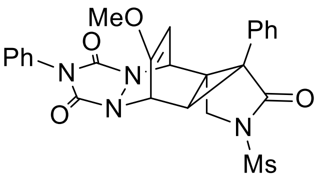

9

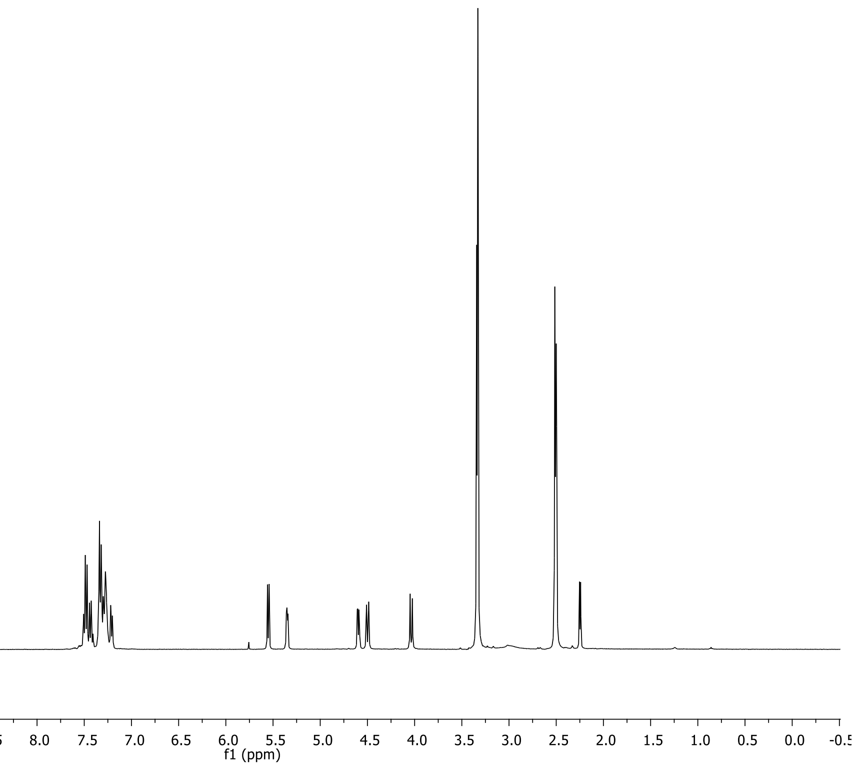

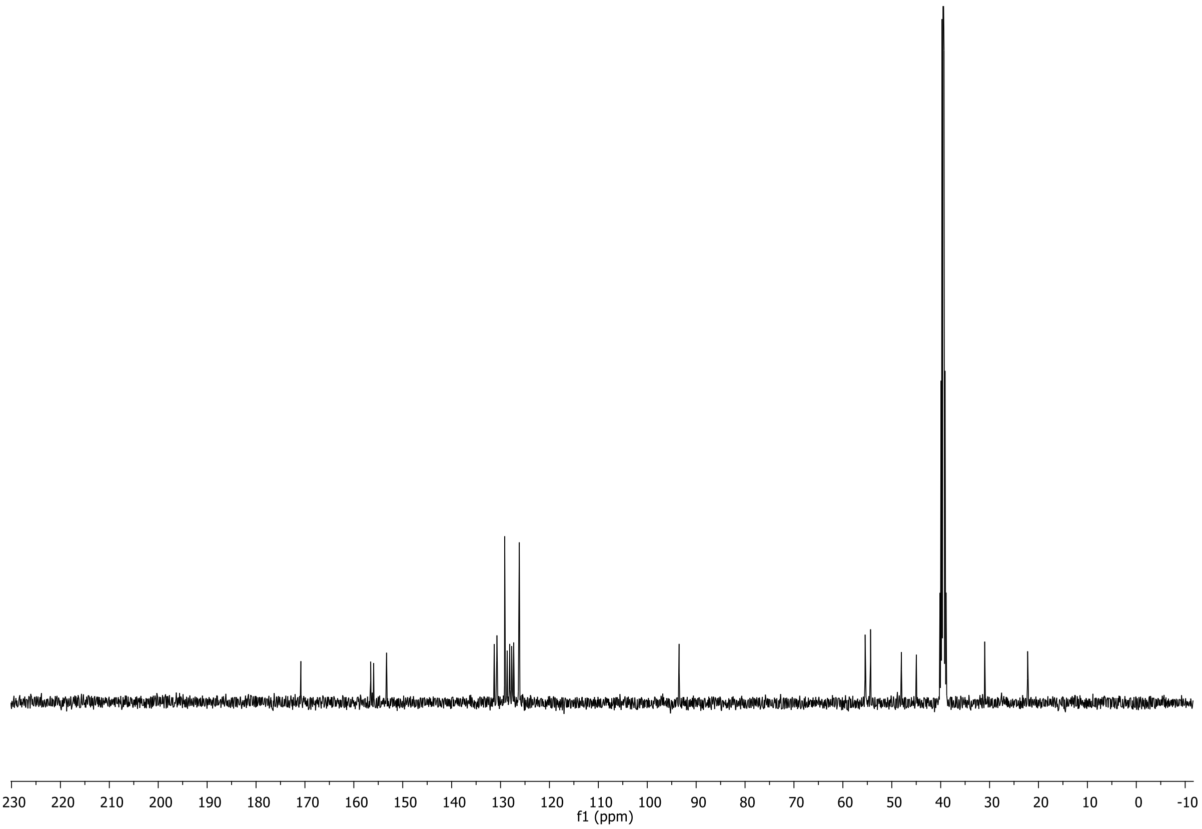

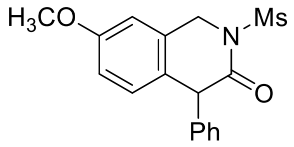
H3CO
N
Ms
O
Ph

10

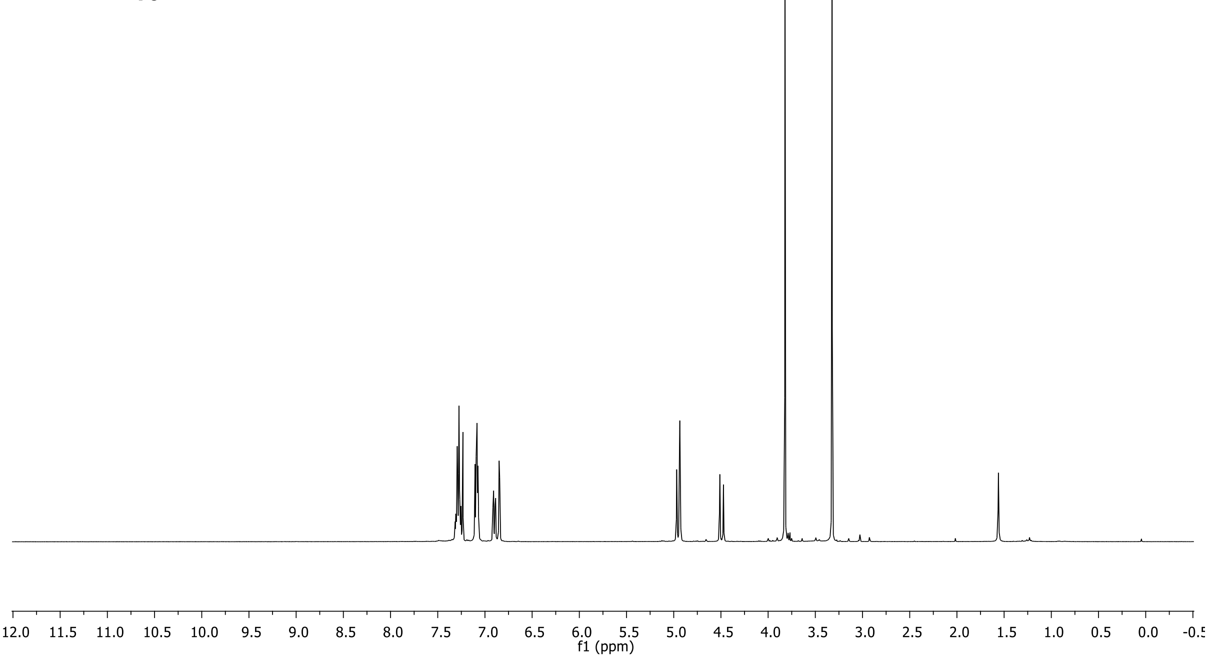
12.0 11.5 11.0 10.5 10.0 9.5 9.0 8.5 8.0 7.5 7.0 6.5 6.0 5.5 5.0 4.5 4.0 3.5 3.0 2.5 2.0 1.5 1.0 0.5 0.0
f1 (ppm)

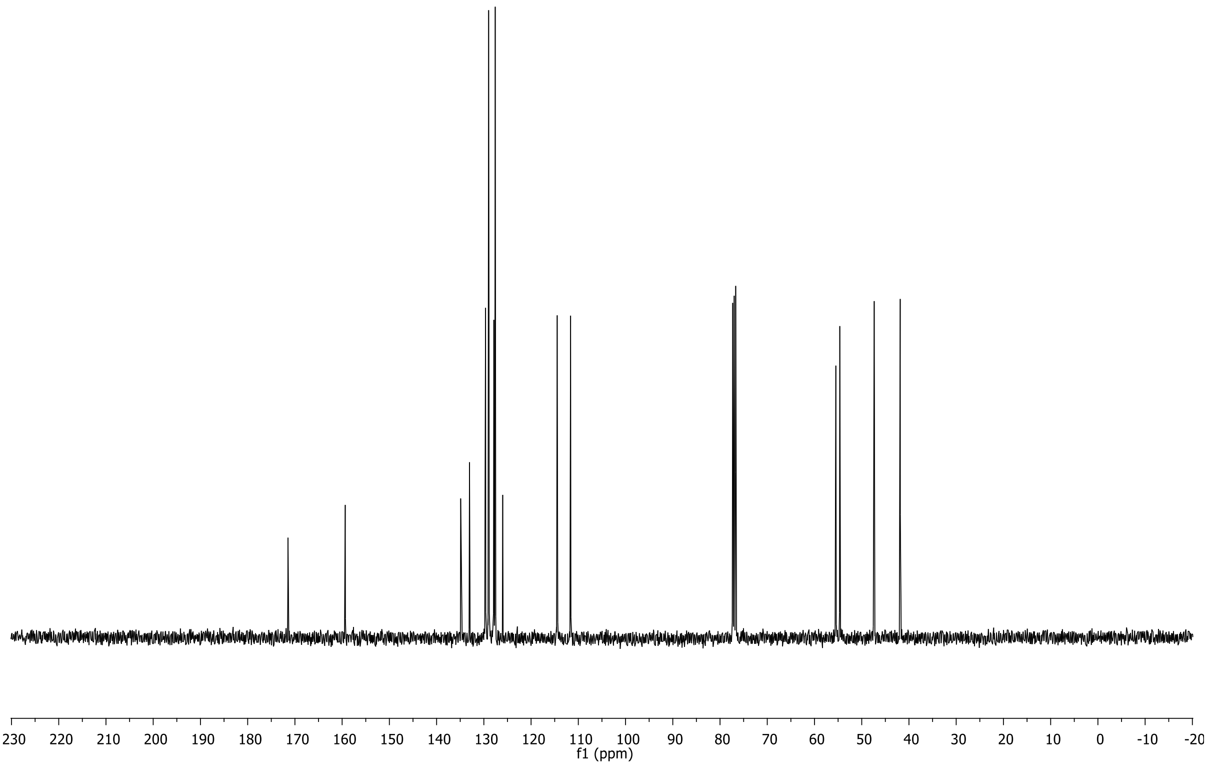
230 220 210 200 190 180 170 160 150 140 130 120 110 100 90 80 70 60 50 40 30 20 10 0 -10 -20
f1 (ppm)

7-Methyl-3-phenyl-indolin-2-one

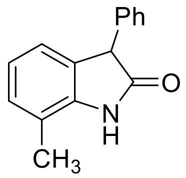

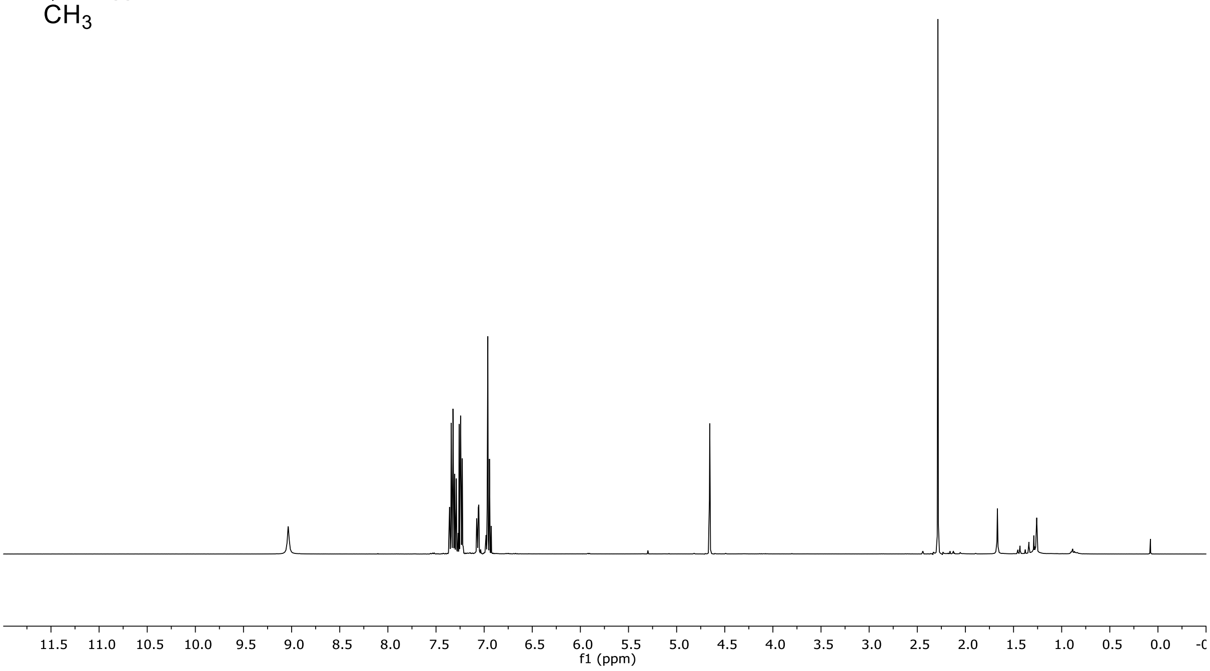

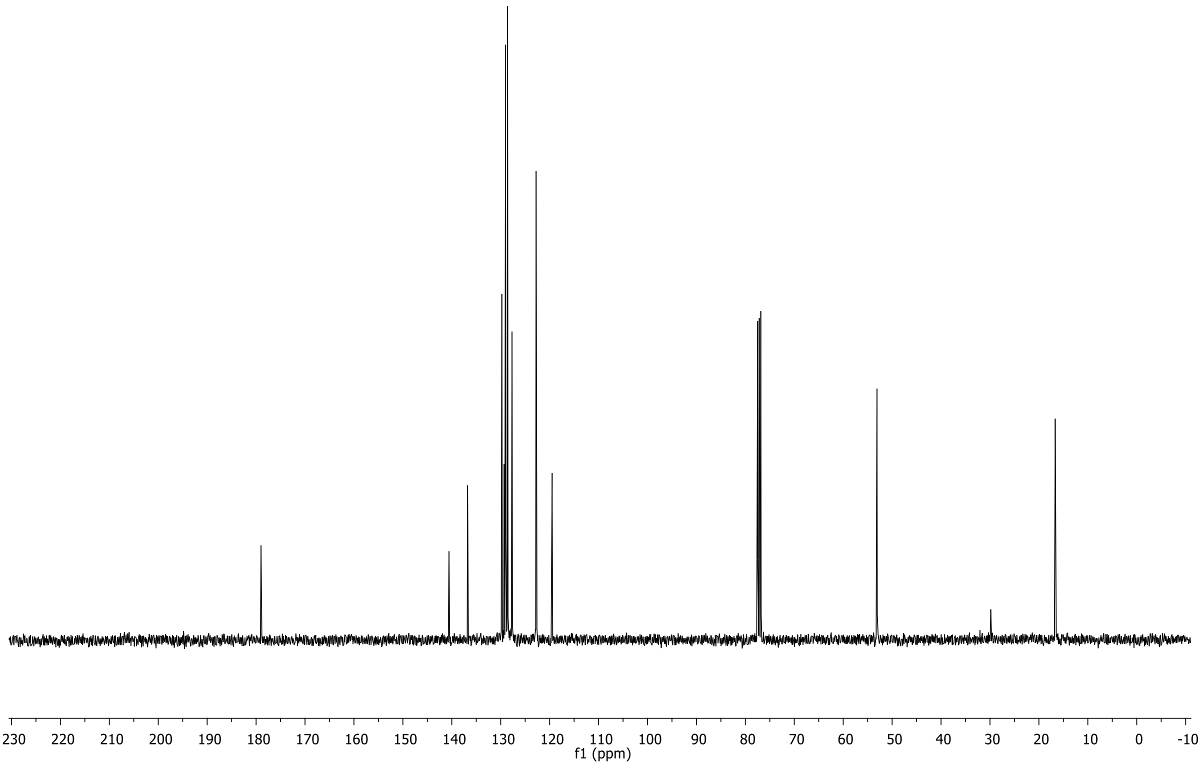

Supplement: Supplementary file 1 — Supplementary [file CHEM-24-17215-s001.pdf]
